# Good neighbors, bad neighbors: the frequent network neighborhood mapping of the hippocampus enlightens several structural factors of the human intelligence on a 414-subject cohort

**DOI:** 10.1038/s41598-020-68914-2

**Published:** 2020-07-20

**Authors:** Máté Fellner, Bálint Varga, Vince Grolmusz

**Affiliations:** 1 PIT Bioinformatics Group, Eötvös University, Budapest, 1117 Hungary; 2 Uratim Ltd., Budapest, 1118 Hungary

**Keywords:** Computational biology and bioinformatics, Neuroscience, Neurology, Mathematics and computing

## Abstract

The human connectome has become the very frequent subject of study of brain-scientists, psychologists and imaging experts in the last decade. With diffusion magnetic resonance imaging techniques, united with advanced data processing algorithms, today we are able to compute braingraphs with several hundred, anatomically identified nodes and thousands of edges, corresponding to the anatomical connections of the brain. The analysis of these graphs without refined mathematical tools is hopeless. These tools need to address the high error rate of the MRI processing workflow, and need to find structural causes or at least correlations of psychological properties and cerebral connections. Until now, structural connectomics was only rarely able of identifying such causes or correlations. In the present work we study the frequent neighbor sets of the most deeply investigated brain area, the hippocampus. By applying the Frequent Network Neighborhood mapping method, we identified frequent neighbor-sets of the hippocampus, which may influence numerous psychological parameters, including intelligence-related ones. We have found “Good Neighbor” sets, which correlate with better test results and also “Bad Neighbor” sets, which correlate with worse test results. Our study utilizes the braingraphs, computed from the imaging data of the Human Connectome Project’s 414 subjects, each with 463 anatomically identified nodes.

## Introduction

Our brain contains approximately 80 billion neurons, each connected to hundreds or even thousands of other neurons. All brain functions are closely connected to this network of the brain, frequently called “the connectome”^1–3^. Today, the neuronal-level connectome (or braingraph), where the nodes correspond to the 80 billion neurons, and two nodes are connected by an edge if the corresponding neurons are connected by an axon, is unknown for us. The only full developed species with known neuronal-level braingraph is that of the nematode *Caenorhabditis elegans*, with 302 neurons, determined in the 80’s by electron-microscopic techniques (^4^, the graph can be downloaded from http://braingraph.org^5^). More recently, serious developments are reported in the mapping of the neuronal-level braingraph of the fruitfly *Drosophila melanogaster* with 100,000 neurons^6^.

With currently available techniques the human braingraph can be constructed and analyzed in a much coarser resolution than the neuronal level, with the help of diffusion magnetic resonance imaging (MRI)^7^. In these graphs, the nodes are anatomically identified $1{-}1.5\,\mathrm{cm}{^2}$ areas of the gray matter (frequently addressed as “ROIs”, i.e., Regions Of Interests), and two nodes are connected by an edge if the diffusion MRI analyzing workflow^7–10^ finds axonal fiber tracts between them. Therefore, we can construct today braingraphs upto 1015 nodes and several thousands edges. One of the most reliable human MRI datasets to date are the public releases of the Human Connectome Project (HCP)^11^.

### The graph-theoretical analysis of the braingraph

The exact, robust and graph-theoretical analysis of the human braingraphs is a fast developing and important area today. Our research group has contributed numerous results in this field, analyzing the HCP data. We have computed hundreds of braingraphs^5^, and prepared the Budapest Reference Connectome Server, which generates the graph of *k*-frequent edges of the human connectome of n = 477 people, where $1\le k\le n$, and the *k*-frequent edges are those, which are present in at least *k* braingraphs out of the n = 477. The parameter *k* is selectable, along with other parameters at the webserver https://pitgroup.org/connectome/, and the resulting consensus graph can be visualized and downloaded from the site^12,13^.

In the work^14^ we have mapped the individually more and less variable lobes of the human brain on 395 subjects, with the help of a natural measure: the distribution function. We have shown that the frontal and the limbic lobes are more conservative, while the edges in the temporal and occipital lobes show more diversity between the individual braingraphs. We have also compared the lobes of the brain by computing numerous graph-theoretical parameters in the sub-graphs, induced by the vertices of the lobes in^15^. We have found that the right temporal and the right parietal lobes have better connectedness-related graph-theoretical parameters, than the left ones (e.g., larger minimum vertex cover, larger Hoffman-bound). More interestingly, the left frontal lobe has better such parameters than the right one.

We have compared the volumetric properties of the male and female brain areas in^16^, and the sex differences in the human brain connectomes in^17–19^. We have shown a strong statistical advantage of the female connectomes in the connectedness-related advanced graph theoretical parameters in a smaller cohort in^17^ and in a larger cohort in^18^. In^19^ we have clarified that the better, connectedness-related braingraph parameter-results of women cannot be due to the brain-volume differences: we have identified 36 large-brain females and 36 small-brain males, such that the brain volumes of all females were larger in the group than those of all males, and the advantage of the women remained valid even after this subject selection.

The development of the connections in the mammal brains is a hot research area today with many open questions. Lots of information were learned from embryonic rat and mouse brain microscopy from the development of single neuronal tracts^20,21^. In human brains much less is known about the phases of the axonal development and growth. By analyzing the features of the publicly available Budapest Reference Connectome Server http://connectome.pitgroup.org, we have discovered the phenomenon of the Consensus Connectome Dynamics (CCD), which, by our hypothesis, describes the individual axonal development of the human brain^22–25^. The CCD phenomenon is also applicable for directing the edges of the braingraph^24,25^.

### Robust methods

The robust analysis of the MR imaging data is an important point in all applications, since there are numerous complex steps, where noise or data processing artifacts may appear in the image processing workflow. For example, one such area is the tractography phase, where the crossing axonal fibers may induce errors in the processing^26–28^. Therefore, the error-correcting analytical methods have an utmost importance in the processing of these data.

Our research group pioneered several such methods, by examining the frequently appearing substructures. This approach will not consider rarely appearing errors, since if we deal with substructures, which appear with a minimum frequency of, say, 80% or 90%, then the infrequent errors will be filtered out. The Budapest Reference Connectome Server generates the *k*-frequent edges^12,13^. In the work^29^ we have mapped the frequently appearing subgraphs of the human connectome. The frequent complete subgraphs of the human braingraph were identified in^30^.

Numerous publications attempt to find correlations between the psychological and anatomical, more exactly, connectomical, or graph theoretical properties of the braingraph (e.g.,^31^). The difficulty of identifying structural-psychological correlations lies in the individual diversity of the cerebral connections. One possible solution to this difficulty is the comparison of the *frequent* substructures with the results of psychological measurements.

In the publication^32^ we defined the Frequent Network Neighborhood Mapping.

### The frequent network neighborhood mapping

Here we would like to formalize the frequent neighborhood mapping. The motivation of the formalism below is identification of the robust, frequent neighborhoods of some important node *u*, where the word “frequent” means that the same neighborhood of *u* appears frequently in the braingraphs of the N subject of ours:

Let *G*(*V*, *E*) be a graph with vertex-set *V* and edge-set *E*. Let *u* be a vertex. Vertex *v* is a neighbor of *u* if the unordered pair $\{u,v\}$ is an edge of *G*. Then $\Gamma (u)$, called the neighbor-set of *u*, contains all the neighbors of vertex *u*, that is:

$$\begin{aligned} \Gamma (u)=\{v\in V: \{u,v\}\in E\}. \end{aligned}$$

Now, let us consider *N* graphs $G_1(V,E_1),G_2(V,E_2),\ldots ,G_N(V,E_N)$ on the very same vertex-set *V*. Let $u\in V$, and let

$$\begin{aligned} \Gamma _i(u)=\{v\in V: \{u,v\}\in E_i\}, \hbox { for } i=1,2,\ldots ,N. \end{aligned}$$

In other words, $\Gamma _i(u)$ is the neighborhood of *u* in graph $G_i$.

We say that the vertex-set $W\subset V$ is a *k*-frequent neighborhood of *u* if there are at least *k* indices *i*, such that $W\subset \Gamma _i(u)$. If, say, $k/N\ge 0.8$, then *W* is a frequent neighbor set of *u* with a cut-off value (or threshold) of 80%.

In the work^32^ we have identified the frequent neighbor sets of the hippocampus of size at most 4, with threshold of 90%. We have also identified the frequent neighbor-sets of the hippocampus, which were more frequent in male and in female subjects, respectively.

### The structural factors of intelligence

Intelligence-related connectomics analyses were published by several authors, e.g.,^33–38^. Most of the previous work on this field applied functional MRI studies, which are usually difficult to reproduce^39^. Here we study structural connectomes, on a large cohort (n = 414), with robust techniques: only the frequent neighbor sets are analyzed. In the present contribution we apply the Frequent Network Neighborhood Mapping method for finding neighbor sets of the hippocampus, which positively or negatively influence some intelligence-measures of the subjects. Since the hippocampus has important roles in the spatial coordination and in turning the short-time memory to long-time memory, it should have a role in performing some intelligence-related tests. The frequent neighbor sets, appearing significantly more frequently with higher scores in these tests, are called “Good Neighbors”. The frequent sets, which appear significantly more frequently with lower scores, are called “Bad Neighbors”.

## Discussion and results

The hippocampus is, perhaps, the most frequently and deeply investigated area of the brain: it is a part of the limbic system, it has a role in turning short-time memory into long-time memory, in spatial orientation, navigation and memory^40–43^. It is a sea-horse-shaped entity, and it is present in the left- and also in the right hemisphere: that is, there are a left- and a right hippocampus in the brain.

Here we identify the frequent hippocampus neighbor sets of size up to 4, for hippocampi in both hemispheres. Next, we investigate whether the presence of these neighbors of the hippocampus have any statistical significance with some, intelligence-related test results of the subjects.

The motivation of this study is as follows: by the best of our knowledge, no connections were proven between the presence or absence of any single connectome-edge and any psychological property of the subjects examined. This failure may be due to the great variability and plasticity of the brain connections^12–14^. Here we want to overcome these difficulties in two-fold strategy:

(i) Instead of the individual appearances of graph-theoretical objects we consider frequent objects;
(ii) Instead of frequent single edges from vertex *u* we consider frequent subsets of the neighbor-set $\Gamma (u)$.

### Measures of intelligence

In the present study we consider two psychological tests, which were administered to the subjects of the Human Connectome Project:

PMAT24_A_CR: Penn Matrix Test: Number of Correct Responses; scored from 0 to 24. This is a multiple choice test where the subject needs to choose the best fit from a list of objects into the one empty position of a small matrix of objects. The PMAT test is believed to assess the mental abstraction and flexibility^44^. The higher scores show better mental abilities. We grouped the scores as “low” between 0 and 16, and “high” between 17 and 24; the cut-off score 17 is the median.

IWRD_TOT: Penn Word Memory Test: Total Number of Correct Responses, scored from 0 to 40. In the first phase of the test, the subjects need to memorize 20 written words. In the recognition phase, 40 words are shown, and the participants need to decide whether the words were seen in the first phase or not. The score is the number of the correct answers. We valued the scores 0–35 as “low” and 36–40 as “high”, the cut-off score 36 is the median.

Table 1 shows the results of the Frequent Network Neighborhood Mapping for these two tests. The table list the numbers of the frequent neighbor sets of the left- and the right hippocampus in the connectomes of the subjects with high- and low PMAT24 and IWRD test scores, respectively.

In the columns, labeled by 1, 2, 3 and 4 the numbers of the 1, 2, 3 and 4-element frequent neighbor-sets are given, for the subjects with high and low test scores. The threshold for “frequent” sets is 80% in the case of the right- and the left hippocampi, and 90% in the case of the union of their neighbor-sets, given in the rows, labelled by “hippocampus”. The column with “sign.” label contains the number of the neighborhood sets of the statistically differing (*p* = 0.01) frequencies in the “low” and the “high” test scores (called briefly “significant sets”). The column with label “sign. for whom” contains the number of the significant sets with higher frequencies in the low and in the high test group, respectively. Note that the sum of the two values of the column with label “sign. for whom” equals to the number in the “sign.” column. In the case of PMAT24 tests, the majority of the significant sets are related to the high test values. This may imply that these neighborhoods of the hippocampus are beneficial for the PMAT24 test results, so, these are the “good neighbors” of the hippocampus.

Some of these “good neighbor” sets of the left hippocampus are listed as follows (we are using the ROI nomenclature at https://github.com/LTS5/cmp_nipype/blob/master/cmtklib/data/parcellation/lausanne2008/ParcellationLausanne2008.xls. The “lh” and the “rh” prefixes abbreviate the “left-hemisphere” and “right-hemisphere” localizations).

Left-Caudate, lh.fusiform_7, lh.inferiorparietal_5, lh.isthmuscingulate_2

or:

Left-Pallidum, lh.lingual_7, lh.superiortemporal_3, lh.transversetemporal_2

The complete list of the “good neighbor” sets of the left hippocampus is available as supplementary Table S1.

In the case of the IWRD test, the majority of the significant sets are related to the low test values. That is, these neighbors are “bad” for the IWRD test results.

Some of the “bad” neighbor sets of the right hippocampus:

rh.insula_4, rh.precuneus_2, rh.precuneus_3, rh.superiortemporal_1

or:

rh.insula_4, rh.precuneus_3, rh.supramarginal_9, rh.transversetemporal_1

All the neighbor-sets of the right hippocampus with significantly higher frequency in subjects with lower-scored IWRD results can be found in supplementary Table S10.

**Table 1:** The table list the numbers of the frequent neighbor sets of the left- and the right hippocampus and their union, labeled by “hippocampus”, in the connectomes of the subjects with high- and low PMAT24 and IWRD test scores, respectively.

		1	2	3	4	Sign.	Sign. for whom	No.
**PMAT24**								
Hippocampus left	High	39	665	6,646	42,854	2,331	2,328	1
Hippocampus left	Low	41	631	5,164	25,824		3	2
Hippocampus right	High	50	873	8,142	48,521	1,788	1,757	3
Hippocampus right	Low	49	817	7,059	37,558		31	4
Hippocampus	High	62	1,325	15,297	113,579	5,345	5,313	5
Hippocampus	Low	54	1,036	10,761	70,252		32	6
**IWRD**								
Hippocampus left	High	39	637	5,684	31,139	963	0	7
Hippocampus left	Low	41	691	6,675	41,200		963	8
Hippocampus right	High	47	833	7,663	43,337	456	41	9
Hippocampus right	Low	49	850	7,705	43,918		415	10
Hippocampus	High	55	1,082	11,219	72,613	5,484	0	11
Hippocampus	Low	62	1,307	15,077	114,860		5,484	12

In the columns, labeled by 1, 2, 3 and 4 the numbers of the 1, 2, 3 and 4-element frequent neighbor-sets are given, for the subjects with high and low test scores. The threshold for “frequent” sets is 80% in the case of the right- and left hippocampi, and 90% in the case of the hippocampus (where we consider the union of the neighbors of the right and the left hippocampi). The column with “sign.” label contains the number of the neighborhood sets of the statistically significantly differing ($p=$ 0.01) frequencies in the “low” and the “high” test scores (called briefly “significant sets”). The column with label “sign. for whom” contains the number of the significant sets with higher frequencies in the low and in the high test group. Note that the sum of the two values of the column with label “sign. for whom” equals to the number in the “sign.” column. In the case of PMAT24 tests, the majority of the significant sets are related to the high test values. In the case of the IWRD test, the majority of the significant sets are related to the low test values. The last column, labeled by “No.”, contains the reference number to the listing of the significant sets in the supplementary material: Table Sx contains the list of the significant sets, corresponding to the row, with reference *x* (where $x=1,2,\ldots ,12$). The supplementary tables can be downloaded in Excel format from http://uratim.com/hintell/tables.zip.

## Materials and methods

The braingraphs in our work was computed from the Human Connectome Project’s (HCP) Public Data Release at http://www.humanconnectome.org/documentation/S500^11^. The data set applied in this study contains the diffusion MRI recordings of 500 healthy human subjects of age 22–35 years. The details of the HCP data acquisition pipeline and the subjects are available at https://www.humanconnectome.org/storage/app/media/documentation/s500/hcps500meg2releasereferencemanual.pdf.

The workflow, by which the graphs were computed by our group from the HCP data set, is described in detail in^5^. In short, we applied the CMTK toolkit^10^ including the FreeSurfer tool^8^ and the MRtrix tractography program^45^. The tractography applied random seeding and the deterministic streamline method with 1 million streamlines. The parcellation labels were specified in the CMTK suite, in the nypipe GitHub repository at the address https://github.com/LTS5/cmp_nipype/blob/master/cmtklib/data/parcellation/lausanne2008/ParcellationLausanne2008.xls.

We were able to complete the braingraph computations for 413 subjects (238 women and 175 men). The graphs are available freely for download at the site: https://braingraph.org/cms/download-pit-group-connectomes/. In this work we have applied unweighted graphs with 463 nodes.

The computation of the frequent neighbor sets of the hippocampus, which facilitated the Frequent Network Neighborhood Mapping, used an apriori-like algorithm^46,47^, with small modifications: http://adataanalyst.com/machine-learning/apriori-algorithm-python-3-0/. The details of the frequent neighbor set mapping is described in detail in^32^.

The statistical analysis used a $\chi ^2$ test with significance bound of $p=0.01$, with Holm-Bonferroni corrections^48^.

## Conclusions

By the application of Frequent Network Neighborhood Mapping, we examined the neighbors of the human hippocampus, and found that some frequent neighbor sets correlate with the better PMAT24 test results, and some frequent neighbor sets correlate with worse IWRD test results. By our knowledge, this is the first demonstration, which statistically connects the intelligence-related test measures with the neighbor-sets of the human hippocampus. Our results are robust, since we have considered only the frequent neighbor sets, therefore, small errors in the data acquisition and processing workflow do not influence our results. We have used a strong p=0.01 significance bound, as an additional robustness precaution.

## Supplementary information

Supplementary Information 1.

Supplementary Information 2.

Supplementary Information 3.

Supplementary Information 4.

Supplementary Information 5.

Supplementary Information 6.

Supplementary Information 7.

Supplementary Information 8.

Supplementary Information 9.

Supplementary Information 10.

Supplementary Information 11.

Supplementary Information 12.

## Data Availability

The data source of this study is Human Connectome Project’s Public Data Release at http://www.humanconnectome.org/documentation/S500^11^. The parcellation data, containing the ROI labels, is listed in the CMTK nypipe GitHub repository https://github.com/LTS5/cmp_nipype/blob/master/cmtklib/data/parcellation/lausanne2008/ParcellationLausanne2008.xls. The braingraphs, computed by our group, can be downloaded from the https://braingraph.org/cms/download-pit-group-connectomes/ site, by choosing the “Full set, 413 brains, 1 million streamlines” option. In the present study we have used exclusively the 463-node resolution graphs. The significant neighbor sets of the hippocampus are listed in 12 supplementary tables (two of which are intentionally left empty), numbered from Table S1 through Table S12; the numbers, following the S letter, correspond to the reference number in the last column of Table 1. The supplementary tables in MS Excel form can be downloaded as a zip file from http://uratim.com/hintell/tables.zip.
