# Supplementary material for: Good neighbors, bad neighbors: the frequent network neighborhood mapping of the hippocampus enlightens several structural factors of the human intelligence on a 414-subject cohort
Source: Sci Rep. 2020 Jul 20;10:11967. doi: 10.1038/s41598-020-68914-2 (PMC7371878; doi:10.1038/s41598-020-68914-2)
Supplement: Supplementary file 1 — Supplementary Information 1. [file 41598_2020_68914_MOESM1_ESM.pdf]

| p-value | Holm-Bonferroni | frequency_upper | frequency_lower | name |
|---|---|---|---|---|
| 5.00E-05 | 0 | 0.92164 | 0.79327 | (Left-Caudate)(lh.fusiform_7)(lh.inferiorparietal_5)(lh.isthmuscingulate_2) |
| 7.00E-05 | 0 | 0.94403 | 0.83173 | (Left-Caudate)(lh.inferiorparietal_5)(lh.isthmuscingulate_2)(lh.superiortemporal_3) |
| 0.00012 | 0 | 0.92537 | 0.80769 | (Left-Caudate)(lh.fusiform_7)(lh.isthmuscingulate_2)(lh.superiortemporal_3) |
| 0.00012 | 0 | 0.92537 | 0.80769 | (Left-Caudate)(lh.isthmuscingulate_2)(lh.superiortemporal_3)(lh.transversetemporal_2) |
| 0.00013 | 0 | 0.92164 | 0.80288 | (Left-Caudate)(lh.fusiform_7)(lh.inferiorparietal_5)(lh.superiortemporal_3) |
| 0.00015 | 0 | 0.95522 | 0.85577 | (Left-Caudate)(Left-Putamen)(lh.isthmuscingulate_2)(lh.superiortemporal_3) |
| 0.00016 | 0 | 0.93657 | 0.82692 | (Left-Caudate)(lh.inferiorparietal_5)(lh.isthmuscingulate_2)(lh.parahippocampal_3) |
| 0.00017 | 0 | 0.95149 | 0.85096 | (Left-Caudate)(Left-Putamen)(lh.inferiorparietal_5)(lh.superiortemporal_3) |
| 0.00017 | 0 | 0.95149 | 0.85096 | (Left-Caudate)(Left-Putamen)(lh.inferiorparietal_5)(lh.isthmuscingulate_2) |
| 0.00018 | 0 | 0.93284 | 0.82212 | (Left-Caudate)(lh.fusiform_7)(lh.isthmuscingulate_2) |
| 0.00018 | 0 | 0.93284 | 0.82212 | (Left-Caudate)(lh.fusiform_7)(lh.isthmuscingulate_2)(lh.superiortemporal_2) |
| 0.00018 | 0 | 0.93284 | 0.82212 | (Left-Caudate)(Left-Putamen)(lh.fusiform_7)(lh.isthmuscingulate_2) |
| 0.00018 | 0 | 0.93284 | 0.82212 | (Left-Caudate)(Left-Thalamus-Proper)(lh.fusiform_7)(lh.isthmuscingulate_2) |
| 0.00018 | 0 | 0.93284 | 0.82212 | (Left-Caudate)(lh.fusiform_7)(lh.isthmuscingulate_2)(lh.isthmuscingulate_3) |
| 0.00019 | 0 | 0.9291 | 0.81731 | (Left-Caudate)(lh.fusiform_7)(lh.inferiorparietal_5)(lh.superiortemporal_2) |
| 0.00021 | 0 | 0.92537 | 0.8125 | (Left-Caudate)(Left-Pallidum)(lh.fusiform_7)(lh.isthmuscingulate_2) |
| 0.00022 | 0 | 0.87313 | 0.74074 | (Left-Caudate)(lh.lingual_7)(lh.superiortemporal_3)(lh.transversetemporal_2) |
| 0.00023 | 0 | 0.92164 | 0.80769 | (Left-Caudate)(lh.inferiorparietal_5)(lh.superiortemporal_3)(lh.transversetemporal_2) |
| 0.00024 | 0 | 0.91791 | 0.80288 | (Left-Caudate)(lh.fusiform_7)(lh.isthmuscingulate_2)(lh.lingual_6) |
| 0.00024 | 0 | 0.91791 | 0.80288 | (Left-Caudate)(lh.fusiform_7)(lh.isthmuscingulate_2)(lh.parahippocampal_3) |
| 0.00025 | 0 | 0.9403 | 0.83654 | (Left-Caudate)(Left-Pallidum)(lh.isthmuscingulate_2)(lh.superiortemporal_3) |
| 0.00025 | 0 | 0.91418 | 0.79808 | (Left-Caudate)(Left-Pallidum)(lh.isthmuscingulate_2)(lh.transversetemporal_2) |
| 0.00025 | 0 | 0.91418 | 0.79808 | (Left-Caudate)(lh.isthmuscingulate_2)(lh.parahippocampal_3)(lh.transversetemporal_2) |
| 0.00026 | 0 | 0.95522 | 0.86058 | (Left-Caudate)(lh.isthmuscingulate_2)(lh.superiortemporal_3) |
| 0.00026 | 0 | 0.95522 | 0.86058 | (Left-Caudate)(lh.isthmuscingulate_2)(lh.isthmuscingulate_3)(lh.superiortemporal_3) |
| 0.00026 | 0 | 0.95522 | 0.86058 | (Left-Caudate)(Left-Thalamus-Proper)(lh.isthmuscingulate_2)(lh.superiortemporal_3) |
| 0.00026 | 0 | 0.95522 | 0.86058 | (Left-Caudate)(lh.isthmuscingulate_2)(lh.superiortemporal_2)(lh.superiortemporal_3) |
| 0.00027 | 0 | 0.91045 | 0.79327 | (Left-Caudate)(lh.fusiform_7)(lh.isthmuscingulate_2)(lh.precuneus_11) |
| 0.00028 | 0 | 0.93657 | 0.83173 | (Left-Caudate)(Left-Pallidum)(lh.inferiorparietal_5)(lh.superiortemporal_3) |
| 0.00028 | 0 | 0.90672 | 0.78846 | (Left-Caudate)(lh.fusiform_7)(lh.superiortemporal_2)(lh.transversetemporal_2) |
| 0.00029 | 0 | 0.90299 | 0.78365 | (Left-Caudate)(Left-Pallidum)(lh.fusiform_7)(lh.transversetemporal_2) |
| 0.0003 | 0 | 0.93284 | 0.82692 | (Left-Caudate)(Left-Putamen)(lh.superiortemporal_3)(lh.transversetemporal_2) |
| 0.0003 | 0 | 0.93284 | 0.82692 | (Left-Caudate)(Left-Pallidum)(lh.inferiorparietal_5)(lh.isthmuscingulate_2) |

| | | | | |
|---|---|---|---|---|
| 0.0003 | 0 | 0.95149 | 0.85577 | (Left-Caudate)(lh.inferiorparietal_5)(lh.superiortemporal_2)(lh.superiortemporal_3) |
| 0.0003 | 0 | 0.95149 | 0.85577 | (Left-Caudate)(lh.inferiorparietal_5)(lh.superiortemporal_3) |
| 0.0003 | 0 | 0.95149 | 0.85577 | (Left-Caudate)(Left-Thalamus-Proper)(lh.inferiorparietal_5)(lh.superiortemporal_3) |
| 0.0003 | 0 | 0.95149 | 0.85577 | (Left-Caudate)(lh.inferiorparietal_5)(lh.isthmuscingulate_3)(lh.superiortemporal_3) |
| 0.00032 | 0 | 0.83209 | 0.69231 | (Left-Accumbens-area)(Left-Caudate)(Left-Pallidum)(lh.transversetemporal_2) |
| 0.00033 | 0 | 0.9291 | 0.82212 | (Left-Caudate)(lh.fusiform_7)(lh.inferiorparietal_5)(lh.isthmuscingulate_3) |
| 0.00033 | 0 | 0.9291 | 0.82212 | (Left-Caudate)(lh.fusiform_7)(lh.insula_2)(lh.isthmuscingulate_2) |
| 0.00033 | 0 | 0.9291 | 0.82212 | (Left-Caudate)(Left-Putamen)(lh.fusiform_7)(lh.inferiorparietal_5) |
| 0.00033 | 0 | 0.9291 | 0.82212 | (Left-Caudate)(Left-Pallidum)(lh.fusiform_7)(lh.superiortemporal_3) |
| 0.00033 | 0 | 0.9291 | 0.82212 | (Left-Caudate)(Left-Putamen)(lh.isthmuscingulate_2)(lh.transversetemporal_2) |
| 0.00033 | 0 | 0.9291 | 0.82212 | (Left-Caudate)(lh.fusiform_7)(lh.inferiorparietal_5) |
| 0.00033 | 0 | 0.9291 | 0.82212 | (Left-Caudate)(Left-Thalamus-Proper)(lh.fusiform_7)(lh.inferiorparietal_5) |
| 0.00033 | 0 | 0.84328 | 0.70673 | (Left-Accumbens-area)(Left-Caudate)(lh.inferiorparietal_5)(lh.isthmuscingulate_2) |
| 0.00034 | 0 | 0.94776 | 0.85096 | (Left-Caudate)(Left-Putamen)(lh.isthmuscingulate_2)(lh.parahippocampal_3) |
| 0.00037 | 0 | 0.92164 | 0.8125 | (Left-Caudate)(Left-Pallidum)(lh.fusiform_7)(lh.inferiorparietal_5) |
| 0.00039 | 0 | 0.91791 | 0.80769 | (Left-Caudate)(lh.inferiorparietal_5)(lh.isthmuscingulate_2)(lh.transversetemporal_2) |
| 0.00039 | 0 | 0.91791 | 0.80769 | (Left-Caudate)(Left-Pallidum)(lh.superiortemporal_3)(lh.transversetemporal_2) |
| 0.00041 | 0 | 0.91418 | 0.80288 | (Left-Caudate)(lh.fusiform_7)(lh.inferiorparietal_5)(lh.lingual_6) |
| 0.00041 | 0 | 0.91418 | 0.80288 | (Left-Caudate)(lh.fusiform_7)(lh.inferiorparietal_5)(lh.parahippocampal_3) |
| 0.00043 | 0 | 0.9403 | 0.84135 | (Left-Caudate)(lh.isthmuscingulate_2)(lh.parahippocampal_3)(lh.superiortemporal_3) |
| 0.00045 | 0 | 0.90672 | 0.79327 | (Left-Caudate)(lh.fusiform_7)(lh.transversetemporal_2) |
| 0.00045 | 0 | 0.90672 | 0.79327 | (Left-Caudate)(lh.fusiform_7)(lh.isthmuscingulate_3)(lh.transversetemporal_2) |
| 0.00045 | 0 | 0.90672 | 0.79327 | (Left-Caudate)(Left-Putamen)(lh.fusiform_7)(lh.transversetemporal_2) |
| 0.00045 | 0 | 0.90672 | 0.79327 | (Left-Caudate)(lh.fusiform_7)(lh.insula_2)(lh.transversetemporal_2) |
| 0.00045 | 0 | 0.90672 | 0.79327 | (Left-Caudate)(Left-Thalamus-Proper)(lh.fusiform_7)(lh.transversetemporal_2) |
| 0.00047 | 0 | 0.93657 | 0.83654 | (Left-Caudate)(lh.inferiorparietal_5)(lh.parahippocampal_3)(lh.superiortemporal_3) |
| 0.00047 | 0 | 0.90299 | 0.78846 | (Left-Caudate)(lh.fusiform_7)(lh.isthmuscingulate_2)(lh.parahippocampal_1) |
| 0.00048 | 0 | 0.82836 | 0.69231 | (Left-Accumbens-area)(Left-Caudate)(lh.superiortemporal_3)(lh.transversetemporal_2) |
| 0.0005 | 0 | 0.83955 | 0.70673 | (Left-Accumbens-area)(Left-Pallidum)(lh.isthmuscingulate_2)(lh.transversetemporal_2) |
| 0.0005 | 0 | 0.93284 | 0.83173 | (Left-Caudate)(lh.fusiform_7)(lh.superiortemporal_2)(lh.superiortemporal_3) |
| 0.0005 | 0 | 0.93284 | 0.83173 | (Left-Caudate)(lh.fusiform_7)(lh.isthmuscingulate_3)(lh.superiortemporal_3) |
| 0.0005 | 0 | 0.93284 | 0.83173 | (Left-Caudate)(lh.fusiform_7)(lh.insula_2)(lh.superiortemporal_3) |
| 0.0005 | 0 | 0.93284 | 0.83173 | (Left-Caudate)(lh.superiortemporal_2)(lh.superiortemporal_3)(lh.transversetemporal_2) |
| 0.0005 | 0 | 0.93284 | 0.83173 | (Left-Caudate)(lh.isthmuscingulate_3)(lh.superiortemporal_3)(lh.transversetemporal_2) |
