# Supplementary material for: Good neighbors, bad neighbors: the frequent network neighborhood mapping of the hippocampus enlightens several structural factors of the human intelligence on a 414-subject cohort
Source: Sci Rep. 2020 Jul 20;10:11967. doi: 10.1038/s41598-020-68914-2 (PMC7371878; doi:10.1038/s41598-020-68914-2)
Supplement: Supplementary file 2 — Supplementary Information 2. [file 41598_2020_68914_MOESM2_ESM.pdf]

p-value	Holm-Bonferroni	frequency_upper	frequency_lower	name
0.00446	1.00E-05	0.76761	0.87019	(lh.bankssts_3)(lh.fusiform_5)(lh.inferiorparietal_4)(lh.insula_1)
0.00823	2.00E-05	0.78169	0.875	(lh.bankssts_3)(lh.insula_1)(lh.lateraloccipital_9)(lh.lingual_8)
0.00966	7.00E-05	0.79577	0.88462	(lh.bankssts_3)(lh.fusiform_5)(lh.inferiorparietal_4)(lh.lingual_8)
