# Supplementary material for: Good neighbors, bad neighbors: the frequent network neighborhood mapping of the hippocampus enlightens several structural factors of the human intelligence on a 414-subject cohort
Source: Sci Rep. 2020 Jul 20;10:11967. doi: 10.1038/s41598-020-68914-2 (PMC7371878; doi:10.1038/s41598-020-68914-2)
Supplement: Supplementary file 3 — Supplementary Information 3. [file 41598_2020_68914_MOESM3_ESM.pdf]

p-value	Holm-Bonferroni	frequency_upper	frequency_lower	name
8.00E-05	1.00E-05	0.83209	0.67788	(rh.fusiform_7)(rh.inferiorparietal_9)(rh.parahippocampal_3)(rh.precuneus_2)
9.00E-05	1.00E-05	0.84701	0.69712	(rh.fusiform_7)(rh.inferiorparietal_9)(rh.precuneus_2)(rh.superiortemporal_3)
9.00E-05	1.00E-05	0.85075	0.70192	(rh.bankssts_2)(rh.fusiform_7)(rh.inferiorparietal_9)(rh.precuneus_2)
9.00E-05	1.00E-05	0.85075	0.70192	(rh.fusiform_7)(rh.inferiorparietal_9)(rh.lingual_7)(rh.precuneus_2)
0.00012	1.00E-05	0.81343	0.65865	(rh.entorhinal_1)(rh.fusiform_7)(rh.inferiorparietal_9)(rh.precuneus_2)
0.00014	1.00E-05	0.84328	0.69712	(rh.fusiform_7)(rh.inferiorparietal_9)(rh.parahippocampal_2)(rh.precuneus_2)
0.00014	1.00E-05	0.85075	0.70673	(rh.fusiform_7)(rh.inferiorparietal_9)(rh.insula_2)(rh.precuneus_2)
0.00014	1.00E-05	0.85075	0.70673	(rh.fusiform_7)(rh.inferiorparietal_9)(rh.isthmuscingulate_2)(rh.precuneus_2)
0.00014	1.00E-05	0.85075	0.70673	(Right-Thalamus-Proper)(rh.fusiform_7)(rh.inferiorparietal_9)(rh.precuneus_2)
0.00014	1.00E-05	0.85075	0.70673	(Right-Putamen)(rh.fusiform_7)(rh.inferiorparietal_9)(rh.precuneus_2)
0.00014	1.00E-05	0.85075	0.70673	(rh.fusiform_7)(rh.inferiorparietal_9)(rh.precuneus_2)
0.00018	1.00E-05	0.89925	0.77404	(rh.inferiorparietal_9)(rh.parahippocampal_2)(rh.precuneus_2)(rh.superiortemporal_3)
0.00021	1.00E-05	0.83582	0.69231	(rh.fusiform_7)(rh.inferiorparietal_9)(rh.precuneus_2)(rh.supramarginal_9)
0.00021	1.00E-05	0.83955	0.69712	(Right-Caudate)(rh.fusiform_7)(rh.inferiorparietal_9)(rh.precuneus_2)
0.00022	1.00E-05	0.84701	0.70673	(Right-Pallidum)(rh.fusiform_7)(rh.inferiorparietal_9)(rh.precuneus_2)
0.00028	1.00E-05	0.90672	0.78846	(rh.inferiorparietal_9)(rh.lingual_7)(rh.precuneus_2)(rh.superiortemporal_3)
0.00028	1.00E-05	0.90672	0.78846	(rh.bankssts_2)(rh.inferiorparietal_9)(rh.precuneus_2)(rh.superiortemporal_3)
0.00032	1.00E-05	0.83209	0.69231	(rh.fusiform_7)(rh.inferiorparietal_9)(rh.parahippocampal_3)(rh.superiortemporal_3)
0.00032	1.00E-05	0.83582	0.69712	(rh.bankssts_2)(rh.fusiform_7)(rh.inferiorparietal_9)(rh.parahippocampal_3)
0.00032	1.00E-05	0.83582	0.69712	(rh.fusiform_7)(rh.inferiorparietal_9)(rh.lingual_7)(rh.parahippocampal_3)
0.00033	1.00E-05	0.83955	0.70192	(rh.fusiform_7)(rh.inferiorparietal_9)(rh.insula_5)(rh.precuneus_2)
0.00033	1.00E-05	0.84328	0.70673	(rh.fusiform_7)(rh.inferiorparietal_9)(rh.precuneus_2)(rh.superiortemporal_9)
0.00034	1.00E-05	0.85075	0.71635	(rh.bankssts_2)(rh.fusiform_7)(rh.inferiorparietal_9)(rh.superiortemporal_3)
0.00034	1.00E-05	0.85075	0.71635	(rh.fusiform_7)(rh.inferiorparietal_9)(rh.lingual_7)(rh.superiortemporal_3)
0.00034	1.00E-05	0.85448	0.72115	(rh.bankssts_2)(rh.fusiform_7)(rh.inferiorparietal_9)(rh.lingual_7)
0.00036	1.00E-05	0.91045	0.7963	(rh.inferiorparietal_9)(rh.lingual_7)(rh.superiortemporal_3)
0.00041	1.00E-05	0.90299	0.78704	(rh.inferiorparietal_9)(rh.parahippocampal_2)(rh.superiortemporal_3)
0.00042	1.00E-05	0.8097	0.66827	(rh.entorhinal_1)(rh.fusiform_7)(rh.inferiorparietal_9)(rh.parahippocampal_3)
0.00044	1.00E-05	0.81343	0.67308	(rh.entorhinal_1)(rh.fusiform_7)(rh.inferiorparietal_9)(rh.superiortemporal_3)
0.00045	1.00E-05	0.81716	0.67788	(rh.bankssts_2)(rh.entorhinal_1)(rh.fusiform_7)(rh.inferiorparietal_9)
0.00045	1.00E-05	0.81716	0.67788	(rh.fusiform_7)(rh.fusiform_8)(rh.inferiorparietal_9)(rh.parahippocampal_3)
0.00045	1.00E-05	0.81716	0.67788	(rh.entorhinal_1)(rh.fusiform_7)(rh.inferiorparietal_9)(rh.lingual_7)
0.00045	1.00E-05	0.90672	0.79327	(rh.inferiorparietal_9)(rh.isthmuscingulate_2)(rh.precuneus_2)(rh.superiortemporal_3)
0.00045	1.00E-05	0.90672	0.79327	(Right-Putamen)(rh.inferiorparietal_9)(rh.precuneus_2)(rh.superiortemporal_3)

0.00045	1.00E-05	0.90672	0.79327	(Right-Thalamus-Proper)(rh.inferiorparietal_9)(rh.precuneus_2)(rh.superiortemporal_3)
0.00046	1.00E-05	0.8209	0.68269	(rh.fusiform_7)(rh.fusiform_8)(rh.inferiorparietal_9)(rh.precuneus_2)
0.00047	1.00E-05	0.90299	0.78846	(rh.bankssts_2)(rh.fusiform_7)(rh.parahippocampal_3)(rh.precuneus_2)
0.00047	1.00E-05	0.90299	0.78846	(rh.fusiform_7)(rh.lingual_7)(rh.parahippocampal_3)(rh.precuneus_2)
0.00047	1.00E-05	0.90299	0.78846	(rh.inferiorparietal_9)(rh.insula_2)(rh.precuneus_2)(rh.superiortemporal_3)
0.00048	1.00E-05	0.89925	0.78365	(rh.fusiform_7)(rh.parahippocampal_3)(rh.precuneus_2)(rh.superiortemporal_3)
0.0005	1.00E-05	0.83582	0.70192	(rh.fusiform_7)(rh.inferiorparietal_9)(rh.superiortemporal_3)(rh.supramarginal_9)
0.0005	1.00E-05	0.83582	0.70192	(Right-Pallidum)(rh.fusiform_7)(rh.inferiorparietal_9)(rh.parahippocampal_3)
0.0005	1.00E-05	0.83582	0.70192	(rh.fusiform_7)(rh.inferiorparietal_10)(rh.inferiorparietal_9)(rh.precuneus_2)
0.0005	1.00E-05	0.83582	0.70192	(Right-Putamen)(rh.fusiform_7)(rh.inferiorparietal_9)(rh.parahippocampal_3)
0.0005	1.00E-05	0.83582	0.70192	(rh.fusiform_7)(rh.inferiorparietal_9)(rh.insula_2)(rh.parahippocampal_3)
0.0005	1.00E-05	0.83582	0.70192	(rh.fusiform_7)(rh.inferiorparietal_9)(rh.isthmuscingulate_2)(rh.parahippocampal_3)
0.0005	1.00E-05	0.83582	0.70192	(rh.fusiform_7)(rh.inferiorparietal_9)(rh.parahippocampal_3)
0.0005	1.00E-05	0.83582	0.70192	(Right-Thalamus-Proper)(rh.fusiform_7)(rh.inferiorparietal_9)(rh.parahippocampal_3)
0.0005	1.00E-05	0.83955	0.70673	(rh.bankssts_2)(rh.fusiform_7)(rh.inferiorparietal_9)(rh.supramarginal_9)
0.0005	1.00E-05	0.83955	0.70673	(rh.fusiform_7)(rh.inferiorparietal_9)(rh.lingual_7)(rh.supramarginal_9)
0.00051	1.00E-05	0.84328	0.71154	(rh.fusiform_7)(rh.inferiorparietal_9)(rh.parahippocampal_2)(rh.superiortemporal_3)
0.00052	1.00E-05	0.84701	0.71635	(rh.bankssts_2)(rh.fusiform_7)(rh.inferiorparietal_9)(rh.parahippocampal_2)
0.00052	1.00E-05	0.84701	0.71635	(rh.fusiform_7)(rh.inferiorparietal_9)(rh.lingual_7)(rh.parahippocampal_2)
0.00052	1.00E-05	0.85075	0.72115	(Right-Thalamus-Proper)(rh.fusiform_7)(rh.inferiorparietal_9)(rh.superiortemporal_3)
0.00052	1.00E-05	0.85075	0.72115	(rh.fusiform_7)(rh.inferiorparietal_9)(rh.isthmuscingulate_2)(rh.superiortemporal_3)
0.00052	1.00E-05	0.85075	0.72115	(Right-Putamen)(rh.fusiform_7)(rh.inferiorparietal_9)(rh.superiortemporal_3)
0.00052	1.00E-05	0.85075	0.72115	(rh.fusiform_7)(rh.inferiorparietal_9)(rh.superiortemporal_3)
0.00052	1.00E-05	0.85075	0.72115	(rh.fusiform_7)(rh.inferiorparietal_9)(rh.insula_2)(rh.superiortemporal_3)
0.00053	1.00E-05	0.85448	0.72596	(Right-Putamen)(rh.fusiform_7)(rh.inferiorparietal_9)(rh.lingual_7)
0.00053	1.00E-05	0.85448	0.72596	(rh.fusiform_7)(rh.inferiorparietal_9)(rh.insula_2)(rh.lingual_7)
0.00053	1.00E-05	0.85448	0.72596	(Right-Thalamus-Proper)(rh.fusiform_7)(rh.inferiorparietal_9)(rh.lingual_7)
0.00053	1.00E-05	0.85448	0.72596	(Right-Thalamus-Proper)(rh.bankssts_2)(rh.fusiform_7)(rh.inferiorparietal_9)
0.00053	1.00E-05	0.85448	0.72596	(rh.fusiform_7)(rh.inferiorparietal_9)(rh.isthmuscingulate_2)(rh.lingual_7)
0.00053	1.00E-05	0.85448	0.72596	(Right-Putamen)(rh.bankssts_2)(rh.fusiform_7)(rh.inferiorparietal_9)
0.00053	1.00E-05	0.85448	0.72596	(rh.bankssts_2)(rh.fusiform_7)(rh.inferiorparietal_9)(rh.isthmuscingulate_2)
0.00053	1.00E-05	0.85448	0.72596	(rh.fusiform_7)(rh.inferiorparietal_9)(rh.lingual_7)
0.00053	1.00E-05	0.85448	0.72596	(rh.bankssts_2)(rh.fusiform_7)(rh.inferiorparietal_9)(rh.insula_2)
0.00053	1.00E-05	0.85448	0.72596	(rh.bankssts_2)(rh.fusiform_7)(rh.inferiorparietal_9)
0.00061	1.00E-05	0.90672	0.7963	(rh.inferiorparietal_9)(rh.insula_2)(rh.lingual_7)(rh.precuneus_2)

0.00061	1.00E-05	0.90672	0.7963	(rh.inferiorparietal_9)(rh.precuneus_2)(rh.superiortemporal_3)
0.00061	1.00E-05	0.90672	0.7963	(rh.inferiorparietal_9)(rh.lingual_7)(rh.parahippocampal_2)
0.00061	1.00E-05	0.92164	0.81731	(rh.bankssts_2)(rh.fusiform_7)(rh.lingual_7)(rh.precuneus_2)
0.00064	1.00E-05	0.91791	0.8125	(rh.fusiform_7)(rh.lingual_7)(rh.precuneus_2)(rh.superiortemporal_3)
0.00064	1.00E-05	0.91791	0.8125	(rh.bankssts_2)(rh.fusiform_7)(rh.precuneus_2)(rh.superiortemporal_3)
0.00067	1.00E-05	0.81716	0.68269	(rh.entorhinal_1)(rh.fusiform_7)(rh.inferiorparietal_9)(rh.superiortemporal_9)
0.00067	1.00E-05	0.81716	0.68269	(Right-Pallidum)(rh.entorhinal_1)(rh.fusiform_7)(rh.inferiorparietal_9)
0.00067	1.00E-05	0.81716	0.68269	(Right-Putamen)(rh.entorhinal_1)(rh.fusiform_7)(rh.inferiorparietal_9)
0.00067	1.00E-05	0.81716	0.68269	(rh.entorhinal_1)(rh.fusiform_7)(rh.inferiorparietal_9)
0.00067	1.00E-05	0.81716	0.68269	(rh.entorhinal_1)(rh.fusiform_7)(rh.inferiorparietal_9)(rh.isthmuscingulate_2)
0.00067	1.00E-05	0.81716	0.68269	(rh.entorhinal_1)(rh.fusiform_7)(rh.inferiorparietal_9)(rh.insula_2)
0.00067	1.00E-05	0.81716	0.68269	(Right-Thalamus-Proper)(rh.entorhinal_1)(rh.fusiform_7)(rh.inferiorparietal_9)
0.00069	1.00E-05	0.8209	0.6875	(rh.fusiform_7)(rh.inferiorparietal_9)(rh.parahippocampal_3)(rh.supramarginal_9)
0.00071	1.00E-05	0.82463	0.69231	(Right-Caudate)(rh.fusiform_7)(rh.inferiorparietal_9)(rh.parahippocampal_3)
0.00072	1.00E-05	0.82836	0.69712	(rh.fusiform_7)(rh.inferiorparietal_9)(rh.insula_5)(rh.parahippocampal_3)
0.00072	1.00E-05	0.82836	0.69712	(rh.fusiform_7)(rh.inferiorparietal_9)(rh.parahippocampal_2)(rh.parahippocampal_3)
0.00072	1.00E-05	0.82836	0.69712	(rh.fusiform_7)(rh.inferiorparietal_9)(rh.precuneus_2)(rh.precuneus_3)
0.00074	1.00E-05	0.83209	0.70192	(rh.fusiform_7)(rh.inferiorparietal_9)(rh.parahippocampal_3)(rh.superiortemporal_9)
0.00074	1.00E-05	0.83209	0.70192	(rh.fusiform_7)(rh.inferiorparietal_9)(rh.parahippocampal_2)(rh.supramarginal_9)
0.00074	1.00E-05	0.83209	0.70192	(Right-Caudate)(rh.fusiform_7)(rh.inferiorparietal_9)(rh.supramarginal_9)
0.00074	1.00E-05	0.83209	0.70192	(rh.fusiform_7)(rh.inferiorparietal_9)(rh.insula_4)(rh.precuneus_2)
0.00074	1.00E-05	0.83209	0.70192	(rh.fusiform_7)(rh.inferiorparietal_4)(rh.inferiorparietal_9)(rh.precuneus_2)
0.00074	1.00E-05	0.90299	0.79327	(rh.fusiform_7)(rh.insula_2)(rh.parahippocampal_3)(rh.precuneus_2)
0.00074	1.00E-05	0.90299	0.79327	(Right-Thalamus-Proper)(rh.fusiform_7)(rh.parahippocampal_3)(rh.precuneus_2)
0.00074	1.00E-05	0.90299	0.79327	(rh.fusiform_7)(rh.parahippocampal_3)(rh.precuneus_2)
0.00074	1.00E-05	0.90299	0.79327	(rh.inferiorparietal_9)(rh.lingual_7)(rh.parahippocampal_2)(rh.superiortemporal_3)
0.00074	1.00E-05	0.90299	0.79327	(rh.fusiform_7)(rh.isthmuscingulate_2)(rh.parahippocampal_3)(rh.precuneus_2)
0.00074	1.00E-05	0.90299	0.79327	(Right-Pallidum)(rh.fusiform_7)(rh.parahippocampal_3)(rh.precuneus_2)
0.00074	1.00E-05	0.90299	0.79327	(Right-Putamen)(rh.fusiform_7)(rh.parahippocampal_3)(rh.precuneus_2)
0.00074	1.00E-05	0.90299	0.79327	(rh.bankssts_2)(rh.inferiorparietal_9)(rh.parahippocampal_2)(rh.superiortemporal_3)
0.00076	1.00E-05	0.89925	0.78846	(rh.inferiorparietal_9)(rh.insula_2)(rh.parahippocampal_2)(rh.precuneus_2)
0.00077	1.00E-05	0.83955	0.71154	(Right-Thalamus-Proper)(rh.fusiform_7)(rh.inferiorparietal_9)(rh.supramarginal_9)
0.00077	1.00E-05	0.83955	0.71154	(rh.fusiform_7)(rh.inferiorparietal_9)(rh.isthmuscingulate_2)(rh.supramarginal_9)
0.00077	1.00E-05	0.83955	0.71154	(rh.inferiorparietal_9)(rh.precuneus_2)(rh.precuneus_4)(rh.superiortemporal_3)
0.00077	1.00E-05	0.83955	0.71154	(Right-Caudate)(rh.fusiform_7)(rh.inferiorparietal_9)(rh.parahippocampal_2)

0.00077	1.00E-05	0.83955	0.71154	(Right-Putamen)(rh.fusiform_7)(rh.inferiorparietal_9)(rh.supramarginal_9)
0.00077	1.00E-05	0.83955	0.71154	(rh.fusiform_7)(rh.inferiorparietal_9)(rh.insula_2)(rh.supramarginal_9)
0.00077	1.00E-05	0.83955	0.71154	(Right-Caudate)(rh.fusiform_7)(rh.inferiorparietal_9)(rh.superiortemporal_3)
0.00078	1.00E-05	0.84328	0.71635	(Right-Caudate)(rh.bankssts_2)(rh.fusiform_7)(rh.inferiorparietal_9)
0.00078	1.00E-05	0.84328	0.71635	(Right-Caudate)(rh.fusiform_7)(rh.inferiorparietal_9)(rh.lingual_7)
0.00078	1.00E-05	0.89552	0.78365	(Right-Caudate)(rh.inferiorparietal_9)(rh.precuneus_2)(rh.superiortemporal_3)
0.00079	1.00E-05	0.84701	0.72115	(rh.fusiform_7)(rh.inferiorparietal_9)(rh.insula_2)(rh.parahippocampal_2)
0.00079	1.00E-05	0.84701	0.72115	(Right-Putamen)(rh.fusiform_7)(rh.inferiorparietal_9)(rh.parahippocampal_2)
0.00079	1.00E-05	0.84701	0.72115	(rh.fusiform_7)(rh.inferiorparietal_9)(rh.isthmuscingulate_2)(rh.parahippocampal_2)
0.00079	1.00E-05	0.84701	0.72115	(Right-Thalamus-Proper)(rh.fusiform_7)(rh.inferiorparietal_9)(rh.parahippocampal_2)
0.00079	1.00E-05	0.84701	0.72115	(rh.fusiform_7)(rh.inferiorparietal_9)(rh.parahippocampal_2)
0.00079	1.00E-05	0.84701	0.72115	(Right-Pallidum)(rh.fusiform_7)(rh.inferiorparietal_9)(rh.superiortemporal_3)
0.00079	1.00E-05	0.89179	0.77885	(rh.inferiorparietal_10)(rh.inferiorparietal_9)(rh.precuneus_2)(rh.superiortemporal_3)
0.00079	1.00E-05	0.89179	0.77885	(rh.inferiorparietal_9)(rh.precuneus_2)(rh.superiortemporal_3)(rh.supramarginal_9)
0.0008	1.00E-05	0.85075	0.72596	(Right-Pallidum)(rh.bankssts_2)(rh.fusiform_7)(rh.inferiorparietal_9)
0.0008	1.00E-05	0.85075	0.72596	(Right-Pallidum)(rh.fusiform_7)(rh.inferiorparietal_9)(rh.lingual_7)
0.00081	1.00E-05	0.85448	0.73077	(Right-Putamen)(rh.fusiform_7)(rh.inferiorparietal_9)(rh.insula_2)
0.00081	1.00E-05	0.85448	0.73077	(Right-Thalamus-Proper)(rh.fusiform_7)(rh.inferiorparietal_9)(rh.insula_2)
0.00081	1.00E-05	0.85448	0.73077	(Right-Putamen)(rh.fusiform_7)(rh.inferiorparietal_9)
0.00081	1.00E-05	0.85448	0.73077	(Right-Putamen)(rh.fusiform_7)(rh.inferiorparietal_9)(rh.isthmuscingulate_2)
0.00081	1.00E-05	0.85448	0.73077	(rh.fusiform_7)(rh.inferiorparietal_9)(rh.isthmuscingulate_2)
0.00081	1.00E-05	0.85448	0.73077	(Right-Putamen)(Right-Thalamus-Proper)(rh.fusiform_7)(rh.inferiorparietal_9)
0.00081	1.00E-05	0.85448	0.73077	(rh.fusiform_7)(rh.inferiorparietal_9)(rh.insula_2)(rh.isthmuscingulate_2)
0.00081	1.00E-05	0.85448	0.73077	(Right-Thalamus-Proper)(rh.fusiform_7)(rh.inferiorparietal_9)(rh.isthmuscingulate_2)
0.00081	1.00E-05	0.85448	0.73077	(rh.fusiform_7)(rh.precuneus_2)(rh.precuneus_4)(rh.superiortemporal_3)
0.00081	1.00E-05	0.85448	0.73077	(rh.fusiform_7)(rh.inferiorparietal_9)(rh.insula_2)
0.00081	1.00E-05	0.85448	0.73077	(Right-Thalamus-Proper)(rh.fusiform_7)(rh.inferiorparietal_9)
0.00081	1.00E-05	0.88433	0.76923	(rh.fusiform_7)(rh.fusiform_8)(rh.parahippocampal_3)(rh.precuneus_2)
0.00082	1.00E-05	0.85821	0.73558	(rh.bankssts_2)(rh.fusiform_7)(rh.precuneus_2)(rh.precuneus_4)
0.00095	1.00E-05	0.8097	0.67788	(rh.entorhinal_1)(rh.fusiform_7)(rh.inferiorparietal_9)(rh.parahippocampal_2)
0.00098	1.00E-05	0.81343	0.68269	(rh.entorhinal_1)(rh.fusiform_7)(rh.inferiorparietal_9)(rh.insula_5)
0.00099	1.00E-05	0.92164	0.82212	(Right-Putamen)(rh.bankssts_2)(rh.fusiform_7)(rh.precuneus_2)
0.00099	1.00E-05	0.92164	0.82212	(rh.fusiform_7)(rh.lingual_7)(rh.precuneus_2)
0.00099	1.00E-05	0.92164	0.82212	(rh.bankssts_2)(rh.fusiform_7)(rh.insula_2)(rh.precuneus_2)
0.00099	1.00E-05	0.92164	0.82212	(rh.bankssts_2)(rh.fusiform_7)(rh.isthmuscingulate_2)(rh.precuneus_2)

0.00099	1.00E-05	0.92164	0.82212	(Right-Putamen)(rh.fusiform_7)(rh.lingual_7)(rh.precuneus_2)
0.00099	1.00E-05	0.92164	0.82212	(Right-Thalamus-Proper)(rh.fusiform_7)(rh.lingual_7)(rh.precuneus_2)
0.00099	1.00E-05	0.92164	0.82212	(rh.bankssts_2)(rh.fusiform_7)(rh.precuneus_2)
0.00099	1.00E-05	0.92164	0.82212	(rh.fusiform_7)(rh.isthmuscingulate_2)(rh.lingual_7)(rh.precuneus_2)
0.00099	1.00E-05	0.92164	0.82212	(rh.fusiform_7)(rh.insula_2)(rh.lingual_7)(rh.precuneus_2)
0.00099	1.00E-05	0.92164	0.82212	(Right-Thalamus-Proper)(rh.bankssts_2)(rh.fusiform_7)(rh.precuneus_2)
0.00099	1.00E-05	0.90299	0.7963	(rh.inferiorparietal_9)(rh.parahippocampal_2)(rh.precuneus_2)
0.00099	1.00E-05	0.90299	0.7963	(rh.inferiorparietal_9)(rh.insula_2)(rh.parahippocampal_2)
0.00104	1.00E-05	0.91791	0.81731	(rh.fusiform_7)(rh.precuneus_2)(rh.superiortemporal_3)
0.00104	1.00E-05	0.91791	0.81731	(rh.fusiform_7)(rh.insula_2)(rh.precuneus_2)(rh.superiortemporal_3)
0.00104	1.00E-05	0.91791	0.81731	(rh.fusiform_7)(rh.isthmuscingulate_2)(rh.precuneus_2)(rh.superiortemporal_3)
0.00104	1.00E-05	0.91791	0.81731	(Right-Thalamus-Proper)(rh.fusiform_7)(rh.precuneus_2)(rh.superiortemporal_3)
0.00104	1.00E-05	0.91791	0.81731	(Right-Putamen)(rh.fusiform_7)(rh.precuneus_2)(rh.superiortemporal_3)
0.00107	1.00E-05	0.98881	0.93269	(rh.bankssts_2)(rh.lingual_7)(rh.precuneus_2)(rh.superiortemporal_3)
0.00107	1.00E-05	0.89552	0.78704	(rh.inferiorparietal_9)(rh.superiortemporal_3)(rh.supramarginal_9)
0.00107	1.00E-05	0.89552	0.78704	(rh.inferiorparietal_10)(rh.inferiorparietal_9)(rh.superiortemporal_3)
0.00112	1.00E-05	0.91045	0.80769	(rh.fusiform_7)(rh.lingual_7)(rh.parahippocampal_2)(rh.precuneus_2)
0.00112	1.00E-05	0.91045	0.80769	(rh.bankssts_2)(rh.inferiorparietal_9)(rh.lingual_7)(rh.superiortemporal_3)
0.00112	1.00E-05	0.91045	0.80769	(rh.bankssts_2)(rh.fusiform_7)(rh.parahippocampal_2)(rh.precuneus_2)
0.00113	1.00E-05	0.83582	0.71154	(Right-Pallidum)(rh.fusiform_7)(rh.inferiorparietal_9)(rh.supramarginal_9)
0.00115	1.00E-05	0.90672	0.80288	(rh.fusiform_7)(rh.parahippocampal_2)(rh.precuneus_2)(rh.superiortemporal_3)
0.00115	1.00E-05	0.90672	0.80288	(rh.bankssts_2)(rh.inferiorparietal_9)(rh.insula_2)(rh.precuneus_2)
0.00116	1.00E-05	0.83955	0.71635	(rh.fusiform_7)(rh.inferiorparietal_9)(rh.insula_5)(rh.superiortemporal_3)
0.00118	1.00E-05	0.84328	0.72115	(Right-Caudate)(rh.fusiform_7)(rh.inferiorparietal_9)
0.00118	1.00E-05	0.84328	0.72115	(Right-Pallidum)(rh.fusiform_7)(rh.inferiorparietal_9)(rh.parahippocampal_2)
0.00118	1.00E-05	0.84328	0.72115	(Right-Caudate)(Right-Putamen)(rh.fusiform_7)(rh.inferiorparietal_9)
0.00118	1.00E-05	0.84328	0.72115	(Right-Caudate)(Right-Thalamus-Proper)(rh.fusiform_7)(rh.inferiorparietal_9)
0.00118	1.00E-05	0.84328	0.72115	(rh.fusiform_7)(rh.inferiorparietal_9)(rh.superiortemporal_3)(rh.superiortemporal_9)
0.00118	1.00E-05	0.84328	0.72115	(Right-Caudate)(rh.fusiform_7)(rh.inferiorparietal_9)(rh.isthmuscingulate_2)
0.00118	1.00E-05	0.84328	0.72115	(rh.fusiform_7)(rh.inferiorparietal_9)(rh.insula_5)(rh.lingual_7)
0.00118	1.00E-05	0.84328	0.72115	(Right-Caudate)(rh.fusiform_7)(rh.inferiorparietal_9)(rh.insula_2)
0.00118	1.00E-05	0.84328	0.72115	(rh.bankssts_2)(rh.fusiform_7)(rh.inferiorparietal_9)(rh.insula_5)
0.00118	1.00E-05	0.90299	0.79808	(rh.inferiorparietal_9)(rh.isthmuscingulate_2)(rh.parahippocampal_2)(rh.superiortempora
0.00118	1.00E-05	0.90299	0.79808	(Right-Putamen)(rh.inferiorparietal_9)(rh.parahippocampal_2)(rh.superiortemporal_3)
0.00118	1.00E-05	0.90299	0.79808	(rh.bankssts_2)(rh.inferiorparietal_9)(rh.parahippocampal_2)(rh.precuneus_2)

0.00118	1.00E-05	0.90299	0.79808	(Right-Thalamus-Proper)(rh.inferiorparietal_9)(rh.parahippocampal_2)(rh.superiortempor
0.00118	1.00E-05	0.90299	0.79808	(rh.inferiorparietal_9)(rh.lingual_7)(rh.parahippocampal_2)(rh.precuneus_2)
0.0012	1.00E-05	0.84701	0.72596	(rh.bankssts_2)(rh.fusiform_7)(rh.inferiorparietal_9)(rh.superiortemporal_9)
0.0012	1.00E-05	0.84701	0.72596	(rh.fusiform_7)(rh.inferiorparietal_9)(rh.lingual_7)(rh.superiortemporal_9)
0.0012	1.00E-05	0.89925	0.79327	(rh.inferiorparietal_9)(rh.insula_2)(rh.parahippocampal_2)(rh.superiortemporal_3)
0.00121	1.00E-05	0.85075	0.73077	(Right-Pallidum)(rh.fusiform_7)(rh.inferiorparietal_9)(rh.insula_2)
0.00121	1.00E-05	0.85075	0.73077	(Right-Pallidum)(Right-Putamen)(rh.fusiform_7)(rh.inferiorparietal_9)
0.00121	1.00E-05	0.85075	0.73077	(Right-Pallidum)(Right-Thalamus-Proper)(rh.fusiform_7)(rh.inferiorparietal_9)
0.00121	1.00E-05	0.85075	0.73077	(Right-Pallidum)(rh.fusiform_7)(rh.inferiorparietal_9)(rh.isthmuscingulate_2)
0.00121	1.00E-05	0.85075	0.73077	(Right-Pallidum)(rh.fusiform_7)(rh.inferiorparietal_9)
0.00122	1.00E-05	0.89552	0.78846	(Right-Caudate)(rh.inferiorparietal_9)(rh.parahippocampal_2)(rh.superiortemporal_3)
0.00122	1.00E-05	0.89552	0.78846	(rh.fusiform_7)(rh.insula_5)(rh.parahippocampal_3)(rh.precuneus_2)
0.00123	1.00E-05	0.85448	0.73558	(rh.inferiorparietal_9)(rh.parahippocampal_3)(rh.precuneus_2)(rh.superiortemporal_3)
0.00124	1.00E-05	0.89179	0.78365	(Right-Caudate)(rh.fusiform_7)(rh.parahippocampal_3)(rh.precuneus_2)
0.00124	1.00E-05	0.85821	0.74038	(rh.bankssts_2)(rh.inferiorparietal_9)(rh.parahippocampal_3)(rh.precuneus_2)
0.00124	1.00E-05	0.85821	0.74038	(rh.fusiform_7)(rh.precuneus_2)(rh.precuneus_4)
0.00124	1.00E-05	0.85821	0.74038	(rh.fusiform_7)(rh.insula_2)(rh.precuneus_2)(rh.precuneus_4)
0.00124	1.00E-05	0.85821	0.74038	(Right-Putamen)(rh.fusiform_7)(rh.precuneus_2)(rh.precuneus_4)
0.00124	1.00E-05	0.85821	0.74038	(Right-Thalamus-Proper)(rh.fusiform_7)(rh.precuneus_2)(rh.precuneus_4)
0.00124	1.00E-05	0.85821	0.74038	(rh.inferiorparietal_9)(rh.lingual_7)(rh.parahippocampal_3)(rh.precuneus_2)
0.00124	1.00E-05	0.85821	0.74038	(rh.fusiform_7)(rh.isthmuscingulate_2)(rh.precuneus_2)(rh.precuneus_4)
0.00125	1.00E-05	0.88806	0.77885	(rh.inferiorparietal_9)(rh.parahippocampal_2)(rh.superiortemporal_3)(rh.supramarginal_[illegible]
0.00126	1.00E-05	0.86567	0.75	(rh.inferiorparietal_9)(rh.insula_5)(rh.parahippocampal_2)(rh.precuneus_2)
0.00127	1.00E-05	0.87313	0.75962	(rh.entorhinal_1)(rh.fusiform_7)(rh.parahippocampal_3)(rh.precuneus_2)
0.00137	1.00E-05	0.80597	0.67788	(Right-Caudate)(rh.entorhinal_1)(rh.fusiform_7)(rh.inferiorparietal_9)
0.00137	1.00E-05	0.80597	0.67788	(rh.entorhinal_1)(rh.fusiform_7)(rh.inferiorparietal_9)(rh.insula_4)
0.00145	1.00E-05	0.81343	0.6875	(rh.bankssts_2)(rh.fusiform_7)(rh.isthmuscingulate_1)(rh.precuneus_4)
0.00151	1.00E-05	0.97761	0.91346	(rh.bankssts_2)(rh.parahippocampal_2)(rh.precuneus_2)(rh.superiortemporal_3)
0.00151	1.00E-05	0.97761	0.91346	(rh.lingual_7)(rh.parahippocampal_2)(rh.precuneus_2)(rh.superiortemporal_3)
0.00154	1.00E-05	0.8209	0.69712	(rh.fusiform_7)(rh.inferiorparietal_9)(rh.precuneus_2)(rh.superiortemporal_1)
0.00154	1.00E-05	0.8209	0.69712	(rh.fusiform_7)(rh.inferiorparietal_10)(rh.inferiorparietal_9)(rh.parahippocampal_3)
0.00154	1.00E-05	0.8209	0.69712	(rh.fusiform_7)(rh.fusiform_8)(rh.inferiorparietal_9)(rh.superiortemporal_3)
0.00154	1.00E-05	0.8209	0.69712	(rh.fusiform_7)(rh.inferiorparietal_9)(rh.insula_4)(rh.parahippocampal_3)
0.00154	1.00E-05	0.8209	0.69712	(rh.fusiform_7)(rh.inferiorparietal_4)(rh.inferiorparietal_9)(rh.parahippocampal_3)
0.00158	1.00E-05	0.82463	0.70192	(rh.fusiform_7)(rh.fusiform_8)(rh.inferiorparietal_9)(rh.lingual_7)

0.00158	1.00E-05	0.82463	0.70192	(rh.entorhinal_1)(rh.inferiorparietal_9)(rh.parahippocampal_3)(rh.precuneus_2)
0.00158	1.00E-05	0.82463	0.70192	(rh.bankssts_2)(rh.fusiform_7)(rh.fusiform_8)(rh.inferiorparietal_9)
0.00159	1.00E-05	0.89925	0.7963	(rh.inferiorparietal_9)(rh.lingual_7)(rh.supramarginal_9)
0.00159	1.00E-05	0.89925	0.7963	(Right-Caudate)(rh.inferiorparietal_9)(rh.insula_2)(rh.lingual_7)
0.00159	1.00E-05	0.89925	0.7963	(Right-Caudate)(rh.bankssts_2)(rh.inferiorparietal_9)(rh.superiortemporal_3)
0.00159	1.00E-05	0.89925	0.7963	(Right-Caudate)(rh.inferiorparietal_9)(rh.superiortemporal_3)
0.00159	1.00E-05	0.89925	0.7963	(Right-Caudate)(rh.inferiorparietal_9)(rh.lingual_7)(rh.precuneus_2)
0.00159	1.00E-05	0.89925	0.7963	(Right-Pallidum)(rh.inferiorparietal_9)(rh.lingual_7)(rh.precuneus_2)
0.00159	1.00E-05	0.89925	0.7963	(Right-Caudate)(rh.inferiorparietal_9)(rh.parahippocampal_2)
0.00159	1.00E-05	0.89925	0.7963	(rh.inferiorparietal_10)(rh.inferiorparietal_9)(rh.lingual_7)
0.00159	1.00E-05	0.92164	0.82692	(Right-Thalamus-Proper)(rh.fusiform_7)(rh.insula_2)(rh.precuneus_2)
0.00159	1.00E-05	0.92164	0.82692	(Right-Thalamus-Proper)(rh.fusiform_7)(rh.precuneus_2)
0.00159	1.00E-05	0.92164	0.82692	(Right-Thalamus-Proper)(rh.fusiform_7)(rh.isthmuscingulate_2)(rh.precuneus_2)
0.00159	1.00E-05	0.92164	0.82692	(Right-Putamen)(Right-Thalamus-Proper)(rh.fusiform_7)(rh.precuneus_2)
0.00159	1.00E-05	0.92164	0.82692	(Right-Putamen)(rh.fusiform_7)(rh.isthmuscingulate_2)(rh.precuneus_2)
0.00159	1.00E-05	0.92164	0.82692	(rh.fusiform_7)(rh.insula_2)(rh.precuneus_2)
0.00159	1.00E-05	0.92164	0.82692	(Right-Putamen)(rh.fusiform_7)(rh.insula_2)(rh.precuneus_2)
0.00159	1.00E-05	0.92164	0.82692	(rh.fusiform_7)(rh.insula_2)(rh.isthmuscingulate_2)(rh.precuneus_2)
0.00159	1.00E-05	0.92164	0.82692	(rh.fusiform_7)(rh.isthmuscingulate_2)(rh.precuneus_2)
0.00159	1.00E-05	0.92164	0.82692	(Right-Putamen)(rh.fusiform_7)(rh.precuneus_2)
0.00159	1.00E-05	0.92164	0.82692	(rh.fusiform_7)(rh.precuneus_2)
0.00162	1.00E-05	0.82836	0.70673	(rh.fusiform_7)(rh.inferiorparietal_9)(rh.insula_5)(rh.supramarginal_9)
0.00162	1.00E-05	0.82836	0.70673	(rh.entorhinal_1)(rh.inferiorparietal_9)(rh.precuneus_2)(rh.superiortemporal_3)
0.00162	1.00E-05	0.82836	0.70673	(rh.fusiform_7)(rh.inferiorparietal_10)(rh.inferiorparietal_9)(rh.supramarginal_9)
0.00165	1.00E-05	0.83209	0.71154	(rh.fusiform_8)(rh.inferiorparietal_9)(rh.parahippocampal_3)(rh.precuneus_2)
0.00165	1.00E-05	0.83209	0.71154	(rh.bankssts_2)(rh.entorhinal_1)(rh.inferiorparietal_9)(rh.precuneus_2)
0.00165	1.00E-05	0.83209	0.71154	(rh.entorhinal_1)(rh.inferiorparietal_9)(rh.lingual_7)(rh.precuneus_2)
0.00165	1.00E-05	0.83209	0.71154	(rh.fusiform_7)(rh.inferiorparietal_9)(rh.superiortemporal_9)(rh.supramarginal_9)
0.00166	1.00E-05	0.91791	0.82212	(Right-Pallidum)(rh.fusiform_7)(rh.lingual_7)(rh.precuneus_2)
0.00166	1.00E-05	0.91791	0.82212	(Right-Pallidum)(rh.bankssts_2)(rh.fusiform_7)(rh.precuneus_2)
0.00168	1.00E-05	0.89179	0.78704	(rh.inferiorparietal_9)(rh.parahippocampal_2)(rh.supramarginal_9)
0.00168	1.00E-05	0.89179	0.78704	(rh.inferiorparietal_10)(rh.inferiorparietal_9)(rh.parahippocampal_2)
0.00169	1.00E-05	0.83582	0.71635	(rh.fusiform_7)(rh.inferiorparietal_9)(rh.insula_5)(rh.parahippocampal_2)
0.00169	1.00E-05	0.83582	0.71635	(rh.fusiform_7)(rh.inferiorparietal_10)(rh.inferiorparietal_9)(rh.superiortemporal_3)
0.00169	1.00E-05	0.83582	0.71635	(rh.inferiorparietal_9)(rh.parahippocampal_2)(rh.precuneus_4)(rh.superiortemporal_3)

0.00171	1.00E-05	0.91418	0.81731	(Right-Pallidum)(rh.fusiform_7)(rh.precuneus_2)(rh.superiortemporal_3)
0.00173	1.00E-05	0.83955	0.72115	(rh.fusiform_7)(rh.inferiorparietal_9)(rh.parahippocampal_2)(rh.superiortemporal_9)
0.00173	1.00E-05	0.83955	0.72115	(rh.bankssts_2)(rh.fusiform_7)(rh.inferiorparietal_10)(rh.inferiorparietal_9)
0.00173	1.00E-05	0.83955	0.72115	(Right-Caudate)(Right-Pallidum)(rh.fusiform_7)(rh.inferiorparietal_9)
0.00173	1.00E-05	0.83955	0.72115	(rh.fusiform_7)(rh.inferiorparietal_10)(rh.inferiorparietal_9)(rh.lingual_7)
0.00173	1.00E-05	0.83955	0.72115	(rh.fusiform_7)(rh.parahippocampal_3)(rh.precuneus_4)(rh.superiortemporal_3)
0.00176	1.00E-05	0.84328	0.72596	(rh.fusiform_7)(rh.inferiorparietal_9)(rh.insula_5)
0.00176	1.00E-05	0.84328	0.72596	(rh.fusiform_7)(rh.inferiorparietal_9)(rh.insula_5)(rh.isthmuscingulate_2)
0.00176	1.00E-05	0.84328	0.72596	(Right-Pallidum)(rh.fusiform_7)(rh.inferiorparietal_9)(rh.insula_5)
0.00176	1.00E-05	0.84328	0.72596	(rh.fusiform_7)(rh.inferiorparietal_9)(rh.insula_2)(rh.insula_5)
0.00176	1.00E-05	0.84328	0.72596	(Right-Putamen)(rh.fusiform_7)(rh.inferiorparietal_9)(rh.insula_5)
0.00176	1.00E-05	0.84328	0.72596	(Right-Thalamus-Proper)(rh.fusiform_7)(rh.inferiorparietal_9)(rh.insula_5)
0.00176	1.00E-05	0.84328	0.72596	(rh.bankssts_2)(rh.fusiform_7)(rh.parahippocampal_3)(rh.precuneus_4)
0.00176	1.00E-05	0.91045	0.8125	(rh.fusiform_7)(rh.parahippocampal_2)(rh.precuneus_2)
0.00176	1.00E-05	0.91045	0.8125	(Right-Caudate)(rh.fusiform_7)(rh.lingual_7)(rh.precuneus_2)
0.00176	1.00E-05	0.91045	0.8125	(Right-Caudate)(rh.bankssts_2)(rh.fusiform_7)(rh.precuneus_2)
0.00176	1.00E-05	0.91045	0.8125	(Right-Thalamus-Proper)(rh.inferiorparietal_9)(rh.lingual_7)(rh.superiortemporal_3)
0.00176	1.00E-05	0.91045	0.8125	(rh.fusiform_7)(rh.isthmuscingulate_2)(rh.parahippocampal_2)(rh.precuneus_2)
0.00176	1.00E-05	0.91045	0.8125	(rh.bankssts_2)(rh.inferiorparietal_9)(rh.superiortemporal_3)
0.00176	1.00E-05	0.91045	0.8125	(Right-Putamen)(rh.bankssts_2)(rh.inferiorparietal_9)(rh.superiortemporal_3)
0.00176	1.00E-05	0.91045	0.8125	(Right-Thalamus-Proper)(rh.fusiform_7)(rh.parahippocampal_2)(rh.precuneus_2)
0.00176	1.00E-05	0.91045	0.8125	(rh.fusiform_7)(rh.insula_2)(rh.parahippocampal_2)(rh.precuneus_2)
0.00176	1.00E-05	0.91045	0.8125	(Right-Putamen)(rh.fusiform_7)(rh.parahippocampal_2)(rh.precuneus_2)
0.00176	1.00E-05	0.91045	0.8125	(Right-Putamen)(rh.inferiorparietal_9)(rh.lingual_7)(rh.superiortemporal_3)
0.00176	1.00E-05	0.91045	0.8125	(rh.bankssts_2)(rh.inferiorparietal_9)(rh.isthmuscingulate_2)(rh.superiortemporal_3)
0.00176	1.00E-05	0.91045	0.8125	(rh.inferiorparietal_9)(rh.isthmuscingulate_2)(rh.lingual_7)(rh.superiortemporal_3)
0.00176	1.00E-05	0.91045	0.8125	(Right-Thalamus-Proper)(rh.bankssts_2)(rh.inferiorparietal_9)(rh.superiortemporal_3)
0.00176	1.00E-05	0.91045	0.8125	(rh.bankssts_2)(rh.inferiorparietal_9)(rh.lingual_7)(rh.precuneus_2)
0.00179	1.00E-05	0.84701	0.73077	(rh.fusiform_7)(rh.inferiorparietal_9)(rh.isthmuscingulate_2)(rh.superiortemporal_9)
0.00179	1.00E-05	0.84701	0.73077	(Right-Pallidum)(rh.fusiform_7)(rh.inferiorparietal_9)(rh.superiortemporal_9)
0.00179	1.00E-05	0.84701	0.73077	(Right-Thalamus-Proper)(rh.fusiform_7)(rh.inferiorparietal_9)(rh.superiortemporal_9)
0.00179	1.00E-05	0.84701	0.73077	(Right-Accumbens-area)(rh.fusiform_7)(rh.precuneus_2)(rh.superiortemporal_3)
0.00179	1.00E-05	0.84701	0.73077	(Right-Putamen)(rh.fusiform_7)(rh.inferiorparietal_9)(rh.superiortemporal_9)
0.00179	1.00E-05	0.84701	0.73077	(rh.fusiform_7)(rh.inferiorparietal_9)(rh.insula_2)(rh.superiortemporal_9)
0.00179	1.00E-05	0.84701	0.73077	(rh.fusiform_7)(rh.inferiorparietal_9)(rh.superiortemporal_9)

0.0018	1.00E-05	0.90672	0.80769	(rh.bankssts_2)(rh.fusiform_7)(rh.lingual_7)(rh.parahippocampal_3)
0.0018	1.00E-05	0.90672	0.80769	(Right-Putamen)(rh.inferiorparietal_9)(rh.insula_2)(rh.precuneus_2)
0.0018	1.00E-05	0.90672	0.80769	(Right-Thalamus-Proper)(rh.inferiorparietal_9)(rh.insula_2)(rh.precuneus_2)
0.0018	1.00E-05	0.90672	0.80769	(rh.bankssts_2)(rh.inferiorparietal_9)(rh.insula_2)(rh.superiortemporal_3)
0.0018	1.00E-05	0.90672	0.80769	(rh.inferiorparietal_9)(rh.insula_2)(rh.isthmuscingulate_2)(rh.precuneus_2)
0.0018	1.00E-05	0.90672	0.80769	(rh.fusiform_7)(rh.lingual_7)(rh.precuneus_2)(rh.supramarginal_9)
0.0018	1.00E-05	0.90672	0.80769	(rh.inferiorparietal_9)(rh.insula_2)(rh.precuneus_2)
0.0018	1.00E-05	0.90672	0.80769	(Right-Caudate)(rh.fusiform_7)(rh.precuneus_2)(rh.superiortemporal_3)
0.0018	1.00E-05	0.90672	0.80769	(rh.inferiorparietal_9)(rh.insula_2)(rh.lingual_7)(rh.superiortemporal_3)
0.0018	1.00E-05	0.90672	0.80769	(rh.bankssts_2)(rh.fusiform_7)(rh.precuneus_2)(rh.supramarginal_9)
0.00182	1.00E-05	0.85075	0.73558	(rh.inferiorparietal_9)(rh.insula_5)(rh.parahippocampal_3)(rh.precuneus_2)
0.00182	1.00E-05	0.85075	0.73558	(rh.fusiform_7)(rh.inferiortemporal_2)(rh.parahippocampal_3)(rh.precuneus_2)
0.00182	1.00E-05	0.85075	0.73558	(rh.inferiorparietal_9)(rh.parahippocampal_2)(rh.parahippocampal_3)(rh.precuneus_2)
0.00182	1.00E-05	0.85075	0.73558	(Right-Accumbens-area)(rh.bankssts_2)(rh.fusiform_7)(rh.precuneus_2)
0.00184	1.00E-05	0.90299	0.80288	(rh.inferiorparietal_9)(rh.isthmuscingulate_2)(rh.parahippocampal_2)(rh.precuneus_2)
0.00184	1.00E-05	0.90299	0.80288	(Right-Caudate)(rh.fusiform_7)(rh.parahippocampal_2)(rh.precuneus_2)
0.00184	1.00E-05	0.90299	0.80288	(rh.bankssts_2)(rh.fusiform_7)(rh.parahippocampal_3)(rh.superiortemporal_3)
0.00184	1.00E-05	0.90299	0.80288	(Right-Putamen)(rh.inferiorparietal_9)(rh.parahippocampal_2)(rh.precuneus_2)
0.00184	1.00E-05	0.90299	0.80288	(Right-Thalamus-Proper)(rh.inferiorparietal_9)(rh.parahippocampal_2)(rh.precuneus_2)
0.00184	1.00E-05	0.90299	0.80288	(rh.fusiform_7)(rh.lingual_7)(rh.parahippocampal_3)(rh.superiortemporal_3)
0.00184	1.00E-05	0.90299	0.80288	(rh.fusiform_7)(rh.precuneus_2)(rh.superiortemporal_3)(rh.supramarginal_9)
0.00185	1.00E-05	0.85448	0.74038	(Right-Pallidum)(rh.fusiform_7)(rh.precuneus_2)(rh.precuneus_4)
0.00186	1.00E-05	0.83209	0.71296	(rh.inferiorparietal_9)(rh.isthmuscingulate_1)(rh.supramarginal_9)
0.00187	1.00E-05	0.85821	0.74519	(rh.inferiorparietal_9)(rh.parahippocampal_3)(rh.precuneus_2)
0.00187	1.00E-05	0.85821	0.74519	(Right-Pallidum)(rh.inferiorparietal_9)(rh.parahippocampal_3)(rh.precuneus_2)
0.00187	1.00E-05	0.85821	0.74519	(Right-Putamen)(rh.inferiorparietal_9)(rh.parahippocampal_3)(rh.precuneus_2)
0.00187	1.00E-05	0.85821	0.74519	(rh.inferiorparietal_9)(rh.insula_2)(rh.parahippocampal_3)(rh.precuneus_2)
0.00187	1.00E-05	0.85821	0.74519	(rh.inferiorparietal_9)(rh.isthmuscingulate_2)(rh.parahippocampal_3)(rh.precuneus_2)
0.00187	1.00E-05	0.85821	0.74519	(rh.fusiform_7)(rh.parahippocampal_3)(rh.precuneus_2)(rh.superiortemporal_6)
0.00187	1.00E-05	0.85821	0.74519	(Right-Thalamus-Proper)(rh.inferiorparietal_9)(rh.parahippocampal_3)(rh.precuneus_2)
0.00188	1.00E-05	0.83955	0.72222	(rh.fusiform_7)(rh.inferiorparietal_9)(rh.supramarginal_9)
0.0019	1.00E-05	0.89552	0.79327	(Right-Pallidum)(rh.inferiorparietal_9)(rh.precuneus_2)(rh.superiortemporal_3)
0.0019	1.00E-05	0.89552	0.79327	(Right-Caudate)(rh.inferiorparietal_9)(rh.parahippocampal_2)(rh.precuneus_2)
0.0019	1.00E-05	0.89552	0.79327	(rh.fusiform_7)(rh.parahippocampal_3)(rh.precuneus_2)(rh.superiortemporal_9)
0.0019	1.00E-05	0.89552	0.79327	(rh.inferiorparietal_9)(rh.lingual_7)(rh.superiortemporal_3)(rh.supramarginal_9)

0.0019	1.00E-05	0.89552	0.79327	(rh.bankssts_2)(rh.inferiorparietal_9)(rh.superiortemporal_3)(rh.supramarginal_9)
0.0019	1.00E-05	0.85448	0.74074	(rh.fusiform_7)(rh.inferiorparietal_9)
0.00191	1.00E-05	0.89179	0.78846	(rh.fusiform_7)(rh.parahippocampal_2)(rh.parahippocampal_3)(rh.precuneus_2)
0.00192	1.00E-05	0.8694	0.75962	(rh.inferiorparietal_9)(rh.insula_5)(rh.precuneus_2)(rh.superiortemporal_3)
0.00193	1.00E-05	0.88806	0.78365	(rh.fusiform_7)(rh.inferiorparietal_4)(rh.parahippocampal_3)(rh.precuneus_2)
0.00193	1.00E-05	0.88806	0.78365	(rh.fusiform_7)(rh.parahippocampal_3)(rh.precuneus_2)(rh.supramarginal_9)
0.00193	1.00E-05	0.87313	0.76442	(rh.bankssts_2)(rh.inferiorparietal_9)(rh.insula_5)(rh.precuneus_2)
0.00193	1.00E-05	0.87313	0.76442	(rh.inferiorparietal_9)(rh.insula_5)(rh.lingual_7)(rh.precuneus_2)
0.00194	1.00E-05	0.87687	0.76923	(rh.entorhinal_1)(rh.fusiform_7)(rh.precuneus_2)(rh.superiortemporal_3)
0.00194	1.00E-05	0.8806	0.77404	(rh.entorhinal_1)(rh.fusiform_7)(rh.lingual_7)(rh.precuneus_2)
0.00194	1.00E-05	0.8806	0.77404	(rh.bankssts_2)(rh.entorhinal_1)(rh.fusiform_7)(rh.precuneus_2)
0.002	1.00E-05	0.80597	0.68269	(Right-Caudate)(rh.fusiform_7)(rh.isthmuscingulate_1)(rh.precuneus_4)
0.00207	1.00E-05	0.98881	0.9375	(Right-Thalamus-Proper)(rh.lingual_7)(rh.precuneus_2)(rh.superiortemporal_3)
0.00207	1.00E-05	0.98881	0.9375	(Right-Putamen)(rh.lingual_7)(rh.precuneus_2)(rh.superiortemporal_3)
0.00207	1.00E-05	0.98881	0.9375	(rh.lingual_7)(rh.precuneus_2)(rh.superiortemporal_3)
0.00207	1.00E-05	0.98881	0.9375	(rh.bankssts_2)(rh.precuneus_2)(rh.superiortemporal_3)
0.00207	1.00E-05	0.98881	0.9375	(rh.bankssts_2)(rh.isthmuscingulate_2)(rh.precuneus_2)(rh.superiortemporal_3)
0.00207	1.00E-05	0.98881	0.9375	(Right-Putamen)(rh.bankssts_2)(rh.precuneus_2)(rh.superiortemporal_3)
0.00207	1.00E-05	0.98881	0.9375	(rh.isthmuscingulate_2)(rh.lingual_7)(rh.precuneus_2)(rh.superiortemporal_3)
0.00207	1.00E-05	0.98881	0.9375	(Right-Thalamus-Proper)(rh.bankssts_2)(rh.precuneus_2)(rh.superiortemporal_3)
0.00207	1.00E-05	0.8097	0.6875	(rh.fusiform_7)(rh.inferiorparietal_9)(rh.parahippocampal_3)(rh.precuneus_3)
0.00207	1.00E-05	0.8097	0.6875	(rh.fusiform_7)(rh.fusiform_8)(rh.inferiorparietal_9)(rh.supramarginal_9)
0.00207	1.00E-05	0.8097	0.6875	(rh.inferiorparietal_9)(rh.insula_5)(rh.precuneus_2)(rh.precuneus_4)
0.00213	1.00E-05	0.81343	0.69231	(rh.fusiform_7)(rh.insula_2)(rh.isthmuscingulate_1)(rh.precuneus_4)
0.00213	1.00E-05	0.81343	0.69231	(Right-Putamen)(rh.fusiform_7)(rh.isthmuscingulate_1)(rh.precuneus_4)
0.00213	1.00E-05	0.81343	0.69231	(Right-Thalamus-Proper)(rh.fusiform_7)(rh.isthmuscingulate_1)(rh.precuneus_4)
0.00213	1.00E-05	0.81343	0.69231	(rh.fusiform_7)(rh.isthmuscingulate_1)(rh.isthmuscingulate_2)(rh.precuneus_4)
0.00219	1.00E-05	0.81716	0.69712	(rh.fusiform_7)(rh.fusiform_8)(rh.inferiorparietal_9)(rh.parahippocampal_2)
0.00226	1.00E-05	0.8209	0.70192	(rh.fusiform_7)(rh.inferiorparietal_9)(rh.superiortemporal_1)(rh.supramarginal_9)
0.00229	1.00E-05	0.97015	0.90385	(Right-Caudate)(rh.parahippocampal_2)(rh.precuneus_2)(rh.superiortemporal_3)
0.00232	1.00E-05	0.82463	0.70673	(rh.fusiform_7)(rh.fusiform_8)(rh.inferiorparietal_9)(rh.superiortemporal_9)
0.00232	1.00E-05	0.82463	0.70673	(rh.fusiform_7)(rh.fusiform_8)(rh.inferiorparietal_9)
0.00232	1.00E-05	0.82463	0.70673	(Right-Thalamus-Proper)(rh.fusiform_7)(rh.fusiform_8)(rh.inferiorparietal_9)
0.00232	1.00E-05	0.82463	0.70673	(Right-Pallidum)(rh.fusiform_7)(rh.fusiform_8)(rh.inferiorparietal_9)
0.00232	1.00E-05	0.82463	0.70673	(rh.entorhinal_1)(rh.inferiorparietal_9)(rh.parahippocampal_2)(rh.precuneus_2)

0.00232	1.00E-05	0.82463	0.70673	(Right-Putamen)(rh.fusiform_7)(rh.fusiform_8)(rh.inferiorparietal_9)
0.00232	1.00E-05	0.82463	0.70673	(rh.fusiform_7)(rh.fusiform_8)(rh.parahippocampal_3)(rh.precuneus_4)
0.00232	1.00E-05	0.82463	0.70673	(rh.fusiform_7)(rh.fusiform_8)(rh.inferiorparietal_9)(rh.insula_2)
0.00232	1.00E-05	0.82463	0.70673	(rh.fusiform_7)(rh.fusiform_8)(rh.inferiorparietal_9)(rh.isthmuscingulate_2)
0.00232	1.00E-05	0.82463	0.70673	(rh.fusiform_7)(rh.inferiorparietal_9)(rh.precuneus_3)(rh.superiortemporal_3)
0.00238	1.00E-05	0.82836	0.71154	(rh.fusiform_7)(rh.inferiorparietal_9)(rh.lingual_7)(rh.precuneus_3)
0.00238	1.00E-05	0.82836	0.71154	(rh.bankssts_2)(rh.fusiform_7)(rh.inferiorparietal_9)(rh.precuneus_3)
0.00244	1.00E-05	0.83209	0.71635	(Right-Putamen)(rh.entorhinal_1)(rh.inferiorparietal_9)(rh.precuneus_2)
0.00244	1.00E-05	0.83209	0.71635	(rh.entorhinal_1)(rh.inferiorparietal_9)(rh.precuneus_2)(rh.superiortemporal_9)
0.00244	1.00E-05	0.83209	0.71635	(rh.entorhinal_1)(rh.inferiorparietal_9)(rh.precuneus_2)
0.00244	1.00E-05	0.83209	0.71635	(Right-Caudate)(rh.fusiform_7)(rh.inferiorparietal_9)(rh.insula_5)
0.00244	1.00E-05	0.83209	0.71635	(rh.inferiorparietal_9)(rh.isthmuscingulate_1)(rh.precuneus_2)(rh.superiortemporal_3)
0.00244	1.00E-05	0.83209	0.71635	(rh.fusiform_7)(rh.inferiorparietal_4)(rh.inferiorparietal_9)(rh.superiortemporal_3)
0.00244	1.00E-05	0.83209	0.71635	(rh.fusiform_7)(rh.parahippocampal_3)(rh.precuneus_4)(rh.supramarginal_9)
0.00244	1.00E-05	0.83209	0.71635	(rh.entorhinal_1)(rh.inferiorparietal_9)(rh.insula_2)(rh.precuneus_2)
0.00244	1.00E-05	0.83209	0.71635	(rh.entorhinal_1)(rh.inferiorparietal_9)(rh.isthmuscingulate_2)(rh.precuneus_2)
0.00244	1.00E-05	0.83209	0.71635	(Right-Caudate)(rh.fusiform_7)(rh.inferiorparietal_10)(rh.inferiorparietal_9)
0.00244	1.00E-05	0.83209	0.71635	(rh.fusiform_7)(rh.inferiorparietal_9)(rh.insula_4)(rh.superiortemporal_3)
0.00244	1.00E-05	0.83209	0.71635	(rh.inferiorparietal_9)(rh.precuneus_4)(rh.superiortemporal_3)(rh.supramarginal_9)
0.00244	1.00E-05	0.83209	0.71635	(Right-Thalamus-Proper)(rh.entorhinal_1)(rh.inferiorparietal_9)(rh.precuneus_2)
0.00244	1.00E-05	0.83209	0.71635	(Right-Pallidum)(rh.entorhinal_1)(rh.inferiorparietal_9)(rh.precuneus_2)
0.00244	1.00E-05	0.83209	0.71635	(rh.fusiform_7)(rh.inferiorparietal_10)(rh.inferiorparietal_9)(rh.parahippocampal_2)
0.00248	1.00E-05	0.89552	0.7963	(rh.inferiorparietal_9)(rh.precuneus_2)(rh.supramarginal_9)
0.00248	1.00E-05	0.89552	0.7963	(rh.inferiorparietal_10)(rh.inferiorparietal_9)(rh.insula_2)
0.00248	1.00E-05	0.89552	0.7963	(Right-Caudate)(rh.inferiorparietal_9)(rh.insula_2)(rh.precuneus_2)
0.00248	1.00E-05	0.89552	0.7963	(Right-Pallidum)(rh.inferiorparietal_9)(rh.parahippocampal_2)
0.00248	1.00E-05	0.89552	0.7963	(rh.inferiorparietal_9)(rh.insula_2)(rh.supramarginal_9)
0.00248	1.00E-05	0.89552	0.7963	(rh.inferiorparietal_10)(rh.inferiorparietal_9)(rh.precuneus_2)
0.00249	1.00E-05	0.83582	0.72115	(rh.bankssts_2)(rh.fusiform_7)(rh.inferiorparietal_4)(rh.inferiorparietal_9)
0.00249	1.00E-05	0.83582	0.72115	(rh.fusiform_7)(rh.inferiorparietal_9)(rh.insula_4)(rh.insula_5)
0.00249	1.00E-05	0.83582	0.72115	(rh.fusiform_7)(rh.inferiorparietal_4)(rh.inferiorparietal_9)(rh.lingual_7)
0.00249	1.00E-05	0.83582	0.72115	(Right-Caudate)(rh.fusiform_7)(rh.parahippocampal_3)(rh.precuneus_4)
0.00249	1.00E-05	0.83582	0.72115	(rh.fusiform_7)(rh.inferiorparietal_9)(rh.insula_4)(rh.lingual_7)
0.00249	1.00E-05	0.83582	0.72115	(rh.bankssts_2)(rh.fusiform_7)(rh.inferiorparietal_9)(rh.insula_4)
0.00249	1.00E-05	0.83582	0.72115	(Right-Caudate)(rh.fusiform_7)(rh.inferiorparietal_9)(rh.superiortemporal_9)

0.00249	1.00E-05	0.83582	0.72115	(rh.inferiorparietal_9)(rh.parahippocampal_2)(rh.precuneus_2)(rh.precuneus_4)
0.00253	1.00E-05	0.92164	0.83173	(rh.bankssts_2)(rh.fusiform_7)(rh.lingual_7)(rh.superiortemporal_3)
0.00255	1.00E-05	0.83955	0.72596	(rh.fusiform_7)(rh.inferiorparietal_9)(rh.insula_5)(rh.superiortemporal_9)
0.00255	1.00E-05	0.83955	0.72596	(rh.fusiform_7)(rh.inferiorparietal_10)(rh.inferiorparietal_9)(rh.insula_2)
0.00255	1.00E-05	0.83955	0.72596	(Right-Thalamus-Proper)(rh.fusiform_7)(rh.inferiorparietal_10)(rh.inferiorparietal_9)
0.00255	1.00E-05	0.83955	0.72596	(rh.fusiform_7)(rh.inferiorparietal_10)(rh.inferiorparietal_9)
0.00255	1.00E-05	0.83955	0.72596	(rh.fusiform_7)(rh.inferiorparietal_10)(rh.inferiorparietal_9)(rh.isthmuscingulate_2)
0.00255	1.00E-05	0.83955	0.72596	(rh.inferiorparietal_9)(rh.insula_2)(rh.precuneus_2)(rh.precuneus_4)
0.00255	1.00E-05	0.83955	0.72596	(Right-Putamen)(rh.fusiform_7)(rh.inferiorparietal_10)(rh.inferiorparietal_9)
0.00259	1.00E-05	0.88806	0.78704	(rh.inferiorparietal_10)(rh.inferiorparietal_9)(rh.supramarginal_9)
0.0026	1.00E-05	0.84328	0.73077	(rh.inferiorparietal_9)(rh.lingual_7)(rh.precuneus_4)(rh.superiortemporal_3)
0.0026	1.00E-05	0.84328	0.73077	(Right-Pallidum)(rh.fusiform_7)(rh.parahippocampal_3)(rh.precuneus_4)
0.0026	1.00E-05	0.84328	0.73077	(rh.bankssts_2)(rh.inferiorparietal_9)(rh.precuneus_4)(rh.superiortemporal_3)
0.0026	1.00E-05	0.84328	0.73077	(Right-Putamen)(rh.fusiform_7)(rh.parahippocampal_3)(rh.precuneus_4)
0.0026	1.00E-05	0.84328	0.73077	(Right-Thalamus-Proper)(rh.fusiform_7)(rh.parahippocampal_3)(rh.precuneus_4)
0.0026	1.00E-05	0.84328	0.73077	(rh.fusiform_7)(rh.isthmuscingulate_1)(rh.precuneus_2)(rh.superiortemporal_3)
0.0026	1.00E-05	0.84328	0.73077	(rh.fusiform_7)(rh.isthmuscingulate_2)(rh.parahippocampal_3)(rh.precuneus_4)
0.0026	1.00E-05	0.84328	0.73077	(rh.fusiform_7)(rh.insula_2)(rh.parahippocampal_3)(rh.precuneus_4)
0.00261	1.00E-05	0.91791	0.82692	(Right-Pallidum)(rh.fusiform_7)(rh.isthmuscingulate_2)(rh.precuneus_2)
0.00261	1.00E-05	0.91791	0.82692	(Right-Pallidum)(Right-Putamen)(rh.fusiform_7)(rh.precuneus_2)
0.00261	1.00E-05	0.91791	0.82692	(Right-Pallidum)(Right-Thalamus-Proper)(rh.fusiform_7)(rh.precuneus_2)
0.00261	1.00E-05	0.91791	0.82692	(Right-Pallidum)(rh.fusiform_7)(rh.insula_2)(rh.precuneus_2)
0.00261	1.00E-05	0.91791	0.82692	(Right-Pallidum)(rh.fusiform_7)(rh.precuneus_2)
0.00265	1.00E-05	0.84701	0.73558	(Right-Caudate)(rh.inferiorparietal_9)(rh.parahippocampal_3)(rh.precuneus_2)
0.00265	1.00E-05	0.84701	0.73558	(rh.bankssts_2)(rh.fusiform_7)(rh.isthmuscingulate_1)(rh.precuneus_2)
0.00265	1.00E-05	0.84701	0.73558	(rh.fusiform_7)(rh.precuneus_4)(rh.superiortemporal_3)(rh.supramarginal_9)
0.00265	1.00E-05	0.84701	0.73558	(rh.fusiform_7)(rh.insula_5)(rh.precuneus_2)(rh.precuneus_4)
0.0027	1.00E-05	0.85075	0.74038	(Right-Accumbens-area)(rh.fusiform_7)(rh.insula_2)(rh.precuneus_2)
0.0027	1.00E-05	0.85075	0.74038	(rh.bankssts_1)(rh.inferiorparietal_9)(rh.precuneus_2)(rh.superiortemporal_3)
0.0027	1.00E-05	0.85075	0.74038	(rh.fusiform_7)(rh.lingual_7)(rh.precuneus_4)(rh.supramarginal_9)
0.0027	1.00E-05	0.85075	0.74038	(Right-Accumbens-area)(Right-Pallidum)(rh.fusiform_7)(rh.precuneus_2)
0.0027	1.00E-05	0.85075	0.74038	(Right-Accumbens-area)(rh.fusiform_7)(rh.isthmuscingulate_2)(rh.precuneus_2)
0.0027	1.00E-05	0.85075	0.74038	(Right-Accumbens-area)(Right-Putamen)(rh.fusiform_7)(rh.precuneus_2)
0.0027	1.00E-05	0.85075	0.74038	(Right-Accumbens-area)(Right-Thalamus-Proper)(rh.fusiform_7)(rh.precuneus_2)
0.0027	1.00E-05	0.85075	0.74038	(rh.bankssts_2)(rh.fusiform_7)(rh.precuneus_4)(rh.supramarginal_9)

0.0027	1.00E-05	0.85075	0.74038	(Right-Accumbens-area)(rh.fusiform_7)(rh.precuneus_2)
0.0027	1.00E-05	0.85075	0.74038	(rh.inferiorparietal_9)(rh.parahippocampal_3)(rh.precuneus_2)(rh.superiortemporal_9)
0.00271	1.00E-05	0.83582	0.72222	(rh.inferiorparietal_9)(rh.precuneus_4)(rh.supramarginal_9)
0.00274	1.00E-05	0.85448	0.74519	(rh.fusiform_7)(rh.fusiform_8)(rh.precuneus_2)(rh.superiortemporal_6)
0.00275	1.00E-05	0.97761	0.91827	(Right-Putamen)(rh.parahippocampal_2)(rh.precuneus_2)(rh.superiortemporal_3)
0.00275	1.00E-05	0.97761	0.91827	(rh.parahippocampal_2)(rh.precuneus_2)(rh.superiortemporal_3)
0.00275	1.00E-05	0.97761	0.91827	(Right-Thalamus-Proper)(rh.parahippocampal_2)(rh.precuneus_2)(rh.superiortemporal_3
0.00275	1.00E-05	0.97761	0.91827	(rh.isthmuscingulate_2)(rh.parahippocampal_2)(rh.precuneus_2)(rh.superiortemporal_3)
0.00275	1.00E-05	0.91045	0.81731	(rh.bankssts_2)(rh.inferiorparietal_9)(rh.precuneus_2)
0.00275	1.00E-05	0.91045	0.81731	(rh.inferiorparietal_9)(rh.isthmuscingulate_2)(rh.superiortemporal_3)
0.00275	1.00E-05	0.91045	0.81731	(Right-Putamen)(rh.inferiorparietal_9)(rh.lingual_7)(rh.precuneus_2)
0.00275	1.00E-05	0.91045	0.81731	(Right-Putamen)(rh.inferiorparietal_9)(rh.isthmuscingulate_2)(rh.superiortemporal_3)
0.00275	1.00E-05	0.91045	0.81731	(rh.inferiorparietal_9)(rh.isthmuscingulate_2)(rh.lingual_7)(rh.precuneus_2)
0.00275	1.00E-05	0.91045	0.81731	(Right-Thalamus-Proper)(rh.inferiorparietal_9)(rh.superiortemporal_3)
0.00275	1.00E-05	0.91045	0.81731	(Right-Putamen)(rh.inferiorparietal_9)(rh.superiortemporal_3)
0.00275	1.00E-05	0.91045	0.81731	(Right-Putamen)(rh.bankssts_2)(rh.inferiorparietal_9)(rh.precuneus_2)
0.00275	1.00E-05	0.91045	0.81731	(rh.fusiform_7)(rh.insula_5)(rh.lingual_7)(rh.precuneus_2)
0.00275	1.00E-05	0.91045	0.81731	(Right-Caudate)(rh.fusiform_7)(rh.insula_2)(rh.precuneus_2)
0.00275	1.00E-05	0.91045	0.81731	(Right-Caudate)(Right-Putamen)(rh.fusiform_7)(rh.precuneus_2)
0.00275	1.00E-05	0.91045	0.81731	(Right-Caudate)(rh.fusiform_7)(rh.precuneus_2)
0.00275	1.00E-05	0.91045	0.81731	(Right-Putamen)(Right-Thalamus-Proper)(rh.inferiorparietal_9)(rh.superiortemporal_3)
0.00275	1.00E-05	0.91045	0.81731	(rh.inferiorparietal_9)(rh.superiortemporal_3)
0.00275	1.00E-05	0.91045	0.81731	(rh.inferiorparietal_9)(rh.lingual_7)(rh.precuneus_2)
0.00275	1.00E-05	0.91045	0.81731	(Right-Thalamus-Proper)(rh.bankssts_2)(rh.inferiorparietal_9)(rh.precuneus_2)
0.00275	1.00E-05	0.91045	0.81731	(Right-Thalamus-Proper)(rh.inferiorparietal_9)(rh.isthmuscingulate_2)(rh.superiortempor
0.00275	1.00E-05	0.91045	0.81731	(rh.bankssts_2)(rh.inferiorparietal_9)(rh.isthmuscingulate_2)(rh.precuneus_2)
0.00275	1.00E-05	0.91045	0.81731	(Right-Thalamus-Proper)(rh.inferiorparietal_9)(rh.lingual_7)(rh.precuneus_2)
0.00275	1.00E-05	0.91045	0.81731	(rh.bankssts_2)(rh.fusiform_7)(rh.insula_5)(rh.precuneus_2)
0.00275	1.00E-05	0.91045	0.81731	(Right-Caudate)(rh.fusiform_7)(rh.isthmuscingulate_2)(rh.precuneus_2)
0.00275	1.00E-05	0.91045	0.81731	(Right-Caudate)(Right-Thalamus-Proper)(rh.fusiform_7)(rh.precuneus_2)
0.00278	1.00E-05	0.85821	0.75	(rh.fusiform_7)(rh.lingual_7)(rh.precuneus_4)(rh.superiortemporal_3)
0.00278	1.00E-05	0.85821	0.75	(rh.bankssts_2)(rh.fusiform_7)(rh.precuneus_4)(rh.superiortemporal_3)
0.0028	1.00E-05	0.90672	0.8125	(rh.fusiform_7)(rh.precuneus_2)(rh.supramarginal_9)
0.0028	1.00E-05	0.90672	0.8125	(rh.fusiform_7)(rh.insula_2)(rh.precuneus_2)(rh.supramarginal_9)
0.0028	1.00E-05	0.90672	0.8125	(rh.fusiform_7)(rh.insula_2)(rh.lingual_7)(rh.parahippocampal_3)

0.0028	1.00E-05	0.90672	0.8125	(Right-Pallidum)(rh.fusiform_7)(rh.lingual_7)(rh.parahippocampal_3)
0.0028	1.00E-05	0.90672	0.8125	(Right-Putamen)(rh.fusiform_7)(rh.lingual_7)(rh.parahippocampal_3)
0.0028	1.00E-05	0.90672	0.8125	(rh.bankssts_2)(rh.fusiform_7)(rh.insula_2)(rh.parahippocampal_3)
0.0028	1.00E-05	0.90672	0.8125	(Right-Thalamus-Proper)(rh.fusiform_7)(rh.lingual_7)(rh.parahippocampal_3)
0.0028	1.00E-05	0.90672	0.8125	(rh.fusiform_7)(rh.insula_5)(rh.precuneus_2)(rh.superiortemporal_3)
0.0028	1.00E-05	0.90672	0.8125	(rh.fusiform_7)(rh.isthmuscingulate_2)(rh.lingual_7)(rh.parahippocampal_3)
0.0028	1.00E-05	0.90672	0.8125	(rh.bankssts_2)(rh.fusiform_7)(rh.parahippocampal_3)
0.0028	1.00E-05	0.90672	0.8125	(Right-Putamen)(rh.fusiform_7)(rh.precuneus_2)(rh.supramarginal_9)
0.0028	1.00E-05	0.90672	0.8125	(Right-Thalamus-Proper)(rh.bankssts_2)(rh.fusiform_7)(rh.parahippocampal_3)
0.0028	1.00E-05	0.90672	0.8125	(Right-Putamen)(rh.bankssts_2)(rh.fusiform_7)(rh.parahippocampal_3)
0.0028	1.00E-05	0.90672	0.8125	(Right-Thalamus-Proper)(rh.fusiform_7)(rh.precuneus_2)(rh.supramarginal_9)
0.0028	1.00E-05	0.90672	0.8125	(Right-Pallidum)(rh.bankssts_2)(rh.fusiform_7)(rh.parahippocampal_3)
0.0028	1.00E-05	0.90672	0.8125	(Right-Putamen)(rh.inferiorparietal_9)(rh.insula_2)(rh.superiortemporal_3)
0.0028	1.00E-05	0.90672	0.8125	(rh.bankssts_2)(rh.fusiform_7)(rh.isthmuscingulate_2)(rh.parahippocampal_3)
0.0028	1.00E-05	0.90672	0.8125	(rh.fusiform_7)(rh.isthmuscingulate_2)(rh.precuneus_2)(rh.supramarginal_9)
0.0028	1.00E-05	0.90672	0.8125	(Right-Pallidum)(rh.fusiform_7)(rh.parahippocampal_2)(rh.precuneus_2)
0.0028	1.00E-05	0.90672	0.8125	(rh.inferiorparietal_9)(rh.insula_2)(rh.isthmuscingulate_2)(rh.superiortemporal_3)
0.0028	1.00E-05	0.90672	0.8125	(rh.fusiform_7)(rh.lingual_7)(rh.parahippocampal_3)
0.0028	1.00E-05	0.90672	0.8125	(rh.inferiorparietal_9)(rh.insula_2)(rh.superiortemporal_3)
0.0028	1.00E-05	0.90672	0.8125	(Right-Thalamus-Proper)(rh.inferiorparietal_9)(rh.insula_2)(rh.superiortemporal_3)
0.00281	1.00E-05	0.80224	0.68269	(rh.inferiorparietal_9)(rh.lingual_7)(rh.parahippocampal_3)(rh.precuneus_4)
0.00281	1.00E-05	0.80224	0.68269	(rh.bankssts_2)(rh.inferiorparietal_9)(rh.parahippocampal_3)(rh.precuneus_4)
0.00281	1.00E-05	0.80224	0.68269	(rh.fusiform_7)(rh.isthmuscingulate_1)(rh.parahippocampal_2)(rh.precuneus_4)
0.00282	1.00E-05	0.86194	0.75481	(rh.bankssts_2)(rh.fusiform_7)(rh.lingual_7)(rh.precuneus_4)
0.00285	1.00E-05	0.90299	0.80769	(rh.fusiform_7)(rh.isthmuscingulate_2)(rh.parahippocampal_3)(rh.superiortemporal_3)
0.00285	1.00E-05	0.90299	0.80769	(Right-Pallidum)(rh.fusiform_7)(rh.parahippocampal_3)(rh.superiortemporal_3)
0.00285	1.00E-05	0.90299	0.80769	(rh.fusiform_7)(rh.parahippocampal_3)(rh.superiortemporal_3)
0.00285	1.00E-05	0.90299	0.80769	(rh.inferiorparietal_9)(rh.insula_2)(rh.lingual_7)(rh.parahippocampal_2)
0.00285	1.00E-05	0.90299	0.80769	(Right-Thalamus-Proper)(rh.fusiform_7)(rh.parahippocampal_3)(rh.superiortemporal_3)
0.00285	1.00E-05	0.90299	0.80769	(rh.fusiform_7)(rh.insula_2)(rh.parahippocampal_3)(rh.superiortemporal_3)
0.00285	1.00E-05	0.90299	0.80769	(Right-Putamen)(rh.fusiform_7)(rh.parahippocampal_3)(rh.superiortemporal_3)
0.00285	1.00E-05	0.90299	0.80769	(rh.bankssts_2)(rh.inferiorparietal_9)(rh.insula_2)(rh.parahippocampal_2)
0.00288	1.00E-05	0.89925	0.80288	(Right-Caudate)(rh.fusiform_7)(rh.precuneus_2)(rh.supramarginal_9)
0.00288	1.00E-05	0.89925	0.80288	(Right-Caudate)(rh.inferiorparietal_9)(rh.lingual_7)(rh.superiortemporal_3)
0.00288	1.00E-05	0.8694	0.76442	(rh.fusiform_7)(rh.precuneus_2)(rh.superiortemporal_3)(rh.superiortemporal_6)

0.00288	1.00E-05	0.8694	0.76442	(rh.entorhinal_1)(rh.fusiform_7)(rh.fusiform_8)(rh.precuneus_2)
0.0029	1.00E-05	0.87313	0.76923	(rh.inferiorparietal_9)(rh.insula_5)(rh.precuneus_2)
0.0029	1.00E-05	0.87313	0.76923	(rh.inferiorparietal_9)(rh.insula_2)(rh.insula_5)(rh.precuneus_2)
0.0029	1.00E-05	0.87313	0.76923	(rh.inferiorparietal_9)(rh.insula_5)(rh.isthmuscingulate_2)(rh.precuneus_2)
0.0029	1.00E-05	0.87313	0.76923	(Right-Thalamus-Proper)(rh.inferiorparietal_9)(rh.insula_5)(rh.precuneus_2)
0.0029	1.00E-05	0.87313	0.76923	(Right-Pallidum)(rh.inferiorparietal_9)(rh.insula_5)(rh.precuneus_2)
0.0029	1.00E-05	0.87313	0.76923	(Right-Putamen)(rh.inferiorparietal_9)(rh.insula_5)(rh.precuneus_2)
0.00291	1.00E-05	0.89552	0.79808	(rh.inferiorparietal_9)(rh.isthmuscingulate_2)(rh.superiortemporal_3)(rh.supramarginal_[illegible]
0.00291	1.00E-05	0.89552	0.79808	(rh.inferiorparietal_10)(rh.inferiorparietal_9)(rh.lingual_7)(rh.superiortemporal_3)
0.00291	1.00E-05	0.89552	0.79808	(Right-Thalamus-Proper)(rh.inferiorparietal_9)(rh.superiortemporal_3)(rh.supramarginal_
0.00291	1.00E-05	0.89552	0.79808	(rh.fusiform_7)(rh.parahippocampal_2)(rh.precuneus_2)(rh.supramarginal_9)
0.00291	1.00E-05	0.89552	0.79808	(Right-Putamen)(rh.inferiorparietal_9)(rh.superiortemporal_3)(rh.supramarginal_9)
0.00291	1.00E-05	0.89552	0.79808	(rh.bankssts_2)(rh.inferiorparietal_10)(rh.inferiorparietal_9)(rh.superiortemporal_3)
0.00292	1.00E-05	0.89179	0.79327	(rh.bankssts_2)(rh.fusiform_7)(rh.fusiform_8)(rh.precuneus_2)
0.00292	1.00E-05	0.89179	0.79327	(rh.inferiorparietal_9)(rh.insula_2)(rh.precuneus_2)(rh.supramarginal_9)
0.00292	1.00E-05	0.89179	0.79327	(rh.inferiorparietal_9)(rh.insula_2)(rh.superiortemporal_3)(rh.supramarginal_9)
0.00292	1.00E-05	0.89179	0.79327	(rh.inferiorparietal_10)(rh.inferiorparietal_9)(rh.insula_2)(rh.precuneus_2)
0.00292	1.00E-05	0.89179	0.79327	(rh.fusiform_7)(rh.fusiform_8)(rh.lingual_7)(rh.precuneus_2)
0.00293	1.00E-05	0.8806	0.77885	(rh.entorhinal_1)(rh.fusiform_7)(rh.insula_2)(rh.precuneus_2)
0.00293	1.00E-05	0.8806	0.77885	(rh.entorhinal_1)(rh.fusiform_7)(rh.precuneus_2)
0.00293	1.00E-05	0.8806	0.77885	(rh.entorhinal_1)(rh.fusiform_7)(rh.isthmuscingulate_2)(rh.precuneus_2)
0.00293	1.00E-05	0.8806	0.77885	(rh.entorhinal_1)(rh.fusiform_7)(rh.precuneus_2)(rh.superiortemporal_9)
0.00293	1.00E-05	0.8806	0.77885	(Right-Thalamus-Proper)(rh.entorhinal_1)(rh.fusiform_7)(rh.precuneus_2)
0.00293	1.00E-05	0.8806	0.77885	(Right-Pallidum)(rh.entorhinal_1)(rh.fusiform_7)(rh.precuneus_2)
0.00293	1.00E-05	0.8806	0.77885	(Right-Putamen)(rh.entorhinal_1)(rh.fusiform_7)(rh.precuneus_2)
0.00293	1.00E-05	0.88806	0.78846	(rh.fusiform_7)(rh.fusiform_8)(rh.precuneus_2)(rh.superiortemporal_3)
0.00293	1.00E-05	0.88806	0.78846	(Right-Caudate)(rh.inferiorparietal_9)(rh.superiortemporal_3)(rh.supramarginal_9)
0.00293	1.00E-05	0.88806	0.78846	(rh.inferiorparietal_10)(rh.inferiorparietal_9)(rh.parahippocampal_2)(rh.superiortempora
0.00293	1.00E-05	0.88806	0.78846	(rh.fusiform_7)(rh.fusiform_8)(rh.lingual_7)(rh.parahippocampal_3)
0.00293	1.00E-05	0.88806	0.78846	(rh.bankssts_2)(rh.fusiform_7)(rh.fusiform_8)(rh.parahippocampal_3)
0.00293	1.00E-05	0.88806	0.78846	(rh.fusiform_7)(rh.insula_4)(rh.parahippocampal_3)(rh.precuneus_2)
0.00293	1.00E-05	0.88806	0.78846	(rh.inferiorparietal_9)(rh.parahippocampal_2)(rh.precuneus_2)(rh.supramarginal_9)
0.00294	1.00E-05	0.88433	0.78365	(rh.fusiform_7)(rh.fusiform_8)(rh.parahippocampal_3)(rh.superiortemporal_3)
0.00294	1.00E-05	0.88433	0.78365	(rh.inferiorparietal_10)(rh.inferiorparietal_9)(rh.superiortemporal_3)(rh.supramarginal_9
0.00294	1.00E-05	0.88433	0.78365	(rh.inferiorparietal_4)(rh.inferiorparietal_9)(rh.precuneus_2)(rh.superiortemporal_3)

0.00294	1.00E-05	0.88433	0.78365	(rh.fusiform_7)(rh.inferiorparietal_10)(rh.parahippocampal_3)(rh.precuneus_2)
0.00296	1.00E-05	0.98507	0.93269	(rh.bankssts_2)(rh.insula_2)(rh.precuneus_2)(rh.superiortemporal_3)
0.00296	1.00E-05	0.98507	0.93269	(rh.insula_2)(rh.lingual_7)(rh.precuneus_2)(rh.superiortemporal_3)
0.003	1.00E-05	0.8097	0.69231	(Right-Pallidum)(rh.fusiform_7)(rh.isthmuscingulate_1)(rh.precuneus_4)
0.00309	1.00E-05	0.81343	0.69712	(rh.fusiform_7)(rh.inferiorparietal_9)(rh.precuneus_3)(rh.supramarginal_9)
0.00309	1.00E-05	0.81343	0.69712	(Right-Caudate)(rh.fusiform_7)(rh.fusiform_8)(rh.inferiorparietal_9)
0.00319	1.00E-05	0.81716	0.70192	(rh.entorhinal_1)(rh.inferiorparietal_9)(rh.precuneus_2)(rh.supramarginal_9)
0.00319	1.00E-05	0.81716	0.70192	(Right-Accumbens-area)(rh.fusiform_7)(rh.fusiform_8)(rh.superiortemporal_6)
0.00328	1.00E-05	0.8209	0.70673	(rh.fusiform_7)(rh.inferiorparietal_9)(rh.parahippocampal_2)(rh.precuneus_3)
0.00328	1.00E-05	0.8209	0.70673	(rh.fusiform_7)(rh.inferiorparietal_4)(rh.inferiorparietal_9)(rh.supramarginal_9)
0.00328	1.00E-05	0.8209	0.70673	(rh.fusiform_7)(rh.inferiorparietal_9)(rh.insula_4)(rh.supramarginal_9)
0.00328	1.00E-05	0.8209	0.70673	(rh.entorhinal_1)(rh.inferiorparietal_9)(rh.insula_4)(rh.precuneus_2)
0.00328	1.00E-05	0.8209	0.70673	(rh.fusiform_7)(rh.fusiform_8)(rh.inferiorparietal_9)(rh.insula_5)
0.00338	1.00E-05	0.97388	0.91346	(rh.insula_2)(rh.parahippocampal_2)(rh.precuneus_2)(rh.superiortemporal_3)
0.00346	1.00E-05	0.82836	0.71635	(rh.fusiform_7)(rh.inferiorparietal_9)(rh.isthmuscingulate_2)(rh.precuneus_3)
0.00346	1.00E-05	0.82836	0.71635	(rh.fusiform_7)(rh.inferiorparietal_9)(rh.insula_2)(rh.precuneus_3)
0.00346	1.00E-05	0.82836	0.71635	(rh.entorhinal_1)(rh.inferiorparietal_9)(rh.insula_5)(rh.precuneus_2)
0.00346	1.00E-05	0.82836	0.71635	(Right-Thalamus-Proper)(rh.fusiform_7)(rh.inferiorparietal_9)(rh.precuneus_3)
0.00346	1.00E-05	0.82836	0.71635	(Right-Putamen)(rh.fusiform_7)(rh.inferiorparietal_9)(rh.precuneus_3)
0.00346	1.00E-05	0.82836	0.71635	(rh.fusiform_7)(rh.inferiorparietal_4)(rh.inferiorparietal_9)(rh.parahippocampal_2)
0.00346	1.00E-05	0.82836	0.71635	(rh.fusiform_7)(rh.inferiorparietal_9)(rh.insula_4)(rh.parahippocampal_2)
0.00346	1.00E-05	0.82836	0.71635	(rh.fusiform_7)(rh.inferiorparietal_9)(rh.precuneus_3)
0.00355	1.00E-05	0.83209	0.72115	(rh.inferiorparietal_10)(rh.inferiorparietal_9)(rh.precuneus_4)(rh.superiortemporal_3)
0.00364	1.00E-05	0.83582	0.72596	(Right-Pallidum)(rh.fusiform_7)(rh.inferiorparietal_9)(rh.insula_4)
0.00364	1.00E-05	0.83582	0.72596	(Right-Thalamus-Proper)(rh.fusiform_7)(rh.inferiorparietal_4)(rh.inferiorparietal_9)
0.00364	1.00E-05	0.83582	0.72596	(rh.fusiform_7)(rh.inferiorparietal_4)(rh.inferiorparietal_9)(rh.insula_2)
0.00364	1.00E-05	0.83582	0.72596	(Right-Putamen)(rh.fusiform_7)(rh.inferiorparietal_9)(rh.insula_4)
0.00364	1.00E-05	0.83582	0.72596	(rh.fusiform_7)(rh.insula_5)(rh.parahippocampal_3)(rh.precuneus_4)
0.00364	1.00E-05	0.83582	0.72596	(rh.fusiform_7)(rh.inferiorparietal_4)(rh.inferiorparietal_9)(rh.isthmuscingulate_2)
0.00364	1.00E-05	0.83582	0.72596	(Right-Putamen)(rh.fusiform_7)(rh.inferiorparietal_4)(rh.inferiorparietal_9)
0.00364	1.00E-05	0.83582	0.72596	(Right-Pallidum)(rh.fusiform_7)(rh.inferiorparietal_10)(rh.inferiorparietal_9)
0.00364	1.00E-05	0.83582	0.72596	(rh.fusiform_7)(rh.inferiorparietal_9)(rh.insula_2)(rh.insula_4)
0.00364	1.00E-05	0.83582	0.72596	(Right-Caudate)(rh.inferiorparietal_9)(rh.precuneus_4)(rh.superiortemporal_3)
0.00364	1.00E-05	0.83582	0.72596	(rh.fusiform_7)(rh.inferiorparietal_9)(rh.insula_4)(rh.isthmuscingulate_2)
0.00364	1.00E-05	0.83582	0.72596	(Right-Thalamus-Proper)(rh.fusiform_7)(rh.inferiorparietal_9)(rh.insula_4)

0.00364	1.00E-05	0.83582	0.72596	(rh.fusiform_7)(rh.inferiorparietal_9)(rh.insula_4)
0.00373	1.00E-05	0.83955	0.73077	(Right-Caudate)(rh.fusiform_7)(rh.isthmuscingulate_1)(rh.precuneus_2)
0.00373	1.00E-05	0.83955	0.73077	(rh.fusiform_7)(rh.parahippocampal_2)(rh.precuneus_4)(rh.supramarginal_9)
0.00373	1.00E-05	0.83955	0.73077	(rh.inferiorparietal_9)(rh.insula_2)(rh.precuneus_4)(rh.superiortemporal_3)
0.0038	1.00E-05	0.89179	0.7963	(Right-Caudate)(rh.inferiorparietal_10)(rh.inferiorparietal_9)
0.0038	1.00E-05	0.89179	0.7963	(Right-Caudate)(Right-Pallidum)(rh.inferiorparietal_9)(rh.lingual_7)
0.0038	1.00E-05	0.89179	0.7963	(Right-Caudate)(rh.inferiorparietal_9)(rh.supramarginal_9)
0.0038	1.00E-05	0.89179	0.7963	(rh.inferiorparietal_4)(rh.inferiorparietal_9)(rh.insula_2)(rh.lingual_7)
0.00381	1.00E-05	0.84328	0.73558	(Right-Putamen)(rh.inferiorparietal_9)(rh.precuneus_4)(rh.superiortemporal_3)
0.00381	1.00E-05	0.84328	0.73558	(rh.inferiorparietal_9)(rh.precuneus_4)(rh.superiortemporal_3)
0.00381	1.00E-05	0.84328	0.73558	(rh.inferiorparietal_9)(rh.insula_4)(rh.parahippocampal_3)(rh.precuneus_2)
0.00381	1.00E-05	0.84328	0.73558	(rh.inferiorparietal_9)(rh.isthmuscingulate_2)(rh.precuneus_4)(rh.superiortemporal_3)
0.00381	1.00E-05	0.84328	0.73558	(rh.bankssts_2)(rh.inferiorparietal_9)(rh.precuneus_2)(rh.precuneus_4)
0.00381	1.00E-05	0.84328	0.73558	(rh.inferiorparietal_9)(rh.lingual_7)(rh.precuneus_2)(rh.precuneus_4)
0.00381	1.00E-05	0.84328	0.73558	(rh.inferiorparietal_9)(rh.parahippocampal_3)(rh.precuneus_2)(rh.supramarginal_9)
0.00381	1.00E-05	0.84328	0.73558	(Right-Thalamus-Proper)(rh.inferiorparietal_9)(rh.precuneus_4)(rh.superiortemporal_3)
0.00381	1.00E-05	0.84328	0.73558	(rh.inferiorparietal_4)(rh.inferiorparietal_9)(rh.parahippocampal_3)(rh.precuneus_2)
0.00381	1.00E-05	0.84328	0.73558	(rh.inferiorparietal_10)(rh.inferiorparietal_9)(rh.parahippocampal_3)(rh.precuneus_2)
0.00381	1.00E-05	0.84328	0.73558	(Right-Caudate)(rh.fusiform_7)(rh.precuneus_4)(rh.supramarginal_9)
0.00384	1.00E-05	0.92537	0.84135	(Right-Thalamus-Proper)(rh.bankssts_2)(rh.fusiform_7)(rh.lingual_7)
0.00384	1.00E-05	0.92537	0.84135	(rh.bankssts_2)(rh.fusiform_7)(rh.insula_2)(rh.lingual_7)
0.00384	1.00E-05	0.92537	0.84135	(rh.bankssts_2)(rh.fusiform_7)(rh.lingual_7)
0.00384	1.00E-05	0.92537	0.84135	(rh.bankssts_2)(rh.fusiform_7)(rh.isthmuscingulate_2)(rh.lingual_7)
0.00384	1.00E-05	0.92537	0.84135	(Right-Putamen)(rh.bankssts_2)(rh.fusiform_7)(rh.lingual_7)
0.00389	1.00E-05	0.84701	0.74038	(rh.fusiform_7)(rh.isthmuscingulate_1)(rh.isthmuscingulate_2)(rh.precuneus_2)
0.00389	1.00E-05	0.84701	0.74038	(rh.fusiform_7)(rh.parahippocampal_3)(rh.superiortemporal_10)(rh.superiortemporal_3)
0.00389	1.00E-05	0.84701	0.74038	(Right-Accumbens-area)(rh.fusiform_7)(rh.insula_5)(rh.precuneus_2)
0.00389	1.00E-05	0.84701	0.74038	(Right-Accumbens-area)(rh.fusiform_7)(rh.precuneus_2)(rh.superiortemporal_9)
0.00389	1.00E-05	0.84701	0.74038	(rh.fusiform_7)(rh.fusiform_8)(rh.precuneus_2)(rh.superiortemporal_10)
0.00389	1.00E-05	0.84701	0.74038	(rh.fusiform_7)(rh.precuneus_2)(rh.precuneus_4)(rh.superiortemporal_9)
0.00389	1.00E-05	0.84701	0.74038	(Right-Caudate)(rh.fusiform_7)(rh.parahippocampal_2)(rh.precuneus_4)
0.00389	1.00E-05	0.84701	0.74038	(Right-Putamen)(rh.fusiform_7)(rh.isthmuscingulate_1)(rh.precuneus_2)
0.00389	1.00E-05	0.84701	0.74038	(rh.bankssts_1)(rh.inferiorparietal_9)(rh.parahippocampal_2)(rh.superiortemporal_3)
0.00389	1.00E-05	0.84701	0.74038	(rh.fusiform_7)(rh.parahippocampal_2)(rh.precuneus_4)(rh.superiortemporal_3)
0.00389	1.00E-05	0.84701	0.74038	(Right-Thalamus-Proper)(rh.fusiform_7)(rh.isthmuscingulate_1)(rh.precuneus_2)

0.00389	1.00E-05	0.84701	0.74038	(rh.fusiform_7)(rh.insula_2)(rh.isthmuscingulate_1)(rh.precuneus_2)
0.00394	1.00E-05	0.83955	0.73148	(rh.inferiorparietal_9)(rh.isthmuscingulate_1)
0.00396	1.00E-05	0.85075	0.74519	(rh.fusiform_7)(rh.insula_2)(rh.precuneus_4)(rh.supramarginal_9)
0.00396	1.00E-05	0.85075	0.74519	(Right-Thalamus-Proper)(rh.fusiform_7)(rh.precuneus_4)(rh.supramarginal_9)
0.00396	1.00E-05	0.85075	0.74519	(rh.fusiform_7)(rh.isthmuscingulate_2)(rh.precuneus_4)(rh.supramarginal_9)
0.00396	1.00E-05	0.85075	0.74519	(Right-Caudate)(rh.fusiform_7)(rh.precuneus_4)(rh.superiortemporal_3)
0.00396	1.00E-05	0.85075	0.74519	(rh.fusiform_7)(rh.fusiform_8)(rh.parahippocampal_3)(rh.superiortemporal_6)
0.00396	1.00E-05	0.85075	0.74519	(rh.bankssts_2)(rh.fusiform_7)(rh.parahippocampal_3)(rh.superiortemporal_10)
0.00396	1.00E-05	0.85075	0.74519	(rh.bankssts_2)(rh.fusiform_7)(rh.parahippocampal_2)(rh.precuneus_4)
0.00396	1.00E-05	0.85075	0.74519	(Right-Putamen)(rh.fusiform_7)(rh.precuneus_4)(rh.supramarginal_9)
0.00397	1.00E-05	0.92164	0.83654	(rh.bankssts_2)(rh.fusiform_7)(rh.insula_2)(rh.superiortemporal_3)
0.00397	1.00E-05	0.92164	0.83654	(rh.fusiform_7)(rh.isthmuscingulate_2)(rh.lingual_7)(rh.superiortemporal_3)
0.00397	1.00E-05	0.92164	0.83654	(rh.bankssts_2)(rh.fusiform_7)(rh.superiortemporal_3)
0.00397	1.00E-05	0.92164	0.83654	(Right-Thalamus-Proper)(rh.fusiform_7)(rh.lingual_7)(rh.superiortemporal_3)
0.00397	1.00E-05	0.92164	0.83654	(rh.bankssts_2)(rh.fusiform_7)(rh.isthmuscingulate_2)(rh.superiortemporal_3)
0.00397	1.00E-05	0.92164	0.83654	(rh.fusiform_7)(rh.lingual_7)(rh.superiortemporal_3)
0.00397	1.00E-05	0.92164	0.83654	(Right-Thalamus-Proper)(rh.bankssts_2)(rh.fusiform_7)(rh.superiortemporal_3)
0.00397	1.00E-05	0.92164	0.83654	(rh.fusiform_7)(rh.insula_2)(rh.lingual_7)(rh.superiortemporal_3)
0.00397	1.00E-05	0.92164	0.83654	(Right-Putamen)(rh.fusiform_7)(rh.lingual_7)(rh.superiortemporal_3)
0.00397	1.00E-05	0.92164	0.83654	(Right-Putamen)(rh.bankssts_2)(rh.fusiform_7)(rh.superiortemporal_3)
0.00397	1.00E-05	0.98881	0.94231	(rh.isthmuscingulate_2)(rh.precuneus_2)(rh.superiortemporal_3)
0.00397	1.00E-05	0.98881	0.94231	(Right-Thalamus-Proper)(rh.precuneus_2)(rh.superiortemporal_3)
0.00397	1.00E-05	0.98881	0.94231	(Right-Thalamus-Proper)(rh.isthmuscingulate_2)(rh.precuneus_2)(rh.superiortemporal_3)
0.00397	1.00E-05	0.98881	0.94231	(Right-Putamen)(Right-Thalamus-Proper)(rh.precuneus_2)(rh.superiortemporal_3)
0.00397	1.00E-05	0.98881	0.94231	(Right-Putamen)(rh.precuneus_2)(rh.superiortemporal_3)
0.00397	1.00E-05	0.98881	0.94231	(rh.precuneus_2)(rh.superiortemporal_3)
0.00397	1.00E-05	0.98881	0.94231	(Right-Putamen)(rh.isthmuscingulate_2)(rh.precuneus_2)(rh.superiortemporal_3)
0.004	1.00E-05	0.84701	0.74074	(rh.inferiorparietal_9)(rh.parahippocampal_3)(rh.supramarginal_9)
0.004	1.00E-05	0.84701	0.74074	(rh.inferiorparietal_9)(rh.precuneus_4)
0.00403	1.00E-05	0.80224	0.6875	(Right-Accumbens-area)(rh.inferiorparietal_9)(rh.lingual_7)(rh.precuneus_2)
0.00403	1.00E-05	0.80224	0.6875	(rh.inferiorparietal_9)(rh.insula_2)(rh.parahippocampal_3)(rh.precuneus_4)
0.00403	1.00E-05	0.80224	0.6875	(Right-Accumbens-area)(rh.bankssts_2)(rh.inferiorparietal_9)(rh.precuneus_2)
0.00403	1.00E-05	0.80224	0.6875	(rh.inferiorparietal_9)(rh.parahippocampal_3)(rh.precuneus_4)
0.00403	1.00E-05	0.80224	0.6875	(Right-Putamen)(rh.inferiorparietal_9)(rh.parahippocampal_3)(rh.precuneus_4)
0.00403	1.00E-05	0.80224	0.6875	(rh.inferiorparietal_9)(rh.isthmuscingulate_2)(rh.parahippocampal_3)(rh.precuneus_4)

0.00403	1.00E-05	0.80224	0.6875	(Right-Accumbens-area)(rh.inferiorparietal_9)(rh.precuneus_2)(rh.superiortemporal_9)
0.00403	1.00E-05	0.80224	0.6875	(Right-Thalamus-Proper)(rh.inferiorparietal_9)(rh.parahippocampal_3)(rh.precuneus_4)
0.00403	1.00E-05	0.80224	0.6875	(rh.inferiorparietal_9)(rh.parahippocampal_2)(rh.precuneus_2)(rh.superiortemporal_10)
0.00403	1.00E-05	0.80224	0.6875	(Right-Pallidum)(rh.inferiorparietal_9)(rh.parahippocampal_3)(rh.precuneus_4)
0.00403	1.00E-05	0.80224	0.6875	(rh.fusiform_7)(rh.insula_5)(rh.isthmuscingulate_1)(rh.precuneus_4)
0.00404	1.00E-05	0.85448	0.75	(rh.fusiform_7)(rh.precuneus_2)(rh.superiortemporal_10)(rh.superiortemporal_3)
0.00404	1.00E-05	0.85448	0.75	(Right-Caudate)(rh.bankssts_2)(rh.fusiform_7)(rh.precuneus_4)
0.00405	1.00E-05	0.86194	0.75926	(rh.inferiorparietal_9)(rh.parahippocampal_3)
0.00411	1.00E-05	0.85821	0.75481	(rh.fusiform_7)(rh.insula_2)(rh.precuneus_4)(rh.superiortemporal_3)
0.00411	1.00E-05	0.85821	0.75481	(Right-Putamen)(rh.fusiform_7)(rh.precuneus_4)(rh.superiortemporal_3)
0.00411	1.00E-05	0.85821	0.75481	(rh.inferiorparietal_9)(rh.lingual_7)(rh.parahippocampal_3)(rh.superiortemporal_3)
0.00411	1.00E-05	0.85821	0.75481	(rh.bankssts_2)(rh.fusiform_7)(rh.precuneus_2)(rh.superiortemporal_10)
0.00411	1.00E-05	0.85821	0.75481	(rh.fusiform_7)(rh.precuneus_4)(rh.superiortemporal_3)
0.00411	1.00E-05	0.85821	0.75481	(rh.inferiorparietal_9)(rh.insula_5)(rh.precuneus_2)(rh.supramarginal_9)
0.00411	1.00E-05	0.85821	0.75481	(rh.fusiform_7)(rh.lingual_7)(rh.precuneus_2)(rh.superiortemporal_10)
0.00411	1.00E-05	0.85821	0.75481	(rh.inferiorparietal_10)(rh.inferiorparietal_9)(rh.insula_5)(rh.precuneus_2)
0.00411	1.00E-05	0.85821	0.75481	(rh.bankssts_2)(rh.inferiorparietal_9)(rh.parahippocampal_3)(rh.superiortemporal_3)
0.00411	1.00E-05	0.85821	0.75481	(rh.fusiform_7)(rh.isthmuscingulate_2)(rh.precuneus_4)(rh.superiortemporal_3)
0.00411	1.00E-05	0.85821	0.75481	(Right-Thalamus-Proper)(rh.fusiform_7)(rh.precuneus_4)(rh.superiortemporal_3)
0.00416	1.00E-05	0.91418	0.82692	(rh.bankssts_2)(rh.fusiform_7)(rh.lingual_7)(rh.parahippocampal_2)
0.00417	1.00E-05	0.86194	0.75962	(rh.inferiorparietal_9)(rh.parahippocampal_2)(rh.precuneus_2)(rh.superiortemporal_9)
0.00417	1.00E-05	0.86194	0.75962	(rh.fusiform_7)(rh.lingual_7)(rh.precuneus_4)
0.00417	1.00E-05	0.86194	0.75962	(Right-Putamen)(rh.bankssts_2)(rh.fusiform_7)(rh.precuneus_4)
0.00417	1.00E-05	0.86194	0.75962	(rh.bankssts_2)(rh.fusiform_7)(rh.insula_2)(rh.precuneus_4)
0.00417	1.00E-05	0.86194	0.75962	(Right-Caudate)(rh.inferiorparietal_9)(rh.insula_5)(rh.precuneus_2)
0.00417	1.00E-05	0.86194	0.75962	(Right-Thalamus-Proper)(rh.bankssts_2)(rh.fusiform_7)(rh.precuneus_4)
0.00417	1.00E-05	0.86194	0.75962	(rh.inferiorparietal_9)(rh.insula_4)(rh.insula_5)(rh.precuneus_2)
0.00417	1.00E-05	0.86194	0.75962	(rh.fusiform_7)(rh.insula_2)(rh.lingual_7)(rh.precuneus_4)
0.00417	1.00E-05	0.86194	0.75962	(Right-Thalamus-Proper)(rh.fusiform_7)(rh.lingual_7)(rh.precuneus_4)
0.00417	1.00E-05	0.86194	0.75962	(rh.fusiform_7)(rh.parahippocampal_3)(rh.superiortemporal_3)(rh.superiortemporal_6)
0.00417	1.00E-05	0.86194	0.75962	(rh.fusiform_7)(rh.isthmuscingulate_2)(rh.lingual_7)(rh.precuneus_4)
0.00417	1.00E-05	0.86194	0.75962	(rh.bankssts_2)(rh.fusiform_7)(rh.precuneus_4)
0.00417	1.00E-05	0.86194	0.75962	(rh.bankssts_2)(rh.fusiform_7)(rh.isthmuscingulate_2)(rh.precuneus_4)
0.00417	1.00E-05	0.86194	0.75962	(Right-Putamen)(rh.fusiform_7)(rh.lingual_7)(rh.precuneus_4)
0.00417	1.00E-05	0.86194	0.75962	(rh.bankssts_2)(rh.inferiorparietal_9)(rh.lingual_7)(rh.parahippocampal_3)

0.00417	1.00E-05	0.80597	0.69231	(Right-Accumbens-area)(rh.fusiform_7)(rh.parahippocampal_3)(rh.superiortemporal_10)
0.00417	1.00E-05	0.80597	0.69231	(rh.fusiform_7)(rh.inferiorparietal_9)(rh.parahippocampal_3)(rh.superiortemporal_1)
0.00423	1.00E-05	0.86567	0.76442	(rh.entorhinal_1)(rh.fusiform_7)(rh.precuneus_2)(rh.supramarginal_9)
0.00423	1.00E-05	0.86567	0.76442	(rh.inferiorparietal_9)(rh.insula_5)(rh.parahippocampal_2)(rh.superiortemporal_3)
0.00424	1.00E-05	0.91045	0.82212	(rh.fusiform_7)(rh.insula_5)(rh.isthmuscingulate_2)(rh.precuneus_2)
0.00424	1.00E-05	0.91045	0.82212	(rh.fusiform_7)(rh.lingual_7)(rh.parahippocampal_2)(rh.superiortemporal_3)
0.00424	1.00E-05	0.91045	0.82212	(rh.fusiform_7)(rh.insula_2)(rh.insula_5)(rh.precuneus_2)
0.00424	1.00E-05	0.91045	0.82212	(rh.bankssts_2)(rh.fusiform_7)(rh.lingual_7)(rh.supramarginal_9)
0.00424	1.00E-05	0.91045	0.82212	(Right-Putamen)(rh.inferiorparietal_9)(rh.precuneus_2)
0.00424	1.00E-05	0.91045	0.82212	(rh.fusiform_7)(rh.insula_5)(rh.precuneus_2)
0.00424	1.00E-05	0.91045	0.82212	(Right-Thalamus-Proper)(rh.inferiorparietal_9)(rh.isthmuscingulate_2)(rh.precuneus_2)
0.00424	1.00E-05	0.91045	0.82212	(rh.fusiform_7)(rh.lingual_7)(rh.precuneus_2)(rh.superiortemporal_9)
0.00424	1.00E-05	0.91045	0.82212	(rh.bankssts_2)(rh.fusiform_7)(rh.parahippocampal_2)(rh.superiortemporal_3)
0.00424	1.00E-05	0.91045	0.82212	(Right-Pallidum)(rh.fusiform_7)(rh.insula_5)(rh.precuneus_2)
0.00424	1.00E-05	0.91045	0.82212	(Right-Putamen)(rh.fusiform_7)(rh.insula_5)(rh.precuneus_2)
0.00424	1.00E-05	0.91045	0.82212	(Right-Thalamus-Proper)(rh.inferiorparietal_9)(rh.precuneus_2)
0.00424	1.00E-05	0.91045	0.82212	(Right-Putamen)(rh.inferiorparietal_9)(rh.isthmuscingulate_2)(rh.precuneus_2)
0.00424	1.00E-05	0.91045	0.82212	(Right-Thalamus-Proper)(rh.fusiform_7)(rh.insula_5)(rh.precuneus_2)
0.00424	1.00E-05	0.91045	0.82212	(rh.bankssts_2)(rh.inferiorparietal_9)(rh.insula_2)(rh.lingual_7)
0.00424	1.00E-05	0.91045	0.82212	(rh.inferiorparietal_9)(rh.precuneus_2)
0.00424	1.00E-05	0.91045	0.82212	(rh.inferiorparietal_9)(rh.isthmuscingulate_2)(rh.precuneus_2)
0.00424	1.00E-05	0.91045	0.82212	(rh.bankssts_2)(rh.fusiform_7)(rh.precuneus_2)(rh.superiortemporal_9)
0.00424	1.00E-05	0.91045	0.82212	(Right-Putamen)(Right-Thalamus-Proper)(rh.inferiorparietal_9)(rh.precuneus_2)
0.00428	1.00E-05	0.8694	0.76923	(rh.bankssts_2)(rh.inferiorparietal_9)(rh.insula_5)(rh.parahippocampal_2)
0.00428	1.00E-05	0.8694	0.76923	(rh.inferiorparietal_9)(rh.insula_5)(rh.lingual_7)(rh.parahippocampal_2)
0.00428	1.00E-05	0.8694	0.76923	(rh.fusiform_7)(rh.lingual_7)(rh.precuneus_2)(rh.superiortemporal_6)
0.00428	1.00E-05	0.8694	0.76923	(rh.bankssts_2)(rh.fusiform_7)(rh.precuneus_2)(rh.superiortemporal_6)
0.00428	1.00E-05	0.8694	0.76923	(rh.entorhinal_1)(rh.fusiform_7)(rh.parahippocampal_2)(rh.precuneus_2)
0.0043	1.00E-05	0.90672	0.81731	(rh.bankssts_2)(rh.inferiorparietal_9)(rh.lingual_7)(rh.parahippocampal_2)
0.0043	1.00E-05	0.90672	0.81731	(Right-Putamen)(rh.fusiform_7)(rh.insula_2)(rh.parahippocampal_3)
0.0043	1.00E-05	0.90672	0.81731	(Right-Pallidum)(Right-Thalamus-Proper)(rh.fusiform_7)(rh.parahippocampal_3)
0.0043	1.00E-05	0.90672	0.81731	(Right-Pallidum)(rh.fusiform_7)(rh.insula_2)(rh.parahippocampal_3)
0.0043	1.00E-05	0.90672	0.81731	(Right-Pallidum)(Right-Putamen)(rh.fusiform_7)(rh.parahippocampal_3)
0.0043	1.00E-05	0.90672	0.81731	(rh.fusiform_7)(rh.isthmuscingulate_2)(rh.parahippocampal_3)
0.0043	1.00E-05	0.90672	0.81731	(Right-Putamen)(rh.fusiform_7)(rh.parahippocampal_3)

0.0043	1.00E-05	0.90672	0.81731	(Right-Putamen)(Right-Thalamus-Proper)(rh.fusiform_7)(rh.parahippocampal_3)
0.0043	1.00E-05	0.90672	0.81731	(rh.fusiform_7)(rh.precuneus_2)(rh.superiortemporal_3)(rh.superiortemporal_9)
0.0043	1.00E-05	0.90672	0.81731	(rh.fusiform_7)(rh.lingual_7)(rh.superiortemporal_3)(rh.supramarginal_9)
0.0043	1.00E-05	0.90672	0.81731	(rh.fusiform_7)(rh.insula_2)(rh.isthmuscingulate_2)(rh.parahippocampal_3)
0.0043	1.00E-05	0.90672	0.81731	(Right-Caudate)(Right-Pallidum)(rh.fusiform_7)(rh.precuneus_2)
0.0043	1.00E-05	0.90672	0.81731	(Right-Thalamus-Proper)(rh.fusiform_7)(rh.parahippocampal_3)
0.0043	1.00E-05	0.90672	0.81731	(Right-Thalamus-Proper)(rh.fusiform_7)(rh.insula_2)(rh.parahippocampal_3)
0.0043	1.00E-05	0.90672	0.81731	(Right-Thalamus-Proper)(rh.fusiform_7)(rh.isthmuscingulate_2)(rh.parahippocampal_3)
0.0043	1.00E-05	0.90672	0.81731	(Right-Putamen)(rh.fusiform_7)(rh.isthmuscingulate_2)(rh.parahippocampal_3)
0.0043	1.00E-05	0.90672	0.81731	(rh.fusiform_7)(rh.parahippocampal_3)
0.0043	1.00E-05	0.90672	0.81731	(rh.bankssts_2)(rh.fusiform_7)(rh.superiortemporal_3)(rh.supramarginal_9)
0.0043	1.00E-05	0.90672	0.81731	(Right-Pallidum)(rh.fusiform_7)(rh.isthmuscingulate_2)(rh.parahippocampal_3)
0.0043	1.00E-05	0.90672	0.81731	(Right-Pallidum)(rh.fusiform_7)(rh.parahippocampal_3)
0.0043	1.00E-05	0.90672	0.81731	(rh.fusiform_7)(rh.insula_2)(rh.parahippocampal_3)
0.00431	1.00E-05	0.8097	0.69712	(rh.inferiorparietal_9)(rh.inferiortemporal_2)(rh.precuneus_2)(rh.superiortemporal_3)
0.00431	1.00E-05	0.8097	0.69712	(rh.inferiorparietal_9)(rh.lingual_7)(rh.precuneus_2)(rh.superiortemporal_10)
0.00431	1.00E-05	0.8097	0.69712	(rh.bankssts_2)(rh.inferiorparietal_9)(rh.precuneus_2)(rh.superiortemporal_10)
0.00432	1.00E-05	0.87313	0.77404	(rh.entorhinal_1)(rh.fusiform_7)(rh.parahippocampal_3)(rh.superiortemporal_3)
0.00435	1.00E-05	0.90299	0.8125	(rh.bankssts_2)(rh.fusiform_7)(rh.inferiorparietal_10)(rh.precuneus_2)
0.00435	1.00E-05	0.90299	0.8125	(Right-Pallidum)(rh.fusiform_7)(rh.precuneus_2)(rh.supramarginal_9)
0.00435	1.00E-05	0.90299	0.8125	(Right-Putamen)(rh.inferiorparietal_9)(rh.insula_2)(rh.parahippocampal_2)
0.00435	1.00E-05	0.90299	0.8125	(Right-Thalamus-Proper)(rh.inferiorparietal_9)(rh.insula_2)(rh.parahippocampal_2)
0.00435	1.00E-05	0.90299	0.8125	(rh.inferiorparietal_9)(rh.insula_2)(rh.isthmuscingulate_2)(rh.parahippocampal_2)
0.00435	1.00E-05	0.90299	0.8125	(rh.bankssts_2)(rh.fusiform_7)(rh.inferiorparietal_4)(rh.precuneus_2)
0.00435	1.00E-05	0.90299	0.8125	(rh.fusiform_7)(rh.inferiorparietal_10)(rh.lingual_7)(rh.precuneus_2)
0.00435	1.00E-05	0.90299	0.8125	(rh.fusiform_7)(rh.inferiorparietal_4)(rh.lingual_7)(rh.precuneus_2)
0.00436	1.00E-05	0.87687	0.77885	(rh.inferiorparietal_9)(rh.precuneus_2)(rh.superiortemporal_1)(rh.superiortemporal_3)
0.00436	1.00E-05	0.87687	0.77885	(rh.bankssts_2)(rh.entorhinal_1)(rh.fusiform_7)(rh.parahippocampal_3)
0.00436	1.00E-05	0.87687	0.77885	(rh.fusiform_7)(rh.parahippocampal_3)(rh.precuneus_2)(rh.precuneus_3)
0.00436	1.00E-05	0.87687	0.77885	(rh.entorhinal_1)(rh.fusiform_7)(rh.lingual_7)(rh.parahippocampal_3)
0.00436	1.00E-05	0.87687	0.77885	(rh.entorhinal_1)(rh.fusiform_7)(rh.insula_5)(rh.precuneus_2)
0.00438	1.00E-05	0.89925	0.80769	(rh.fusiform_7)(rh.inferiorparietal_10)(rh.precuneus_2)(rh.superiortemporal_3)
0.00438	1.00E-05	0.89925	0.80769	(rh.fusiform_7)(rh.insula_5)(rh.lingual_7)(rh.parahippocampal_3)
0.00438	1.00E-05	0.89925	0.80769	(rh.fusiform_7)(rh.inferiorparietal_4)(rh.precuneus_2)(rh.superiortemporal_3)
0.00438	1.00E-05	0.89925	0.80769	(Right-Caudate)(Right-Putamen)(rh.inferiorparietal_9)(rh.superiortemporal_3)

0.00438	1.00E-05	0.89925	0.80769	(Right-Caudate)(Right-Thalamus-Proper)(rh.inferiorparietal_9)(rh.superiortemporal_3)
0.00438	1.00E-05	0.89925	0.80769	(Right-Pallidum)(rh.inferiorparietal_9)(rh.insula_2)(rh.precuneus_2)
0.00438	1.00E-05	0.89925	0.80769	(Right-Caudate)(rh.bankssts_2)(rh.inferiorparietal_9)(rh.precuneus_2)
0.00438	1.00E-05	0.89925	0.80769	(rh.bankssts_2)(rh.fusiform_7)(rh.insula_5)(rh.parahippocampal_3)
0.00438	1.00E-05	0.89925	0.80769	(Right-Caudate)(rh.inferiorparietal_9)(rh.isthmuscingulate_2)(rh.superiortemporal_3)
0.00438	1.00E-05	0.89925	0.80769	(rh.fusiform_7)(rh.insula_5)(rh.parahippocampal_2)(rh.precuneus_2)
0.00439	1.00E-05	0.8806	0.78365	(rh.inferiorparietal_9)(rh.precuneus_2)(rh.precuneus_3)(rh.superiortemporal_3)
0.00441	1.00E-05	0.89552	0.80288	(Right-Caudate)(rh.inferiorparietal_9)(rh.insula_2)(rh.superiortemporal_3)
0.00441	1.00E-05	0.89552	0.80288	(rh.inferiorparietal_10)(rh.inferiorparietal_9)(rh.lingual_7)(rh.precuneus_2)
0.00441	1.00E-05	0.89552	0.80288	(rh.fusiform_7)(rh.insula_5)(rh.parahippocampal_3)(rh.superiortemporal_3)
0.00441	1.00E-05	0.89552	0.80288	(Right-Caudate)(rh.bankssts_2)(rh.fusiform_7)(rh.parahippocampal_3)
0.00441	1.00E-05	0.89552	0.80288	(rh.bankssts_2)(rh.inferiorparietal_9)(rh.precuneus_2)(rh.supramarginal_9)
0.00441	1.00E-05	0.89552	0.80288	(rh.inferiorparietal_10)(rh.inferiorparietal_9)(rh.isthmuscingulate_2)(rh.superiortemporal
0.00441	1.00E-05	0.89552	0.80288	(Right-Thalamus-Proper)(rh.inferiorparietal_10)(rh.inferiorparietal_9)(rh.superiortempora
0.00441	1.00E-05	0.89552	0.80288	(rh.bankssts_2)(rh.inferiorparietal_10)(rh.inferiorparietal_9)(rh.precuneus_2)
0.00441	1.00E-05	0.89552	0.80288	(Right-Caudate)(rh.inferiorparietal_9)(rh.insula_2)(rh.parahippocampal_2)
0.00441	1.00E-05	0.89552	0.80288	(Right-Caudate)(rh.fusiform_7)(rh.lingual_7)(rh.parahippocampal_3)
0.00441	1.00E-05	0.89552	0.80288	(Right-Putamen)(rh.inferiorparietal_10)(rh.inferiorparietal_9)(rh.superiortemporal_3)
0.00441	1.00E-05	0.89552	0.80288	(rh.inferiorparietal_9)(rh.lingual_7)(rh.precuneus_2)(rh.supramarginal_9)
0.00442	1.00E-05	0.88806	0.79327	(rh.inferiorparietal_10)(rh.inferiorparietal_9)(rh.parahippocampal_2)(rh.precuneus_2)
0.00442	1.00E-05	0.88806	0.79327	(Right-Thalamus-Proper)(rh.fusiform_7)(rh.fusiform_8)(rh.parahippocampal_3)
0.00442	1.00E-05	0.88806	0.79327	(Right-Caudate)(rh.inferiorparietal_10)(rh.inferiorparietal_9)(rh.superiortemporal_3)
0.00442	1.00E-05	0.88806	0.79327	(rh.inferiorparietal_9)(rh.insula_2)(rh.parahippocampal_2)(rh.supramarginal_9)
0.00442	1.00E-05	0.88806	0.79327	(rh.fusiform_7)(rh.fusiform_8)(rh.isthmuscingulate_2)(rh.parahippocampal_3)
0.00442	1.00E-05	0.88806	0.79327	(rh.fusiform_7)(rh.fusiform_8)(rh.parahippocampal_3)
0.00442	1.00E-05	0.88806	0.79327	(rh.fusiform_7)(rh.fusiform_8)(rh.insula_2)(rh.parahippocampal_3)
0.00442	1.00E-05	0.88806	0.79327	(rh.fusiform_7)(rh.parahippocampal_3)(rh.superiortemporal_3)(rh.supramarginal_9)
0.00442	1.00E-05	0.88806	0.79327	(Right-Pallidum)(rh.fusiform_7)(rh.fusiform_8)(rh.parahippocampal_3)
0.00442	1.00E-05	0.88806	0.79327	(Right-Putamen)(rh.fusiform_7)(rh.fusiform_8)(rh.parahippocampal_3)
0.00442	1.00E-05	0.89179	0.79808	(rh.inferiorparietal_10)(rh.inferiorparietal_9)(rh.insula_2)(rh.superiortemporal_3)
0.00442	1.00E-05	0.89179	0.79808	(rh.bankssts_2)(rh.fusiform_7)(rh.parahippocampal_3)(rh.supramarginal_9)
0.00442	1.00E-05	0.89179	0.79808	(Right-Thalamus-Proper)(rh.fusiform_7)(rh.fusiform_8)(rh.precuneus_2)
0.00442	1.00E-05	0.89179	0.79808	(rh.fusiform_7)(rh.fusiform_8)(rh.insula_2)(rh.precuneus_2)
0.00442	1.00E-05	0.89179	0.79808	(rh.fusiform_7)(rh.fusiform_8)(rh.isthmuscingulate_2)(rh.precuneus_2)
0.00442	1.00E-05	0.89179	0.79808	(rh.fusiform_7)(rh.fusiform_8)(rh.precuneus_2)

0.00442	1.00E-05	0.89179	0.79808	(Right-Pallidum)(rh.inferiorparietal_9)(rh.parahippocampal_2)(rh.superiortemporal_3)
0.00442	1.00E-05	0.89179	0.79808	(Right-Putamen)(rh.fusiform_7)(rh.fusiform_8)(rh.precuneus_2)
0.00442	1.00E-05	0.89179	0.79808	(Right-Pallidum)(rh.inferiorparietal_9)(rh.parahippocampal_2)(rh.precuneus_2)
0.00442	1.00E-05	0.89179	0.79808	(Right-Pallidum)(rh.fusiform_7)(rh.fusiform_8)(rh.precuneus_2)
0.00442	1.00E-05	0.89179	0.79808	(Right-Caudate)(rh.fusiform_7)(rh.parahippocampal_3)(rh.superiortemporal_3)
0.00442	1.00E-05	0.89179	0.79808	(rh.fusiform_7)(rh.lingual_7)(rh.parahippocampal_3)(rh.supramarginal_9)
0.00444	1.00E-05	0.81343	0.70192	(rh.fusiform_7)(rh.precuneus_4)(rh.precuneus_7)(rh.supramarginal_9)
0.00444	1.00E-05	0.81343	0.70192	(rh.inferiorparietal_9)(rh.inferiortemporal_2)(rh.lingual_7)(rh.precuneus_2)
0.00444	1.00E-05	0.81343	0.70192	(rh.entorhinal_1)(rh.fusiform_7)(rh.isthmuscingulate_1)(rh.precuneus_2)
0.00444	1.00E-05	0.81343	0.70192	(rh.fusiform_7)(rh.fusiform_8)(rh.inferiorparietal_9)(rh.insula_4)
0.00444	1.00E-05	0.81343	0.70192	(rh.bankssts_2)(rh.inferiorparietal_9)(rh.inferiortemporal_2)(rh.precuneus_2)
0.00444	1.00E-05	0.81343	0.70192	(Right-Accumbens-area)(rh.fusiform_7)(rh.parahippocampal_3)(rh.superiortemporal_6)
0.00444	1.00E-05	0.81343	0.70192	(rh.entorhinal_1)(rh.fusiform_7)(rh.parahippocampal_3)(rh.precuneus_4)
0.00458	1.00E-05	0.81716	0.70673	(rh.entorhinal_1)(rh.inferiorparietal_10)(rh.inferiorparietal_9)(rh.precuneus_2)
0.00458	1.00E-05	0.81716	0.70673	(rh.entorhinal_1)(rh.fusiform_8)(rh.inferiorparietal_9)(rh.precuneus_2)
0.00458	1.00E-05	0.81716	0.70673	(Right-Caudate)(rh.fusiform_7)(rh.inferiorparietal_9)(rh.precuneus_3)
0.00472	1.00E-05	0.8209	0.71154	(Right-Caudate)(rh.entorhinal_1)(rh.inferiorparietal_9)(rh.precuneus_2)
0.00472	1.00E-05	0.8209	0.71154	(rh.fusiform_7)(rh.precuneus_4)(rh.precuneus_7)(rh.superiortemporal_3)
0.00472	1.00E-05	0.8209	0.71154	(Right-Caudate)(rh.fusiform_7)(rh.precuneus_4)(rh.precuneus_7)
0.00472	1.00E-05	0.8209	0.71154	(rh.fusiform_7)(rh.inferiorparietal_9)(rh.superiortemporal_1)(rh.superiortemporal_3)
0.00486	1.00E-05	0.82463	0.71635	(rh.bankssts_2)(rh.fusiform_7)(rh.inferiorparietal_9)(rh.superiortemporal_1)
0.00486	1.00E-05	0.82463	0.71635	(rh.entorhinal_1)(rh.inferiorparietal_9)(rh.parahippocampal_3)(rh.superiortemporal_3)
0.00486	1.00E-05	0.82463	0.71635	(rh.fusiform_7)(rh.lingual_7)(rh.precuneus_4)(rh.precuneus_7)
0.00486	1.00E-05	0.82463	0.71635	(Right-Pallidum)(rh.fusiform_7)(rh.inferiorparietal_9)(rh.precuneus_3)
0.00486	1.00E-05	0.82463	0.71635	(Right-Caudate)(rh.fusiform_7)(rh.inferiorparietal_4)(rh.inferiorparietal_9)
0.00486	1.00E-05	0.82463	0.71635	(rh.fusiform_7)(rh.inferiorparietal_9)(rh.lingual_7)(rh.superiortemporal_1)
0.00486	1.00E-05	0.82463	0.71635	(rh.bankssts_2)(rh.fusiform_7)(rh.precuneus_4)(rh.precuneus_7)
0.00494	1.00E-05	0.97761	0.92308	(Right-Caudate)(rh.bankssts_2)(rh.precuneus_2)(rh.superiortemporal_3)
0.00494	1.00E-05	0.97761	0.92308	(Right-Caudate)(rh.lingual_7)(rh.precuneus_2)(rh.superiortemporal_3)
0.00499	1.00E-05	0.82836	0.72115	(rh.fusiform_7)(rh.isthmuscingulate_1)(rh.parahippocampal_3)(rh.precuneus_2)
0.00499	1.00E-05	0.82836	0.72115	(rh.fusiform_7)(rh.inferiorparietal_10)(rh.inferiorparietal_9)(rh.insula_5)
0.00499	1.00E-05	0.82836	0.72115	(rh.fusiform_7)(rh.inferiorparietal_4)(rh.inferiorparietal_9)(rh.insula_5)
0.00499	1.00E-05	0.82836	0.72115	(rh.fusiform_7)(rh.inferiorparietal_4)(rh.parahippocampal_3)(rh.precuneus_4)
0.00499	1.00E-05	0.82836	0.72115	(rh.entorhinal_1)(rh.inferiorparietal_9)(rh.lingual_7)(rh.parahippocampal_3)
0.00499	1.00E-05	0.82836	0.72115	(rh.bankssts_2)(rh.entorhinal_1)(rh.inferiorparietal_9)(rh.parahippocampal_3)

0.00513	1.00E-05	0.83209	0.72596	(rh.fusiform_7)(rh.parahippocampal_2)(rh.parahippocampal_3)(rh.precuneus_4)
0.00513	1.00E-05	0.83209	0.72596	(rh.entorhinal_1)(rh.inferiorparietal_9)(rh.lingual_7)(rh.superiortemporal_3)
0.00513	1.00E-05	0.83209	0.72596	(Right-Pallidum)(rh.fusiform_7)(rh.inferiorparietal_4)(rh.inferiorparietal_9)
0.00513	1.00E-05	0.83209	0.72596	(rh.fusiform_7)(rh.inferiorparietal_9)(rh.insula_4)(rh.superiortemporal_9)
0.00513	1.00E-05	0.83209	0.72596	(rh.inferiorparietal_9)(rh.precuneus_2)(rh.precuneus_4)(rh.supramarginal_9)
0.00513	1.00E-05	0.83209	0.72596	(rh.bankssts_2)(rh.entorhinal_1)(rh.inferiorparietal_9)(rh.superiortemporal_3)
0.00513	1.00E-05	0.83209	0.72596	(rh.fusiform_7)(rh.inferiorparietal_10)(rh.inferiorparietal_9)(rh.superiortemporal_9)
0.00513	1.00E-05	0.83209	0.72596	(rh.fusiform_8)(rh.inferiorparietal_9)(rh.parahippocampal_3)(rh.superiortemporal_3)
0.00513	1.00E-05	0.83209	0.72596	(rh.fusiform_8)(rh.inferiorparietal_9)(rh.parahippocampal_2)(rh.precuneus_2)
0.00513	1.00E-05	0.83209	0.72596	(rh.inferiorparietal_10)(rh.inferiorparietal_9)(rh.precuneus_2)(rh.precuneus_4)
0.00526	1.00E-05	0.83582	0.73077	(Right-Caudate)(rh.inferiorparietal_9)(rh.precuneus_2)(rh.precuneus_4)
0.00526	1.00E-05	0.83582	0.73077	(rh.fusiform_7)(rh.parahippocampal_3)(rh.precuneus_4)(rh.superiortemporal_9)
0.00526	1.00E-05	0.83582	0.73077	(rh.fusiform_8)(rh.inferiorparietal_9)(rh.lingual_7)(rh.parahippocampal_3)
0.00526	1.00E-05	0.83582	0.73077	(rh.entorhinal_1)(rh.fusiform_7)(rh.precuneus_2)(rh.superiortemporal_6)
0.00526	1.00E-05	0.83582	0.73077	(rh.bankssts_2)(rh.entorhinal_1)(rh.inferiorparietal_9)(rh.lingual_7)
0.00526	1.00E-05	0.83582	0.73077	(rh.fusiform_7)(rh.isthmuscingulate_1)(rh.parahippocampal_2)(rh.precuneus_2)
0.00526	1.00E-05	0.83582	0.73077	(rh.inferiorparietal_9)(rh.insula_2)(rh.parahippocampal_2)(rh.precuneus_4)
0.00526	1.00E-05	0.83582	0.73077	(rh.fusiform_8)(rh.inferiorparietal_9)(rh.precuneus_2)(rh.superiortemporal_3)
0.00526	1.00E-05	0.83582	0.73077	(rh.bankssts_2)(rh.fusiform_8)(rh.inferiorparietal_9)(rh.parahippocampal_3)
0.00539	1.00E-05	0.83955	0.73558	(rh.fusiform_8)(rh.inferiorparietal_9)(rh.lingual_7)(rh.precuneus_2)
0.00539	1.00E-05	0.83955	0.73558	(rh.bankssts_2)(rh.fusiform_8)(rh.inferiorparietal_9)(rh.precuneus_2)
0.00552	1.00E-05	0.84328	0.74038	(Right-Thalamus-Proper)(rh.inferiorparietal_9)(rh.precuneus_2)(rh.precuneus_4)
0.00552	1.00E-05	0.84328	0.74038	(rh.inferiorparietal_9)(rh.isthmuscingulate_2)(rh.precuneus_2)(rh.precuneus_4)
0.00552	1.00E-05	0.84328	0.74038	(Right-Pallidum)(rh.fusiform_7)(rh.isthmuscingulate_1)(rh.precuneus_2)
0.00552	1.00E-05	0.84328	0.74038	(rh.inferiorparietal_9)(rh.precuneus_2)(rh.precuneus_7)(rh.superiortemporal_3)
0.00552	1.00E-05	0.84328	0.74038	(rh.inferiorparietal_9)(rh.precuneus_2)(rh.precuneus_4)
0.00552	1.00E-05	0.84328	0.74038	(Right-Putamen)(rh.inferiorparietal_9)(rh.precuneus_2)(rh.precuneus_4)
0.00552	1.00E-05	0.84328	0.74038	(Right-Accumbens-area)(rh.fusiform_7)(rh.parahippocampal_3)(rh.superiortemporal_3)
0.00553	1.00E-05	0.98507	0.9375	(Right-Putamen)(rh.insula_2)(rh.precuneus_2)(rh.superiortemporal_3)
0.00553	1.00E-05	0.98507	0.9375	(rh.insula_2)(rh.isthmuscingulate_2)(rh.precuneus_2)(rh.superiortemporal_3)
0.00553	1.00E-05	0.98507	0.9375	(Right-Thalamus-Proper)(rh.insula_2)(rh.precuneus_2)(rh.superiortemporal_3)
0.00553	1.00E-05	0.98507	0.9375	(rh.insula_2)(rh.precuneus_2)(rh.superiortemporal_3)
0.00555	1.00E-05	0.83582	0.73148	(rh.fusiform_7)(rh.inferiorparietal_4)(rh.inferiorparietal_9)
0.00564	1.00E-05	0.84701	0.74519	(rh.fusiform_7)(rh.precuneus_2)(rh.superiortemporal_10)(rh.supramarginal_9)
0.00564	1.00E-05	0.84701	0.74519	(Right-Pallidum)(rh.fusiform_7)(rh.precuneus_4)(rh.supramarginal_9)

0.00564	1.00E-05	0.84701	0.74519	(rh.fusiform_7)(rh.parahippocampal_2)(rh.precuneus_2)(rh.superiortemporal_10)
0.00564	1.00E-05	0.84701	0.74519	(Right-Accumbens-area)(rh.bankssts_2)(rh.fusiform_7)(rh.parahippocampal_3)
0.00564	1.00E-05	0.84701	0.74519	(rh.bankssts_1)(rh.inferiorparietal_9)(rh.parahippocampal_2)(rh.precuneus_2)
0.00568	1.00E-05	0.88806	0.7963	(Right-Pallidum)(rh.inferiorparietal_9)(rh.supramarginal_9)
0.00568	1.00E-05	0.88806	0.7963	(Right-Caudate)(Right-Pallidum)(rh.inferiorparietal_9)(rh.precuneus_2)
0.00568	1.00E-05	0.88806	0.7963	(rh.inferiorparietal_4)(rh.inferiorparietal_9)(rh.insula_2)(rh.precuneus_2)
0.00568	1.00E-05	0.88806	0.7963	(Right-Pallidum)(rh.inferiorparietal_10)(rh.inferiorparietal_9)
0.00568	1.00E-05	0.88806	0.7963	(rh.inferiorparietal_4)(rh.inferiorparietal_9)(rh.superiortemporal_3)
0.00568	1.00E-05	0.88806	0.7963	(rh.inferiorparietal_4)(rh.inferiorparietal_9)(rh.lingual_7)(rh.precuneus_2)
0.00573	1.00E-05	0.80224	0.69231	(Right-Accumbens-area)(Right-Putamen)(rh.inferiorparietal_9)(rh.precuneus_2)
0.00573	1.00E-05	0.80224	0.69231	(Right-Accumbens-area)(Right-Thalamus-Proper)(rh.inferiorparietal_9)(rh.precuneus_2)
0.00573	1.00E-05	0.80224	0.69231	(rh.inferiorparietal_9)(rh.insula_5)(rh.precuneus_4)(rh.supramarginal_9)
0.00573	1.00E-05	0.80224	0.69231	(Right-Accumbens-area)(Right-Pallidum)(rh.inferiorparietal_9)(rh.precuneus_2)
0.00573	1.00E-05	0.80224	0.69231	(Right-Accumbens-area)(rh.inferiorparietal_9)(rh.precuneus_2)
0.00573	1.00E-05	0.80224	0.69231	(Right-Accumbens-area)(rh.inferiorparietal_9)(rh.insula_2)(rh.precuneus_2)
0.00573	1.00E-05	0.80224	0.69231	(Right-Accumbens-area)(rh.inferiorparietal_9)(rh.isthmuscingulate_2)(rh.precuneus_2)
0.00576	1.00E-05	0.85075	0.75	(rh.inferiorparietal_9)(rh.parahippocampal_2)(rh.parahippocampal_3)(rh.superiortempor
0.00576	1.00E-05	0.85075	0.75	(Right-Putamen)(rh.fusiform_7)(rh.parahippocampal_3)(rh.superiortemporal_10)
0.00576	1.00E-05	0.85075	0.75	(rh.fusiform_7)(rh.isthmuscingulate_2)(rh.parahippocampal_2)(rh.precuneus_4)
0.00576	1.00E-05	0.85075	0.75	(rh.fusiform_7)(rh.insula_5)(rh.parahippocampal_3)(rh.superiortemporal_10)
0.00576	1.00E-05	0.85075	0.75	(rh.fusiform_7)(rh.isthmuscingulate_2)(rh.parahippocampal_3)(rh.superiortemporal_10)
0.00576	1.00E-05	0.85075	0.75	(Right-Accumbens-area)(rh.bankssts_2)(rh.fusiform_7)(rh.superiortemporal_3)
0.00576	1.00E-05	0.85075	0.75	(rh.fusiform_7)(rh.parahippocampal_3)(rh.superiortemporal_10)
0.00576	1.00E-05	0.85075	0.75	(Right-Thalamus-Proper)(rh.fusiform_7)(rh.parahippocampal_3)(rh.superiortemporal_10)
0.00576	1.00E-05	0.85075	0.75	(Right-Thalamus-Proper)(rh.fusiform_7)(rh.parahippocampal_2)(rh.precuneus_4)
0.00576	1.00E-05	0.85075	0.75	(rh.inferiorparietal_9)(rh.insula_5)(rh.parahippocampal_3)(rh.superiortemporal_3)
0.00576	1.00E-05	0.85075	0.75	(Right-Putamen)(rh.fusiform_7)(rh.parahippocampal_2)(rh.precuneus_4)
0.00576	1.00E-05	0.85075	0.75	(rh.fusiform_7)(rh.insula_2)(rh.parahippocampal_2)(rh.precuneus_4)
0.00576	1.00E-05	0.85075	0.75	(Right-Pallidum)(rh.fusiform_7)(rh.parahippocampal_3)(rh.superiortemporal_10)
0.00576	1.00E-05	0.85075	0.75	(Right-Accumbens-area)(rh.fusiform_7)(rh.lingual_7)(rh.superiortemporal_3)
0.00576	1.00E-05	0.85075	0.75	(rh.fusiform_7)(rh.insula_2)(rh.parahippocampal_3)(rh.superiortemporal_10)
0.00578	1.00E-05	0.8806	0.78704	(rh.inferiorparietal_9)(rh.superiortemporal_1)(rh.supramarginal_9)
0.00582	1.00E-05	0.85821	0.75926	(rh.bankssts_1)(rh.inferiorparietal_9)
0.00588	1.00E-05	0.85448	0.75481	(rh.bankssts_2)(rh.inferiorparietal_9)(rh.parahippocampal_2)(rh.parahippocampal_3)
0.00588	1.00E-05	0.85448	0.75481	(rh.bankssts_1)(rh.bankssts_2)(rh.inferiorparietal_9)(rh.superiortemporal_3)

0.00588	1.00E-05	0.85448	0.75481	(rh.inferiorparietal_9)(rh.insula_5)(rh.parahippocampal_2)(rh.supramarginal_9)
0.00588	1.00E-05	0.85448	0.75481	(Right-Pallidum)(rh.fusiform_7)(rh.precuneus_4)(rh.superiortemporal_3)
0.00588	1.00E-05	0.85448	0.75481	(rh.bankssts_2)(rh.inferiorparietal_9)(rh.insula_5)(rh.parahippocampal_3)
0.00588	1.00E-05	0.85448	0.75481	(rh.inferiorparietal_9)(rh.insula_5)(rh.lingual_7)(rh.parahippocampal_3)
0.00588	1.00E-05	0.85448	0.75481	(rh.inferiorparietal_9)(rh.lingual_7)(rh.parahippocampal_2)(rh.parahippocampal_3)
0.00588	1.00E-05	0.85448	0.75481	(Right-Caudate)(rh.fusiform_7)(rh.insula_2)(rh.precuneus_4)
0.00588	1.00E-05	0.85448	0.75481	(Right-Caudate)(rh.fusiform_7)(rh.isthmuscingulate_2)(rh.precuneus_4)
0.00588	1.00E-05	0.85448	0.75481	(Right-Caudate)(Right-Putamen)(rh.fusiform_7)(rh.precuneus_4)
0.00588	1.00E-05	0.85448	0.75481	(Right-Caudate)(Right-Thalamus-Proper)(rh.fusiform_7)(rh.precuneus_4)
0.00588	1.00E-05	0.85448	0.75481	(Right-Caudate)(rh.fusiform_7)(rh.precuneus_4)
0.00588	1.00E-05	0.85448	0.75481	(Right-Accumbens-area)(rh.bankssts_2)(rh.fusiform_7)(rh.lingual_7)
0.00592	1.00E-05	0.80597	0.69712	(rh.inferiorparietal_9)(rh.precuneus_2)(rh.precuneus_4)(rh.superiortemporal_9)
0.00599	1.00E-05	0.85821	0.75962	(Right-Thalamus-Proper)(rh.inferiorparietal_9)(rh.parahippocampal_3)(rh.superiortempor[illegible]
0.00599	1.00E-05	0.85821	0.75962	(Right-Thalamus-Proper)(rh.fusiform_7)(rh.precuneus_2)(rh.superiortemporal_10)
0.00599	1.00E-05	0.85821	0.75962	(rh.fusiform_7)(rh.precuneus_2)(rh.superiortemporal_6)(rh.supramarginal_9)
0.00599	1.00E-05	0.85821	0.75962	(rh.fusiform_7)(rh.insula_5)(rh.precuneus_2)(rh.superiortemporal_10)
0.00599	1.00E-05	0.85821	0.75962	(Right-Pallidum)(rh.bankssts_2)(rh.fusiform_7)(rh.precuneus_4)
0.00599	1.00E-05	0.85821	0.75962	(Right-Putamen)(rh.inferiorparietal_9)(rh.parahippocampal_3)(rh.superiortemporal_3)
0.00599	1.00E-05	0.85821	0.75962	(rh.fusiform_7)(rh.insula_2)(rh.precuneus_2)(rh.superiortemporal_10)
0.00599	1.00E-05	0.85821	0.75962	(Right-Pallidum)(rh.fusiform_7)(rh.lingual_7)(rh.precuneus_4)
0.00599	1.00E-05	0.85821	0.75962	(rh.fusiform_7)(rh.isthmuscingulate_2)(rh.precuneus_2)(rh.superiortemporal_10)
0.00599	1.00E-05	0.85821	0.75962	(rh.fusiform_7)(rh.fusiform_8)(rh.superiortemporal_3)(rh.superiortemporal_6)
0.00599	1.00E-05	0.85821	0.75962	(Right-Pallidum)(rh.inferiorparietal_9)(rh.parahippocampal_3)(rh.superiortemporal_3)
0.00599	1.00E-05	0.85821	0.75962	(rh.fusiform_7)(rh.precuneus_2)(rh.superiortemporal_10)
0.00599	1.00E-05	0.85821	0.75962	(Right-Caudate)(rh.fusiform_7)(rh.precuneus_2)(rh.precuneus_7)
0.00599	1.00E-05	0.85821	0.75962	(rh.inferiorparietal_4)(rh.inferiorparietal_9)(rh.insula_5)(rh.precuneus_2)
0.00599	1.00E-05	0.85821	0.75962	(rh.inferiorparietal_9)(rh.isthmuscingulate_2)(rh.parahippocampal_3)(rh.superiortempora[illegible]
0.00599	1.00E-05	0.85821	0.75962	(Right-Pallidum)(rh.fusiform_7)(rh.precuneus_2)(rh.superiortemporal_10)
0.00599	1.00E-05	0.85821	0.75962	(Right-Putamen)(rh.fusiform_7)(rh.precuneus_2)(rh.superiortemporal_10)
0.00599	1.00E-05	0.85821	0.75962	(rh.inferiorparietal_9)(rh.parahippocampal_3)(rh.superiortemporal_3)
0.00599	1.00E-05	0.85821	0.75962	(rh.inferiorparietal_9)(rh.insula_2)(rh.parahippocampal_3)(rh.superiortemporal_3)
0.00599	1.00E-05	0.85821	0.75962	(rh.fusiform_7)(rh.inferiortemporal_2)(rh.precuneus_2)(rh.superiortemporal_3)
0.00599	1.00E-05	0.85821	0.75962	(rh.fusiform_7)(rh.precuneus_2)(rh.precuneus_7)(rh.superiortemporal_3)
0.00599	1.00E-05	0.92537	0.84615	(Right-Putamen)(rh.fusiform_7)(rh.isthmuscingulate_2)(rh.lingual_7)
0.00599	1.00E-05	0.92537	0.84615	(Right-Putamen)(rh.fusiform_7)(rh.insula_2)(rh.lingual_7)

0.00599	1.00E-05	0.92537	0.84615	(rh.fusiform_7)(rh.lingual_7)
0.00599	1.00E-05	0.92537	0.84615	(rh.bankssts_2)(rh.fusiform_7)(rh.insula_2)
0.00599	1.00E-05	0.92537	0.84615	(Right-Thalamus-Proper)(rh.fusiform_7)(rh.insula_2)(rh.lingual_7)
0.00599	1.00E-05	0.92537	0.84615	(rh.fusiform_7)(rh.insula_2)(rh.lingual_7)
0.00599	1.00E-05	0.92537	0.84615	(Right-Putamen)(Right-Thalamus-Proper)(rh.bankssts_2)(rh.fusiform_7)
0.00599	1.00E-05	0.92537	0.84615	(Right-Thalamus-Proper)(rh.bankssts_2)(rh.fusiform_7)(rh.isthmuscingulate_2)
0.00599	1.00E-05	0.92537	0.84615	(Right-Thalamus-Proper)(rh.fusiform_7)(rh.isthmuscingulate_2)(rh.lingual_7)
0.00599	1.00E-05	0.92537	0.84615	(Right-Thalamus-Proper)(rh.bankssts_2)(rh.fusiform_7)(rh.insula_2)
0.00599	1.00E-05	0.92537	0.84615	(rh.bankssts_2)(rh.fusiform_7)(rh.insula_2)(rh.isthmuscingulate_2)
0.00599	1.00E-05	0.92537	0.84615	(Right-Putamen)(rh.bankssts_2)(rh.fusiform_7)(rh.insula_2)
0.00599	1.00E-05	0.92537	0.84615	(Right-Putamen)(rh.fusiform_7)(rh.lingual_7)
0.00599	1.00E-05	0.92537	0.84615	(Right-Thalamus-Proper)(rh.fusiform_7)(rh.lingual_7)
0.00599	1.00E-05	0.92537	0.84615	(rh.fusiform_7)(rh.insula_2)(rh.isthmuscingulate_2)(rh.lingual_7)
0.00599	1.00E-05	0.92537	0.84615	(Right-Putamen)(Right-Thalamus-Proper)(rh.fusiform_7)(rh.lingual_7)
0.00599	1.00E-05	0.92537	0.84615	(Right-Putamen)(rh.bankssts_2)(rh.fusiform_7)(rh.isthmuscingulate_2)
0.00599	1.00E-05	0.92537	0.84615	(Right-Thalamus-Proper)(rh.bankssts_2)(rh.fusiform_7)
0.00599	1.00E-05	0.92537	0.84615	(rh.bankssts_2)(rh.fusiform_7)
0.00599	1.00E-05	0.92537	0.84615	(Right-Putamen)(rh.bankssts_2)(rh.fusiform_7)
0.00599	1.00E-05	0.92537	0.84615	(rh.fusiform_7)(rh.isthmuscingulate_2)(rh.lingual_7)
0.00599	1.00E-05	0.92537	0.84615	(rh.bankssts_2)(rh.fusiform_7)(rh.isthmuscingulate_2)
0.00609	1.00E-05	0.86194	0.76442	(Right-Thalamus-Proper)(rh.fusiform_7)(rh.isthmuscingulate_2)(rh.precuneus_4)
0.00609	1.00E-05	0.86194	0.76442	(Right-Putamen)(rh.fusiform_7)(rh.precuneus_4)
0.00609	1.00E-05	0.86194	0.76442	(Right-Thalamus-Proper)(rh.fusiform_7)(rh.precuneus_4)
0.00609	1.00E-05	0.86194	0.76442	(Right-Thalamus-Proper)(rh.bankssts_2)(rh.inferiorparietal_9)(rh.parahippocampal_3)
0.00609	1.00E-05	0.86194	0.76442	(rh.fusiform_7)(rh.lingual_7)(rh.parahippocampal_3)(rh.superiortemporal_6)
0.00609	1.00E-05	0.86194	0.76442	(rh.bankssts_2)(rh.fusiform_7)(rh.precuneus_2)(rh.precuneus_7)
0.00609	1.00E-05	0.86194	0.76442	(rh.inferiorparietal_9)(rh.isthmuscingulate_2)(rh.lingual_7)(rh.parahippocampal_3)
0.00609	1.00E-05	0.86194	0.76442	(rh.inferiorparietal_9)(rh.lingual_7)(rh.parahippocampal_3)
0.00609	1.00E-05	0.86194	0.76442	(rh.inferiorparietal_9)(rh.insula_4)(rh.parahippocampal_2)(rh.precuneus_2)
0.00609	1.00E-05	0.86194	0.76442	(Right-Pallidum)(rh.bankssts_2)(rh.inferiorparietal_9)(rh.parahippocampal_3)
0.00609	1.00E-05	0.86194	0.76442	(Right-Putamen)(Right-Thalamus-Proper)(rh.fusiform_7)(rh.precuneus_4)
0.00609	1.00E-05	0.86194	0.76442	(Right-Putamen)(rh.fusiform_7)(rh.isthmuscingulate_2)(rh.precuneus_4)
0.00609	1.00E-05	0.86194	0.76442	(Right-Thalamus-Proper)(rh.inferiorparietal_9)(rh.lingual_7)(rh.parahippocampal_3)
0.00609	1.00E-05	0.86194	0.76442	(rh.fusiform_7)(rh.isthmuscingulate_2)(rh.precuneus_4)
0.00609	1.00E-05	0.86194	0.76442	(Right-Caudate)(rh.inferiorparietal_9)(rh.insula_5)(rh.parahippocampal_2)

0.00609	1.00E-05	0.86194	0.76442	(Right-Thalamus-Proper)(rh.fusiform_7)(rh.insula_2)(rh.precuneus_4)
0.00609	1.00E-05	0.86194	0.76442	(rh.bankssts_2)(rh.fusiform_7)(rh.parahippocampal_3)(rh.superiortemporal_6)
0.00609	1.00E-05	0.86194	0.76442	(Right-Pallidum)(rh.inferiorparietal_9)(rh.lingual_7)(rh.parahippocampal_3)
0.00609	1.00E-05	0.86194	0.76442	(rh.bankssts_2)(rh.inferiorparietal_9)(rh.isthmuscingulate_2)(rh.parahippocampal_3)
0.00609	1.00E-05	0.86194	0.76442	(rh.inferiorparietal_9)(rh.insula_2)(rh.lingual_7)(rh.parahippocampal_3)
0.00609	1.00E-05	0.86194	0.76442	(rh.fusiform_7)(rh.insula_2)(rh.precuneus_4)
0.00609	1.00E-05	0.86194	0.76442	(rh.fusiform_7)(rh.lingual_7)(rh.precuneus_2)(rh.precuneus_7)
0.00609	1.00E-05	0.86194	0.76442	(Right-Putamen)(rh.inferiorparietal_9)(rh.lingual_7)(rh.parahippocampal_3)
0.00609	1.00E-05	0.86194	0.76442	(Right-Putamen)(rh.fusiform_7)(rh.insula_2)(rh.precuneus_4)
0.00609	1.00E-05	0.86194	0.76442	(rh.bankssts_2)(rh.inferiorparietal_9)(rh.insula_2)(rh.parahippocampal_3)
0.00609	1.00E-05	0.86194	0.76442	(rh.fusiform_7)(rh.inferiortemporal_2)(rh.lingual_7)(rh.precuneus_2)
0.00609	1.00E-05	0.86194	0.76442	(rh.fusiform_7)(rh.precuneus_4)
0.00609	1.00E-05	0.86194	0.76442	(Right-Putamen)(rh.bankssts_2)(rh.inferiorparietal_9)(rh.parahippocampal_3)
0.00609	1.00E-05	0.86194	0.76442	(rh.fusiform_7)(rh.insula_2)(rh.isthmuscingulate_2)(rh.precuneus_4)
0.00609	1.00E-05	0.86194	0.76442	(rh.bankssts_2)(rh.fusiform_7)(rh.inferiortemporal_2)(rh.precuneus_2)
0.00609	1.00E-05	0.86194	0.76442	(rh.bankssts_2)(rh.inferiorparietal_9)(rh.parahippocampal_3)
0.00609	1.00E-05	0.86194	0.76442	(rh.fusiform_7)(rh.parahippocampal_2)(rh.precuneus_2)(rh.superiortemporal_6)
0.00612	1.00E-05	0.8097	0.70192	(Right-Accumbens-area)(Right-Caudate)(rh.fusiform_7)(rh.superiortemporal_10)
0.00612	1.00E-05	0.8097	0.70192	(Right-Pallidum)(rh.inferiorparietal_9)(rh.precuneus_2)(rh.superiortemporal_10)
0.00612	1.00E-05	0.8097	0.70192	(rh.inferiorparietal_9)(rh.parahippocampal_3)(rh.precuneus_2)(rh.superiortemporal_6)
0.00612	1.00E-05	0.8097	0.70192	(Right-Thalamus-Proper)(rh.inferiorparietal_9)(rh.precuneus_2)(rh.superiortemporal_10)
0.00612	1.00E-05	0.8097	0.70192	(rh.inferiorparietal_9)(rh.insula_2)(rh.precuneus_2)(rh.superiortemporal_10)
0.00612	1.00E-05	0.8097	0.70192	(rh.inferiorparietal_9)(rh.isthmuscingulate_2)(rh.precuneus_2)(rh.superiortemporal_10)
0.00612	1.00E-05	0.8097	0.70192	(rh.entorhinal_1)(rh.fusiform_7)(rh.precuneus_4)(rh.supramarginal_9)
0.00612	1.00E-05	0.8097	0.70192	(Right-Accumbens-area)(rh.fusiform_7)(rh.superiortemporal_6)(rh.supramarginal_9)
0.00612	1.00E-05	0.8097	0.70192	(rh.inferiorparietal_9)(rh.insula_5)(rh.precuneus_2)(rh.superiortemporal_10)
0.00612	1.00E-05	0.8097	0.70192	(rh.fusiform_7)(rh.fusiform_8)(rh.inferiorparietal_10)(rh.inferiorparietal_9)
0.00612	1.00E-05	0.8097	0.70192	(rh.inferiorparietal_9)(rh.insula_5)(rh.precuneus_4)(rh.superiortemporal_3)
0.00612	1.00E-05	0.8097	0.70192	(rh.inferiorparietal_9)(rh.precuneus_2)(rh.superiortemporal_10)
0.00612	1.00E-05	0.8097	0.70192	(Right-Putamen)(rh.inferiorparietal_9)(rh.precuneus_2)(rh.superiortemporal_10)
0.00615	1.00E-05	0.92164	0.84135	(Right-Putamen)(rh.fusiform_7)(rh.insula_2)(rh.superiortemporal_3)
0.00615	1.00E-05	0.92164	0.84135	(rh.fusiform_7)(rh.insula_2)(rh.isthmuscingulate_2)(rh.superiortemporal_3)
0.00615	1.00E-05	0.92164	0.84135	(Right-Putamen)(Right-Thalamus-Proper)(rh.fusiform_7)(rh.superiortemporal_3)
0.00615	1.00E-05	0.92164	0.84135	(rh.fusiform_7)(rh.isthmuscingulate_2)(rh.superiortemporal_3)
0.00615	1.00E-05	0.92164	0.84135	(rh.fusiform_7)(rh.superiortemporal_3)

0.00615	1.00E-05	0.92164	0.84135	(Right-Thalamus-Proper)(rh.fusiform_7)(rh.insula_2)(rh.superiortemporal_3)
0.00615	1.00E-05	0.92164	0.84135	(Right-Pallidum)(rh.bankssts_2)(rh.fusiform_7)(rh.lingual_7)
0.00615	1.00E-05	0.92164	0.84135	(rh.fusiform_7)(rh.insula_2)(rh.superiortemporal_3)
0.00615	1.00E-05	0.92164	0.84135	(Right-Putamen)(rh.fusiform_7)(rh.isthmuscingulate_2)(rh.superiortemporal_3)
0.00615	1.00E-05	0.92164	0.84135	(Right-Thalamus-Proper)(rh.fusiform_7)(rh.superiortemporal_3)
0.00615	1.00E-05	0.92164	0.84135	(Right-Thalamus-Proper)(rh.fusiform_7)(rh.isthmuscingulate_2)(rh.superiortemporal_3)
0.00615	1.00E-05	0.92164	0.84135	(Right-Putamen)(rh.fusiform_7)(rh.superiortemporal_3)
0.00619	1.00E-05	0.86567	0.76923	(rh.inferiorparietal_9)(rh.precuneus_2)(rh.superiortemporal_3)(rh.superiortemporal_9)
0.00627	1.00E-05	0.91791	0.83654	(Right-Pallidum)(rh.fusiform_7)(rh.lingual_7)(rh.superiortemporal_3)
0.00627	1.00E-05	0.91791	0.83654	(Right-Pallidum)(rh.bankssts_2)(rh.fusiform_7)(rh.superiortemporal_3)
0.00628	1.00E-05	0.8694	0.77404	(Right-Thalamus-Proper)(rh.inferiorparietal_9)(rh.insula_5)(rh.parahippocampal_2)
0.00628	1.00E-05	0.8694	0.77404	(rh.inferiorparietal_9)(rh.insula_5)(rh.parahippocampal_2)
0.00628	1.00E-05	0.8694	0.77404	(rh.fusiform_7)(rh.isthmuscingulate_2)(rh.precuneus_2)(rh.superiortemporal_6)
0.00628	1.00E-05	0.8694	0.77404	(rh.inferiorparietal_9)(rh.lingual_7)(rh.precuneus_2)(rh.superiortemporal_9)
0.00628	1.00E-05	0.8694	0.77404	(Right-Caudate)(rh.entorhinal_1)(rh.fusiform_7)(rh.precuneus_2)
0.00628	1.00E-05	0.8694	0.77404	(rh.fusiform_7)(rh.insula_2)(rh.precuneus_2)(rh.superiortemporal_6)
0.00628	1.00E-05	0.8694	0.77404	(rh.bankssts_2)(rh.inferiorparietal_9)(rh.precuneus_2)(rh.superiortemporal_9)
0.00628	1.00E-05	0.8694	0.77404	(Right-Thalamus-Proper)(rh.fusiform_7)(rh.precuneus_2)(rh.superiortemporal_6)
0.00628	1.00E-05	0.8694	0.77404	(rh.fusiform_7)(rh.precuneus_2)(rh.superiortemporal_6)
0.00628	1.00E-05	0.8694	0.77404	(Right-Putamen)(rh.inferiorparietal_9)(rh.insula_5)(rh.parahippocampal_2)
0.00628	1.00E-05	0.8694	0.77404	(Right-Pallidum)(rh.fusiform_7)(rh.precuneus_2)(rh.superiortemporal_6)
0.00628	1.00E-05	0.8694	0.77404	(Right-Putamen)(rh.fusiform_7)(rh.precuneus_2)(rh.superiortemporal_6)
0.00628	1.00E-05	0.8694	0.77404	(Right-Pallidum)(rh.inferiorparietal_9)(rh.insula_5)(rh.parahippocampal_2)
0.00628	1.00E-05	0.8694	0.77404	(rh.entorhinal_1)(rh.fusiform_7)(rh.insula_4)(rh.precuneus_2)
0.00628	1.00E-05	0.8694	0.77404	(rh.inferiorparietal_9)(rh.insula_2)(rh.insula_5)(rh.parahippocampal_2)
0.00628	1.00E-05	0.8694	0.77404	(rh.inferiorparietal_9)(rh.insula_5)(rh.isthmuscingulate_2)(rh.parahippocampal_2)
0.00632	1.00E-05	0.81343	0.70673	(Right-Accumbens-area)(Right-Putamen)(rh.fusiform_7)(rh.superiortemporal_10)
0.00632	1.00E-05	0.81343	0.70673	(Right-Accumbens-area)(Right-Pallidum)(rh.fusiform_7)(rh.superiortemporal_10)
0.00632	1.00E-05	0.81343	0.70673	(rh.bankssts_2)(rh.inferiorparietal_9)(rh.insula_5)(rh.precuneus_4)
0.00632	1.00E-05	0.81343	0.70673	(Right-Putamen)(rh.inferiorparietal_9)(rh.inferiortemporal_2)(rh.precuneus_2)
0.00632	1.00E-05	0.81343	0.70673	(Right-Thalamus-Proper)(rh.inferiorparietal_9)(rh.inferiortemporal_2)(rh.precuneus_2)
0.00632	1.00E-05	0.81343	0.70673	(Right-Accumbens-area)(Right-Thalamus-Proper)(rh.fusiform_7)(rh.superiortemporal_10)
0.00632	1.00E-05	0.81343	0.70673	(rh.entorhinal_1)(rh.inferiorparietal_4)(rh.inferiorparietal_9)(rh.precuneus_2)
0.00632	1.00E-05	0.81343	0.70673	(rh.inferiorparietal_9)(rh.inferiortemporal_2)(rh.isthmuscingulate_2)(rh.precuneus_2)
0.00632	1.00E-05	0.81343	0.70673	(Right-Accumbens-area)(rh.fusiform_7)(rh.insula_5)(rh.superiortemporal_10)

0.00632	1.00E-05	0.81343	0.70673	(rh.inferiorparietal_9)(rh.inferiortemporal_2)(rh.insula_2)(rh.precuneus_2)
0.00632	1.00E-05	0.81343	0.70673	(Right-Pallidum)(rh.inferiorparietal_9)(rh.inferiortemporal_2)(rh.precuneus_2)
0.00632	1.00E-05	0.81343	0.70673	(rh.inferiorparietal_9)(rh.insula_5)(rh.lingual_7)(rh.precuneus_4)
0.00632	1.00E-05	0.81343	0.70673	(rh.inferiorparietal_9)(rh.inferiortemporal_2)(rh.precuneus_2)
0.00632	1.00E-05	0.81343	0.70673	(Right-Accumbens-area)(rh.fusiform_7)(rh.insula_2)(rh.superiortemporal_10)
0.00632	1.00E-05	0.81343	0.70673	(Right-Accumbens-area)(rh.fusiform_7)(rh.isthmuscingulate_2)(rh.superiortemporal_10)
0.00636	1.00E-05	0.87313	0.77885	(rh.bankssts_2)(rh.inferiorparietal_9)(rh.insula_5)(rh.superiortemporal_3)
0.00636	1.00E-05	0.87313	0.77885	(rh.inferiorparietal_9)(rh.insula_5)(rh.lingual_7)(rh.superiortemporal_3)
0.00636	1.00E-05	0.87313	0.77885	(rh.fusiform_7)(rh.fusiform_8)(rh.parahippocampal_3)(rh.supramarginal_9)
0.00636	1.00E-05	0.87313	0.77885	(rh.fusiform_7)(rh.parahippocampal_3)(rh.precuneus_2)(rh.superiortemporal_1)
0.00638	1.00E-05	0.91418	0.83173	(Right-Thalamus-Proper)(rh.bankssts_2)(rh.fusiform_7)(rh.parahippocampal_2)
0.00638	1.00E-05	0.91418	0.83173	(Right-Putamen)(rh.bankssts_2)(rh.fusiform_7)(rh.parahippocampal_2)
0.00638	1.00E-05	0.91418	0.83173	(Right-Putamen)(rh.fusiform_7)(rh.lingual_7)(rh.parahippocampal_2)
0.00638	1.00E-05	0.91418	0.83173	(rh.bankssts_2)(rh.fusiform_7)(rh.parahippocampal_2)
0.00638	1.00E-05	0.91418	0.83173	(Right-Thalamus-Proper)(rh.fusiform_7)(rh.lingual_7)(rh.parahippocampal_2)
0.00638	1.00E-05	0.91418	0.83173	(rh.fusiform_7)(rh.isthmuscingulate_2)(rh.lingual_7)(rh.parahippocampal_2)
0.00638	1.00E-05	0.91418	0.83173	(rh.fusiform_7)(rh.insula_2)(rh.lingual_7)(rh.parahippocampal_2)
0.00638	1.00E-05	0.91418	0.83173	(rh.bankssts_2)(rh.fusiform_7)(rh.insula_2)(rh.parahippocampal_2)
0.00638	1.00E-05	0.91418	0.83173	(rh.bankssts_2)(rh.fusiform_7)(rh.isthmuscingulate_2)(rh.parahippocampal_2)
0.00638	1.00E-05	0.91418	0.83173	(Right-Caudate)(rh.bankssts_2)(rh.fusiform_7)(rh.lingual_7)
0.00638	1.00E-05	0.91418	0.83173	(rh.fusiform_7)(rh.lingual_7)(rh.parahippocampal_2)
0.00643	1.00E-05	0.87687	0.78365	(rh.entorhinal_1)(rh.fusiform_7)(rh.isthmuscingulate_2)(rh.parahippocampal_3)
0.00643	1.00E-05	0.87687	0.78365	(Right-Thalamus-Proper)(rh.entorhinal_1)(rh.fusiform_7)(rh.parahippocampal_3)
0.00643	1.00E-05	0.87687	0.78365	(Right-Putamen)(rh.entorhinal_1)(rh.fusiform_7)(rh.parahippocampal_3)
0.00643	1.00E-05	0.87687	0.78365	(Right-Caudate)(rh.fusiform_7)(rh.fusiform_8)(rh.parahippocampal_3)
0.00643	1.00E-05	0.87687	0.78365	(rh.fusiform_7)(rh.fusiform_8)(rh.precuneus_2)(rh.supramarginal_9)
0.00643	1.00E-05	0.87687	0.78365	(rh.entorhinal_1)(rh.fusiform_7)(rh.parahippocampal_3)(rh.superiortemporal_9)
0.00643	1.00E-05	0.87687	0.78365	(Right-Pallidum)(rh.entorhinal_1)(rh.fusiform_7)(rh.parahippocampal_3)
0.00643	1.00E-05	0.87687	0.78365	(rh.inferiorparietal_9)(rh.superiortemporal_1)(rh.superiortemporal_3)(rh.supramarginal_[illegible]
0.00643	1.00E-05	0.87687	0.78365	(rh.entorhinal_1)(rh.fusiform_7)(rh.parahippocampal_3)
0.00643	1.00E-05	0.87687	0.78365	(rh.entorhinal_1)(rh.fusiform_7)(rh.insula_2)(rh.parahippocampal_3)
0.00643	1.00E-05	0.87687	0.78365	(rh.bankssts_2)(rh.inferiorparietal_9)(rh.insula_5)(rh.lingual_7)
0.00646	1.00E-05	0.91045	0.82692	(Right-Putamen)(rh.bankssts_2)(rh.fusiform_7)(rh.supramarginal_9)
0.00646	1.00E-05	0.91045	0.82692	(rh.fusiform_7)(rh.isthmuscingulate_2)(rh.precuneus_2)(rh.superiortemporal_9)
0.00646	1.00E-05	0.91045	0.82692	(Right-Putamen)(rh.fusiform_7)(rh.lingual_7)(rh.supramarginal_9)

0.00646	1.00E-05	0.91045	0.82692	(rh.inferiorparietal_9)(rh.insula_2)(rh.lingual_7)
0.00646	1.00E-05	0.91045	0.82692	(Right-Thalamus-Proper)(rh.fusiform_7)(rh.precuneus_2)(rh.superiortemporal_9)
0.00646	1.00E-05	0.91045	0.82692	(rh.fusiform_7)(rh.insula_2)(rh.parahippocampal_2)(rh.superiortemporal_3)
0.00646	1.00E-05	0.91045	0.82692	(Right-Thalamus-Proper)(rh.inferiorparietal_9)(rh.insula_2)(rh.lingual_7)
0.00646	1.00E-05	0.91045	0.82692	(Right-Putamen)(rh.fusiform_7)(rh.precuneus_2)(rh.superiortemporal_9)
0.00646	1.00E-05	0.91045	0.82692	(Right-Thalamus-Proper)(rh.fusiform_7)(rh.parahippocampal_2)(rh.superiortemporal_3)
0.00646	1.00E-05	0.91045	0.82692	(rh.bankssts_2)(rh.inferiorparietal_9)(rh.insula_2)
0.00646	1.00E-05	0.91045	0.82692	(Right-Thalamus-Proper)(rh.bankssts_2)(rh.inferiorparietal_9)(rh.insula_2)
0.00646	1.00E-05	0.91045	0.82692	(rh.fusiform_7)(rh.isthmuscingulate_2)(rh.parahippocampal_2)(rh.superiortemporal_3)
0.00646	1.00E-05	0.91045	0.82692	(Right-Thalamus-Proper)(rh.bankssts_2)(rh.fusiform_7)(rh.supramarginal_9)
0.00646	1.00E-05	0.91045	0.82692	(rh.bankssts_2)(rh.fusiform_7)(rh.insula_2)(rh.supramarginal_9)
0.00646	1.00E-05	0.91045	0.82692	(rh.inferiorparietal_9)(rh.insula_2)(rh.isthmuscingulate_2)(rh.lingual_7)
0.00646	1.00E-05	0.91045	0.82692	(rh.fusiform_7)(rh.insula_2)(rh.lingual_7)(rh.supramarginal_9)
0.00646	1.00E-05	0.91045	0.82692	(Right-Caudate)(rh.bankssts_2)(rh.fusiform_7)(rh.superiortemporal_3)
0.00646	1.00E-05	0.91045	0.82692	(rh.fusiform_7)(rh.insula_2)(rh.precuneus_2)(rh.superiortemporal_9)
0.00646	1.00E-05	0.91045	0.82692	(Right-Caudate)(rh.fusiform_7)(rh.lingual_7)(rh.superiortemporal_3)
0.00646	1.00E-05	0.91045	0.82692	(rh.bankssts_2)(rh.fusiform_7)(rh.isthmuscingulate_2)(rh.supramarginal_9)
0.00646	1.00E-05	0.91045	0.82692	(Right-Putamen)(rh.inferiorparietal_9)(rh.insula_2)(rh.lingual_7)
0.00646	1.00E-05	0.91045	0.82692	(Right-Putamen)(rh.fusiform_7)(rh.parahippocampal_2)(rh.superiortemporal_3)
0.00646	1.00E-05	0.91045	0.82692	(rh.bankssts_2)(rh.inferiorparietal_9)(rh.insula_2)(rh.isthmuscingulate_2)
0.00646	1.00E-05	0.91045	0.82692	(rh.fusiform_7)(rh.parahippocampal_2)(rh.superiortemporal_3)
0.00646	1.00E-05	0.91045	0.82692	(Right-Pallidum)(rh.fusiform_7)(rh.precuneus_2)(rh.superiortemporal_9)
0.00646	1.00E-05	0.91045	0.82692	(rh.fusiform_7)(rh.isthmuscingulate_2)(rh.lingual_7)(rh.supramarginal_9)
0.00646	1.00E-05	0.91045	0.82692	(rh.fusiform_7)(rh.lingual_7)(rh.supramarginal_9)
0.00646	1.00E-05	0.91045	0.82692	(Right-Thalamus-Proper)(rh.fusiform_7)(rh.lingual_7)(rh.supramarginal_9)
0.00646	1.00E-05	0.91045	0.82692	(Right-Putamen)(rh.bankssts_2)(rh.inferiorparietal_9)(rh.insula_2)
0.00646	1.00E-05	0.91045	0.82692	(rh.fusiform_7)(rh.precuneus_2)(rh.superiortemporal_9)
0.00646	1.00E-05	0.91045	0.82692	(rh.bankssts_2)(rh.fusiform_7)(rh.supramarginal_9)
0.00649	1.00E-05	0.8806	0.78846	(rh.fusiform_7)(rh.fusiform_8)(rh.parahippocampal_2)(rh.precuneus_2)
0.00649	1.00E-05	0.8806	0.78846	(rh.bankssts_2)(rh.entorhinal_1)(rh.fusiform_7)(rh.superiortemporal_3)
0.00649	1.00E-05	0.8806	0.78846	(Right-Caudate)(rh.fusiform_7)(rh.fusiform_8)(rh.precuneus_2)
0.00649	1.00E-05	0.8806	0.78846	(rh.inferiorparietal_4)(rh.inferiorparietal_9)(rh.parahippocampal_2)(rh.superiortemporal_
0.00649	1.00E-05	0.8806	0.78846	(rh.entorhinal_1)(rh.fusiform_7)(rh.lingual_7)(rh.superiortemporal_3)
0.00652	1.00E-05	0.81716	0.71154	(rh.fusiform_7)(rh.inferiorparietal_9)(rh.parahippocampal_2)(rh.superiortemporal_1)
0.00652	1.00E-05	0.81716	0.71154	(rh.fusiform_7)(rh.inferiorparietal_9)(rh.insula_5)(rh.precuneus_3)

0.00652	1.00E-05	0.81716	0.71154	(Right-Accumbens-area)(Right-Caudate)(rh.fusiform_7)(rh.superiortemporal_6)
0.00652	1.00E-05	0.81716	0.71154	(rh.fusiform_7)(rh.fusiform_8)(rh.isthmuscingulate_1)(rh.precuneus_2)
0.00652	1.00E-05	0.81716	0.71154	(rh.entorhinal_1)(rh.fusiform_7)(rh.precuneus_4)(rh.superiortemporal_3)
0.00652	1.00E-05	0.81716	0.71154	(Right-Caudate)(rh.fusiform_7)(rh.inferiorparietal_9)(rh.superiortemporal_1)
0.00652	1.00E-05	0.81716	0.71154	(rh.entorhinal_1)(rh.inferiorparietal_9)(rh.superiortemporal_3)(rh.supramarginal_9)
0.00653	1.00E-05	0.90672	0.82212	(rh.bankssts_2)(rh.inferiorparietal_9)(rh.isthmuscingulate_2)(rh.parahippocampal_2)
0.00653	1.00E-05	0.90672	0.82212	(Right-Thalamus-Proper)(rh.bankssts_2)(rh.inferiorparietal_9)(rh.parahippocampal_2)
0.00653	1.00E-05	0.90672	0.82212	(Right-Caudate)(rh.fusiform_7)(rh.lingual_7)(rh.parahippocampal_2)
0.00653	1.00E-05	0.90672	0.82212	(rh.inferiorparietal_9)(rh.isthmuscingulate_2)(rh.lingual_7)(rh.parahippocampal_2)
0.00653	1.00E-05	0.90672	0.82212	(rh.fusiform_7)(rh.isthmuscingulate_2)(rh.superiortemporal_3)(rh.supramarginal_9)
0.00653	1.00E-05	0.90672	0.82212	(rh.fusiform_7)(rh.superiortemporal_3)(rh.supramarginal_9)
0.00653	1.00E-05	0.90672	0.82212	(Right-Thalamus-Proper)(rh.fusiform_7)(rh.superiortemporal_3)(rh.supramarginal_9)
0.00653	1.00E-05	0.90672	0.82212	(Right-Thalamus-Proper)(rh.inferiorparietal_9)(rh.lingual_7)(rh.parahippocampal_2)
0.00653	1.00E-05	0.90672	0.82212	(rh.bankssts_2)(rh.inferiorparietal_9)(rh.parahippocampal_2)
0.00653	1.00E-05	0.90672	0.82212	(Right-Putamen)(rh.fusiform_7)(rh.superiortemporal_3)(rh.supramarginal_9)
0.00653	1.00E-05	0.90672	0.82212	(Right-Putamen)(rh.bankssts_2)(rh.inferiorparietal_9)(rh.parahippocampal_2)
0.00653	1.00E-05	0.90672	0.82212	(rh.fusiform_7)(rh.insula_2)(rh.superiortemporal_3)(rh.supramarginal_9)
0.00653	1.00E-05	0.90672	0.82212	(Right-Caudate)(rh.bankssts_2)(rh.fusiform_7)(rh.parahippocampal_2)
0.00653	1.00E-05	0.90672	0.82212	(Right-Putamen)(rh.inferiorparietal_9)(rh.lingual_7)(rh.parahippocampal_2)
0.00654	1.00E-05	0.88433	0.79327	(rh.fusiform_7)(rh.fusiform_8)(rh.parahippocampal_3)(rh.superiortemporal_9)
0.00654	1.00E-05	0.88433	0.79327	(Right-Caudate)(rh.fusiform_7)(rh.parahippocampal_3)(rh.supramarginal_9)
0.00654	2.00E-05	0.88433	0.79327	(rh.fusiform_7)(rh.fusiform_8)(rh.insula_5)(rh.parahippocampal_3)
0.00654	2.00E-05	0.88433	0.79327	(rh.inferiorparietal_10)(rh.inferiorparietal_9)(rh.precuneus_2)(rh.supramarginal_9)
0.00654	2.00E-05	0.88433	0.79327	(rh.bankssts_2)(rh.entorhinal_1)(rh.fusiform_7)(rh.lingual_7)
0.00657	2.00E-05	0.90299	0.81731	(rh.fusiform_7)(rh.inferiorparietal_10)(rh.isthmuscingulate_2)(rh.precuneus_2)
0.00657	2.00E-05	0.90299	0.81731	(rh.bankssts_2)(rh.fusiform_7)(rh.insula_4)(rh.precuneus_2)
0.00657	2.00E-05	0.90299	0.81731	(Right-Putamen)(rh.fusiform_7)(rh.inferiorparietal_10)(rh.precuneus_2)
0.00657	2.00E-05	0.90299	0.81731	(rh.fusiform_7)(rh.inferiorparietal_10)(rh.insula_2)(rh.precuneus_2)
0.00657	2.00E-05	0.90299	0.81731	(rh.fusiform_7)(rh.inferiorparietal_10)(rh.precuneus_2)
0.00657	2.00E-05	0.90299	0.81731	(rh.fusiform_7)(rh.insula_4)(rh.insula_5)(rh.precuneus_2)
0.00657	2.00E-05	0.90299	0.81731	(Right-Thalamus-Proper)(rh.fusiform_7)(rh.inferiorparietal_10)(rh.precuneus_2)
0.00657	2.00E-05	0.90299	0.81731	(Right-Thalamus-Proper)(rh.fusiform_7)(rh.inferiorparietal_4)(rh.precuneus_2)
0.00657	2.00E-05	0.90299	0.81731	(rh.fusiform_7)(rh.inferiorparietal_4)(rh.precuneus_2)
0.00657	2.00E-05	0.90299	0.81731	(Right-Caudate)(rh.fusiform_7)(rh.lingual_7)(rh.supramarginal_9)
0.00657	2.00E-05	0.90299	0.81731	(rh.fusiform_7)(rh.inferiorparietal_4)(rh.insula_2)(rh.precuneus_2)

0.00657	2.00E-05	0.90299	0.81731	(Right-Putamen)(rh.fusiform_7)(rh.inferiorparietal_4)(rh.precuneus_2)
0.00657	2.00E-05	0.90299	0.81731	(rh.fusiform_7)(rh.inferiorparietal_4)(rh.isthmuscingulate_2)(rh.precuneus_2)
0.00657	2.00E-05	0.90299	0.81731	(Right-Caudate)(rh.fusiform_7)(rh.parahippocampal_2)(rh.superiortemporal_3)
0.00657	2.00E-05	0.90299	0.81731	(Right-Caudate)(rh.bankssts_2)(rh.fusiform_7)(rh.supramarginal_9)
0.00657	2.00E-05	0.90299	0.81731	(rh.fusiform_7)(rh.insula_4)(rh.lingual_7)(rh.precuneus_2)
0.00657	2.00E-05	0.88806	0.79808	(rh.fusiform_7)(rh.fusiform_8)(rh.insula_5)(rh.precuneus_2)
0.00657	2.00E-05	0.88806	0.79808	(Right-Caudate)(rh.inferiorparietal_9)(rh.parahippocampal_2)(rh.supramarginal_9)
0.00657	2.00E-05	0.88806	0.79808	(Right-Caudate)(rh.inferiorparietal_9)(rh.precuneus_2)(rh.supramarginal_9)
0.00657	2.00E-05	0.88806	0.79808	(rh.fusiform_7)(rh.inferiorparietal_4)(rh.parahippocampal_3)(rh.superiortemporal_3)
0.00657	2.00E-05	0.88806	0.79808	(rh.fusiform_7)(rh.precuneus_2)(rh.superiortemporal_1)(rh.supramarginal_9)
0.00657	2.00E-05	0.88806	0.79808	(rh.fusiform_7)(rh.fusiform_8)(rh.precuneus_2)(rh.superiortemporal_9)
0.00657	2.00E-05	0.88806	0.79808	(Right-Caudate)(rh.inferiorparietal_10)(rh.inferiorparietal_9)(rh.precuneus_2)
0.0066	2.00E-05	0.89925	0.8125	(Right-Caudate)(rh.inferiorparietal_9)(rh.lingual_7)(rh.parahippocampal_2)
0.0066	2.00E-05	0.89925	0.8125	(rh.fusiform_7)(rh.lingual_7)(rh.parahippocampal_3)(rh.superiortemporal_9)
0.0066	2.00E-05	0.89925	0.8125	(Right-Caudate)(Right-Thalamus-Proper)(rh.inferiorparietal_9)(rh.precuneus_2)
0.0066	2.00E-05	0.89925	0.8125	(rh.fusiform_7)(rh.lingual_7)(rh.parahippocampal_2)(rh.supramarginal_9)
0.0066	2.00E-05	0.89925	0.8125	(Right-Pallidum)(rh.bankssts_2)(rh.inferiorparietal_9)(rh.precuneus_2)
0.0066	2.00E-05	0.89925	0.8125	(rh.fusiform_7)(rh.parahippocampal_2)(rh.precuneus_2)(rh.superiortemporal_9)
0.0066	2.00E-05	0.89925	0.8125	(Right-Caudate)(rh.inferiorparietal_9)(rh.isthmuscingulate_2)(rh.precuneus_2)
0.0066	2.00E-05	0.89925	0.8125	(Right-Pallidum)(rh.inferiorparietal_9)(rh.lingual_7)(rh.superiortemporal_3)
0.0066	2.00E-05	0.89925	0.8125	(rh.fusiform_7)(rh.insula_5)(rh.parahippocampal_3)
0.0066	2.00E-05	0.89925	0.8125	(rh.bankssts_2)(rh.fusiform_7)(rh.parahippocampal_3)(rh.superiortemporal_9)
0.0066	2.00E-05	0.89925	0.8125	(Right-Caudate)(Right-Putamen)(rh.inferiorparietal_9)(rh.precuneus_2)
0.0066	2.00E-05	0.89925	0.8125	(Right-Pallidum)(rh.inferiorparietal_9)(rh.insula_2)(rh.superiortemporal_3)
0.0066	2.00E-05	0.89925	0.8125	(rh.bankssts_2)(rh.fusiform_7)(rh.parahippocampal_2)(rh.supramarginal_9)
0.0066	2.00E-05	0.89925	0.8125	(Right-Putamen)(rh.fusiform_7)(rh.insula_5)(rh.parahippocampal_3)
0.0066	2.00E-05	0.89925	0.8125	(Right-Thalamus-Proper)(rh.fusiform_7)(rh.insula_5)(rh.parahippocampal_3)
0.0066	2.00E-05	0.89925	0.8125	(Right-Pallidum)(rh.fusiform_7)(rh.insula_5)(rh.parahippocampal_3)
0.0066	2.00E-05	0.89925	0.8125	(rh.fusiform_7)(rh.insula_2)(rh.insula_5)(rh.parahippocampal_3)
0.0066	2.00E-05	0.89925	0.8125	(Right-Caudate)(rh.inferiorparietal_9)(rh.precuneus_2)
0.0066	2.00E-05	0.89925	0.8125	(Right-Caudate)(rh.fusiform_7)(rh.superiortemporal_3)(rh.supramarginal_9)
0.0066	2.00E-05	0.89925	0.8125	(Right-Caudate)(rh.bankssts_2)(rh.inferiorparietal_9)(rh.parahippocampal_2)
0.0066	2.00E-05	0.89925	0.8125	(Right-Caudate)(rh.fusiform_7)(rh.insula_5)(rh.precuneus_2)
0.0066	2.00E-05	0.89925	0.8125	(Right-Pallidum)(rh.bankssts_2)(rh.inferiorparietal_9)(rh.superiortemporal_3)
0.0066	2.00E-05	0.89925	0.8125	(rh.fusiform_7)(rh.insula_4)(rh.precuneus_2)(rh.superiortemporal_3)

0.0066	2.00E-05	0.89925	0.8125	(rh.fusiform_7)(rh.insula_5)(rh.isthmuscingulate_2)(rh.parahippocampal_3)
0.0066	2.00E-05	0.89179	0.80288	(rh.fusiform_7)(rh.inferiorparietal_4)(rh.lingual_7)(rh.parahippocampal_3)
0.0066	2.00E-05	0.89179	0.80288	(rh.fusiform_7)(rh.parahippocampal_2)(rh.parahippocampal_3)(rh.superiortemporal_3)
0.0066	2.00E-05	0.89179	0.80288	(Right-Pallidum)(rh.fusiform_7)(rh.parahippocampal_3)(rh.supramarginal_9)
0.0066	2.00E-05	0.89179	0.80288	(rh.inferiorparietal_9)(rh.lingual_7)(rh.parahippocampal_2)(rh.supramarginal_9)
0.0066	2.00E-05	0.89179	0.80288	(rh.fusiform_7)(rh.inferiorparietal_4)(rh.parahippocampal_2)(rh.precuneus_2)
0.0066	2.00E-05	0.89179	0.80288	(Right-Putamen)(rh.fusiform_7)(rh.parahippocampal_3)(rh.supramarginal_9)
0.0066	2.00E-05	0.89179	0.80288	(rh.fusiform_7)(rh.inferiorparietal_10)(rh.precuneus_2)(rh.supramarginal_9)
0.0066	2.00E-05	0.89179	0.80288	(rh.bankssts_2)(rh.inferiorparietal_9)(rh.parahippocampal_2)(rh.supramarginal_9)
0.0066	2.00E-05	0.89179	0.80288	(Right-Thalamus-Proper)(rh.fusiform_7)(rh.parahippocampal_3)(rh.supramarginal_9)
0.0066	2.00E-05	0.89179	0.80288	(rh.bankssts_2)(rh.fusiform_7)(rh.inferiorparietal_4)(rh.parahippocampal_3)
0.0066	2.00E-05	0.89179	0.80288	(rh.fusiform_7)(rh.precuneus_2)(rh.precuneus_3)(rh.superiortemporal_3)
0.0066	2.00E-05	0.89179	0.80288	(rh.fusiform_7)(rh.insula_2)(rh.parahippocampal_3)(rh.supramarginal_9)
0.0066	2.00E-05	0.89179	0.80288	(rh.fusiform_7)(rh.isthmuscingulate_2)(rh.parahippocampal_3)(rh.supramarginal_9)
0.0066	2.00E-05	0.89179	0.80288	(rh.fusiform_7)(rh.parahippocampal_3)(rh.supramarginal_9)
0.0066	2.00E-05	0.89179	0.80288	(rh.fusiform_7)(rh.inferiorparietal_10)(rh.parahippocampal_2)(rh.precuneus_2)
0.0066	2.00E-05	0.89552	0.80769	(Right-Caudate)(rh.fusiform_7)(rh.parahippocampal_3)
0.0066	2.00E-05	0.89552	0.80769	(Right-Caudate)(rh.fusiform_7)(rh.inferiorparietal_10)(rh.precuneus_2)
0.0066	2.00E-05	0.89552	0.80769	(rh.fusiform_7)(rh.lingual_7)(rh.precuneus_2)(rh.precuneus_3)
0.0066	2.00E-05	0.89552	0.80769	(Right-Caudate)(Right-Thalamus-Proper)(rh.fusiform_7)(rh.parahippocampal_3)
0.0066	2.00E-05	0.89552	0.80769	(rh.bankssts_2)(rh.inferiorparietal_9)(rh.insula_2)(rh.supramarginal_9)
0.0066	2.00E-05	0.89552	0.80769	(Right-Caudate)(rh.fusiform_7)(rh.parahippocampal_2)(rh.supramarginal_9)
0.0066	2.00E-05	0.89552	0.80769	(rh.inferiorparietal_9)(rh.isthmuscingulate_2)(rh.precuneus_2)(rh.supramarginal_9)
0.0066	2.00E-05	0.89552	0.80769	(rh.fusiform_7)(rh.insula_5)(rh.precuneus_2)(rh.supramarginal_9)
0.0066	2.00E-05	0.89552	0.80769	(rh.inferiorparietal_10)(rh.inferiorparietal_9)(rh.isthmuscingulate_2)(rh.precuneus_2)
0.0066	2.00E-05	0.89552	0.80769	(Right-Putamen)(rh.inferiorparietal_9)(rh.precuneus_2)(rh.supramarginal_9)
0.0066	2.00E-05	0.89552	0.80769	(Right-Caudate)(rh.fusiform_7)(rh.insula_2)(rh.parahippocampal_3)
0.0066	2.00E-05	0.89552	0.80769	(Right-Caudate)(Right-Pallidum)(rh.fusiform_7)(rh.parahippocampal_3)
0.0066	2.00E-05	0.89552	0.80769	(Right-Caudate)(rh.fusiform_7)(rh.isthmuscingulate_2)(rh.parahippocampal_3)
0.0066	2.00E-05	0.89552	0.80769	(rh.bankssts_2)(rh.fusiform_7)(rh.parahippocampal_2)(rh.parahippocampal_3)
0.0066	2.00E-05	0.89552	0.80769	(rh.fusiform_7)(rh.parahippocampal_3)(rh.superiortemporal_3)(rh.superiortemporal_9)
0.0066	2.00E-05	0.89552	0.80769	(Right-Caudate)(Right-Putamen)(rh.fusiform_7)(rh.parahippocampal_3)
0.0066	2.00E-05	0.89552	0.80769	(rh.inferiorparietal_9)(rh.insula_2)(rh.lingual_7)(rh.supramarginal_9)
0.0066	2.00E-05	0.89552	0.80769	(Right-Putamen)(rh.inferiorparietal_10)(rh.inferiorparietal_9)(rh.precuneus_2)
0.0066	2.00E-05	0.89552	0.80769	(rh.bankssts_2)(rh.fusiform_7)(rh.precuneus_2)(rh.precuneus_3)

0.0066	2.00E-05	0.89552	0.80769	(rh.fusiform_7)(rh.lingual_7)(rh.parahippocampal_2)(rh.parahippocampal_3)
0.0066	2.00E-05	0.89552	0.80769	(Right-Thalamus-Proper)(rh.inferiorparietal_10)(rh.inferiorparietal_9)(rh.precuneus_2)
0.0066	2.00E-05	0.89552	0.80769	(rh.fusiform_7)(rh.parahippocampal_2)(rh.superiortemporal_3)(rh.supramarginal_9)
0.0066	2.00E-05	0.89552	0.80769	(Right-Thalamus-Proper)(rh.inferiorparietal_9)(rh.precuneus_2)(rh.supramarginal_9)
0.00672	2.00E-05	0.8209	0.71635	(Right-Accumbens-area)(Right-Thalamus-Proper)(rh.fusiform_7)(rh.superiortemporal_6)
0.00672	2.00E-05	0.8209	0.71635	(Right-Accumbens-area)(rh.fusiform_7)(rh.insula_2)(rh.superiortemporal_6)
0.00672	2.00E-05	0.8209	0.71635	(Right-Accumbens-area)(Right-Pallidum)(rh.fusiform_7)(rh.superiortemporal_6)
0.00672	2.00E-05	0.8209	0.71635	(rh.fusiform_7)(rh.fusiform_8)(rh.precuneus_4)(rh.supramarginal_9)
0.00672	2.00E-05	0.8209	0.71635	(rh.entorhinal_1)(rh.inferiorparietal_9)(rh.lingual_7)(rh.supramarginal_9)
0.00672	2.00E-05	0.8209	0.71635	(rh.fusiform_7)(rh.inferiorparietal_9)(rh.precuneus_3)(rh.superiortemporal_9)
0.00672	2.00E-05	0.8209	0.71635	(rh.bankssts_2)(rh.entorhinal_1)(rh.fusiform_7)(rh.precuneus_4)
0.00672	2.00E-05	0.8209	0.71635	(Right-Accumbens-area)(Right-Putamen)(rh.fusiform_7)(rh.superiortemporal_6)
0.00672	2.00E-05	0.8209	0.71635	(rh.bankssts_2)(rh.entorhinal_1)(rh.inferiorparietal_9)(rh.supramarginal_9)
0.00672	2.00E-05	0.8209	0.71635	(rh.entorhinal_1)(rh.inferiorparietal_9)(rh.parahippocampal_2)(rh.parahippocampal_3)
0.00672	2.00E-05	0.8209	0.71635	(Right-Accumbens-area)(rh.fusiform_7)(rh.isthmuscingulate_2)(rh.superiortemporal_6)
0.00692	2.00E-05	0.82463	0.72115	(rh.fusiform_7)(rh.inferiorparietal_9)(rh.superiortemporal_1)
0.00692	2.00E-05	0.82463	0.72115	(rh.inferiorparietal_9)(rh.precuneus_2)(rh.superiortemporal_3)(rh.superiortemporal_6)
0.00692	2.00E-05	0.82463	0.72115	(rh.fusiform_7)(rh.inferiorparietal_9)(rh.isthmuscingulate_2)(rh.superiortemporal_1)
0.00692	2.00E-05	0.82463	0.72115	(Right-Thalamus-Proper)(rh.fusiform_7)(rh.precuneus_4)(rh.precuneus_7)
0.00692	2.00E-05	0.82463	0.72115	(rh.fusiform_7)(rh.isthmuscingulate_2)(rh.precuneus_4)(rh.precuneus_7)
0.00692	2.00E-05	0.82463	0.72115	(Right-Putamen)(rh.fusiform_7)(rh.precuneus_4)(rh.precuneus_7)
0.00692	2.00E-05	0.82463	0.72115	(rh.fusiform_7)(rh.insula_2)(rh.precuneus_4)(rh.precuneus_7)
0.00692	2.00E-05	0.82463	0.72115	(rh.entorhinal_1)(rh.inferiorparietal_9)(rh.parahippocampal_2)(rh.superiortemporal_3)
0.00692	2.00E-05	0.82463	0.72115	(rh.fusiform_7)(rh.inferiorparietal_9)(rh.insula_2)(rh.superiortemporal_1)
0.00692	2.00E-05	0.82463	0.72115	(Right-Putamen)(rh.fusiform_7)(rh.inferiorparietal_9)(rh.superiortemporal_1)
0.00692	2.00E-05	0.82463	0.72115	(Right-Thalamus-Proper)(rh.fusiform_7)(rh.inferiorparietal_9)(rh.superiortemporal_1)
0.00712	2.00E-05	0.82836	0.72596	(Right-Putamen)(rh.entorhinal_1)(rh.inferiorparietal_9)(rh.parahippocampal_3)
0.00712	2.00E-05	0.82836	0.72596	(rh.inferiorparietal_9)(rh.isthmuscingulate_1)(rh.superiortemporal_3)(rh.supramarginal_[illegible]
0.00712	2.00E-05	0.82836	0.72596	(rh.entorhinal_1)(rh.inferiorparietal_9)(rh.parahippocampal_3)(rh.superiortemporal_9)
0.00712	2.00E-05	0.82836	0.72596	(rh.fusiform_7)(rh.isthmuscingulate_1)(rh.precuneus_2)(rh.superiortemporal_1)
0.00712	2.00E-05	0.82836	0.72596	(rh.entorhinal_1)(rh.inferiorparietal_9)(rh.parahippocampal_3)
0.00712	2.00E-05	0.82836	0.72596	(rh.fusiform_7)(rh.inferiorparietal_4)(rh.inferiorparietal_9)(rh.superiortemporal_9)
0.00712	2.00E-05	0.82836	0.72596	(rh.entorhinal_1)(rh.inferiorparietal_9)(rh.lingual_7)(rh.parahippocampal_2)
0.00712	2.00E-05	0.82836	0.72596	(rh.inferiorparietal_9)(rh.parahippocampal_2)(rh.precuneus_4)(rh.supramarginal_9)
0.00712	2.00E-05	0.82836	0.72596	(rh.fusiform_8)(rh.inferiorparietal_9)(rh.parahippocampal_2)(rh.parahippocampal_3)

0.00712	2.00E-05	0.82836	0.72596	(rh.entorhinal_1)(rh.inferiorparietal_9)(rh.isthmuscingulate_2)(rh.parahippocampal_3)
0.00712	2.00E-05	0.82836	0.72596	(rh.bankssts_2)(rh.entorhinal_1)(rh.inferiorparietal_9)(rh.parahippocampal_2)
0.00712	2.00E-05	0.82836	0.72596	(rh.inferiorparietal_9)(rh.isthmuscingulate_1)(rh.parahippocampal_2)(rh.superiortempora[illegible]
0.00712	2.00E-05	0.82836	0.72596	(rh.entorhinal_1)(rh.inferiorparietal_9)(rh.insula_2)(rh.parahippocampal_3)
0.00712	2.00E-05	0.82836	0.72596	(Right-Pallidum)(rh.entorhinal_1)(rh.inferiorparietal_9)(rh.parahippocampal_3)
0.00712	2.00E-05	0.82836	0.72596	(rh.fusiform_7)(rh.fusiform_8)(rh.precuneus_4)(rh.superiortemporal_3)
0.00712	2.00E-05	0.82836	0.72596	(Right-Thalamus-Proper)(rh.entorhinal_1)(rh.inferiorparietal_9)(rh.parahippocampal_3)
0.00716	2.00E-05	0.98134	0.93269	(rh.bankssts_2)(rh.lingual_7)(rh.parahippocampal_2)(rh.superiortemporal_3)
0.00732	2.00E-05	0.83209	0.73077	(Right-Thalamus-Proper)(rh.entorhinal_1)(rh.inferiorparietal_9)(rh.superiortemporal_3)
0.00732	2.00E-05	0.83209	0.73077	(rh.bankssts_2)(rh.fusiform_7)(rh.fusiform_8)(rh.precuneus_4)
0.00732	2.00E-05	0.83209	0.73077	(rh.entorhinal_1)(rh.inferiorparietal_9)(rh.insula_2)(rh.superiortemporal_3)
0.00732	2.00E-05	0.83209	0.73077	(rh.inferiorparietal_9)(rh.insula_2)(rh.isthmuscingulate_1)(rh.precuneus_2)
0.00732	2.00E-05	0.83209	0.73077	(rh.inferiorparietal_9)(rh.insula_2)(rh.precuneus_4)(rh.supramarginal_9)
0.00732	2.00E-05	0.83209	0.73077	(rh.entorhinal_1)(rh.inferiorparietal_9)(rh.superiortemporal_3)
0.00732	2.00E-05	0.83209	0.73077	(rh.entorhinal_1)(rh.inferiorparietal_9)(rh.superiortemporal_3)(rh.superiortemporal_9)
0.00732	2.00E-05	0.83209	0.73077	(Right-Putamen)(rh.entorhinal_1)(rh.inferiorparietal_9)(rh.superiortemporal_3)
0.00732	2.00E-05	0.83209	0.73077	(Right-Accumbens-area)(rh.entorhinal_1)(rh.fusiform_7)(rh.superiortemporal_3)
0.00732	2.00E-05	0.83209	0.73077	(Right-Pallidum)(rh.entorhinal_1)(rh.inferiorparietal_9)(rh.superiortemporal_3)
0.00732	2.00E-05	0.83209	0.73077	(rh.entorhinal_1)(rh.inferiorparietal_9)(rh.isthmuscingulate_2)(rh.superiortemporal_3)
0.00752	2.00E-05	0.83582	0.73558	(rh.fusiform_8)(rh.inferiorparietal_9)(rh.parahippocampal_3)
0.00752	2.00E-05	0.83582	0.73558	(Right-Putamen)(rh.entorhinal_1)(rh.inferiorparietal_9)(rh.lingual_7)
0.00752	2.00E-05	0.83582	0.73558	(Right-Pallidum)(rh.bankssts_2)(rh.entorhinal_1)(rh.inferiorparietal_9)
0.00752	2.00E-05	0.83582	0.73558	(rh.fusiform_8)(rh.inferiorparietal_9)(rh.isthmuscingulate_2)(rh.parahippocampal_3)
0.00752	2.00E-05	0.83582	0.73558	(Right-Thalamus-Proper)(rh.entorhinal_1)(rh.inferiorparietal_9)(rh.lingual_7)
0.00752	2.00E-05	0.83582	0.73558	(Right-Accumbens-area)(rh.fusiform_7)(rh.fusiform_8)(rh.parahippocampal_3)
0.00752	2.00E-05	0.83582	0.73558	(rh.fusiform_8)(rh.inferiorparietal_9)(rh.insula_2)(rh.parahippocampal_3)
0.00752	2.00E-05	0.83582	0.73558	(rh.fusiform_7)(rh.insula_5)(rh.isthmuscingulate_1)(rh.precuneus_2)
0.00752	2.00E-05	0.83582	0.73558	(rh.bankssts_2)(rh.entorhinal_1)(rh.inferiorparietal_9)
0.00752	2.00E-05	0.83582	0.73558	(rh.entorhinal_1)(rh.inferiorparietal_9)(rh.lingual_7)(rh.superiortemporal_9)
0.00752	2.00E-05	0.83582	0.73558	(Right-Accumbens-area)(rh.fusiform_7)(rh.parahippocampal_3)(rh.supramarginal_9)
0.00752	2.00E-05	0.83582	0.73558	(rh.inferiorparietal_9)(rh.isthmuscingulate_1)(rh.lingual_7)(rh.superiortemporal_3)
0.00752	2.00E-05	0.83582	0.73558	(Right-Pallidum)(rh.fusiform_8)(rh.inferiorparietal_9)(rh.parahippocampal_3)
0.00752	2.00E-05	0.83582	0.73558	(rh.bankssts_2)(rh.entorhinal_1)(rh.inferiorparietal_9)(rh.isthmuscingulate_2)
0.00752	2.00E-05	0.83582	0.73558	(rh.bankssts_2)(rh.entorhinal_1)(rh.inferiorparietal_9)(rh.insula_2)
0.00752	2.00E-05	0.83582	0.73558	(rh.bankssts_2)(rh.entorhinal_1)(rh.inferiorparietal_9)(rh.superiortemporal_9)

0.00752	2.00E-05	0.83582	0.73558	(Right-Pallidum)(rh.entorhinal_1)(rh.inferiorparietal_9)(rh.lingual_7)
0.00752	2.00E-05	0.83582	0.73558	(Right-Accumbens-area)(rh.bankssts_2)(rh.entorhinal_1)(rh.fusiform_7)
0.00752	2.00E-05	0.83582	0.73558	(Right-Thalamus-Proper)(rh.bankssts_2)(rh.entorhinal_1)(rh.inferiorparietal_9)
0.00752	2.00E-05	0.83582	0.73558	(Right-Putamen)(rh.fusiform_8)(rh.inferiorparietal_9)(rh.parahippocampal_3)
0.00752	2.00E-05	0.83582	0.73558	(rh.fusiform_8)(rh.inferiorparietal_9)(rh.parahippocampal_3)(rh.superiortemporal_9)
0.00752	2.00E-05	0.83582	0.73558	(rh.entorhinal_1)(rh.inferiorparietal_9)(rh.lingual_7)
0.00752	2.00E-05	0.83582	0.73558	(Right-Thalamus-Proper)(rh.fusiform_8)(rh.inferiorparietal_9)(rh.parahippocampal_3)
0.00752	2.00E-05	0.83582	0.73558	(rh.entorhinal_1)(rh.inferiorparietal_9)(rh.insula_2)(rh.lingual_7)
0.00752	2.00E-05	0.83582	0.73558	(Right-Caudate)(rh.inferiorparietal_9)(rh.parahippocampal_2)(rh.precuneus_4)
0.00752	2.00E-05	0.83582	0.73558	(rh.bankssts_2)(rh.inferiorparietal_9)(rh.isthmuscingulate_1)(rh.superiortemporal_3)
0.00752	2.00E-05	0.83582	0.73558	(rh.entorhinal_1)(rh.inferiorparietal_9)(rh.isthmuscingulate_2)(rh.lingual_7)
0.00752	2.00E-05	0.83582	0.73558	(rh.fusiform_7)(rh.precuneus_4)(rh.superiortemporal_1)(rh.supramarginal_9)
0.00752	2.00E-05	0.83582	0.73558	(Right-Putamen)(rh.bankssts_2)(rh.entorhinal_1)(rh.inferiorparietal_9)
0.00758	2.00E-05	0.98881	0.94712	(rh.bankssts_2)(rh.insula_2)(rh.lingual_7)(rh.precuneus_2)
0.00772	2.00E-05	0.83955	0.74038	(rh.fusiform_8)(rh.inferiorparietal_9)(rh.precuneus_2)(rh.superiortemporal_9)
0.00772	2.00E-05	0.83955	0.74038	(Right-Pallidum)(rh.fusiform_8)(rh.inferiorparietal_9)(rh.precuneus_2)
0.00772	2.00E-05	0.83955	0.74038	(rh.fusiform_7)(rh.insula_5)(rh.precuneus_4)(rh.supramarginal_9)
0.00772	2.00E-05	0.83955	0.74038	(Right-Putamen)(rh.fusiform_8)(rh.inferiorparietal_9)(rh.precuneus_2)
0.00772	2.00E-05	0.83955	0.74038	(rh.inferiorparietal_9)(rh.lingual_7)(rh.parahippocampal_2)(rh.precuneus_4)
0.00772	2.00E-05	0.83955	0.74038	(rh.fusiform_8)(rh.inferiorparietal_9)(rh.isthmuscingulate_2)(rh.precuneus_2)
0.00772	2.00E-05	0.83955	0.74038	(Right-Thalamus-Proper)(rh.fusiform_8)(rh.inferiorparietal_9)(rh.precuneus_2)
0.00772	2.00E-05	0.83955	0.74038	(Right-Accumbens-area)(rh.fusiform_7)(rh.superiortemporal_3)(rh.supramarginal_9)
0.00772	2.00E-05	0.83955	0.74038	(rh.bankssts_2)(rh.inferiorparietal_9)(rh.parahippocampal_2)(rh.precuneus_4)
0.00772	2.00E-05	0.83955	0.74038	(Right-Accumbens-area)(rh.fusiform_7)(rh.fusiform_8)(rh.superiortemporal_3)
0.00772	2.00E-05	0.83955	0.74038	(rh.fusiform_8)(rh.inferiorparietal_9)(rh.insula_2)(rh.precuneus_2)
0.00772	2.00E-05	0.83955	0.74038	(rh.fusiform_7)(rh.insula_4)(rh.precuneus_2)(rh.precuneus_4)
0.00772	2.00E-05	0.83955	0.74038	(rh.fusiform_7)(rh.isthmuscingulate_1)(rh.superiortemporal_3)(rh.supramarginal_9)
0.00772	2.00E-05	0.83955	0.74038	(rh.fusiform_8)(rh.inferiorparietal_9)(rh.precuneus_2)
0.00772	2.00E-05	0.83955	0.74038	(rh.fusiform_7)(rh.isthmuscingulate_1)(rh.precuneus_2)(rh.superiortemporal_9)
0.00791	2.00E-05	0.84328	0.74519	(Right-Accumbens-area)(Right-Caudate)(rh.fusiform_7)(rh.supramarginal_9)
0.00791	2.00E-05	0.84328	0.74519	(rh.inferiorparietal_9)(rh.insula_2)(rh.lingual_7)(rh.precuneus_4)
0.00791	2.00E-05	0.84328	0.74519	(Right-Accumbens-area)(Right-Caudate)(rh.fusiform_7)(rh.parahippocampal_3)
0.00791	2.00E-05	0.84328	0.74519	(Right-Accumbens-area)(rh.bankssts_2)(rh.fusiform_7)(rh.supramarginal_9)
0.00791	2.00E-05	0.84328	0.74519	(Right-Accumbens-area)(rh.bankssts_2)(rh.fusiform_7)(rh.fusiform_8)
0.00791	2.00E-05	0.84328	0.74519	(rh.inferiorparietal_9)(rh.parahippocampal_3)(rh.superiortemporal_3)(rh.supramarginal_[illegible]

0.00791	2.00E-05	0.84328	0.74519	(rh.bankssts_2)(rh.inferiorparietal_9)(rh.insula_2)(rh.precuneus_4)
0.00791	2.00E-05	0.84328	0.74519	(rh.fusiform_7)(rh.parahippocampal_3)(rh.precuneus_2)(rh.precuneus_7)
0.00791	2.00E-05	0.84328	0.74519	(rh.fusiform_7)(rh.inferiorparietal_10)(rh.precuneus_4)(rh.superiortemporal_3)
0.00791	2.00E-05	0.84328	0.74519	(rh.bankssts_2)(rh.fusiform_7)(rh.isthmuscingulate_1)(rh.supramarginal_9)
0.00805	2.00E-05	0.80224	0.69712	(rh.inferiorparietal_10)(rh.inferiorparietal_9)(rh.insula_5)(rh.precuneus_4)
0.0081	2.00E-05	0.84701	0.75	(rh.bankssts_2)(rh.fusiform_7)(rh.isthmuscingulate_1)(rh.superiortemporal_3)
0.0081	2.00E-05	0.84701	0.75	(rh.fusiform_7)(rh.isthmuscingulate_1)(rh.lingual_7)(rh.superiortemporal_3)
0.0081	2.00E-05	0.84701	0.75	(rh.fusiform_7)(rh.inferiortemporal_2)(rh.parahippocampal_3)(rh.superiortemporal_3)
0.0081	2.00E-05	0.84701	0.75	(rh.inferiorparietal_9)(rh.insula_4)(rh.insula_5)(rh.parahippocampal_3)
0.0081	2.00E-05	0.84701	0.75	(rh.inferiorparietal_4)(rh.inferiorparietal_9)(rh.parahippocampal_3)
0.0081	2.00E-05	0.84701	0.75	(Right-Accumbens-area)(Right-Caudate)(rh.fusiform_7)(rh.superiortemporal_3)
0.0081	2.00E-05	0.84701	0.75	(Right-Accumbens-area)(Right-Putamen)(rh.fusiform_7)(rh.parahippocampal_3)
0.0081	2.00E-05	0.84701	0.75	(rh.inferiorparietal_9)(rh.lingual_7)(rh.parahippocampal_3)(rh.supramarginal_9)
0.0081	2.00E-05	0.84701	0.75	(Right-Accumbens-area)(Right-Pallidum)(rh.fusiform_7)(rh.parahippocampal_3)
0.0081	2.00E-05	0.84701	0.75	(Right-Accumbens-area)(Right-Thalamus-Proper)(rh.fusiform_7)(rh.parahippocampal_3)
0.0081	2.00E-05	0.84701	0.75	(rh.bankssts_2)(rh.inferiorparietal_9)(rh.parahippocampal_3)(rh.supramarginal_9)
0.0081	2.00E-05	0.84701	0.75	(Right-Pallidum)(rh.fusiform_7)(rh.parahippocampal_2)(rh.precuneus_4)
0.0081	2.00E-05	0.84701	0.75	(rh.inferiorparietal_9)(rh.middletemporal_4)(rh.supramarginal_9)
0.0081	2.00E-05	0.84701	0.75	(Right-Accumbens-area)(rh.fusiform_7)(rh.insula_2)(rh.parahippocampal_3)
0.0081	2.00E-05	0.84701	0.75	(Right-Caudate)(rh.inferiorparietal_9)(rh.parahippocampal_3)(rh.superiortemporal_3)
0.0081	2.00E-05	0.84701	0.75	(rh.inferiorparietal_9)(rh.insula_5)(rh.parahippocampal_2)(rh.parahippocampal_3)
0.0081	2.00E-05	0.84701	0.75	(Right-Caudate)(rh.inferiorparietal_9)(rh.parahippocampal_2)(rh.parahippocampal_3)
0.0081	2.00E-05	0.84701	0.75	(Right-Accumbens-area)(rh.fusiform_7)(rh.isthmuscingulate_2)(rh.parahippocampal_3)
0.0081	2.00E-05	0.84701	0.75	(rh.fusiform_7)(rh.fusiform_8)(rh.superiortemporal_6)(rh.supramarginal_9)
0.0081	2.00E-05	0.84701	0.75	(rh.fusiform_7)(rh.insula_5)(rh.precuneus_4)(rh.superiortemporal_3)
0.0081	2.00E-05	0.84701	0.75	(rh.bankssts_2)(rh.fusiform_7)(rh.inferiorparietal_10)(rh.precuneus_4)
0.0081	2.00E-05	0.84701	0.75	(Right-Caudate)(rh.fusiform_7)(rh.precuneus_2)(rh.superiortemporal_10)
0.00828	2.00E-05	0.85075	0.75481	(rh.bankssts_2)(rh.fusiform_7)(rh.insula_5)(rh.precuneus_4)
0.00828	2.00E-05	0.85075	0.75481	(Right-Caudate)(Right-Pallidum)(rh.fusiform_7)(rh.precuneus_4)
0.00828	2.00E-05	0.85075	0.75481	(Right-Accumbens-area)(rh.fusiform_7)(rh.superiortemporal_3)
0.00828	2.00E-05	0.85075	0.75481	(Right-Accumbens-area)(Right-Thalamus-Proper)(rh.fusiform_7)(rh.superiortemporal_3)
0.00828	2.00E-05	0.85075	0.75481	(rh.fusiform_7)(rh.insula_5)(rh.lingual_7)(rh.precuneus_4)
0.00828	2.00E-05	0.85075	0.75481	(Right-Accumbens-area)(Right-Caudate)(rh.bankssts_2)(rh.fusiform_7)
0.00828	2.00E-05	0.85075	0.75481	(Right-Accumbens-area)(rh.fusiform_7)(rh.isthmuscingulate_2)(rh.superiortemporal_3)
0.00828	2.00E-05	0.85075	0.75481	(Right-Accumbens-area)(Right-Pallidum)(rh.fusiform_7)(rh.superiortemporal_3)

0.00828	2.00E-05	0.85075	0.75481	(Right-Accumbens-area)(Right-Putamen)(rh.fusiform_7)(rh.superiortemporal_3)
0.00828	2.00E-05	0.85075	0.75481	(Right-Caudate)(rh.bankssts_2)(rh.inferiorparietal_9)(rh.parahippocampal_3)
0.00828	2.00E-05	0.85075	0.75481	(rh.inferiorparietal_9)(rh.parahippocampal_3)(rh.superiortemporal_3)(rh.superiortempor
0.00828	2.00E-05	0.85075	0.75481	(Right-Caudate)(rh.inferiorparietal_9)(rh.lingual_7)(rh.parahippocampal_3)
0.00828	2.00E-05	0.85075	0.75481	(Right-Accumbens-area)(rh.fusiform_7)(rh.insula_2)(rh.superiortemporal_3)
0.00828	2.00E-05	0.85075	0.75481	(rh.bankssts_1)(rh.inferiorparietal_9)(rh.insula_2)(rh.superiortemporal_3)
0.00828	2.00E-05	0.85075	0.75481	(rh.fusiform_7)(rh.inferiortemporal_2)(rh.parahippocampal_2)(rh.precuneus_2)
0.00828	2.00E-05	0.85075	0.75481	(rh.fusiform_7)(rh.inferiortemporal_2)(rh.lingual_7)(rh.parahippocampal_3)
0.00828	2.00E-05	0.85075	0.75481	(rh.bankssts_2)(rh.fusiform_7)(rh.inferiortemporal_2)(rh.parahippocampal_3)
0.00828	2.00E-05	0.85075	0.75481	(rh.bankssts_2)(rh.fusiform_7)(rh.isthmuscingulate_1)(rh.lingual_7)
0.00828	2.00E-05	0.85075	0.75481	(rh.fusiform_7)(rh.parahippocampal_2)(rh.precuneus_2)(rh.precuneus_7)
0.00828	2.00E-05	0.85075	0.75481	(rh.fusiform_7)(rh.parahippocampal_3)(rh.superiortemporal_6)(rh.supramarginal_9)
0.00828	2.00E-05	0.85075	0.75481	(rh.fusiform_7)(rh.precuneus_2)(rh.precuneus_7)(rh.supramarginal_9)
0.00828	2.00E-05	0.85075	0.75481	(rh.fusiform_7)(rh.insula_4)(rh.precuneus_2)(rh.superiortemporal_10)
0.00828	2.00E-05	0.85075	0.75481	(rh.bankssts_1)(rh.inferiorparietal_9)(rh.insula_2)(rh.precuneus_2)
0.00833	2.00E-05	0.88433	0.7963	(rh.inferiorparietal_4)(rh.inferiorparietal_9)(rh.parahippocampal_2)
0.00833	2.00E-05	0.88433	0.7963	(rh.inferiorparietal_9)(rh.lingual_7)(rh.superiortemporal_1)
0.00833	2.00E-05	0.88433	0.7963	(Right-Pallidum)(rh.inferiorparietal_4)(rh.inferiorparietal_9)(rh.lingual_7)
0.00833	2.00E-05	0.80597	0.70192	(rh.fusiform_7)(rh.fusiform_8)(rh.inferiorparietal_4)(rh.inferiorparietal_9)
0.00834	2.00E-05	0.86194	0.76852	(rh.inferiorparietal_9)(rh.insula_5)(rh.supramarginal_9)
0.00834	2.00E-05	0.86194	0.76852	(rh.inferiorparietal_9)(rh.middletemporal_4)
0.00839	2.00E-05	0.87687	0.78704	(rh.inferiorparietal_9)(rh.insula_5)
0.00839	2.00E-05	0.87687	0.78704	(rh.inferiorparietal_9)(rh.parahippocampal_2)(rh.superiortemporal_1)
0.00846	2.00E-05	0.85448	0.75962	(rh.bankssts_2)(rh.inferiorparietal_9)(rh.parahippocampal_3)(rh.superiortemporal_9)
0.00846	2.00E-05	0.85448	0.75962	(Right-Accumbens-area)(rh.bankssts_2)(rh.fusiform_7)
0.00846	2.00E-05	0.85448	0.75962	(Right-Putamen)(rh.inferiorparietal_9)(rh.insula_5)(rh.parahippocampal_3)
0.00846	2.00E-05	0.85448	0.75962	(Right-Accumbens-area)(Right-Thalamus-Proper)(rh.bankssts_2)(rh.fusiform_7)
0.00846	2.00E-05	0.85448	0.75962	(rh.bankssts_1)(rh.inferiorparietal_9)(rh.lingual_7)(rh.superiortemporal_3)
0.00846	2.00E-05	0.85448	0.75962	(Right-Putamen)(rh.inferiorparietal_9)(rh.parahippocampal_2)(rh.parahippocampal_3)
0.00846	2.00E-05	0.85448	0.75962	(Right-Accumbens-area)(Right-Putamen)(rh.fusiform_7)(rh.lingual_7)
0.00846	2.00E-05	0.85448	0.75962	(rh.bankssts_1)(rh.fusiform_7)(rh.precuneus_2)(rh.superiortemporal_3)
0.00846	2.00E-05	0.85448	0.75962	(Right-Thalamus-Proper)(rh.inferiorparietal_9)(rh.parahippocampal_2)(rh.parahippocamp
0.00846	2.00E-05	0.85448	0.75962	(Right-Accumbens-area)(rh.bankssts_2)(rh.fusiform_7)(rh.insula_2)
0.00846	2.00E-05	0.85448	0.75962	(Right-Accumbens-area)(Right-Pallidum)(rh.fusiform_7)(rh.lingual_7)
0.00846	2.00E-05	0.85448	0.75962	(rh.inferiorparietal_9)(rh.middletemporal_4)(rh.precuneus_2)(rh.superiortemporal_3)

0.00846	3.00E-05	0.85448	0.75962	(Right-Accumbens-area)(rh.fusiform_7)(rh.insula_2)(rh.lingual_7)
0.00846	3.00E-05	0.85448	0.75962	(Right-Thalamus-Proper)(rh.bankssts_1)(rh.inferiorparietal_9)(rh.superiortemporal_3)
0.00846	3.00E-05	0.85448	0.75962	(rh.inferiorparietal_9)(rh.insula_5)(rh.parahippocampal_3)
0.00846	3.00E-05	0.85448	0.75962	(rh.inferiorparietal_9)(rh.insula_2)(rh.parahippocampal_2)(rh.parahippocampal_3)
0.00846	3.00E-05	0.85448	0.75962	(rh.inferiorparietal_9)(rh.lingual_7)(rh.parahippocampal_3)(rh.superiortemporal_9)
0.00846	3.00E-05	0.85448	0.75962	(rh.inferiorparietal_9)(rh.insula_5)(rh.isthmuscingulate_2)(rh.parahippocampal_3)
0.00846	3.00E-05	0.85448	0.75962	(Right-Putamen)(rh.bankssts_1)(rh.inferiorparietal_9)(rh.superiortemporal_3)
0.00846	3.00E-05	0.85448	0.75962	(Right-Accumbens-area)(rh.fusiform_7)(rh.lingual_7)
0.00846	3.00E-05	0.85448	0.75962	(Right-Accumbens-area)(rh.fusiform_7)(rh.isthmuscingulate_2)(rh.lingual_7)
0.00846	3.00E-05	0.85448	0.75962	(Right-Thalamus-Proper)(rh.inferiorparietal_9)(rh.insula_5)(rh.parahippocampal_3)
0.00846	3.00E-05	0.85448	0.75962	(Right-Pallidum)(rh.inferiorparietal_9)(rh.insula_5)(rh.parahippocampal_3)
0.00846	3.00E-05	0.85448	0.75962	(Right-Pallidum)(rh.inferiorparietal_9)(rh.parahippocampal_2)(rh.parahippocampal_3)
0.00846	3.00E-05	0.85448	0.75962	(Right-Accumbens-area)(Right-Putamen)(rh.bankssts_2)(rh.fusiform_7)
0.00846	3.00E-05	0.85448	0.75962	(Right-Accumbens-area)(Right-Thalamus-Proper)(rh.fusiform_7)(rh.lingual_7)
0.00846	3.00E-05	0.85448	0.75962	(Right-Accumbens-area)(Right-Pallidum)(rh.bankssts_2)(rh.fusiform_7)
0.00846	3.00E-05	0.85448	0.75962	(Right-Accumbens-area)(rh.bankssts_2)(rh.fusiform_7)(rh.isthmuscingulate_2)
0.00846	3.00E-05	0.85448	0.75962	(rh.inferiorparietal_9)(rh.isthmuscingulate_2)(rh.parahippocampal_2)(rh.parahippocampa
0.00846	3.00E-05	0.85448	0.75962	(rh.bankssts_1)(rh.bankssts_2)(rh.inferiorparietal_9)(rh.precuneus_2)
0.00846	3.00E-05	0.85448	0.75962	(rh.bankssts_1)(rh.inferiorparietal_9)(rh.superiortemporal_3)
0.00846	3.00E-05	0.85448	0.75962	(rh.inferiorparietal_9)(rh.insula_2)(rh.insula_5)(rh.parahippocampal_3)
0.00846	3.00E-05	0.85448	0.75962	(rh.inferiorparietal_9)(rh.parahippocampal_2)(rh.parahippocampal_3)
0.00846	3.00E-05	0.85448	0.75962	(rh.bankssts_1)(rh.inferiorparietal_9)(rh.isthmuscingulate_2)(rh.superiortemporal_3)
0.00861	3.00E-05	0.8097	0.70673	(rh.entorhinal_1)(rh.fusiform_7)(rh.fusiform_8)(rh.precuneus_4)
0.00861	3.00E-05	0.8097	0.70673	(rh.inferiorparietal_9)(rh.inferiortemporal_2)(rh.precuneus_2)(rh.superiortemporal_9)
0.00861	3.00E-05	0.8097	0.70673	(rh.inferiorparietal_9)(rh.inferiortemporal_2)(rh.insula_5)(rh.precuneus_2)
0.00864	3.00E-05	0.85821	0.76442	(rh.inferiorparietal_9)(rh.insula_5)(rh.superiortemporal_3)(rh.supramarginal_9)
0.00864	3.00E-05	0.85821	0.76442	(rh.bankssts_2)(rh.fusiform_7)(rh.fusiform_8)(rh.superiortemporal_6)
0.00864	3.00E-05	0.85821	0.76442	(rh.fusiform_7)(rh.fusiform_8)(rh.lingual_7)(rh.superiortemporal_6)
0.00864	3.00E-05	0.85821	0.76442	(Right-Pallidum)(rh.fusiform_7)(rh.isthmuscingulate_2)(rh.precuneus_4)
0.00864	3.00E-05	0.85821	0.76442	(Right-Pallidum)(Right-Thalamus-Proper)(rh.fusiform_7)(rh.precuneus_4)
0.00864	3.00E-05	0.85821	0.76442	(rh.bankssts_1)(rh.bankssts_2)(rh.fusiform_7)(rh.precuneus_2)
0.00864	3.00E-05	0.85821	0.76442	(rh.inferiorparietal_9)(rh.insula_4)(rh.insula_5)(rh.parahippocampal_2)
0.00864	3.00E-05	0.85821	0.76442	(Right-Pallidum)(rh.fusiform_7)(rh.precuneus_4)
0.00864	3.00E-05	0.85821	0.76442	(Right-Pallidum)(rh.fusiform_7)(rh.insula_2)(rh.precuneus_4)
0.00864	3.00E-05	0.85821	0.76442	(Right-Caudate)(rh.fusiform_7)(rh.precuneus_2)(rh.superiortemporal_6)

0.00864	3.00E-05	0.85821	0.76442	(Right-Pallidum)(Right-Putamen)(rh.fusiform_7)(rh.precuneus_4)
0.00878	3.00E-05	0.97761	0.92788	(Right-Caudate)(rh.precuneus_2)(rh.superiortemporal_3)
0.00878	3.00E-05	0.97761	0.92788	(Right-Caudate)(rh.isthmuscingulate_2)(rh.precuneus_2)(rh.superiortemporal_3)
0.00878	3.00E-05	0.97761	0.92788	(rh.insula_2)(rh.lingual_7)(rh.parahippocampal_2)(rh.precuneus_2)
0.00878	3.00E-05	0.97761	0.92788	(Right-Caudate)(Right-Thalamus-Proper)(rh.precuneus_2)(rh.superiortemporal_3)
0.00878	3.00E-05	0.97761	0.92788	(Right-Caudate)(Right-Putamen)(rh.precuneus_2)(rh.superiortemporal_3)
0.00878	3.00E-05	0.97761	0.92788	(rh.bankssts_2)(rh.insula_2)(rh.parahippocampal_2)(rh.precuneus_2)
0.00879	3.00E-05	0.96269	0.90385	(rh.parahippocampal_2)(rh.precuneus_2)(rh.superiortemporal_3)(rh.supramarginal_9)
0.0088	3.00E-05	0.86194	0.76923	(rh.fusiform_7)(rh.parahippocampal_3)(rh.superiortemporal_6)
0.0088	3.00E-05	0.86194	0.76923	(Right-Pallidum)(rh.inferiorparietal_9)(rh.parahippocampal_3)
0.0088	3.00E-05	0.86194	0.76923	(rh.bankssts_2)(rh.inferiorparietal_9)(rh.insula_5)(rh.supramarginal_9)
0.0088	3.00E-05	0.86194	0.76923	(Right-Thalamus-Proper)(rh.inferiorparietal_9)(rh.insula_2)(rh.parahippocampal_3)
0.0088	3.00E-05	0.86194	0.76923	(rh.fusiform_7)(rh.insula_2)(rh.parahippocampal_3)(rh.superiortemporal_6)
0.0088	3.00E-05	0.86194	0.76923	(Right-Pallidum)(Right-Thalamus-Proper)(rh.inferiorparietal_9)(rh.parahippocampal_3)
0.0088	3.00E-05	0.86194	0.76923	(rh.inferiorparietal_9)(rh.insula_2)(rh.isthmuscingulate_2)(rh.parahippocampal_3)
0.0088	3.00E-05	0.86194	0.76923	(Right-Pallidum)(rh.inferiorparietal_9)(rh.isthmuscingulate_2)(rh.parahippocampal_3)
0.0088	3.00E-05	0.86194	0.76923	(rh.inferiorparietal_9)(rh.insula_2)(rh.parahippocampal_3)
0.0088	3.00E-05	0.86194	0.76923	(rh.fusiform_7)(rh.inferiortemporal_2)(rh.isthmuscingulate_2)(rh.precuneus_2)
0.0088	3.00E-05	0.86194	0.76923	(Right-Pallidum)(rh.fusiform_7)(rh.parahippocampal_3)(rh.superiortemporal_6)
0.0088	3.00E-05	0.86194	0.76923	(rh.fusiform_7)(rh.insula_5)(rh.precuneus_2)(rh.superiortemporal_6)
0.0088	3.00E-05	0.86194	0.76923	(rh.fusiform_7)(rh.inferiortemporal_2)(rh.insula_2)(rh.precuneus_2)
0.0088	3.00E-05	0.86194	0.76923	(rh.inferiorparietal_9)(rh.insula_5)(rh.lingual_7)(rh.supramarginal_9)
0.0088	3.00E-05	0.86194	0.76923	(rh.entorhinal_1)(rh.fusiform_7)(rh.inferiorparietal_10)(rh.precuneus_2)
0.0088	3.00E-05	0.86194	0.76923	(Right-Thalamus-Proper)(rh.fusiform_7)(rh.inferiortemporal_2)(rh.precuneus_2)
0.0088	3.00E-05	0.86194	0.76923	(rh.fusiform_7)(rh.insula_2)(rh.precuneus_2)(rh.precuneus_7)
0.0088	3.00E-05	0.86194	0.76923	(Right-Thalamus-Proper)(rh.fusiform_7)(rh.precuneus_2)(rh.precuneus_7)
0.0088	3.00E-05	0.86194	0.76923	(Right-Pallidum)(rh.fusiform_7)(rh.inferiortemporal_2)(rh.precuneus_2)
0.0088	3.00E-05	0.86194	0.76923	(Right-Putamen)(rh.inferiorparietal_9)(rh.isthmuscingulate_2)(rh.parahippocampal_3)
0.0088	3.00E-05	0.86194	0.76923	(rh.fusiform_7)(rh.superiortemporal_3)(rh.superiortemporal_6)(rh.supramarginal_9)
0.0088	3.00E-05	0.86194	0.76923	(Right-Thalamus-Proper)(rh.inferiorparietal_9)(rh.isthmuscingulate_2)(rh.parahippocamp[illegible]
0.0088	3.00E-05	0.86194	0.76923	(Right-Pallidum)(Right-Putamen)(rh.inferiorparietal_9)(rh.parahippocampal_3)
0.0088	3.00E-05	0.86194	0.76923	(rh.entorhinal_1)(rh.fusiform_7)(rh.parahippocampal_3)(rh.supramarginal_9)
0.0088	3.00E-05	0.86194	0.76923	(rh.fusiform_7)(rh.precuneus_2)(rh.precuneus_7)
0.0088	3.00E-05	0.86194	0.76923	(rh.inferiorparietal_9)(rh.insula_5)(rh.precuneus_2)(rh.superiortemporal_9)
0.0088	3.00E-05	0.86194	0.76923	(Right-Pallidum)(rh.inferiorparietal_9)(rh.insula_2)(rh.parahippocampal_3)

0.0088	3.00E-05	0.86194	0.76923	(rh.inferiorparietal_9)(rh.isthmuscingulate_2)(rh.parahippocampal_3)
0.0088	3.00E-05	0.86194	0.76923	(Right-Thalamus-Proper)(rh.fusiform_7)(rh.parahippocampal_3)(rh.superiortemporal_6)
0.0088	3.00E-05	0.86194	0.76923	(Right-Putamen)(Right-Thalamus-Proper)(rh.inferiorparietal_9)(rh.parahippocampal_3)
0.0088	3.00E-05	0.86194	0.76923	(Right-Putamen)(rh.fusiform_7)(rh.precuneus_2)(rh.precuneus_7)
0.0088	3.00E-05	0.86194	0.76923	(rh.fusiform_7)(rh.inferiortemporal_2)(rh.precuneus_2)
0.0088	3.00E-05	0.86194	0.76923	(Right-Putamen)(rh.inferiorparietal_9)(rh.parahippocampal_3)
0.0088	3.00E-05	0.86194	0.76923	(Right-Putamen)(rh.fusiform_7)(rh.parahippocampal_3)(rh.superiortemporal_6)
0.0088	3.00E-05	0.86194	0.76923	(Right-Thalamus-Proper)(rh.inferiorparietal_9)(rh.parahippocampal_3)
0.0088	3.00E-05	0.86194	0.76923	(rh.fusiform_7)(rh.isthmuscingulate_2)(rh.parahippocampal_3)(rh.superiortemporal_6)
0.0088	3.00E-05	0.86194	0.76923	(rh.fusiform_7)(rh.isthmuscingulate_2)(rh.precuneus_2)(rh.precuneus_7)
0.0088	3.00E-05	0.86194	0.76923	(rh.entorhinal_1)(rh.fusiform_7)(rh.inferiorparietal_4)(rh.precuneus_2)
0.0088	3.00E-05	0.86194	0.76923	(Right-Putamen)(rh.inferiorparietal_9)(rh.insula_2)(rh.parahippocampal_3)
0.0088	3.00E-05	0.86194	0.76923	(Right-Putamen)(rh.fusiform_7)(rh.inferiortemporal_2)(rh.precuneus_2)
0.0089	3.00E-05	0.81343	0.71154	(rh.inferiorparietal_9)(rh.insula_5)(rh.precuneus_4)
0.0089	3.00E-05	0.81343	0.71154	(rh.inferiorparietal_9)(rh.insula_5)(rh.isthmuscingulate_2)(rh.precuneus_4)
0.0089	3.00E-05	0.81343	0.71154	(Right-Pallidum)(rh.inferiorparietal_9)(rh.insula_5)(rh.precuneus_4)
0.0089	3.00E-05	0.81343	0.71154	(Right-Putamen)(rh.inferiorparietal_9)(rh.insula_5)(rh.precuneus_4)
0.0089	3.00E-05	0.81343	0.71154	(rh.fusiform_7)(rh.inferiorparietal_10)(rh.inferiorparietal_9)(rh.precuneus_3)
0.0089	3.00E-05	0.81343	0.71154	(rh.fusiform_7)(rh.isthmuscingulate_1)(rh.precuneus_7)(rh.superiortemporal_3)
0.0089	3.00E-05	0.81343	0.71154	(rh.entorhinal_1)(rh.inferiorparietal_9)(rh.parahippocampal_2)(rh.supramarginal_9)
0.0089	3.00E-05	0.81343	0.71154	(rh.inferiorparietal_9)(rh.insula_2)(rh.insula_5)(rh.precuneus_4)
0.0089	3.00E-05	0.81343	0.71154	(Right-Caudate)(rh.fusiform_7)(rh.isthmuscingulate_1)(rh.precuneus_7)
0.0089	3.00E-05	0.81343	0.71154	(Right-Thalamus-Proper)(rh.inferiorparietal_9)(rh.insula_5)(rh.precuneus_4)
0.0089	3.00E-05	0.81343	0.71154	(rh.entorhinal_1)(rh.inferiorparietal_9)(rh.parahippocampal_3)(rh.supramarginal_9)
0.00896	3.00E-05	0.86567	0.77404	(rh.entorhinal_1)(rh.fusiform_7)(rh.fusiform_8)(rh.parahippocampal_3)
0.00896	3.00E-05	0.86567	0.77404	(rh.entorhinal_1)(rh.fusiform_7)(rh.superiortemporal_3)(rh.supramarginal_9)
0.00896	3.00E-05	0.86567	0.77404	(rh.inferiorparietal_9)(rh.insula_4)(rh.precuneus_2)(rh.superiortemporal_3)
0.00911	3.00E-05	0.8694	0.77885	(rh.entorhinal_1)(rh.fusiform_7)(rh.lingual_7)(rh.supramarginal_9)
0.00911	3.00E-05	0.8694	0.77885	(Right-Thalamus-Proper)(rh.inferiorparietal_9)(rh.precuneus_2)(rh.superiortemporal_9)
0.00911	3.00E-05	0.8694	0.77885	(rh.inferiorparietal_9)(rh.insula_2)(rh.precuneus_2)(rh.superiortemporal_9)
0.00911	3.00E-05	0.8694	0.77885	(rh.inferiorparietal_9)(rh.insula_4)(rh.lingual_7)(rh.precuneus_2)
0.00911	3.00E-05	0.8694	0.77885	(rh.bankssts_2)(rh.entorhinal_1)(rh.fusiform_7)(rh.supramarginal_9)
0.00911	3.00E-05	0.8694	0.77885	(rh.bankssts_2)(rh.inferiorparietal_9)(rh.insula_4)(rh.precuneus_2)
0.00911	3.00E-05	0.8694	0.77885	(Right-Pallidum)(rh.inferiorparietal_9)(rh.precuneus_2)(rh.superiortemporal_9)
0.00911	3.00E-05	0.8694	0.77885	(rh.inferiorparietal_9)(rh.isthmuscingulate_2)(rh.precuneus_2)(rh.superiortemporal_9)

0.00911	3.00E-05	0.8694	0.77885	(rh.entorhinal_1)(rh.fusiform_7)(rh.fusiform_8)(rh.superiortemporal_3)
0.00911	3.00E-05	0.8694	0.77885	(rh.inferiorparietal_9)(rh.precuneus_2)(rh.superiortemporal_9)
0.00911	3.00E-05	0.8694	0.77885	(Right-Putamen)(rh.inferiorparietal_9)(rh.precuneus_2)(rh.superiortemporal_9)
0.00919	3.00E-05	0.81716	0.71635	(rh.inferiorparietal_9)(rh.parahippocampal_2)(rh.precuneus_2)(rh.superiortemporal_6)
0.00919	3.00E-05	0.81716	0.71635	(Right-Accumbens-area)(rh.fusiform_7)(rh.superiortemporal_6)(rh.superiortemporal_9)
0.00919	4.00E-05	0.81716	0.71635	(rh.entorhinal_1)(rh.inferiorparietal_9)(rh.insula_4)(rh.parahippocampal_3)
0.00919	4.00E-05	0.81716	0.71635	(Right-Accumbens-area)(rh.fusiform_7)(rh.insula_5)(rh.superiortemporal_6)
0.00919	4.00E-05	0.81716	0.71635	(rh.bankssts_2)(rh.fusiform_7)(rh.isthmuscingulate_1)(rh.precuneus_7)
0.00923	4.00E-05	0.92537	0.85096	(Right-Putamen)(rh.fusiform_7)
0.00923	4.00E-05	0.92537	0.85096	(Right-Putamen)(Right-Thalamus-Proper)(rh.fusiform_7)(rh.isthmuscingulate_2)
0.00923	4.00E-05	0.92537	0.85096	(Right-Putamen)(Right-Thalamus-Proper)(rh.fusiform_7)
0.00923	4.00E-05	0.92537	0.85096	(rh.fusiform_7)
0.00923	4.00E-05	0.92537	0.85096	(rh.fusiform_7)(rh.isthmuscingulate_2)
0.00923	4.00E-05	0.92537	0.85096	(Right-Thalamus-Proper)(rh.fusiform_7)(rh.isthmuscingulate_2)
0.00923	4.00E-05	0.92537	0.85096	(Right-Putamen)(rh.fusiform_7)(rh.isthmuscingulate_2)
0.00923	4.00E-05	0.92537	0.85096	(Right-Putamen)(rh.fusiform_7)(rh.insula_2)(rh.isthmuscingulate_2)
0.00923	4.00E-05	0.92537	0.85096	(Right-Putamen)(Right-Thalamus-Proper)(rh.fusiform_7)(rh.insula_2)
0.00923	4.00E-05	0.92537	0.85096	(rh.fusiform_7)(rh.insula_2)(rh.isthmuscingulate_2)
0.00923	4.00E-05	0.92537	0.85096	(Right-Thalamus-Proper)(rh.fusiform_7)(rh.insula_2)
0.00923	4.00E-05	0.92537	0.85096	(Right-Thalamus-Proper)(rh.fusiform_7)
0.00923	4.00E-05	0.92537	0.85096	(Right-Thalamus-Proper)(rh.fusiform_7)(rh.insula_2)(rh.isthmuscingulate_2)
0.00923	4.00E-05	0.92537	0.85096	(rh.fusiform_7)(rh.insula_2)
0.00923	4.00E-05	0.92537	0.85096	(Right-Putamen)(rh.fusiform_7)(rh.insula_2)
0.00925	4.00E-05	0.87313	0.78365	(rh.fusiform_7)(rh.fusiform_8)(rh.inferiorparietal_4)(rh.parahippocampal_3)
0.00925	4.00E-05	0.87313	0.78365	(rh.inferiorparietal_9)(rh.insula_2)(rh.insula_5)(rh.superiortemporal_3)
0.00925	4.00E-05	0.87313	0.78365	(Right-Putamen)(rh.inferiorparietal_9)(rh.insula_5)(rh.superiortemporal_3)
0.00925	4.00E-05	0.87313	0.78365	(rh.inferiorparietal_9)(rh.parahippocampal_2)(rh.superiortemporal_1)(rh.superiortempor
0.00925	4.00E-05	0.87313	0.78365	(rh.entorhinal_1)(rh.fusiform_7)(rh.insula_5)(rh.parahippocampal_3)
0.00925	4.00E-05	0.87313	0.78365	(Right-Pallidum)(rh.inferiorparietal_9)(rh.insula_5)(rh.superiortemporal_3)
0.00925	4.00E-05	0.87313	0.78365	(rh.entorhinal_1)(rh.fusiform_7)(rh.fusiform_8)(rh.lingual_7)
0.00925	4.00E-05	0.87313	0.78365	(rh.fusiform_7)(rh.lingual_7)(rh.superiortemporal_3)(rh.superiortemporal_6)
0.00925	4.00E-05	0.87313	0.78365	(Right-Thalamus-Proper)(rh.inferiorparietal_9)(rh.insula_5)(rh.superiortemporal_3)
0.00925	4.00E-05	0.87313	0.78365	(rh.inferiorparietal_9)(rh.insula_5)(rh.isthmuscingulate_2)(rh.superiortemporal_3)
0.00925	4.00E-05	0.87313	0.78365	(rh.inferiorparietal_9)(rh.parahippocampal_2)(rh.precuneus_2)(rh.superiortemporal_1)
0.00925	4.00E-05	0.87313	0.78365	(rh.inferiorparietal_9)(rh.insula_5)(rh.superiortemporal_3)

0.00925	4.00E-05	0.87313	0.78365	(rh.bankssts_2)(rh.fusiform_7)(rh.superiortemporal_3)(rh.superiortemporal_6)
0.00925	4.00E-05	0.87313	0.78365	(rh.bankssts_2)(rh.entorhinal_1)(rh.fusiform_7)(rh.fusiform_8)
0.00938	4.00E-05	0.87687	0.78846	(rh.inferiorparietal_9)(rh.precuneus_2)(rh.superiortemporal_1)(rh.supramarginal_9)
0.00938	4.00E-05	0.87687	0.78846	(Right-Thalamus-Proper)(rh.inferiorparietal_9)(rh.insula_5)(rh.lingual_7)
0.00938	4.00E-05	0.87687	0.78846	(Right-Thalamus-Proper)(rh.bankssts_2)(rh.inferiorparietal_9)(rh.insula_5)
0.00938	4.00E-05	0.87687	0.78846	(rh.inferiorparietal_9)(rh.insula_5)(rh.isthmuscingulate_2)(rh.lingual_7)
0.00938	4.00E-05	0.87687	0.78846	(Right-Putamen)(rh.bankssts_2)(rh.inferiorparietal_9)(rh.insula_5)
0.00938	4.00E-05	0.87687	0.78846	(rh.inferiorparietal_9)(rh.insula_5)(rh.lingual_7)
0.00938	4.00E-05	0.87687	0.78846	(rh.bankssts_2)(rh.inferiorparietal_9)(rh.insula_5)
0.00938	4.00E-05	0.87687	0.78846	(rh.bankssts_2)(rh.inferiorparietal_9)(rh.insula_2)(rh.insula_5)
0.00938	4.00E-05	0.87687	0.78846	(Right-Pallidum)(rh.inferiorparietal_9)(rh.insula_5)(rh.lingual_7)
0.00938	4.00E-05	0.87687	0.78846	(Right-Pallidum)(rh.bankssts_2)(rh.inferiorparietal_9)(rh.insula_5)
0.00938	4.00E-05	0.87687	0.78846	(rh.bankssts_2)(rh.inferiorparietal_9)(rh.insula_5)(rh.isthmuscingulate_2)
0.00938	4.00E-05	0.87687	0.78846	(rh.fusiform_7)(rh.fusiform_8)(rh.insula_4)(rh.parahippocampal_3)
0.00938	4.00E-05	0.87687	0.78846	(rh.inferiorparietal_9)(rh.insula_2)(rh.insula_5)(rh.lingual_7)
0.00938	4.00E-05	0.87687	0.78846	(rh.fusiform_7)(rh.fusiform_8)(rh.parahippocampal_2)(rh.parahippocampal_3)
0.00938	4.00E-05	0.87687	0.78846	(Right-Putamen)(rh.inferiorparietal_9)(rh.insula_5)(rh.lingual_7)
0.0094	4.00E-05	0.92164	0.84615	(Right-Pallidum)(rh.fusiform_7)(rh.lingual_7)
0.0094	4.00E-05	0.92164	0.84615	(Right-Pallidum)(Right-Thalamus-Proper)(rh.bankssts_2)(rh.fusiform_7)
0.0094	4.00E-05	0.92164	0.84615	(Right-Pallidum)(rh.bankssts_2)(rh.fusiform_7)(rh.insula_2)
0.0094	4.00E-05	0.92164	0.84615	(Right-Pallidum)(rh.fusiform_7)(rh.insula_2)(rh.lingual_7)
0.0094	4.00E-05	0.92164	0.84615	(Right-Pallidum)(Right-Putamen)(rh.fusiform_7)(rh.lingual_7)
0.0094	4.00E-05	0.92164	0.84615	(Right-Pallidum)(Right-Putamen)(rh.bankssts_2)(rh.fusiform_7)
0.0094	4.00E-05	0.92164	0.84615	(Right-Pallidum)(rh.bankssts_2)(rh.fusiform_7)(rh.isthmuscingulate_2)
0.0094	4.00E-05	0.92164	0.84615	(Right-Pallidum)(rh.bankssts_2)(rh.fusiform_7)
0.0094	4.00E-05	0.92164	0.84615	(Right-Pallidum)(rh.fusiform_7)(rh.isthmuscingulate_2)(rh.lingual_7)
0.0094	4.00E-05	0.92164	0.84615	(Right-Pallidum)(Right-Thalamus-Proper)(rh.fusiform_7)(rh.lingual_7)
0.00948	4.00E-05	0.8209	0.72115	(rh.entorhinal_1)(rh.fusiform_7)(rh.isthmuscingulate_2)(rh.precuneus_4)
0.00948	4.00E-05	0.8209	0.72115	(Right-Pallidum)(rh.entorhinal_1)(rh.fusiform_7)(rh.precuneus_4)
0.00948	4.00E-05	0.8209	0.72115	(Right-Putamen)(rh.entorhinal_1)(rh.fusiform_7)(rh.precuneus_4)
0.00948	4.00E-05	0.8209	0.72115	(rh.fusiform_8)(rh.inferiorparietal_9)(rh.parahippocampal_3)(rh.supramarginal_9)
0.00948	4.00E-05	0.8209	0.72115	(rh.entorhinal_1)(rh.inferiorparietal_9)(rh.insula_4)(rh.superiortemporal_3)
0.00948	4.00E-05	0.8209	0.72115	(Right-Pallidum)(rh.fusiform_7)(rh.inferiorparietal_9)(rh.superiortemporal_1)
0.00948	5.00E-05	0.8209	0.72115	(rh.entorhinal_1)(rh.inferiorparietal_9)(rh.isthmuscingulate_2)(rh.supramarginal_9)
0.00948	5.00E-05	0.8209	0.72115	(Right-Pallidum)(rh.fusiform_7)(rh.precuneus_4)(rh.precuneus_7)

0.00948	5.00E-05	0.8209	0.72115	(rh.entorhinal_1)(rh.fusiform_7)(rh.precuneus_4)(rh.superiortemporal_9)
0.00948	5.00E-05	0.8209	0.72115	(rh.entorhinal_1)(rh.fusiform_7)(rh.insula_2)(rh.precuneus_4)
0.00948	5.00E-05	0.8209	0.72115	(rh.entorhinal_1)(rh.inferiorparietal_9)(rh.superiortemporal_9)(rh.supramarginal_9)
0.00948	5.00E-05	0.8209	0.72115	(Right-Pallidum)(rh.entorhinal_1)(rh.inferiorparietal_9)(rh.supramarginal_9)
0.00948	5.00E-05	0.8209	0.72115	(Right-Putamen)(rh.entorhinal_1)(rh.inferiorparietal_9)(rh.supramarginal_9)
0.00948	5.00E-05	0.8209	0.72115	(Right-Thalamus-Proper)(rh.entorhinal_1)(rh.fusiform_7)(rh.precuneus_4)
0.00948	5.00E-05	0.8209	0.72115	(Right-Thalamus-Proper)(rh.entorhinal_1)(rh.inferiorparietal_9)(rh.supramarginal_9)
0.00948	5.00E-05	0.8209	0.72115	(rh.fusiform_7)(rh.inferiorparietal_4)(rh.inferiorparietal_9)(rh.insula_4)
0.00948	5.00E-05	0.8209	0.72115	(rh.fusiform_7)(rh.parahippocampal_3)(rh.precuneus_4)(rh.superiortemporal_1)
0.00948	5.00E-05	0.8209	0.72115	(rh.fusiform_7)(rh.inferiorparietal_10)(rh.inferiorparietal_9)(rh.insula_4)
0.00948	5.00E-05	0.8209	0.72115	(rh.entorhinal_1)(rh.inferiorparietal_9)(rh.insula_2)(rh.supramarginal_9)
0.00949	5.00E-05	0.8806	0.79327	(rh.inferiorparietal_4)(rh.inferiorparietal_9)(rh.parahippocampal_2)(rh.precuneus_2)
0.00949	5.00E-05	0.8806	0.79327	(Right-Pallidum)(rh.entorhinal_1)(rh.fusiform_7)(rh.superiortemporal_3)
0.00949	5.00E-05	0.8806	0.79327	(Right-Putamen)(rh.entorhinal_1)(rh.fusiform_7)(rh.superiortemporal_3)
0.00949	5.00E-05	0.8806	0.79327	(Right-Thalamus-Proper)(rh.entorhinal_1)(rh.fusiform_7)(rh.superiortemporal_3)
0.00949	5.00E-05	0.8806	0.79327	(rh.entorhinal_1)(rh.fusiform_7)(rh.isthmuscingulate_2)(rh.superiortemporal_3)
0.00949	5.00E-05	0.8806	0.79327	(rh.entorhinal_1)(rh.fusiform_7)(rh.insula_2)(rh.superiortemporal_3)
0.00949	5.00E-05	0.8806	0.79327	(rh.entorhinal_1)(rh.fusiform_7)(rh.superiortemporal_3)
0.00949	5.00E-05	0.8806	0.79327	(rh.fusiform_7)(rh.fusiform_8)(rh.insula_4)(rh.precuneus_2)
0.00949	5.00E-05	0.8806	0.79327	(rh.entorhinal_1)(rh.fusiform_7)(rh.superiortemporal_3)(rh.superiortemporal_9)
0.00955	5.00E-05	0.91791	0.84135	(Right-Pallidum)(Right-Putamen)(rh.fusiform_7)(rh.superiortemporal_3)
0.00955	5.00E-05	0.91791	0.84135	(Right-Pallidum)(Right-Thalamus-Proper)(rh.fusiform_7)(rh.superiortemporal_3)
0.00955	5.00E-05	0.91791	0.84135	(Right-Pallidum)(rh.fusiform_7)(rh.insula_2)(rh.superiortemporal_3)
0.00955	5.00E-05	0.91791	0.84135	(Right-Pallidum)(rh.fusiform_7)(rh.isthmuscingulate_2)(rh.superiortemporal_3)
0.00955	5.00E-05	0.91791	0.84135	(Right-Pallidum)(rh.fusiform_7)(rh.superiortemporal_3)
0.00959	5.00E-05	0.88433	0.79808	(Right-Thalamus-Proper)(rh.entorhinal_1)(rh.fusiform_7)(rh.lingual_7)
0.00959	5.00E-05	0.88433	0.79808	(rh.entorhinal_1)(rh.fusiform_7)(rh.lingual_7)(rh.superiortemporal_9)
0.00959	5.00E-05	0.88433	0.79808	(Right-Pallidum)(rh.bankssts_2)(rh.entorhinal_1)(rh.fusiform_7)
0.00959	5.00E-05	0.88433	0.79808	(rh.inferiorparietal_10)(rh.inferiorparietal_9)(rh.insula_2)(rh.supramarginal_9)
0.00959	5.00E-05	0.88433	0.79808	(Right-Putamen)(rh.bankssts_2)(rh.entorhinal_1)(rh.fusiform_7)
0.00959	5.00E-05	0.88433	0.79808	(rh.bankssts_2)(rh.entorhinal_1)(rh.fusiform_7)
0.00959	5.00E-05	0.88433	0.79808	(Right-Pallidum)(rh.inferiorparietal_9)(rh.superiortemporal_3)(rh.supramarginal_9)
0.00959	5.00E-05	0.88433	0.79808	(rh.entorhinal_1)(rh.fusiform_7)(rh.insula_2)(rh.lingual_7)
0.00959	5.00E-05	0.88433	0.79808	(rh.fusiform_7)(rh.parahippocampal_2)(rh.precuneus_2)(rh.precuneus_3)
0.00959	5.00E-05	0.88433	0.79808	(rh.entorhinal_1)(rh.fusiform_7)(rh.isthmuscingulate_2)(rh.lingual_7)

0.00959	5.00E-05	0.88433	0.79808	(Right-Pallidum)(rh.entorhinal_1)(rh.fusiform_7)(rh.lingual_7)
0.00959	5.00E-05	0.88433	0.79808	(rh.entorhinal_1)(rh.fusiform_7)(rh.lingual_7)
0.00959	5.00E-05	0.88433	0.79808	(Right-Thalamus-Proper)(rh.bankssts_2)(rh.entorhinal_1)(rh.fusiform_7)
0.00959	5.00E-05	0.88433	0.79808	(Right-Putamen)(rh.entorhinal_1)(rh.fusiform_7)(rh.lingual_7)
0.00959	6.00E-05	0.88433	0.79808	(rh.bankssts_2)(rh.entorhinal_1)(rh.fusiform_7)(rh.isthmuscingulate_2)
0.00959	6.00E-05	0.88433	0.79808	(rh.bankssts_2)(rh.entorhinal_1)(rh.fusiform_7)(rh.insula_2)
0.00959	6.00E-05	0.88433	0.79808	(rh.bankssts_2)(rh.entorhinal_1)(rh.fusiform_7)(rh.superiortemporal_9)
0.00959	6.00E-05	0.88433	0.79808	(rh.fusiform_7)(rh.inferiorparietal_10)(rh.parahippocampal_3)(rh.superiortemporal_3)
0.00959	6.00E-05	0.88433	0.79808	(rh.fusiform_7)(rh.insula_5)(rh.parahippocampal_3)(rh.supramarginal_9)
0.00966	6.00E-05	0.91418	0.83654	(Right-Caudate)(rh.bankssts_2)(rh.fusiform_7)(rh.isthmuscingulate_2)
0.00966	6.00E-05	0.91418	0.83654	(Right-Putamen)(rh.fusiform_7)(rh.parahippocampal_2)
0.00966	6.00E-05	0.91418	0.83654	(rh.fusiform_7)(rh.insula_2)(rh.parahippocampal_2)
0.00966	6.00E-05	0.91418	0.83654	(Right-Thalamus-Proper)(rh.fusiform_7)(rh.insula_2)(rh.parahippocampal_2)
0.00966	6.00E-05	0.91418	0.83654	(Right-Caudate)(rh.fusiform_7)(rh.insula_2)(rh.lingual_7)
0.00966	6.00E-05	0.91418	0.83654	(Right-Putamen)(Right-Thalamus-Proper)(rh.fusiform_7)(rh.parahippocampal_2)
0.00966	6.00E-05	0.91418	0.83654	(rh.fusiform_7)(rh.isthmuscingulate_2)(rh.parahippocampal_2)
0.00966	6.00E-05	0.91418	0.83654	(Right-Caudate)(Right-Putamen)(rh.fusiform_7)(rh.lingual_7)
0.00966	6.00E-05	0.91418	0.83654	(Right-Caudate)(Right-Thalamus-Proper)(rh.bankssts_2)(rh.fusiform_7)
0.00966	6.00E-05	0.91418	0.83654	(Right-Caudate)(Right-Thalamus-Proper)(rh.fusiform_7)(rh.lingual_7)
0.00966	6.00E-05	0.91418	0.83654	(rh.bankssts_2)(rh.fusiform_7)(rh.insula_5)(rh.lingual_7)
0.00966	6.00E-05	0.91418	0.83654	(Right-Caudate)(rh.fusiform_7)(rh.isthmuscingulate_2)(rh.lingual_7)
0.00966	6.00E-05	0.91418	0.83654	(rh.fusiform_7)(rh.parahippocampal_2)
0.00966	6.00E-05	0.91418	0.83654	(Right-Caudate)(Right-Putamen)(rh.bankssts_2)(rh.fusiform_7)
0.00966	6.00E-05	0.91418	0.83654	(Right-Thalamus-Proper)(rh.bankssts_2)(rh.inferiorparietal_9)(rh.lingual_7)
0.00966	6.00E-05	0.91418	0.83654	(Right-Caudate)(rh.bankssts_2)(rh.fusiform_7)(rh.insula_2)
0.00966	6.00E-05	0.91418	0.83654	(Right-Caudate)(rh.fusiform_7)(rh.lingual_7)
0.00966	6.00E-05	0.91418	0.83654	(Right-Putamen)(rh.fusiform_7)(rh.insula_2)(rh.parahippocampal_2)
0.00966	6.00E-05	0.91418	0.83654	(Right-Putamen)(rh.fusiform_7)(rh.isthmuscingulate_2)(rh.parahippocampal_2)
0.00966	6.00E-05	0.91418	0.83654	(rh.bankssts_2)(rh.inferiorparietal_9)(rh.isthmuscingulate_2)(rh.lingual_7)
0.00966	6.00E-05	0.91418	0.83654	(rh.bankssts_2)(rh.inferiorparietal_9)(rh.lingual_7)
0.00966	6.00E-05	0.91418	0.83654	(Right-Putamen)(rh.bankssts_2)(rh.inferiorparietal_9)(rh.lingual_7)
0.00966	6.00E-05	0.91418	0.83654	(Right-Thalamus-Proper)(rh.fusiform_7)(rh.parahippocampal_2)
0.00966	7.00E-05	0.91418	0.83654	(rh.fusiform_7)(rh.insula_2)(rh.isthmuscingulate_2)(rh.parahippocampal_2)
0.00966	7.00E-05	0.91418	0.83654	(Right-Thalamus-Proper)(rh.fusiform_7)(rh.isthmuscingulate_2)(rh.parahippocampal_2)
0.00966	7.00E-05	0.91418	0.83654	(Right-Caudate)(rh.bankssts_2)(rh.fusiform_7)

0.00967	7.00E-05	0.88806	0.80288	(rh.inferiorparietal_10)(rh.inferiorparietal_9)(rh.insula_2)(rh.parahippocampal_2)
0.00967	7.00E-05	0.88806	0.80288	(rh.fusiform_7)(rh.insula_4)(rh.parahippocampal_3)(rh.superiortemporal_3)
0.00967	7.00E-05	0.88806	0.80288	(rh.fusiform_7)(rh.inferiorparietal_10)(rh.lingual_7)(rh.parahippocampal_3)
0.00967	7.00E-05	0.88806	0.80288	(Right-Caudate)(rh.fusiform_7)(rh.parahippocampal_2)(rh.parahippocampal_3)
0.00967	7.00E-05	0.88806	0.80288	(Right-Caudate)(rh.fusiform_7)(rh.insula_5)(rh.parahippocampal_3)
0.00967	7.00E-05	0.88806	0.80288	(rh.bankssts_2)(rh.inferiorparietal_4)(rh.inferiorparietal_9)(rh.superiortemporal_3)
0.00967	7.00E-05	0.88806	0.80288	(Right-Caudate)(rh.inferiorparietal_9)(rh.insula_2)(rh.supramarginal_9)
0.00967	7.00E-05	0.88806	0.80288	(rh.fusiform_7)(rh.inferiorparietal_4)(rh.insula_5)(rh.parahippocampal_3)
0.00967	7.00E-05	0.88806	0.80288	(rh.inferiorparietal_4)(rh.inferiorparietal_9)(rh.insula_2)(rh.superiortemporal_3)
0.00967	7.00E-05	0.88806	0.80288	(rh.inferiorparietal_4)(rh.inferiorparietal_9)(rh.lingual_7)(rh.superiortemporal_3)
0.00967	7.00E-05	0.88806	0.80288	(rh.fusiform_7)(rh.precuneus_2)(rh.superiortemporal_1)(rh.superiortemporal_3)
0.00967	7.00E-05	0.88806	0.80288	(rh.bankssts_2)(rh.fusiform_7)(rh.inferiorparietal_10)(rh.parahippocampal_3)
0.00967	7.00E-05	0.88806	0.80288	(rh.fusiform_7)(rh.inferiorparietal_4)(rh.precuneus_2)(rh.supramarginal_9)
0.00974	7.00E-05	0.91045	0.83173	(Right-Pallidum)(rh.bankssts_2)(rh.fusiform_7)(rh.parahippocampal_2)
0.00974	7.00E-05	0.91045	0.83173	(Right-Putamen)(Right-Thalamus-Proper)(rh.fusiform_7)(rh.supramarginal_9)
0.00974	8.00E-05	0.91045	0.83173	(rh.inferiorparietal_9)(rh.insula_2)
0.00974	8.00E-05	0.91045	0.83173	(Right-Caudate)(Right-Thalamus-Proper)(rh.fusiform_7)(rh.superiortemporal_3)
0.00974	8.00E-05	0.91045	0.83173	(Right-Putamen)(Right-Thalamus-Proper)(rh.inferiorparietal_9)(rh.insula_2)
0.00974	8.00E-05	0.91045	0.83173	(rh.fusiform_7)(rh.supramarginal_9)
0.00974	8.00E-05	0.91045	0.83173	(rh.fusiform_7)(rh.insula_2)(rh.isthmuscingulate_2)(rh.supramarginal_9)
0.00974	8.00E-05	0.91045	0.83173	(rh.fusiform_7)(rh.insula_2)(rh.supramarginal_9)
0.00974	8.00E-05	0.91045	0.83173	(Right-Putamen)(rh.fusiform_7)(rh.supramarginal_9)
0.00974	8.00E-05	0.91045	0.83173	(rh.fusiform_7)(rh.insula_5)(rh.lingual_7)(rh.superiortemporal_3)
0.00974	8.00E-05	0.91045	0.83173	(Right-Caudate)(rh.fusiform_7)(rh.superiortemporal_3)
0.00974	8.00E-05	0.91045	0.83173	(Right-Putamen)(rh.inferiorparietal_9)(rh.insula_2)
0.00974	8.00E-05	0.91045	0.83173	(Right-Putamen)(rh.inferiorparietal_9)(rh.insula_2)(rh.isthmuscingulate_2)
0.00974	8.00E-05	0.91045	0.83173	(Right-Caudate)(rh.fusiform_7)(rh.insula_2)(rh.superiortemporal_3)
0.00974	8.00E-05	0.91045	0.83173	(Right-Thalamus-Proper)(rh.inferiorparietal_9)(rh.insula_2)
0.00974	8.00E-05	0.91045	0.83173	(Right-Thalamus-Proper)(rh.fusiform_7)(rh.supramarginal_9)
0.00974	8.00E-05	0.91045	0.83173	(Right-Pallidum)(rh.fusiform_7)(rh.lingual_7)(rh.parahippocampal_2)
0.00974	8.00E-05	0.91045	0.83173	(Right-Thalamus-Proper)(rh.inferiorparietal_9)(rh.insula_2)(rh.isthmuscingulate_2)
0.00974	9.00E-05	0.91045	0.83173	(rh.inferiorparietal_9)(rh.insula_2)(rh.isthmuscingulate_2)
0.00974	9.00E-05	0.91045	0.83173	(rh.bankssts_2)(rh.fusiform_7)(rh.insula_5)(rh.superiortemporal_3)
0.00974	9.00E-05	0.91045	0.83173	(Right-Putamen)(rh.fusiform_7)(rh.insula_2)(rh.supramarginal_9)
0.00974	9.00E-05	0.91045	0.83173	(Right-Caudate)(Right-Putamen)(rh.fusiform_7)(rh.superiortemporal_3)

0.00974	9.00E-05	0.91045	0.83173	(Right-Caudate)(rh.fusiform_7)(rh.isthmuscingulate_2)(rh.superiortemporal_3)
0.00974	9.00E-05	0.91045	0.83173	(Right-Thalamus-Proper)(rh.fusiform_7)(rh.isthmuscingulate_2)(rh.supramarginal_9)
0.00974	9.00E-05	0.91045	0.83173	(Right-Thalamus-Proper)(rh.fusiform_7)(rh.insula_2)(rh.supramarginal_9)
0.00974	9.00E-05	0.91045	0.83173	(rh.fusiform_7)(rh.isthmuscingulate_2)(rh.supramarginal_9)
0.00974	9.00E-05	0.91045	0.83173	(Right-Putamen)(rh.fusiform_7)(rh.isthmuscingulate_2)(rh.supramarginal_9)
0.00974	9.00E-05	0.89179	0.80769	(Right-Pallidum)(rh.fusiform_7)(rh.inferiorparietal_4)(rh.parahippocampal_3)
0.00974	9.00E-05	0.89179	0.80769	(rh.bankssts_2)(rh.fusiform_7)(rh.precuneus_2)(rh.superiortemporal_1)
0.00974	9.00E-05	0.89179	0.80769	(rh.bankssts_2)(rh.fusiform_7)(rh.insula_4)(rh.parahippocampal_3)
0.00974	0.0001	0.89179	0.80769	(Right-Caudate)(rh.fusiform_7)(rh.inferiorparietal_4)(rh.precuneus_2)
0.00974	0.0001	0.89179	0.80769	(rh.fusiform_7)(rh.fusiform_8)(rh.lingual_7)(rh.superiortemporal_3)
0.00974	0.0001	0.89179	0.80769	(rh.fusiform_7)(rh.insula_4)(rh.parahippocampal_2)(rh.precuneus_2)
0.00974	0.0001	0.89179	0.80769	(rh.fusiform_7)(rh.insula_4)(rh.insula_5)(rh.parahippocampal_3)
0.00974	0.0001	0.89179	0.80769	(Right-Thalamus-Proper)(rh.fusiform_7)(rh.inferiorparietal_4)(rh.parahippocampal_3)
0.00974	0.0001	0.89179	0.80769	(rh.fusiform_7)(rh.inferiorparietal_4)(rh.insula_2)(rh.parahippocampal_3)
0.00974	0.0001	0.89179	0.80769	(rh.bankssts_2)(rh.fusiform_7)(rh.fusiform_8)(rh.superiortemporal_3)
0.00974	0.0001	0.89179	0.80769	(Right-Thalamus-Proper)(rh.inferiorparietal_9)(rh.parahippocampal_2)(rh.supramarginal_
0.00974	0.0001	0.89179	0.80769	(rh.fusiform_7)(rh.inferiorparietal_4)(rh.isthmuscingulate_2)(rh.parahippocampal_3)
0.00974	0.0001	0.89179	0.80769	(Right-Putamen)(rh.fusiform_7)(rh.inferiorparietal_4)(rh.parahippocampal_3)
0.00974	0.00011	0.89179	0.80769	(rh.fusiform_7)(rh.lingual_7)(rh.precuneus_2)(rh.superiortemporal_1)
0.00974	0.00011	0.89179	0.80769	(Right-Putamen)(rh.inferiorparietal_9)(rh.parahippocampal_2)(rh.supramarginal_9)
0.00974	0.00011	0.89179	0.80769	(rh.fusiform_7)(rh.inferiorparietal_4)(rh.parahippocampal_3)
0.00974	0.00011	0.89179	0.80769	(rh.fusiform_7)(rh.insula_4)(rh.lingual_7)(rh.parahippocampal_3)
0.00974	0.00011	0.89179	0.80769	(rh.inferiorparietal_9)(rh.isthmuscingulate_2)(rh.parahippocampal_2)(rh.supramarginal_[illegible]
0.00977	0.00011	0.82463	0.72596	(rh.entorhinal_1)(rh.inferiorparietal_9)(rh.insula_5)(rh.parahippocampal_3)
0.00977	0.00011	0.82463	0.72596	(rh.inferiorparietal_9)(rh.lingual_7)(rh.precuneus_2)(rh.superiortemporal_6)
0.00977	0.00011	0.82463	0.72596	(rh.bankssts_2)(rh.inferiorparietal_9)(rh.precuneus_2)(rh.superiortemporal_6)
0.00977	0.00011	0.82463	0.72596	(rh.fusiform_8)(rh.inferiorparietal_9)(rh.insula_4)(rh.parahippocampal_3)
0.00977	0.00012	0.82463	0.72596	(Right-Accumbens-area)(rh.entorhinal_1)(rh.fusiform_7)(rh.supramarginal_9)
0.00977	0.00012	0.82463	0.72596	(rh.entorhinal_1)(rh.inferiorparietal_9)(rh.insula_4)(rh.lingual_7)
0.00977	0.00012	0.82463	0.72596	(Right-Caudate)(rh.fusiform_8)(rh.inferiorparietal_9)(rh.parahippocampal_3)
0.00977	0.00012	0.82463	0.72596	(rh.bankssts_2)(rh.entorhinal_1)(rh.inferiorparietal_9)(rh.insula_4)
0.00977	0.00012	0.82463	0.72596	(rh.fusiform_8)(rh.inferiorparietal_9)(rh.precuneus_2)(rh.supramarginal_9)
0.00977	0.00012	0.82463	0.72596	(Right-Caudate)(rh.fusiform_7)(rh.fusiform_8)(rh.precuneus_4)
0.00978	0.00013	0.89552	0.8125	(Right-Thalamus-Proper)(rh.fusiform_7)(rh.parahippocampal_2)(rh.parahippocampal_3)
0.00978	0.00013	0.89552	0.8125	(Right-Pallidum)(rh.inferiorparietal_9)(rh.insula_2)(rh.parahippocampal_2)

0.00978	0.00013	0.89552	0.8125	(rh.bankssts_2)(rh.inferiorparietal_10)(rh.inferiorparietal_9)(rh.insula_2)
0.00978	0.00013	0.89552	0.8125	(rh.inferiorparietal_10)(rh.inferiorparietal_9)(rh.insula_2)(rh.lingual_7)
0.00978	0.00013	0.89552	0.8125	(rh.fusiform_7)(rh.insula_2)(rh.precuneus_2)(rh.precuneus_3)
0.00978	0.00013	0.89552	0.8125	(Right-Putamen)(rh.fusiform_7)(rh.precuneus_2)(rh.precuneus_3)
0.00978	0.00014	0.89552	0.8125	(rh.fusiform_7)(rh.isthmuscingulate_2)(rh.precuneus_2)(rh.precuneus_3)
0.00978	0.00014	0.89552	0.8125	(rh.inferiorparietal_9)(rh.insula_2)(rh.isthmuscingulate_2)(rh.supramarginal_9)
0.00978	0.00014	0.89552	0.8125	(Right-Thalamus-Proper)(rh.inferiorparietal_9)(rh.insula_2)(rh.supramarginal_9)
0.00978	0.00014	0.89552	0.8125	(Right-Putamen)(rh.fusiform_7)(rh.parahippocampal_2)(rh.parahippocampal_3)
0.00978	0.00014	0.89552	0.8125	(rh.fusiform_7)(rh.isthmuscingulate_2)(rh.parahippocampal_2)(rh.parahippocampal_3)
0.00978	0.00014	0.89552	0.8125	(rh.fusiform_7)(rh.inferiorparietal_4)(rh.insula_5)(rh.precuneus_2)
0.00978	0.00015	0.89552	0.8125	(rh.fusiform_7)(rh.precuneus_2)(rh.precuneus_3)
0.00978	0.00015	0.89552	0.8125	(rh.fusiform_7)(rh.insula_2)(rh.parahippocampal_2)(rh.parahippocampal_3)
0.00978	0.00015	0.89552	0.8125	(Right-Pallidum)(rh.fusiform_7)(rh.parahippocampal_2)(rh.parahippocampal_3)
0.00978	0.00015	0.89552	0.8125	(Right-Putamen)(rh.inferiorparietal_9)(rh.insula_2)(rh.supramarginal_9)
0.00978	0.00016	0.89552	0.8125	(Right-Thalamus-Proper)(rh.fusiform_7)(rh.precuneus_2)(rh.precuneus_3)
0.00978	0.00016	0.89552	0.8125	(rh.bankssts_2)(rh.fusiform_7)(rh.fusiform_8)(rh.lingual_7)
0.00978	0.00016	0.89552	0.8125	(rh.fusiform_7)(rh.parahippocampal_2)(rh.parahippocampal_3)
0.00978	0.00016	0.89552	0.8125	(rh.fusiform_7)(rh.precuneus_2)(rh.superiortemporal_9)(rh.supramarginal_9)
0.00979	0.00017	0.90672	0.82692	(Right-Caudate)(rh.fusiform_7)(rh.isthmuscingulate_2)(rh.parahippocampal_2)
0.00979	0.00017	0.90672	0.82692	(Right-Pallidum)(rh.fusiform_7)(rh.parahippocampal_2)(rh.superiortemporal_3)
0.00979	0.00017	0.90672	0.82692	(Right-Caudate)(Right-Putamen)(rh.fusiform_7)(rh.parahippocampal_2)
0.00979	0.00018	0.90672	0.82692	(Right-Pallidum)(rh.fusiform_7)(rh.lingual_7)(rh.supramarginal_9)
0.00979	0.00018	0.90672	0.82692	(Right-Caudate)(rh.fusiform_7)(rh.insula_2)(rh.parahippocampal_2)
0.00979	0.00018	0.90672	0.82692	(Right-Caudate)(Right-Thalamus-Proper)(rh.fusiform_7)(rh.parahippocampal_2)
0.00979	0.00019	0.90672	0.82692	(Right-Pallidum)(rh.bankssts_2)(rh.fusiform_7)(rh.supramarginal_9)
0.00979	0.00019	0.90672	0.82692	(rh.inferiorparietal_9)(rh.isthmuscingulate_2)(rh.parahippocampal_2)
0.00979	0.00019	0.90672	0.82692	(Right-Caudate)(rh.fusiform_7)(rh.parahippocampal_2)
0.00979	0.0002	0.90672	0.82692	(Right-Putamen)(rh.inferiorparietal_9)(rh.parahippocampal_2)
0.00979	0.0002	0.90672	0.82692	(Right-Putamen)(rh.inferiorparietal_9)(rh.isthmuscingulate_2)(rh.parahippocampal_2)
0.00979	0.0002	0.90672	0.82692	(Right-Thalamus-Proper)(rh.inferiorparietal_9)(rh.parahippocampal_2)
0.00979	0.00021	0.90672	0.82692	(rh.parahippocampal_2)(rh.precuneus_2)(rh.precuneus_4)(rh.superiortemporal_3)
0.00979	0.00021	0.90672	0.82692	(rh.inferiorparietal_9)(rh.parahippocampal_2)
0.00979	0.00022	0.90672	0.82692	(Right-Putamen)(Right-Thalamus-Proper)(rh.inferiorparietal_9)(rh.parahippocampal_2)
0.00979	0.00022	0.90672	0.82692	(Right-Thalamus-Proper)(rh.inferiorparietal_9)(rh.isthmuscingulate_2)(rh.parahippocamp[illegible]
0.00981	0.00023	0.89925	0.81731	(rh.fusiform_7)(rh.insula_2)(rh.parahippocampal_2)(rh.supramarginal_9)

0.00981	0.00023	0.89925	0.81731	(Right-Putamen)(rh.fusiform_7)(rh.parahippocampal_2)(rh.supramarginal_9)
0.00981	0.00024	0.89925	0.81731	(rh.fusiform_7)(rh.parahippocampal_3)(rh.superiortemporal_9)
0.00981	0.00024	0.89925	0.81731	(Right-Thalamus-Proper)(rh.fusiform_7)(rh.parahippocampal_2)(rh.supramarginal_9)
0.00981	0.00025	0.89925	0.81731	(Right-Pallidum)(Right-Thalamus-Proper)(rh.inferiorparietal_9)(rh.precuneus_2)
0.00981	0.00026	0.89925	0.81731	(Right-Thalamus-Proper)(rh.fusiform_7)(rh.parahippocampal_3)(rh.superiortemporal_9)
0.00981	0.00026	0.89925	0.81731	(Right-Caudate)(rh.inferiorparietal_9)(rh.isthmuscingulate_2)(rh.parahippocampal_2)
0.00981	0.00027	0.89925	0.81731	(Right-Putamen)(rh.fusiform_7)(rh.parahippocampal_3)(rh.superiortemporal_9)
0.00981	0.00028	0.89925	0.81731	(Right-Pallidum)(Right-Putamen)(rh.inferiorparietal_9)(rh.precuneus_2)
0.00981	0.00029	0.89925	0.81731	(Right-Caudate)(rh.fusiform_7)(rh.precuneus_2)(rh.superiortemporal_9)
0.00981	0.00029	0.89925	0.81731	(Right-Pallidum)(rh.inferiorparietal_9)(rh.superiortemporal_3)
0.00981	0.0003	0.89925	0.81731	(Right-Pallidum)(rh.inferiorparietal_9)(rh.isthmuscingulate_2)(rh.superiortemporal_3)
0.00981	0.00031	0.89925	0.81731	(Right-Pallidum)(rh.fusiform_7)(rh.inferiorparietal_10)(rh.precuneus_2)
0.00981	0.00032	0.89925	0.81731	(Right-Pallidum)(rh.inferiorparietal_9)(rh.isthmuscingulate_2)(rh.precuneus_2)
0.00981	0.00033	0.89925	0.81731	(Right-Caudate)(rh.bankssts_2)(rh.inferiorparietal_9)(rh.insula_2)
0.00981	0.00034	0.89925	0.81731	(Right-Pallidum)(rh.inferiorparietal_9)(rh.precuneus_2)
0.00981	0.00036	0.89925	0.81731	(rh.bankssts_2)(rh.inferiorparietal_9)(rh.lingual_7)(rh.supramarginal_9)
0.00981	0.00037	0.89925	0.81731	(Right-Pallidum)(rh.fusiform_7)(rh.inferiorparietal_4)(rh.precuneus_2)
0.00981	0.00038	0.89925	0.81731	(Right-Pallidum)(Right-Putamen)(rh.inferiorparietal_9)(rh.superiortemporal_3)
0.00981	0.0004	0.89925	0.81731	(Right-Caudate)(Right-Putamen)(rh.inferiorparietal_9)(rh.parahippocampal_2)
0.00981	0.00042	0.89925	0.81731	(Right-Caudate)(Right-Thalamus-Proper)(rh.inferiorparietal_9)(rh.parahippocampal_2)
0.00981	0.00043	0.89925	0.81731	(Right-Pallidum)(rh.fusiform_7)(rh.parahippocampal_3)(rh.superiortemporal_9)
0.00981	0.00045	0.89925	0.81731	(rh.fusiform_7)(rh.insula_2)(rh.parahippocampal_3)(rh.superiortemporal_9)
0.00981	0.00048	0.89925	0.81731	(rh.fusiform_7)(rh.isthmuscingulate_2)(rh.parahippocampal_2)(rh.supramarginal_9)
0.00981	0.0005	0.89925	0.81731	(rh.fusiform_7)(rh.parahippocampal_2)(rh.supramarginal_9)
0.00981	0.00053	0.89925	0.81731	(rh.fusiform_7)(rh.isthmuscingulate_2)(rh.parahippocampal_3)(rh.superiortemporal_9)
0.00981	0.00056	0.89925	0.81731	(Right-Pallidum)(Right-Thalamus-Proper)(rh.inferiorparietal_9)(rh.superiortemporal_3)
0.00981	0.00059	0.90299	0.82212	(Right-Pallidum)(rh.fusiform_7)(rh.superiortemporal_3)(rh.supramarginal_9)
0.00981	0.00063	0.90299	0.82212	(rh.fusiform_7)(rh.insula_2)(rh.insula_4)(rh.precuneus_2)
0.00981	0.00067	0.90299	0.82212	(Right-Thalamus-Proper)(rh.fusiform_7)(rh.insula_4)(rh.precuneus_2)
0.00981	0.00071	0.90299	0.82212	(Right-Caudate)(rh.fusiform_7)(rh.isthmuscingulate_2)(rh.supramarginal_9)
0.00981	0.00077	0.90299	0.82212	(Right-Caudate)(rh.fusiform_7)(rh.supramarginal_9)
0.00981	0.00083	0.90299	0.82212	(Right-Putamen)(rh.fusiform_7)(rh.insula_4)(rh.precuneus_2)
0.00981	0.00091	0.90299	0.82212	(Right-Caudate)(Right-Thalamus-Proper)(rh.fusiform_7)(rh.supramarginal_9)
0.00981	0.001	0.90299	0.82212	(Right-Pallidum)(rh.fusiform_7)(rh.insula_4)(rh.precuneus_2)
0.00981	0.00111	0.90299	0.82212	(rh.fusiform_7)(rh.insula_4)(rh.isthmuscingulate_2)(rh.precuneus_2)

0.00981	0.00125	0.90299	0.82212	(rh.fusiform_7)(rh.insula_5)(rh.precuneus_2)(rh.superiortemporal_9)
0.00981	0.00143	0.90299	0.82212	(Right-Caudate)(Right-Putamen)(rh.fusiform_7)(rh.supramarginal_9)
0.00981	0.00167	0.90299	0.82212	(Right-Caudate)(rh.fusiform_7)(rh.insula_2)(rh.supramarginal_9)
0.00981	0.002	0.90299	0.82212	(rh.fusiform_7)(rh.insula_4)(rh.precuneus_2)
0.00991	0.0025	0.99254	0.95673	(rh.bankssts_2)(rh.lingual_7)(rh.superiortemporal_3)
0.00991	0.00333	0.99254	0.95673	(rh.bankssts_2)(rh.isthmuscingulate_2)(rh.lingual_7)(rh.superiortemporal_3)
0.00991	0.005	0.99254	0.95673	(Right-Thalamus-Proper)(rh.bankssts_2)(rh.lingual_7)(rh.superiortemporal_3)
0.00991	0.01	0.99254	0.95673	(Right-Putamen)(rh.bankssts_2)(rh.lingual_7)(rh.superiortemporal_3)

al_3)

-al_3)

9)

)

al_3)

9)

.9)

l_3)

)

_3)
al_3)

al_3)

·al_3)

al_3)

9)

3)

[illegible])

al_3)

9)

al_9)

ıal_3)

al_3)

al_3)

al_3)

9)

)

al_2)
