# Supplementary material for: Good neighbors, bad neighbors: the frequent network neighborhood mapping of the hippocampus enlightens several structural factors of the human intelligence on a 414-subject cohort
Source: Sci Rep. 2020 Jul 20;10:11967. doi: 10.1038/s41598-020-68914-2 (PMC7371878; doi:10.1038/s41598-020-68914-2)
Supplement: Supplementary file 4 — Supplementary Information 4. [file 41598_2020_68914_MOESM4_ESM.pdf]

p-value	Holm-Bonferroni	frequency_upper	frequency_lower	name
0.00236	1.00E-05	0.71831	0.83654	(rh.inferiorparietal_4)(rh.middletemporal_1)(rh.parahippocampal_1)(rh.precuneus_3)
0.00286	1.00E-05	0.73239	0.84615	(rh.middletemporal_1)(rh.parahippocampal_1)(rh.precuneus_3)
0.0033	1.00E-05	0.71127	0.82692	(rh.inferiorparietal_10)(rh.middletemporal_1)(rh.parahippocampal_1)(rh.precuneus_3)
0.00442	1.00E-05	0.73239	0.84135	(rh.lingual_7)(rh.middletemporal_1)(rh.parahippocampal_1)(rh.precuneus_3)
0.00455	1.00E-05	0.77985	0.87981	(rh.inferiorparietal_4)(rh.lingual_5)(rh.middletemporal_1)
0.00455	1.00E-05	0.77985	0.87981	(Right-Thalamus-Proper)(rh.inferiorparietal_4)(rh.lingual_5)(rh.middletemporal_1)
0.00455	1.00E-05	0.77985	0.87981	(Right-Putamen)(rh.inferiorparietal_4)(rh.lingual_5)(rh.middletemporal_1)
0.00455	1.00E-05	0.77985	0.87981	(rh.inferiorparietal_4)(rh.isthmuscingulate_2)(rh.lingual_5)(rh.middletemporal_1)
0.00484	1.00E-05	0.77465	0.875	(rh.inferiorparietal_4)(rh.middletemporal_1)(rh.precuneus_3)(rh.superiortemporal_1)
0.00552	1.00E-05	0.71831	0.82692	(rh.middletemporal_1)(rh.parahippocampal_1)(rh.precuneus_3)(rh.supramarginal_9)
0.00567	1.00E-05	0.78873	0.88462	(rh.inferiorparietal_10)(rh.inferiorparietal_4)(rh.middletemporal_1)(rh.precuneus_3)
0.0057	1.00E-05	0.76493	0.86538	(Right-Putamen)(rh.lingual_5)(rh.middletemporal_1)(rh.precuneus_3)
0.0057	1.00E-05	0.76493	0.86538	(rh.isthmuscingulate_2)(rh.lingual_5)(rh.middletemporal_1)(rh.precuneus_3)
0.0057	1.00E-05	0.76493	0.86538	(Right-Thalamus-Proper)(rh.lingual_5)(rh.middletemporal_1)(rh.precuneus_3)
0.00592	1.00E-05	0.75373	0.85577	(Right-Pallidum)(rh.lingual_5)(rh.middletemporal_1)(rh.precuneus_3)
0.00637	1.00E-05	0.76056	0.86058	(rh.inferiorparietal_4)(rh.lingual_5)(rh.middletemporal_1)(rh.precuneus_3)
0.0064	1.00E-05	0.77239	0.87019	(Right-Pallidum)(rh.inferiorparietal_4)(rh.lingual_5)(rh.middletemporal_1)
0.00704	2.00E-05	0.73881	0.84135	(rh.lingual_5)(rh.middletemporal_1)(rh.precuneus_3)(rh.superiortemporal_1)
0.00718	2.00E-05	0.77985	0.875	(rh.bankssts_2)(rh.inferiorparietal_4)(rh.lingual_5)(rh.middletemporal_1)
0.00718	2.00E-05	0.77985	0.875	(rh.inferiorparietal_4)(rh.lingual_5)(rh.lingual_7)(rh.middletemporal_1)
0.00737	2.00E-05	0.73944	0.84135	(rh.inferiorparietal_10)(rh.inferiorparietal_4)(rh.middletemporal_1)(rh.parahippocampal_
0.00751	2.00E-05	0.76866	0.86538	(Right-Caudate)(rh.inferiorparietal_4)(rh.lingual_5)(rh.middletemporal_1)
0.00816	2.00E-05	0.72388	0.82692	(rh.lingual_5)(rh.middletemporal_1)(rh.superiortemporal_1)(rh.superiortemporal_9)
0.00823	2.00E-05	0.78169	0.875	(rh.inferiorparietal_10)(rh.parahippocampal_1)(rh.precuneus_3)(rh.superiortemporal_1)
0.00876	3.00E-05	0.76493	0.86058	(rh.bankssts_2)(rh.lingual_5)(rh.middletemporal_1)(rh.precuneus_3)
0.00876	3.00E-05	0.76493	0.86058	(rh.lingual_5)(rh.lingual_7)(rh.middletemporal_1)(rh.precuneus_3)
0.00893	3.00E-05	0.78873	0.87981	(rh.insula_2)(rh.parahippocampal_1)(rh.precuneus_3)(rh.superiortemporal_1)
0.00893	3.00E-05	0.78873	0.87981	(rh.inferiorparietal_4)(rh.parahippocampal_1)(rh.precuneus_3)(rh.superiortemporal_1)
0.009	3.00E-05	0.75373	0.85096	(Right-Caudate)(rh.lingual_5)(rh.middletemporal_1)(rh.precuneus_3)
0.00966	7.00E-05	0.79577	0.88462	(Right-Pallidum)(rh.parahippocampal_1)(rh.precuneus_3)(rh.superiortemporal_1)
0.0097	7.00E-05	0.76056	0.85577	(rh.inferiorparietal_4)(rh.middletemporal_1)(rh.parahippocampal_1)

- 1)
