# Supplementary material for: Good neighbors, bad neighbors: the frequent network neighborhood mapping of the hippocampus enlightens several structural factors of the human intelligence on a 414-subject cohort
Source: Sci Rep. 2020 Jul 20;10:11967. doi: 10.1038/s41598-020-68914-2 (PMC7371878; doi:10.1038/s41598-020-68914-2)
Supplement: Supplementary file 5 — Supplementary Information 5. [file 41598_2020_68914_MOESM5_ESM.pdf]

p-value	Holm-Bonferroni	frequency_upper	frequency_lower	name
5.00E-05	0	0.97388	0.87981	(lh.inferiorparietal_5)(lh.isthmuscingulate_2)(rh.precuneus_2)(rh.superiortemporal_3)
9.00E-05	0	0.91791	0.79327	(lh.isthmuscingulate_2)(rh.bankssts_2)(rh.fusiform_7)(rh.precuneus_2)
9.00E-05	0	0.91791	0.79327	(lh.isthmuscingulate_2)(rh.fusiform_7)(rh.lingual_7)(rh.precuneus_2)
9.00E-05	0	0.91418	0.78846	(lh.isthmuscingulate_2)(rh.fusiform_7)(rh.precuneus_2)(rh.superiortemporal_3)
0.00011	0	0.94776	0.84135	(Left-Caudate)(lh.inferiorparietal_5)(lh.superiortemporal_3)(rh.precuneus_2)
0.00011	0	0.98507	0.90865	(lh.isthmuscingulate_2)(rh.lingual_7)(rh.precuneus_2)(rh.superiortemporal_3)
0.00011	0	0.98507	0.90865	(lh.isthmuscingulate_2)(rh.bankssts_2)(rh.precuneus_2)(rh.superiortemporal_3)
0.00012	0	0.92537	0.80769	(Left-Caudate)(lh.isthmuscingulate_2)(lh.transversetemporal_2)(rh.superiortemporal_3)
0.00013	0	0.94403	0.83654	(Left-Caudate)(lh.inferiorparietal_5)(lh.isthmuscingulate_2)(rh.superiortemporal_3)
0.00013	0	0.94403	0.83654	(Left-Caudate)(lh.inferiorparietal_5)(lh.isthmuscingulate_2)(lh.superiortemporal_3)
0.00014	0	0.91791	0.79808	(lh.isthmuscingulate_2)(rh.fusiform_7)(rh.insula_2)(rh.precuneus_2)
0.00014	0	0.91791	0.79808	(lh.isthmuscingulate_2)(lh.superiortemporal_2)(rh.fusiform_7)(rh.precuneus_2)
0.00014	0	0.91791	0.79808	(lh.isthmuscingulate_2)(lh.isthmuscingulate_3)(rh.fusiform_7)(rh.precuneus_2)
0.00014	0	0.91791	0.79808	(Right-Thalamus-Proper)(lh.isthmuscingulate_2)(rh.fusiform_7)(rh.precuneus_2)
0.00014	0	0.91791	0.79808	(lh.isthmuscingulate_2)(rh.fusiform_7)(rh.isthmuscingulate_2)(rh.precuneus_2)
0.00014	0	0.91791	0.79808	(lh.isthmuscingulate_2)(rh.fusiform_7)(rh.precuneus_2)
0.00014	0	0.91791	0.79808	(Right-Putamen)(lh.isthmuscingulate_2)(rh.fusiform_7)(rh.precuneus_2)
0.00014	0	0.91791	0.79808	(Left-Thalamus-Proper)(lh.isthmuscingulate_2)(rh.fusiform_7)(rh.precuneus_2)
0.00014	0	0.91791	0.79808	(Left-Putamen)(lh.isthmuscingulate_2)(rh.fusiform_7)(rh.precuneus_2)
0.00015	0	0.95522	0.85577	(Left-Caudate)(lh.isthmuscingulate_2)(rh.parahippocampal_2)(rh.precuneus_2)
0.00015	0	0.95522	0.85577	(Left-Caudate)(lh.isthmuscingulate_2)(rh.insula_2)(rh.precuneus_2)
0.00017	0	0.97388	0.88942	(lh.isthmuscingulate_2)(rh.parahippocampal_2)(rh.precuneus_2)(rh.superiortemporal_3)
0.00017	0	0.98134	0.90385	(Left-Putamen)(lh.inferiorparietal_5)(lh.isthmuscingulate_2)(rh.precuneus_2)
0.00019	0	0.96269	0.87019	(Left-Caudate)(lh.isthmuscingulate_2)(rh.bankssts_2)(rh.precuneus_2)
0.00019	0	0.9291	0.81731	(Left-Caudate)(lh.superiortemporal_3)(lh.transversetemporal_2)(rh.precuneus_2)
0.00021	0	0.92537	0.8125	(Left-Caudate)(lh.isthmuscingulate_2)(lh.superiortemporal_3)(lh.transversetemporal_2)
0.00022	0	0.97015	0.88462	(lh.inferiorparietal_5)(lh.isthmuscingulate_2)(lh.superiortemporal_3)(rh.precuneus_2)
0.00022	0	0.95896	0.86538	(Left-Caudate)(lh.superiortemporal_3)(rh.bankssts_2)(rh.precuneus_2)
0.00022	0	0.98507	0.91346	(Right-Putamen)(lh.isthmuscingulate_2)(rh.precuneus_2)(rh.superiortemporal_3)
0.00022	0	0.98507	0.91346	(lh.isthmuscingulate_2)(rh.precuneus_2)(rh.superiortemporal_3)
0.00022	0	0.98507	0.91346	(Left-Putamen)(lh.isthmuscingulate_2)(rh.precuneus_2)(rh.superiortemporal_3)
0.00022	0	0.98507	0.91346	(Right-Thalamus-Proper)(lh.isthmuscingulate_2)(rh.precuneus_2)(rh.superiortemporal_3)
0.00022	0	0.98507	0.91346	(Left-Thalamus-Proper)(lh.isthmuscingulate_2)(rh.precuneus_2)(rh.superiortemporal_3)

0.00022	0	0.98507	0.91346	(lh.isthmuscingulate_2)(lh.superiortemporal_2)(rh.precuneus_2)(rh.superiortemporal_3)
0.00022	0	0.98507	0.91346	(lh.isthmuscingulate_2)(lh.isthmuscingulate_3)(rh.precuneus_2)(rh.superiortemporal_3)
0.00022	0	0.98507	0.91346	(lh.isthmuscingulate_2)(rh.isthmuscingulate_2)(rh.precuneus_2)(rh.superiortemporal_3)
0.00022	0	0.94403	0.84135	(Left-Caudate)(lh.inferiorparietal_5)(lh.superiortemporal_3)(rh.parahippocampal_2)
0.00026	0	0.95522	0.86058	(Left-Caudate)(Left-Putamen)(lh.isthmuscingulate_2)(lh.superiortemporal_3)
0.00028	0	0.93657	0.83173	(Left-Caudate)(lh.inferiorparietal_5)(lh.isthmuscingulate_2)(lh.parahippocampal_3)
0.00028	0	0.93657	0.83173	(Left-Caudate)(lh.fusiform_7)(lh.superiortemporal_2)(rh.precuneus_2)
0.00028	0	0.90672	0.78846	(rh.inferiorparietal_9)(rh.lingual_7)(rh.precuneus_2)(rh.superiortemporal_3)
0.00028	0	0.90672	0.78846	(rh.bankssts_2)(rh.inferiorparietal_9)(rh.precuneus_2)(rh.superiortemporal_3)
0.00028	0	0.90672	0.78846	(lh.superiortemporal_2)(rh.inferiorparietal_9)(rh.precuneus_2)(rh.superiortemporal_3)
0.00029	0	0.90299	0.78365	(lh.isthmuscingulate_2)(lh.parahippocampal_3)(rh.fusiform_7)(rh.precuneus_2)
0.0003	0	0.93284	0.82692	(Left-Caudate)(lh.fusiform_7)(lh.isthmuscingulate_2)(rh.isthmuscingulate_2)
0.0003	0	0.93284	0.82692	(Left-Caudate)(lh.fusiform_7)(lh.isthmuscingulate_2)(lh.superiortemporal_2)
0.0003	0	0.93284	0.82692	(Left-Caudate)(Left-Putamen)(lh.fusiform_7)(lh.isthmuscingulate_2)
0.0003	0	0.93284	0.82692	(Left-Caudate)(Left-Thalamus-Proper)(lh.fusiform_7)(lh.isthmuscingulate_2)
0.0003	0	0.95149	0.85577	(Left-Caudate)(Left-Putamen)(lh.inferiorparietal_5)(lh.superiortemporal_3)
0.0003	0	0.95149	0.85577	(Left-Caudate)(Left-Putamen)(lh.inferiorparietal_5)(lh.isthmuscingulate_2)
0.0003	0	0.95149	0.85577	(Left-Caudate)(rh.parahippocampal_2)(rh.precuneus_2)(rh.superiortemporal_3)
0.0003	0	0.95149	0.85577	(Left-Caudate)(lh.inferiorparietal_5)(lh.superiortemporal_3)(rh.bankssts_2)
0.00031	0	0.97388	0.89423	(lh.inferiorparietal_5)(lh.isthmuscingulate_2)(rh.insula_2)(rh.precuneus_2)
0.00032	0	0.98134	0.90865	(lh.inferiorparietal_5)(lh.isthmuscingulate_2)(rh.lingual_7)(rh.precuneus_2)
0.00032	0	0.98134	0.90865	(lh.isthmuscingulate_2)(rh.insula_2)(rh.precuneus_2)(rh.superiortemporal_3)
0.00032	0	0.98134	0.90865	(lh.inferiorparietal_5)(lh.isthmuscingulate_2)(rh.bankssts_2)(rh.precuneus_2)
0.00033	0	0.9291	0.82212	(Left-Caudate)(lh.superiortemporal_3)(lh.transversetemporal_2)(rh.superiortemporal_3)
0.00033	0	0.9291	0.82212	(Left-Caudate)(lh.fusiform_7)(lh.isthmuscingulate_2)(rh.superiortemporal_3)
0.00033	0	0.99254	0.93269	(Left-Putamen)(lh.isthmuscingulate_2)(rh.bankssts_2)(rh.precuneus_2)
0.00033	0	0.99254	0.93269	(Left-Putamen)(lh.isthmuscingulate_2)(rh.lingual_7)(rh.precuneus_2)
0.00033	0	0.96269	0.875	(lh.fusiform_7)(lh.isthmuscingulate_2)(rh.bankssts_2)(rh.precuneus_2)
0.00033	0	0.96269	0.875	(Left-Caudate)(lh.isthmuscingulate_2)(lh.superiortemporal_2)(rh.precuneus_2)
0.00033	0	0.96269	0.875	(Left-Caudate)(lh.isthmuscingulate_2)(lh.isthmuscingulate_3)(rh.precuneus_2)
0.00033	0	0.96269	0.875	(lh.fusiform_7)(lh.isthmuscingulate_2)(rh.lingual_7)(rh.precuneus_2)
0.00033	0	0.96269	0.875	(Left-Caudate)(lh.isthmuscingulate_2)(rh.precuneus_2)
0.00033	0	0.96269	0.875	(Left-Caudate)(Right-Putamen)(lh.isthmuscingulate_2)(rh.precuneus_2)
0.00033	0	0.96269	0.875	(Left-Caudate)(lh.isthmuscingulate_2)(rh.isthmuscingulate_2)(rh.precuneus_2)
