# Supplementary material for: Good neighbors, bad neighbors: the frequent network neighborhood mapping of the hippocampus enlightens several structural factors of the human intelligence on a 414-subject cohort
Source: Sci Rep. 2020 Jul 20;10:11967. doi: 10.1038/s41598-020-68914-2 (PMC7371878; doi:10.1038/s41598-020-68914-2)
Supplement: Supplementary file 6 — Supplementary Information 6. [file 41598_2020_68914_MOESM6_ESM.pdf]

p-value	Holm-Bonferroni	frequency_upper	frequency_lower	name
0.00131	0	0.89437	0.97115	(lh.bankssts_3)(lh.fusiform_5)(lh.isthmuscingulate_3)(rh.precuneus_3)
0.00131	0	0.89437	0.97115	(Right-Thalamus-Proper)(lh.bankssts_3)(lh.fusiform_5)(rh.precuneus_3)
0.00131	0	0.89437	0.97115	(lh.bankssts_3)(lh.fusiform_5)(rh.precuneus_3)
0.00131	0	0.89437	0.97115	(lh.bankssts_3)(lh.fusiform_5)(rh.lingual_7)(rh.precuneus_3)
0.00131	0	0.89437	0.97115	(Right-Putamen)(lh.bankssts_3)(lh.fusiform_5)(rh.precuneus_3)
0.00296	0	0.89437	0.96635	(lh.bankssts_3)(lh.fusiform_5)(rh.bankssts_2)(rh.precuneus_3)
0.00296	0	0.89437	0.96635	(Right-Thalamus-Proper)(lh.bankssts_3)(lh.fusiform_5)(lh.insula_1)
0.00296	0	0.89437	0.96635	(Right-Putamen)(lh.bankssts_3)(lh.fusiform_5)(lh.insula_1)
0.00296	0	0.89437	0.96635	(lh.bankssts_3)(lh.fusiform_5)(lh.insula_1)(lh.isthmuscingulate_3)
0.00296	0	0.89437	0.96635	(lh.bankssts_3)(lh.fusiform_5)(lh.insula_1)
0.00296	0	0.89437	0.96635	(lh.bankssts_3)(lh.fusiform_5)(lh.insula_1)(rh.lingual_7)
0.00311	0	0.88732	0.96154	(Right-Pallidum)(lh.bankssts_3)(lh.fusiform_5)(rh.precuneus_3)
0.00328	0	0.87324	0.95192	(Right-Thalamus-Proper)(lh.bankssts_3)(lh.insula_1)(rh.precuneus_3)
0.00328	0	0.87324	0.95192	(lh.bankssts_3)(lh.insula_1)(rh.lingual_7)(rh.precuneus_3)
0.00328	0	0.87324	0.95192	(lh.bankssts_3)(lh.fusiform_5)(lh.insula_1)(lh.lingual_8)
0.00328	0	0.87324	0.95192	(Right-Putamen)(lh.bankssts_3)(lh.insula_1)(rh.precuneus_3)
0.00328	0	0.87324	0.95192	(lh.bankssts_3)(lh.insula_1)(lh.isthmuscingulate_3)(rh.precuneus_3)
0.00328	0	0.87324	0.95192	(lh.bankssts_3)(lh.insula_1)(rh.precuneus_3)
0.00328	0	0.87324	0.95192	(lh.bankssts_3)(lh.fusiform_5)(lh.insula_1)(rh.inferiorparietal_4)
0.00617	0	0.8662	0.94231	(lh.bankssts_3)(lh.insula_1)(lh.lingual_8)(rh.inferiorparietal_4)
0.00617	0	0.8662	0.94231	(Right-Pallidum)(lh.bankssts_3)(lh.insula_1)(rh.precuneus_3)
0.00617	0	0.89437	0.96154	(Right-Caudate)(lh.bankssts_3)(lh.fusiform_5)(rh.precuneus_3)
0.00617	0	0.89437	0.96154	(lh.bankssts_3)(lh.fusiform_5)(lh.lingual_8)(rh.inferiorparietal_4)
0.00617	0	0.89437	0.96154	(lh.bankssts_3)(lh.fusiform_5)(lh.insula_1)(rh.bankssts_2)
0.00627	0	0.87324	0.94712	(lh.bankssts_3)(lh.insula_1)(rh.bankssts_2)(rh.precuneus_3)
0.00629	1.00E-05	0.88732	0.95673	(Right-Thalamus-Proper)(lh.bankssts_3)(lh.lingual_8)(rh.precuneus_3)
0.00629	1.00E-05	0.88732	0.95673	(lh.bankssts_3)(lh.lingual_8)(rh.lingual_7)(rh.precuneus_3)
0.00629	1.00E-05	0.88732	0.95673	(lh.bankssts_3)(lh.lingual_8)(rh.precuneus_3)
0.00629	1.00E-05	0.88732	0.95673	(Right-Pallidum)(lh.bankssts_3)(lh.fusiform_5)(lh.insula_1)
0.00629	1.00E-05	0.88732	0.95673	(Right-Putamen)(lh.bankssts_3)(lh.lingual_8)(rh.precuneus_3)
0.00629	1.00E-05	0.88732	0.95673	(lh.bankssts_3)(rh.inferiorparietal_4)(rh.precuneus_3)
0.00629	1.00E-05	0.88732	0.95673	(lh.bankssts_3)(lh.isthmuscingulate_3)(lh.lingual_8)(rh.precuneus_3)
