# Supplementary material for: Good neighbors, bad neighbors: the frequent network neighborhood mapping of the hippocampus enlightens several structural factors of the human intelligence on a 414-subject cohort
Source: Sci Rep. 2020 Jul 20;10:11967. doi: 10.1038/s41598-020-68914-2 (PMC7371878; doi:10.1038/s41598-020-68914-2)
Supplement: Supplementary file 7 — Supplementary Information 7. [file 41598_2020_68914_MOESM7_ESM.pdf]

p-value	Holm-Bonferroni	frequency_upper	frequency_lower	name
