# Supplementary material for: Good neighbors, bad neighbors: the frequent network neighborhood mapping of the hippocampus enlightens several structural factors of the human intelligence on a 414-subject cohort
Source: Sci Rep. 2020 Jul 20;10:11967. doi: 10.1038/s41598-020-68914-2 (PMC7371878; doi:10.1038/s41598-020-68914-2)
Supplement: Supplementary file 8 — Supplementary Information 8. [file 41598_2020_68914_MOESM8_ESM.pdf]

p-value	Holm-Bonferroni	frequency_upper	frequency_lower	name
4.00E-05	1.00E-05	0.6787	0.84422	(lh.inferiorparietal_4)(lh.inferiorparietal_5)(lh.precuneus_11)(lh.supramarginal_1)
5.00E-05	1.00E-05	0.67509	0.8392	(lh.inferiorparietal_4)(lh.precuneus_11)(lh.superiortemporal_3)(lh.supramarginal_1)
6.00E-05	1.00E-05	0.68231	0.84422	(lh.inferiorparietal_4)(lh.insula_2)(lh.precuneus_11)(lh.supramarginal_1)
6.00E-05	1.00E-05	0.66426	0.82915	(Left-Pallidum)(lh.inferiorparietal_4)(lh.precuneus_11)(lh.supramarginal_1)
6.00E-05	1.00E-05	0.68953	0.84925	(Left-Putamen)(lh.inferiorparietal_4)(lh.precuneus_11)(lh.supramarginal_1)
9.00E-05	1.00E-05	0.69314	0.84925	(Left-Thalamus-Proper)(lh.inferiorparietal_4)(lh.precuneus_11)(lh.supramarginal_1)
9.00E-05	1.00E-05	0.69314	0.84925	(lh.inferiorparietal_4)(lh.precuneus_11)(lh.supramarginal_1)
9.00E-05	1.00E-05	0.69314	0.84925	(lh.inferiorparietal_4)(lh.precuneus_11)(lh.superiortemporal_2)(lh.supramarginal_1)
9.00E-05	1.00E-05	0.69314	0.84925	(lh.inferiorparietal_4)(lh.isthmuscingulate_3)(lh.precuneus_11)(lh.supramarginal_1)
9.00E-05	1.00E-05	0.67509	0.83417	(lh.inferiorparietal_4)(lh.inferiorparietal_5)(lh.superiortemporal_3)(lh.supramarginal_1)
0.0001	1.00E-05	0.68231	0.8392	(lh.inferiorparietal_4)(lh.inferiorparietal_5)(lh.insula_2)(lh.supramarginal_1)
0.0001	1.00E-05	0.68231	0.8392	(lh.inferiorparietal_4)(lh.lingual_8)(lh.precuneus_11)(lh.supramarginal_1)
0.0001	1.00E-05	0.68231	0.8392	(lh.inferiorparietal_4)(lh.lingual_6)(lh.precuneus_11)(lh.supramarginal_1)
0.0001	1.00E-05	0.66426	0.82412	(lh.inferiorparietal_4)(lh.precuneus_11)(lh.supramarginal_1)(lh.transversetemporal_2)
0.0001	1.00E-05	0.66426	0.82412	(Left-Pallidum)(lh.inferiorparietal_4)(lh.inferiorparietal_5)(lh.supramarginal_1)
0.00011	1.00E-05	0.68953	0.84422	(Left-Putamen)(lh.inferiorparietal_4)(lh.inferiorparietal_5)(lh.supramarginal_1)
0.00015	1.00E-05	0.69314	0.84422	(lh.inferiorparietal_4)(lh.inferiorparietal_5)(lh.superiortemporal_2)(lh.supramarginal_1)
0.00015	1.00E-05	0.69314	0.84422	(lh.inferiorparietal_4)(lh.inferiorparietal_5)(lh.supramarginal_1)
0.00015	1.00E-05	0.69314	0.84422	(Left-Thalamus-Proper)(lh.inferiorparietal_4)(lh.inferiorparietal_5)(lh.supramarginal_1)
0.00015	1.00E-05	0.69314	0.84422	(lh.inferiorparietal_4)(lh.inferiorparietal_5)(lh.isthmuscingulate_3)(lh.supramarginal_1)
0.00016	1.00E-05	0.67509	0.82915	(Left-Pallidum)(lh.inferiorparietal_4)(lh.insula_2)(lh.supramarginal_1)
0.00016	1.00E-05	0.67509	0.82915	(lh.bankssts_3)(lh.inferiorparietal_4)(lh.precuneus_11)(lh.supramarginal_1)
0.00017	1.00E-05	0.68231	0.83417	(lh.inferiorparietal_4)(lh.isthmuscingulate_2)(lh.precuneus_11)(lh.supramarginal_1)
0.00017	1.00E-05	0.68231	0.83417	(lh.inferiorparietal_4)(lh.inferiorparietal_5)(lh.lingual_6)(lh.supramarginal_1)
0.00017	1.00E-05	0.68231	0.83417	(lh.inferiorparietal_4)(lh.inferiorparietal_5)(lh.lingual_8)(lh.supramarginal_1)
0.00018	1.00E-05	0.66426	0.8191	(lh.inferiorparietal_4)(lh.inferiorparietal_5)(lh.supramarginal_1)(lh.transversetemporal_2)
0.00019	1.00E-05	0.68953	0.8392	(lh.inferiorparietal_4)(lh.superiortemporal_2)(lh.superiortemporal_3)(lh.supramarginal_1)
0.00019	1.00E-05	0.68953	0.8392	(Left-Thalamus-Proper)(lh.inferiorparietal_4)(lh.superiortemporal_3)(lh.supramarginal_1)
0.00019	1.00E-05	0.68953	0.8392	(lh.inferiorparietal_4)(lh.isthmuscingulate_3)(lh.superiortemporal_3)(lh.supramarginal_1)
0.00019	1.00E-05	0.68953	0.8392	(lh.fusiform_5)(lh.inferiorparietal_4)(lh.precuneus_11)(lh.supramarginal_1)
0.00019	1.00E-05	0.68953	0.8392	(Left-Putamen)(lh.inferiorparietal_4)(lh.superiortemporal_3)(lh.supramarginal_1)
0.00019	1.00E-05	0.68953	0.8392	(lh.inferiorparietal_4)(lh.superiortemporal_3)(lh.supramarginal_1)
0.0002	1.00E-05	0.67148	0.82412	(lh.inferiorparietal_4)(lh.parahippocampal_3)(lh.precuneus_11)(lh.supramarginal_1)
0.00021	1.00E-05	0.69675	0.84422	(lh.inferiorparietal_4)(lh.insula_2)(lh.supramarginal_1)
0.00021	1.00E-05	0.69675	0.84422	(Left-Putamen)(lh.inferiorparietal_4)(lh.insula_2)(lh.supramarginal_1)

0.00021	1.00E-05	0.69675	0.84422	(lh.inferiorparietal_4)(lh.insula_2)(lh.superiortemporal_2)(lh.supramarginal_1)
0.00021	1.00E-05	0.69675	0.84422	(Left-Thalamus-Proper)(lh.inferiorparietal_4)(lh.insula_2)(lh.supramarginal_1)
0.00021	1.00E-05	0.69675	0.84422	(lh.inferiorparietal_4)(lh.insula_2)(lh.isthmuscingulate_3)(lh.supramarginal_1)
0.00022	1.00E-05	0.6787	0.82915	(lh.inferiorparietal_4)(lh.lingual_8)(lh.superiortemporal_3)(lh.supramarginal_1)
0.00022	1.00E-05	0.6787	0.82915	(Left-Pallidum)(lh.inferiorparietal_4)(lh.supramarginal_1)
0.00022	1.00E-05	0.6787	0.82915	(Left-Pallidum)(lh.inferiorparietal_4)(lh.isthmuscingulate_3)(lh.supramarginal_1)
0.00022	1.00E-05	0.6787	0.82915	(lh.inferiorparietal_4)(lh.lingual_6)(lh.superiortemporal_3)(lh.supramarginal_1)
0.00022	1.00E-05	0.6787	0.82915	(Left-Pallidum)(lh.inferiorparietal_4)(lh.superiortemporal_2)(lh.supramarginal_1)
0.00022	1.00E-05	0.6787	0.82915	(Left-Pallidum)(Left-Putamen)(lh.inferiorparietal_4)(lh.supramarginal_1)
0.00022	1.00E-05	0.6787	0.82915	(Left-Pallidum)(Left-Thalamus-Proper)(lh.inferiorparietal_4)(lh.supramarginal_1)
0.00023	1.00E-05	0.70397	0.84925	(Left-Putamen)(Left-Thalamus-Proper)(lh.inferiorparietal_4)(lh.supramarginal_1)
0.00023	1.00E-05	0.70397	0.84925	(Left-Putamen)(lh.inferiorparietal_4)(lh.isthmuscingulate_3)(lh.supramarginal_1)
0.00023	1.00E-05	0.70397	0.84925	(Left-Putamen)(lh.inferiorparietal_4)(lh.superiortemporal_2)(lh.supramarginal_1)
0.00023	1.00E-05	0.70397	0.84925	(Left-Putamen)(lh.inferiorparietal_4)(lh.supramarginal_1)
0.00024	1.00E-05	0.68592	0.83417	(lh.inferiorparietal_4)(lh.insula_2)(lh.lingual_8)(lh.supramarginal_1)
0.00024	1.00E-05	0.68592	0.83417	(lh.inferiorparietal_4)(lh.insula_2)(lh.lingual_6)(lh.supramarginal_1)
0.00024	1.00E-05	0.68592	0.83417	(lh.inferiorparietal_4)(lh.insula_2)(lh.superiortemporal_3)(lh.supramarginal_1)
0.00024	1.00E-05	0.66787	0.8191	(Left-Pallidum)(lh.inferiorparietal_4)(lh.lingual_8)(lh.supramarginal_1)
0.00024	1.00E-05	0.66787	0.8191	(Left-Pallidum)(lh.inferiorparietal_4)(lh.lingual_6)(lh.supramarginal_1)
0.00026	1.00E-05	0.69314	0.8392	(Left-Putamen)(lh.inferiorparietal_4)(lh.lingual_6)(lh.supramarginal_1)
0.00026	1.00E-05	0.69314	0.8392	(Left-Putamen)(lh.inferiorparietal_4)(lh.lingual_8)(lh.supramarginal_1)
0.00027	1.00E-05	0.65704	0.80905	(Left-Caudate)(lh.inferiorparietal_4)(lh.precuneus_11)(lh.supramarginal_1)
0.00027	1.00E-05	0.67509	0.82412	(lh.bankssts_3)(lh.inferiorparietal_4)(lh.inferiorparietal_5)(lh.supramarginal_1)
0.00027	1.00E-05	0.67509	0.82412	(Left-Putamen)(lh.inferiorparietal_4)(lh.supramarginal_1)(lh.transversetemporal_2)
0.0003	1.00E-05	0.68231	0.82915	(lh.inferiorparietal_4)(lh.inferiorparietal_5)(lh.isthmuscingulate_2)(lh.supramarginal_1)
0.00031	1.00E-05	0.70758	0.84925	(Left-Thalamus-Proper)(lh.inferiorparietal_4)(lh.superiortemporal_2)(lh.supramarginal_1)
0.00031	1.00E-05	0.70758	0.84925	(lh.inferiorparietal_4)(lh.superiortemporal_2)(lh.supramarginal_1)
0.00031	1.00E-05	0.70758	0.84925	(lh.inferiorparietal_4)(lh.isthmuscingulate_3)(lh.superiortemporal_2)(lh.supramarginal_1)
0.00031	1.00E-05	0.70758	0.84925	(Left-Thalamus-Proper)(lh.inferiorparietal_4)(lh.isthmuscingulate_3)(lh.supramarginal_1)
0.00031	1.00E-05	0.70758	0.84925	(Left-Thalamus-Proper)(lh.inferiorparietal_4)(lh.supramarginal_1)
0.00032	1.00E-05	0.68953	0.83417	(lh.fusiform_5)(lh.inferiorparietal_4)(lh.inferiorparietal_5)(lh.supramarginal_1)
0.00033	1.00E-05	0.67148	0.8191	(Left-Pallidum)(lh.inferiorparietal_4)(lh.superiortemporal_3)(lh.supramarginal_1)
0.00033	1.00E-05	0.67148	0.8191	(lh.inferiorparietal_4)(lh.inferiorparietal_5)(lh.parahippocampal_3)(lh.supramarginal_1)
0.00033	1.00E-05	0.67148	0.8191	(lh.bankssts_3)(lh.inferiorparietal_4)(lh.superiortemporal_3)(lh.supramarginal_1)
0.00035	1.00E-05	0.79422	0.91457	(lh.inferiorparietal_4)(lh.inferiorparietal_5)(lh.lingual_6)(lh.precuneus_11)
0.00036	1.00E-05	0.69675	0.8392	(lh.inferiorparietal_4)(lh.lingual_8)(lh.superiortemporal_2)(lh.supramarginal_1)

0.00036	1.00E-05	0.69675	0.8392	(lh.inferiorparietal_4)(lh.lingual_6)(lh.supramarginal_1)
0.00036	1.00E-05	0.69675	0.8392	(lh.inferiorparietal_4)(lh.isthmuscingulate_3)(lh.lingual_6)(lh.supramarginal_1)
0.00036	1.00E-05	0.69675	0.8392	(Left-Thalamus-Proper)(lh.inferiorparietal_4)(lh.lingual_8)(lh.supramarginal_1)
0.00036	1.00E-05	0.69675	0.8392	(lh.inferiorparietal_4)(lh.isthmuscingulate_3)(lh.lingual_8)(lh.supramarginal_1)
0.00036	1.00E-05	0.69675	0.8392	(Left-Thalamus-Proper)(lh.inferiorparietal_4)(lh.lingual_6)(lh.supramarginal_1)
0.00036	1.00E-05	0.69675	0.8392	(lh.inferiorparietal_4)(lh.lingual_6)(lh.superiortemporal_2)(lh.supramarginal_1)
0.00036	1.00E-05	0.69675	0.8392	(lh.inferiorparietal_4)(lh.lingual_8)(lh.supramarginal_1)
0.00036	1.00E-05	0.80144	0.9196	(lh.inferiorparietal_4)(lh.inferiorparietal_5)(lh.insula_2)(lh.precuneus_11)
0.00036	1.00E-05	0.66065	0.80905	(Left-Pallidum)(lh.bankssts_3)(lh.inferiorparietal_4)(lh.supramarginal_1)
0.00036	1.00E-05	0.80866	0.92462	(Left-Putamen)(lh.inferiorparietal_4)(lh.inferiorparietal_5)(lh.precuneus_11)
0.00036	1.00E-05	0.6787	0.82412	(lh.inferiorparietal_4)(lh.isthmuscingulate_2)(lh.superiortemporal_3)(lh.supramarginal_1)
0.00036	1.00E-05	0.6787	0.82412	(lh.inferiorparietal_4)(lh.isthmuscingulate_3)(lh.supramarginal_1)(lh.transversetemporal_2
0.00036	1.00E-05	0.6787	0.82412	(lh.inferiorparietal_4)(lh.superiortemporal_2)(lh.supramarginal_1)(lh.transversetemporal_
0.00036	1.00E-05	0.6787	0.82412	(Left-Thalamus-Proper)(lh.inferiorparietal_4)(lh.supramarginal_1)(lh.transversetemporal_2
0.00036	1.00E-05	0.6787	0.82412	(lh.bankssts_3)(lh.inferiorparietal_4)(lh.insula_2)(lh.supramarginal_1)
0.00036	1.00E-05	0.6787	0.82412	(lh.inferiorparietal_4)(lh.supramarginal_1)(lh.transversetemporal_2)
0.0004	1.00E-05	0.68592	0.82915	(lh.inferiorparietal_4)(lh.insula_2)(lh.isthmuscingulate_2)(lh.supramarginal_1)
0.0004	1.00E-05	0.68592	0.82915	(lh.fusiform_5)(lh.inferiorparietal_4)(lh.superiortemporal_3)(lh.supramarginal_1)
0.0004	1.00E-05	0.68592	0.82915	(Left-Putamen)(lh.bankssts_3)(lh.inferiorparietal_4)(lh.supramarginal_1)
0.0004	1.00E-05	0.66787	0.81407	(Left-Pallidum)(lh.inferiorparietal_4)(lh.isthmuscingulate_2)(lh.supramarginal_1)
0.00044	1.00E-05	0.65704	0.80402	(Left-Caudate)(lh.inferiorparietal_4)(lh.inferiorparietal_5)(lh.supramarginal_1)
0.00044	1.00E-05	0.69314	0.83417	(Left-Putamen)(lh.inferiorparietal_4)(lh.isthmuscingulate_2)(lh.supramarginal_1)
0.00044	1.00E-05	0.69314	0.83417	(lh.fusiform_5)(lh.inferiorparietal_4)(lh.insula_2)(lh.supramarginal_1)
0.00044	1.00E-05	0.69314	0.83417	(lh.fusiform_5)(lh.inferiorparietal_4)(lh.lingual_8)(lh.supramarginal_1)
0.00044	1.00E-05	0.67509	0.8191	(lh.inferiorparietal_4)(lh.parahippocampal_3)(lh.superiortemporal_3)(lh.supramarginal_1)
0.00044	1.00E-05	0.67509	0.8191	(Left-Pallidum)(lh.fusiform_5)(lh.inferiorparietal_4)(lh.supramarginal_1)
0.00046	1.00E-05	0.78339	0.90452	(Left-Pallidum)(lh.inferiorparietal_4)(lh.inferiorparietal_5)(lh.precuneus_11)
0.00048	1.00E-05	0.70036	0.8392	(Left-Putamen)(lh.fusiform_5)(lh.inferiorparietal_4)(lh.supramarginal_1)
0.00054	1.00E-05	0.68953	0.82915	(lh.bankssts_3)(lh.inferiorparietal_4)(lh.isthmuscingulate_3)(lh.supramarginal_1)
0.00054	1.00E-05	0.68953	0.82915	(Left-Thalamus-Proper)(lh.bankssts_3)(lh.inferiorparietal_4)(lh.supramarginal_1)
0.00054	1.00E-05	0.68953	0.82915	(lh.inferiorparietal_4)(lh.lingual_6)(lh.lingual_8)(lh.supramarginal_1)
0.00054	1.00E-05	0.68953	0.82915	(lh.bankssts_3)(lh.inferiorparietal_4)(lh.supramarginal_1)
0.00054	1.00E-05	0.68953	0.82915	(lh.bankssts_3)(lh.inferiorparietal_4)(lh.superiortemporal_2)(lh.supramarginal_1)
0.00059	1.00E-05	0.66065	0.80402	(lh.bankssts_3)(lh.inferiorparietal_4)(lh.supramarginal_1)(lh.transversetemporal_2)
0.00059	1.00E-05	0.69675	0.83417	(Left-Thalamus-Proper)(lh.inferiorparietal_4)(lh.isthmuscingulate_2)(lh.supramarginal_1)
0.00059	1.00E-05	0.69675	0.83417	(lh.inferiorparietal_4)(lh.isthmuscingulate_2)(lh.superiortemporal_2)(lh.supramarginal_1)

0.0006	1.00E-05	0.6787	0.8191	(lh.bankssts_3)(lh.inferiorparietal_4)(lh.lingual_8)(lh.supramarginal_1)
0.0006	1.00E-05	0.6787	0.8191	(lh.bankssts_3)(lh.inferiorparietal_4)(lh.lingual_6)(lh.supramarginal_1)
0.0006	1.00E-05	0.6787	0.8191	(lh.inferiorparietal_4)(lh.insula_2)(lh.parahippocampal_3)(lh.supramarginal_1)
0.00065	1.00E-05	0.70397	0.8392	(lh.fusiform_5)(lh.inferiorparietal_4)(lh.superiortemporal_2)(lh.supramarginal_1)
0.00065	1.00E-05	0.70397	0.8392	(lh.fusiform_5)(lh.inferiorparietal_4)(lh.supramarginal_1)
0.00065	1.00E-05	0.70397	0.8392	(Left-Thalamus-Proper)(lh.fusiform_5)(lh.inferiorparietal_4)(lh.supramarginal_1)
0.00065	1.00E-05	0.70397	0.8392	(lh.fusiform_5)(lh.inferiorparietal_4)(lh.isthmuscingulate_3)(lh.supramarginal_1)
0.00065	1.00E-05	0.66787	0.80905	(Left-Caudate)(Left-Putamen)(lh.inferiorparietal_4)(lh.supramarginal_1)
0.00066	1.00E-05	0.68592	0.82412	(lh.inferiorparietal_4)(lh.parahippocampal_3)(lh.superiortemporal_2)(lh.supramarginal_1)
0.00066	1.00E-05	0.68592	0.82412	(lh.inferiorparietal_4)(lh.isthmuscingulate_2)(lh.lingual_8)(lh.supramarginal_1)
0.00066	1.00E-05	0.68592	0.82412	(lh.inferiorparietal_4)(lh.insula_1)(lh.precuneus_11)(lh.supramarginal_1)
0.00066	1.00E-05	0.68592	0.82412	(lh.inferiorparietal_4)(lh.isthmuscingulate_2)(lh.lingual_6)(lh.supramarginal_1)
0.00066	1.00E-05	0.68592	0.82412	(lh.inferiorparietal_4)(lh.parahippocampal_3)(lh.supramarginal_1)
0.00066	1.00E-05	0.68592	0.82412	(lh.inferiorparietal_4)(lh.isthmuscingulate_3)(lh.parahippocampal_3)(lh.supramarginal_1)
0.00066	1.00E-05	0.68592	0.82412	(Left-Putamen)(lh.inferiorparietal_4)(lh.parahippocampal_3)(lh.supramarginal_1)
0.00066	1.00E-05	0.68592	0.82412	(Left-Thalamus-Proper)(lh.inferiorparietal_4)(lh.parahippocampal_3)(lh.supramarginal_1)
0.00068	1.00E-05	0.72924	0.8593	(lh.inferiorparietal_4)(lh.inferiorparietal_5)(lh.precuneus_11)(lh.superiortemporal_8)
0.00068	1.00E-05	0.80144	0.91457	(lh.inferiorparietal_4)(lh.inferiorparietal_5)(lh.lingual_8)(lh.precuneus_11)
0.00071	1.00E-05	0.81588	0.92462	(lh.inferiorparietal_4)(lh.inferiorparietal_5)(lh.isthmuscingulate_3)(lh.precuneus_11)
0.00071	1.00E-05	0.81588	0.92462	(Left-Thalamus-Proper)(lh.inferiorparietal_4)(lh.inferiorparietal_5)(lh.precuneus_11)
0.00071	1.00E-05	0.81588	0.92462	(lh.inferiorparietal_4)(lh.inferiorparietal_5)(lh.precuneus_11)
0.00072	1.00E-05	0.67509	0.81407	(lh.inferiorparietal_4)(lh.lingual_8)(lh.parahippocampal_3)(lh.supramarginal_1)
0.00072	1.00E-05	0.67509	0.81407	(lh.inferiorparietal_4)(lh.lingual_6)(lh.parahippocampal_3)(lh.supramarginal_1)
0.00072	1.00E-05	0.69314	0.82915	(lh.fusiform_5)(lh.inferiorparietal_4)(lh.lingual_6)(lh.supramarginal_1)
0.00072	1.00E-05	0.69314	0.82915	(lh.fusiform_5)(lh.inferiorparietal_4)(lh.isthmuscingulate_2)(lh.supramarginal_1)
0.00074	1.00E-05	0.78873	0.90452	(Left-Putamen)(lh.bankssts_3)(lh.inferiorparietal_4)(lh.inferiorparietal_5)
0.00076	1.00E-05	0.79577	0.90955	(Left-Putamen)(lh.bankssts_3)(lh.inferiorparietal_4)(lh.precuneus_11)
0.00078	1.00E-05	0.66426	0.80402	(Left-Pallidum)(lh.inferiorparietal_4)(lh.parahippocampal_3)(lh.supramarginal_1)
0.00083	1.00E-05	0.78339	0.8995	(lh.inferiorparietal_4)(lh.inferiorparietal_5)(lh.precuneus_11)(lh.transversetemporal_2)
0.00087	1.00E-05	0.67148	0.80905	(Left-Caudate)(lh.inferiorparietal_4)(lh.superiortemporal_2)(lh.supramarginal_1)
0.00087	1.00E-05	0.67148	0.80905	(Left-Caudate)(lh.inferiorparietal_4)(lh.supramarginal_1)
0.00087	1.00E-05	0.67148	0.80905	(Left-Caudate)(lh.inferiorparietal_4)(lh.isthmuscingulate_3)(lh.supramarginal_1)
0.00087	1.00E-05	0.67148	0.80905	(Left-Caudate)(Left-Thalamus-Proper)(lh.inferiorparietal_4)(lh.supramarginal_1)
0.00091	1.00E-05	0.79783	0.90955	(lh.inferiorparietal_4)(lh.inferiorparietal_5)(lh.insula_2)(lh.lingual_6)
0.00091	1.00E-05	0.79783	0.90955	(lh.inferiorparietal_4)(lh.inferiorparietal_5)(lh.precuneus_11)(lh.superiortemporal_3)
0.00094	1.00E-05	0.80505	0.91457	(Left-Putamen)(lh.inferiorparietal_4)(lh.inferiorparietal_5)(lh.lingual_6)

0.00094	1.00E-05	0.80505	0.91457	(lh.inferiorparietal_4)(lh.insula_2)(lh.lingual_6)(lh.precuneus_11)
0.00096	1.00E-05	0.6787	0.81407	(lh.bankssts_3)(lh.inferiorparietal_4)(lh.isthmuscingulate_2)(lh.supramarginal_1)
0.00097	1.00E-05	0.81227	0.9196	(Left-Putamen)(lh.inferiorparietal_4)(lh.lingual_6)(lh.precuneus_11)
0.00106	1.00E-05	0.68592	0.8191	(lh.bankssts_3)(lh.fusiform_5)(lh.inferiorparietal_4)(lh.supramarginal_1)
0.00106	1.00E-05	0.74729	0.86935	(lh.inferiorparietal_4)(lh.inferiorparietal_5)(lh.lingual_7)(lh.precuneus_11)
0.00109	1.00E-05	0.90614	0.9799	(Left-Putamen)(lh.inferiorparietal_5)(lh.lingual_6)(lh.precuneus_11)
0.00113	1.00E-05	0.88087	0.96482	(Left-Putamen)(lh.inferiorparietal_5)(lh.precuneus_11)(lh.transversetemporal_2)
0.00115	1.00E-05	0.67509	0.80905	(lh.inferiorparietal_4)(lh.isthmuscingulate_2)(lh.parahippocampal_3)(lh.supramarginal_1)
0.00117	1.00E-05	0.76761	0.88442	(Left-Pallidum)(lh.bankssts_3)(lh.inferiorparietal_4)(lh.inferiorparietal_5)
0.00122	1.00E-05	0.77465	0.88945	(Left-Pallidum)(lh.bankssts_3)(lh.inferiorparietal_4)(lh.precuneus_11)
0.00127	1.00E-05	0.68231	0.81407	(lh.inferiorparietal_4)(lh.insula_1)(lh.superiortemporal_3)(lh.supramarginal_1)
0.00127	1.00E-05	0.68231	0.81407	(lh.fusiform_5)(lh.inferiorparietal_4)(lh.parahippocampal_3)(lh.supramarginal_1)
0.00129	1.00E-05	0.89892	0.97487	(lh.inferiorparietal_5)(lh.insula_2)(lh.lingual_6)(lh.precuneus_11)
0.00129	1.00E-05	0.86643	0.95477	(lh.inferiorparietal_5)(lh.lingual_6)(lh.precuneus_11)(lh.transversetemporal_2)
0.0013	1.00E-05	0.76895	0.88442	(lh.inferiorparietal_5)(lh.precuneus_11)(lh.superiortemporal_3)(lh.supramarginal_1)
0.00132	1.00E-05	0.78873	0.8995	(lh.bankssts_3)(lh.inferiorparietal_4)(lh.inferiorparietal_5)(lh.insula_2)
0.00133	1.00E-05	0.81588	0.9196	(lh.inferiorparietal_4)(lh.inferiorparietal_5)(lh.precuneus_11)(lh.superiortemporal_2)
0.00136	1.00E-05	0.79577	0.90452	(lh.bankssts_3)(lh.inferiorparietal_4)(lh.inferiorparietal_5)(lh.precuneus_11)
0.00136	1.00E-05	0.79577	0.90452	(lh.bankssts_3)(lh.inferiorparietal_4)(lh.insula_2)(lh.precuneus_11)
0.00139	1.00E-05	0.72563	0.84925	(lh.inferiorparietal_4)(lh.inferiorparietal_5)(lh.lingual_6)(lh.superiortemporal_8)
0.00141	1.00E-05	0.68953	0.8191	(lh.inferiorparietal_4)(lh.insula_1)(lh.insula_2)(lh.supramarginal_1)
0.00141	1.00E-05	0.68953	0.8191	(lh.inferiorparietal_4)(lh.inferiorparietal_5)(lh.insula_1)(lh.supramarginal_1)
0.00143	1.00E-05	0.7509	0.86935	(lh.inferiorparietal_5)(lh.precuneus_11)(lh.supramarginal_1)(lh.transversetemporal_2)
0.00144	1.00E-05	0.9278	0.98995	(Left-Putamen)(Left-Thalamus-Proper)(lh.inferiorparietal_5)(lh.precuneus_11)
0.00144	1.00E-05	0.9278	0.98995	(Left-Putamen)(lh.inferiorparietal_5)(lh.precuneus_11)
0.00144	1.00E-05	0.9278	0.98995	(Left-Putamen)(lh.inferiorparietal_5)(lh.isthmuscingulate_3)(lh.precuneus_11)
0.00151	1.00E-05	0.73285	0.85427	(lh.inferiorparietal_4)(lh.lingual_6)(lh.precuneus_11)(lh.superiortemporal_8)
0.00155	1.00E-05	0.79061	0.8995	(lh.inferiorparietal_4)(lh.inferiorparietal_5)(lh.parahippocampal_3)(lh.precuneus_11)
0.00155	1.00E-05	0.69675	0.82412	(Left-Putamen)(lh.inferiorparietal_4)(lh.insula_1)(lh.supramarginal_1)
0.00163	1.00E-05	0.79783	0.90452	(Left-Pallidum)(lh.inferiorparietal_4)(lh.inferiorparietal_5)(lh.insula_2)
0.0017	1.00E-05	0.80505	0.90955	(lh.inferiorparietal_4)(lh.inferiorparietal_5)(lh.insula_2)(lh.lingual_8)
0.0017	1.00E-05	0.80505	0.90955	(Left-Pallidum)(lh.inferiorparietal_4)(lh.insula_2)(lh.precuneus_11)
0.00174	1.00E-05	0.87726	0.9598	(lh.inferiorparietal_5)(lh.insula_2)(lh.precuneus_11)(lh.transversetemporal_2)
0.00177	1.00E-05	0.81227	0.91457	(Left-Putamen)(lh.inferiorparietal_4)(lh.inferiorparietal_5)(lh.lingual_8)
0.00177	1.00E-05	0.81227	0.91457	(lh.fusiform_5)(lh.inferiorparietal_4)(lh.inferiorparietal_5)(lh.precuneus_11)
0.00177	1.00E-05	0.81227	0.91457	(lh.inferiorparietal_4)(lh.inferiorparietal_5)(lh.isthmuscingulate_3)(lh.lingual_6)

0.00177	1.00E-05	0.81227	0.91457	(lh.inferiorparietal_4)(lh.insula_2)(lh.lingual_8)(lh.precuneus_11)
0.00177	1.00E-05	0.81227	0.91457	(Left-Thalamus-Proper)(lh.inferiorparietal_4)(lh.inferiorparietal_5)(lh.lingual_6)
0.00177	1.00E-05	0.81227	0.91457	(lh.inferiorparietal_4)(lh.inferiorparietal_5)(lh.lingual_6)
0.00177	1.00E-05	0.74729	0.86432	(lh.precuneus_11)(lh.superiortemporal_3)(lh.supramarginal_1)(lh.transversetemporal_2)
0.00183	1.00E-05	0.81949	0.9196	(Left-Thalamus-Proper)(lh.inferiorparietal_4)(lh.lingual_6)(lh.precuneus_11)
0.00183	1.00E-05	0.81949	0.9196	(lh.inferiorparietal_4)(lh.inferiorparietal_5)(lh.insula_2)
0.00183	1.00E-05	0.81949	0.9196	(lh.inferiorparietal_4)(lh.inferiorparietal_5)(lh.insula_2)(lh.isthmuscingulate_3)
0.00183	1.00E-05	0.81949	0.9196	(Left-Thalamus-Proper)(lh.inferiorparietal_4)(lh.inferiorparietal_5)(lh.insula_2)
0.00183	1.00E-05	0.81949	0.9196	(Left-Putamen)(lh.inferiorparietal_4)(lh.lingual_8)(lh.precuneus_11)
0.00183	1.00E-05	0.81949	0.9196	(lh.inferiorparietal_4)(lh.isthmuscingulate_3)(lh.lingual_6)(lh.precuneus_11)
0.00183	1.00E-05	0.81949	0.9196	(lh.inferiorparietal_4)(lh.lingual_6)(lh.precuneus_11)
0.00183	1.00E-05	0.81949	0.9196	(Left-Putamen)(lh.inferiorparietal_4)(lh.inferiorparietal_5)(lh.insula_2)
0.00184	1.00E-05	0.72924	0.84925	(lh.fusiform_6)(lh.inferiorparietal_4)(lh.inferiorparietal_5)(lh.precuneus_11)
0.00187	1.00E-05	0.77978	0.88945	(lh.inferiorparietal_5)(lh.insula_2)(lh.precuneus_11)(lh.supramarginal_1)
0.00188	1.00E-05	0.82671	0.92462	(lh.inferiorparietal_4)(lh.insula_2)(lh.isthmuscingulate_3)(lh.precuneus_11)
0.00188	1.00E-05	0.82671	0.92462	(lh.inferiorparietal_4)(lh.insula_2)(lh.precuneus_11)
0.00188	1.00E-05	0.82671	0.92462	(Left-Putamen)(lh.inferiorparietal_4)(lh.inferiorparietal_5)(lh.isthmuscingulate_3)
0.00188	1.00E-05	0.82671	0.92462	(Left-Thalamus-Proper)(lh.inferiorparietal_4)(lh.insula_2)(lh.precuneus_11)
0.00188	1.00E-05	0.82671	0.92462	(Left-Putamen)(lh.inferiorparietal_4)(lh.inferiorparietal_5)
0.00188	1.00E-05	0.82671	0.92462	(Left-Putamen)(lh.inferiorparietal_4)(lh.insula_2)(lh.precuneus_11)
0.00188	1.00E-05	0.82671	0.92462	(Left-Putamen)(Left-Thalamus-Proper)(lh.inferiorparietal_4)(lh.inferiorparietal_5)
0.00191	1.00E-05	0.92058	0.98492	(Left-Thalamus-Proper)(lh.inferiorparietal_5)(lh.insula_2)(lh.precuneus_11)
0.00191	1.00E-05	0.92058	0.98492	(Left-Putamen)(lh.inferiorparietal_5)(lh.insula_2)(lh.precuneus_11)
0.00191	1.00E-05	0.92058	0.98492	(lh.inferiorparietal_5)(lh.insula_2)(lh.precuneus_11)
0.00191	1.00E-05	0.92058	0.98492	(lh.inferiorparietal_5)(lh.insula_2)(lh.isthmuscingulate_3)(lh.precuneus_11)
0.00191	1.00E-05	0.83394	0.92965	(Left-Putamen)(lh.inferiorparietal_4)(lh.precuneus_11)
0.00191	1.00E-05	0.83394	0.92965	(Left-Putamen)(lh.inferiorparietal_4)(lh.isthmuscingulate_3)(lh.precuneus_11)
0.00191	1.00E-05	0.83394	0.92965	(Left-Putamen)(Left-Thalamus-Proper)(lh.inferiorparietal_4)(lh.precuneus_11)
0.00198	1.00E-05	0.76761	0.8794	(lh.bankssts_3)(lh.inferiorparietal_4)(lh.inferiorparietal_5)(lh.parahippocampal_3)
0.00198	1.00E-05	0.76761	0.8794	(lh.bankssts_3)(lh.inferiorparietal_4)(lh.inferiorparietal_5)(lh.transversetemporal_2)
0.00198	1.00E-05	0.787	0.89447	(Left-Putamen)(lh.inferiorparietal_5)(lh.precuneus_11)(lh.supramarginal_1)
0.002	1.00E-05	0.73646	0.85427	(lh.inferiorparietal_4)(lh.inferiorparietal_5)(lh.parahippocampal_1)(lh.precuneus_11)
0.00204	1.00E-05	0.70036	0.82412	(Left-Thalamus-Proper)(lh.inferiorparietal_4)(lh.insula_1)(lh.supramarginal_1)
0.00204	1.00E-05	0.70036	0.82412	(lh.inferiorparietal_4)(lh.insula_1)(lh.superiortemporal_2)(lh.supramarginal_1)
0.00204	1.00E-05	0.70036	0.82412	(lh.inferiorparietal_4)(lh.insula_1)(lh.isthmuscingulate_3)(lh.supramarginal_1)
0.00204	1.00E-05	0.70036	0.82412	(lh.inferiorparietal_4)(lh.insula_1)(lh.supramarginal_1)

0.00205	1.00E-05	0.71841	0.8392	(lh.inferiorparietal_4)(lh.parahippocampal_1)(lh.precuneus_11)(lh.superiortemporal_8)
0.00205	1.00E-05	0.76173	0.87437	(Left-Putamen)(lh.precuneus_11)(lh.supramarginal_1)(lh.transversetemporal_2)
0.00205	1.00E-05	0.76173	0.87437	(Brain_Stem)(lh.inferiorparietal_4)(lh.inferiorparietal_5)(lh.precuneus_11)
0.00208	1.00E-05	0.77465	0.88442	(lh.bankssts_3)(lh.inferiorparietal_4)(lh.precuneus_11)(lh.transversetemporal_2)
0.00208	1.00E-05	0.77465	0.88442	(lh.bankssts_3)(lh.inferiorparietal_4)(lh.parahippocampal_3)(lh.precuneus_11)
0.00209	1.00E-05	0.79422	0.8995	(Left-Pallidum)(lh.inferiorparietal_4)(lh.lingual_8)(lh.precuneus_11)
0.00209	1.00E-05	0.79422	0.8995	(lh.inferiorparietal_4)(lh.inferiorparietal_5)(lh.lingual_6)(lh.superiortemporal_3)
0.00217	1.00E-05	0.74368	0.8593	(lh.fusiform_7)(lh.inferiorparietal_4)(lh.parahippocampal_1)(lh.precuneus_11)
0.00217	1.00E-05	0.74368	0.8593	(lh.inferiorparietal_4)(lh.insula_4)(lh.parahippocampal_1)(lh.precuneus_11)
0.0022	1.00E-05	0.68953	0.81407	(lh.inferiorparietal_4)(lh.insula_1)(lh.lingual_8)(lh.supramarginal_1)
0.0022	1.00E-05	0.68953	0.81407	(lh.inferiorparietal_4)(lh.insula_1)(lh.lingual_6)(lh.supramarginal_1)
0.0022	1.00E-05	0.80144	0.90452	(Left-Pallidum)(Left-Putamen)(lh.inferiorparietal_4)(lh.inferiorparietal_5)
0.0022	1.00E-05	0.80144	0.90452	(lh.inferiorparietal_4)(lh.inferiorparietal_5)(lh.lingual_6)(lh.lingual_8)
0.0022	1.00E-05	0.80144	0.90452	(Left-Pallidum)(lh.inferiorparietal_4)(lh.inferiorparietal_5)(lh.isthmuscingulate_3)
0.0022	1.00E-05	0.80144	0.90452	(Left-Pallidum)(lh.inferiorparietal_4)(lh.inferiorparietal_5)
0.0022	1.00E-05	0.80144	0.90452	(Left-Putamen)(lh.inferiorparietal_4)(lh.precuneus_11)(lh.transversetemporal_2)
0.0022	1.00E-05	0.80144	0.90452	(lh.inferiorparietal_4)(lh.lingual_6)(lh.precuneus_11)(lh.superiortemporal_3)
0.0022	1.00E-05	0.80144	0.90452	(Left-Pallidum)(Left-Thalamus-Proper)(lh.inferiorparietal_4)(lh.inferiorparietal_5)
0.00223	1.00E-05	0.72563	0.84422	(lh.bankssts_3)(lh.inferiorparietal_4)(lh.inferiorparietal_5)(lh.superiortemporal_8)
0.00229	1.00E-05	0.78873	0.89447	(lh.bankssts_3)(lh.inferiorparietal_4)(lh.inferiorparietal_5)(lh.lingual_6)
0.00229	1.00E-05	0.78873	0.89447	(lh.bankssts_3)(lh.inferiorparietal_4)(lh.inferiorparietal_5)(lh.lingual_8)
0.00229	1.00E-05	0.78873	0.89447	(Left-Pallidum)(lh.inferiorparietal_4)(lh.inferiorparietal_5)(lh.lingual_6)
0.00231	1.00E-05	0.80866	0.90955	(Left-Pallidum)(lh.inferiorparietal_4)(lh.precuneus_11)
0.00231	1.00E-05	0.80866	0.90955	(Left-Pallidum)(Left-Thalamus-Proper)(lh.inferiorparietal_4)(lh.precuneus_11)
0.00231	1.00E-05	0.80866	0.90955	(Left-Pallidum)(lh.inferiorparietal_4)(lh.isthmuscingulate_3)(lh.precuneus_11)
0.00231	1.00E-05	0.80866	0.90955	(lh.inferiorparietal_4)(lh.lingual_6)(lh.lingual_8)(lh.precuneus_11)
0.00231	1.00E-05	0.80866	0.90955	(Left-Pallidum)(Left-Putamen)(lh.inferiorparietal_4)(lh.precuneus_11)
0.00231	1.00E-05	0.88809	0.96482	(lh.inferiorparietal_5)(lh.isthmuscingulate_3)(lh.precuneus_11)(lh.transversetemporal_2)
0.00231	1.00E-05	0.88809	0.96482	(lh.inferiorparietal_5)(lh.precuneus_11)(lh.transversetemporal_2)
0.00231	1.00E-05	0.88809	0.96482	(Left-Thalamus-Proper)(lh.inferiorparietal_5)(lh.precuneus_11)(lh.transversetemporal_2)
0.00234	1.00E-05	0.91336	0.9799	(lh.inferiorparietal_5)(lh.lingual_6)(lh.precuneus_11)
0.00234	1.00E-05	0.91336	0.9799	(Left-Thalamus-Proper)(lh.inferiorparietal_5)(lh.lingual_6)(lh.precuneus_11)
0.00234	1.00E-05	0.91336	0.9799	(lh.inferiorparietal_5)(lh.isthmuscingulate_3)(lh.lingual_6)(lh.precuneus_11)
0.00235	1.00E-05	0.7509	0.86432	(lh.inferiorparietal_4)(lh.inferiorparietal_5)(lh.insula_4)(lh.precuneus_11)
0.00235	1.00E-05	0.7509	0.86432	(lh.inferiorparietal_4)(lh.inferiorparietal_5)(lh.insula_2)(lh.lingual_7)
0.00238	1.00E-05	0.79577	0.8995	(lh.bankssts_3)(lh.inferiorparietal_4)(lh.lingual_8)(lh.precuneus_11)

0.00238	1.00E-05	0.79577	0.8995	(lh.bankssts_3)(lh.inferiorparietal_4)(lh.lingual_6)(lh.precuneus_11)
0.00238	1.00E-05	0.79577	0.8995	(Left-Putamen)(lh.bankssts_3)(lh.inferiorparietal_4)(lh.lingual_6)
0.00238	1.00E-05	0.79577	0.8995	(Left-Pallidum)(lh.inferiorparietal_4)(lh.lingual_6)(lh.precuneus_11)
0.00238	1.00E-05	0.79577	0.8995	(Left-Putamen)(lh.bankssts_3)(lh.inferiorparietal_4)(lh.lingual_8)
0.00238	1.00E-05	0.79577	0.8995	(Left-Putamen)(lh.inferiorparietal_4)(lh.inferiorparietal_5)(lh.transversetemporal_2)
0.00244	1.00E-05	0.73285	0.84925	(lh.bankssts_3)(lh.inferiorparietal_4)(lh.precuneus_11)(lh.superiortemporal_8)
0.00244	1.00E-05	0.73285	0.84925	(lh.fusiform_7)(lh.inferiorparietal_4)(lh.inferiorparietal_5)(lh.superiortemporal_8)
0.00244	1.00E-05	0.73285	0.84925	(lh.inferiorparietal_4)(lh.inferiorparietal_5)(lh.lingual_8)(lh.superiortemporal_8)
0.00244	1.00E-05	0.73285	0.84925	(Left-Pallidum)(lh.inferiorparietal_4)(lh.inferiorparietal_5)(lh.lingual_7)
0.00246	1.00E-05	0.7148	0.83417	(lh.inferiorparietal_4)(lh.inferiorparietal_5)(lh.parahippocampal_1)(lh.superiortemporal_8)
0.00247	1.00E-05	0.88087	0.9598	(Left-Pallidum)(lh.inferiorparietal_5)(lh.lingual_6)(lh.precuneus_11)
0.00251	1.00E-05	0.78339	0.88945	(lh.precuneus_11)(lh.superiortemporal_3)(lh.supramarginal_1)
0.00251	1.00E-05	0.78339	0.88945	(lh.precuneus_11)(lh.superiortemporal_2)(lh.superiortemporal_3)(lh.supramarginal_1)
0.00251	1.00E-05	0.78339	0.88945	(Left-Thalamus-Proper)(lh.precuneus_11)(lh.superiortemporal_3)(lh.supramarginal_1)
0.00251	1.00E-05	0.78339	0.88945	(Left-Putamen)(lh.precuneus_11)(lh.superiortemporal_3)(lh.supramarginal_1)
0.00251	1.00E-05	0.78339	0.88945	(lh.isthmuscingulate_3)(lh.precuneus_11)(lh.superiortemporal_3)(lh.supramarginal_1)
0.00254	1.00E-05	0.75812	0.86935	(lh.inferiorparietal_4)(lh.insula_2)(lh.lingual_7)(lh.precuneus_11)
0.00254	1.00E-05	0.75812	0.86935	(lh.insula_2)(lh.precuneus_11)(lh.supramarginal_1)(lh.transversetemporal_2)
0.00254	1.00E-05	0.75812	0.86935	(Left-Putamen)(lh.inferiorparietal_4)(lh.inferiorparietal_5)(lh.lingual_7)
0.0026	1.00E-05	0.68592	0.80905	(lh.inferiorparietal_4)(lh.lingual_7)(lh.precuneus_11)(lh.superiortemporal_8)
0.00265	1.00E-05	0.74007	0.85427	(lh.inferiorparietal_4)(lh.inferiorparietal_5)(lh.insula_4)(lh.parahippocampal_1)
0.00265	1.00E-05	0.74007	0.85427	(Left-Pallidum)(lh.inferiorparietal_4)(lh.lingual_7)(lh.precuneus_11)
0.00265	1.00E-05	0.74007	0.85427	(Left-Pallidum)(lh.inferiorparietal_4)(lh.inferiorparietal_5)(lh.superiortemporal_8)
0.00265	1.00E-05	0.74007	0.85427	(lh.fusiform_7)(lh.inferiorparietal_4)(lh.precuneus_11)(lh.superiortemporal_8)
0.00265	1.00E-05	0.74007	0.85427	(lh.inferiorparietal_4)(lh.lingual_8)(lh.precuneus_11)(lh.superiortemporal_8)
0.00265	1.00E-05	0.74007	0.85427	(Left-Amygdala)(lh.inferiorparietal_4)(lh.inferiorparietal_5)(lh.precuneus_11)
0.00265	1.00E-05	0.74007	0.85427	(lh.fusiform_7)(lh.inferiorparietal_4)(lh.inferiorparietal_5)(lh.parahippocampal_1)
0.00265	1.00E-05	0.74007	0.85427	(Left-Pallidum)(lh.precuneus_11)(lh.supramarginal_1)(lh.transversetemporal_2)
0.00266	1.00E-05	0.79061	0.89447	(lh.inferiorparietal_5)(lh.precuneus_11)(lh.supramarginal_1)
0.00266	1.00E-05	0.79061	0.89447	(lh.inferiorparietal_5)(lh.precuneus_11)(lh.superiortemporal_2)(lh.supramarginal_1)
0.00266	1.00E-05	0.79061	0.89447	(lh.inferiorparietal_5)(lh.isthmuscingulate_3)(lh.precuneus_11)(lh.supramarginal_1)
0.00266	1.00E-05	0.79061	0.89447	(Left-Thalamus-Proper)(lh.inferiorparietal_5)(lh.precuneus_11)(lh.supramarginal_1)
0.00269	1.00E-05	0.85921	0.94472	(Left-Pallidum)(lh.inferiorparietal_5)(lh.precuneus_11)(lh.transversetemporal_2)
0.00269	1.00E-05	0.72202	0.8392	(lh.fusiform_6)(lh.inferiorparietal_4)(lh.inferiorparietal_5)(lh.lingual_6)
0.00269	1.00E-05	0.72202	0.8392	(lh.lingual_7)(lh.precuneus_11)(lh.superiortemporal_3)(lh.supramarginal_1)
0.00273	1.00E-05	0.76534	0.87437	(Left-Putamen)(lh.inferiorparietal_4)(lh.lingual_7)(lh.precuneus_11)

0.00273	1.00E-05	0.76534	0.87437	(lh.precuneus_11)(lh.superiortemporal_2)(lh.supramarginal_1)(lh.transversetemporal_2)
0.00273	1.00E-05	0.76534	0.87437	(Left-Thalamus-Proper)(lh.precuneus_11)(lh.supramarginal_1)(lh.transversetemporal_2)
0.00282	1.00E-05	0.79783	0.8995	(lh.inferiorparietal_4)(lh.insula_2)(lh.precuneus_11)(lh.transversetemporal_2)
0.00287	1.00E-05	0.74729	0.8593	(Left-Thalamus-Proper)(lh.inferiorparietal_4)(lh.inferiorparietal_5)(lh.superiortemporal_8)
0.00287	1.00E-05	0.74729	0.8593	(Left-Putamen)(lh.inferiorparietal_4)(lh.inferiorparietal_5)(lh.superiortemporal_8)
0.00287	1.00E-05	0.74729	0.8593	(lh.inferiorparietal_4)(lh.inferiorparietal_5)(lh.insula_2)(lh.superiortemporal_8)
0.00287	1.00E-05	0.74729	0.8593	(lh.inferiorparietal_4)(lh.inferiorparietal_5)(lh.lingual_6)(lh.lingual_7)
0.00287	1.00E-05	0.74729	0.8593	(Left-Pallidum)(lh.inferiorparietal_4)(lh.precuneus_11)(lh.superiortemporal_8)
0.00287	1.00E-05	0.74729	0.8593	(lh.inferiorparietal_4)(lh.inferiorparietal_5)(lh.superiortemporal_8)
0.00287	1.00E-05	0.74729	0.8593	(lh.inferiorparietal_4)(lh.inferiorparietal_5)(lh.isthmuscingulate_3)(lh.superiortemporal_8)
0.00293	2.00E-05	0.77256	0.8794	(Left-Pallidum)(lh.insula_2)(lh.precuneus_11)(lh.supramarginal_1)
0.00293	2.00E-05	0.77256	0.8794	(lh.lingual_6)(lh.precuneus_11)(lh.superiortemporal_3)(lh.supramarginal_1)
0.00294	2.00E-05	0.72924	0.84422	(lh.inferiorparietal_5)(lh.lingual_7)(lh.precuneus_11)(lh.supramarginal_1)
0.00294	2.00E-05	0.72924	0.84422	(lh.fusiform_6)(lh.inferiorparietal_4)(lh.lingual_6)(lh.precuneus_11)
0.00294	2.00E-05	0.72924	0.84422	(lh.fusiform_6)(lh.inferiorparietal_4)(lh.inferiorparietal_5)(lh.lingual_8)
0.00297	2.00E-05	0.80505	0.90452	(lh.inferiorparietal_4)(lh.inferiorparietal_5)(lh.isthmuscingulate_2)(lh.precuneus_11)
0.00304	2.00E-05	0.89892	0.96985	(Left-Pallidum)(lh.inferiorparietal_5)(lh.insula_2)(lh.precuneus_11)
0.00311	2.00E-05	0.75451	0.86432	(lh.inferiorparietal_4)(lh.isthmuscingulate_3)(lh.precuneus_11)(lh.superiortemporal_8)
0.00311	2.00E-05	0.75451	0.86432	(Brain_Stem)(lh.inferiorparietal_4)(lh.inferiorparietal_5)(lh.lingual_6)
0.00311	2.00E-05	0.75451	0.86432	(lh.inferiorparietal_4)(lh.precuneus_11)(lh.superiortemporal_8)
0.00311	2.00E-05	0.75451	0.86432	(lh.inferiorparietal_5)(lh.superiortemporal_3)(lh.supramarginal_1)(lh.transversetemporal_[illegible]
0.00311	2.00E-05	0.75451	0.86432	(Left-Thalamus-Proper)(lh.inferiorparietal_4)(lh.precuneus_11)(lh.superiortemporal_8)
0.00311	2.00E-05	0.75451	0.86432	(lh.inferiorparietal_4)(lh.insula_2)(lh.precuneus_11)(lh.superiortemporal_8)
0.00311	2.00E-05	0.75451	0.86432	(Left-Putamen)(lh.inferiorparietal_4)(lh.precuneus_11)(lh.superiortemporal_8)
0.00311	2.00E-05	0.75451	0.86432	(lh.lingual_6)(lh.precuneus_11)(lh.supramarginal_1)(lh.transversetemporal_2)
0.00311	2.00E-05	0.75451	0.86432	(lh.inferiorparietal_4)(lh.lingual_6)(lh.lingual_7)(lh.precuneus_11)
0.00312	2.00E-05	0.81227	0.90955	(lh.inferiorparietal_4)(lh.inferiorparietal_5)(lh.lingual_6)(lh.superiortemporal_2)
0.00312	2.00E-05	0.81227	0.90955	(Left-Putamen)(lh.inferiorparietal_4)(lh.inferiorparietal_5)(lh.superiortemporal_3)
0.00314	2.00E-05	0.77978	0.88442	(lh.inferiorparietal_5)(lh.lingual_6)(lh.precuneus_11)(lh.supramarginal_1)
0.00314	2.00E-05	0.77978	0.88442	(lh.insula_2)(lh.precuneus_11)(lh.superiortemporal_3)(lh.supramarginal_1)
0.0032	2.00E-05	0.73646	0.84925	(lh.fusiform_6)(lh.inferiorparietal_4)(lh.lingual_8)(lh.precuneus_11)
0.00321	2.00E-05	0.93502	0.98995	(Left-Thalamus-Proper)(lh.inferiorparietal_5)(lh.isthmuscingulate_3)(lh.precuneus_11)
0.00321	2.00E-05	0.93502	0.98995	(lh.inferiorparietal_5)(lh.isthmuscingulate_3)(lh.precuneus_11)
0.00321	2.00E-05	0.93502	0.98995	(lh.inferiorparietal_5)(lh.precuneus_11)
0.00321	2.00E-05	0.93502	0.98995	(Left-Thalamus-Proper)(lh.inferiorparietal_5)(lh.precuneus_11)
0.00326	2.00E-05	0.81949	0.91457	(lh.inferiorparietal_4)(lh.inferiorparietal_5)(lh.insula_2)(lh.superiortemporal_2)

0.00326	2.00E-05	0.81949	0.91457	(Left-Thalamus-Proper)(lh.inferiorparietal_4)(lh.inferiorparietal_5)(lh.lingual_8)
0.00326	2.00E-05	0.81949	0.91457	(lh.inferiorparietal_4)(lh.inferiorparietal_5)(lh.isthmuscingulate_3)(lh.lingual_8)
0.00326	2.00E-05	0.81949	0.91457	(lh.inferiorparietal_4)(lh.lingual_6)(lh.precuneus_11)(lh.superiortemporal_2)
0.00326	2.00E-05	0.81949	0.91457	(lh.inferiorparietal_4)(lh.inferiorparietal_5)(lh.lingual_8)
0.00326	2.00E-05	0.81949	0.91457	(Left-Putamen)(lh.inferiorparietal_4)(lh.precuneus_11)(lh.superiortemporal_3)
0.00329	2.00E-05	0.8917	0.96482	(lh.inferiorparietal_5)(lh.lingual_6)(lh.precuneus_11)(lh.superiortemporal_3)
0.00339	2.00E-05	0.68953	0.80905	(lh.inferiorparietal_4)(lh.insula_1)(lh.isthmuscingulate_2)(lh.supramarginal_1)
0.00339	2.00E-05	0.82671	0.9196	(lh.inferiorparietal_4)(lh.insula_2)(lh.precuneus_11)(lh.superiortemporal_2)
0.00339	2.00E-05	0.82671	0.9196	(Left-Thalamus-Proper)(lh.inferiorparietal_4)(lh.lingual_8)(lh.precuneus_11)
0.00339	2.00E-05	0.82671	0.9196	(lh.inferiorparietal_4)(lh.isthmuscingulate_3)(lh.lingual_8)(lh.precuneus_11)
0.00339	2.00E-05	0.82671	0.9196	(Left-Putamen)(lh.inferiorparietal_4)(lh.inferiorparietal_5)(lh.superiortemporal_2)
0.00339	2.00E-05	0.82671	0.9196	(lh.inferiorparietal_4)(lh.lingual_8)(lh.precuneus_11)
0.00345	2.00E-05	0.77465	0.8794	(Left-Pallidum)(lh.bankssts_3)(lh.inferiorparietal_4)(lh.lingual_6)
0.00345	2.00E-05	0.77465	0.8794	(Left-Pallidum)(lh.bankssts_3)(lh.inferiorparietal_4)(lh.lingual_8)
0.00345	2.00E-05	0.77465	0.8794	(Left-Pallidum)(lh.inferiorparietal_4)(lh.inferiorparietal_5)(lh.transversetemporal_2)
0.00348	2.00E-05	0.88448	0.9598	(Left-Putamen)(lh.lingual_6)(lh.precuneus_11)(lh.transversetemporal_2)
0.00348	2.00E-05	0.88448	0.9598	(Left-Putamen)(lh.inferiorparietal_5)(lh.lingual_6)(lh.transversetemporal_2)
0.00348	2.00E-05	0.74368	0.85427	(lh.bankssts_3)(lh.precuneus_11)(lh.supramarginal_1)(lh.transversetemporal_2)
0.00349	2.00E-05	0.70758	0.82412	(lh.lingual_7)(lh.precuneus_11)(lh.supramarginal_1)(lh.transversetemporal_2)
0.0035	2.00E-05	0.83394	0.92462	(lh.inferiorparietal_4)(lh.inferiorparietal_5)
0.0035	2.00E-05	0.83394	0.92462	(Left-Thalamus-Proper)(lh.inferiorparietal_4)(lh.inferiorparietal_5)(lh.isthmuscingulate_3)
0.0035	2.00E-05	0.83394	0.92462	(Left-Putamen)(lh.inferiorparietal_4)(lh.precuneus_11)(lh.superiortemporal_2)
0.0035	2.00E-05	0.83394	0.92462	(lh.inferiorparietal_4)(lh.inferiorparietal_5)(lh.isthmuscingulate_3)
0.0035	2.00E-05	0.83394	0.92462	(Left-Thalamus-Proper)(lh.inferiorparietal_4)(lh.inferiorparietal_5)
0.00352	2.00E-05	0.72563	0.8392	(lh.fusiform_7)(lh.inferiorparietal_4)(lh.parahippocampal_1)(lh.superiortemporal_8)
0.00356	2.00E-05	0.79422	0.89447	(lh.inferiorparietal_4)(lh.lingual_8)(lh.precuneus_11)(lh.transversetemporal_2)
0.00356	2.00E-05	0.79422	0.89447	(lh.insula_2)(lh.precuneus_11)(lh.superiortemporal_2)(lh.supramarginal_1)
0.00356	2.00E-05	0.79422	0.89447	(Left-Putamen)(lh.insula_2)(lh.precuneus_11)(lh.supramarginal_1)
0.00356	2.00E-05	0.79422	0.89447	(Left-Thalamus-Proper)(lh.insula_2)(lh.precuneus_11)(lh.supramarginal_1)
0.00356	2.00E-05	0.79422	0.89447	(lh.insula_2)(lh.precuneus_11)(lh.supramarginal_1)
0.00356	2.00E-05	0.79422	0.89447	(lh.insula_2)(lh.isthmuscingulate_3)(lh.precuneus_11)(lh.supramarginal_1)
0.0036	2.00E-05	0.84116	0.92965	(lh.inferiorparietal_4)(lh.precuneus_11)
0.0036	2.00E-05	0.84116	0.92965	(Left-Thalamus-Proper)(lh.inferiorparietal_4)(lh.isthmuscingulate_3)(lh.precuneus_11)
0.0036	2.00E-05	0.84116	0.92965	(lh.inferiorparietal_4)(lh.isthmuscingulate_3)(lh.precuneus_11)
0.0036	2.00E-05	0.84116	0.92965	(Left-Thalamus-Proper)(lh.inferiorparietal_4)(lh.precuneus_11)
0.00362	2.00E-05	0.76895	0.87437	(Left-Putamen)(lh.inferiorparietal_5)(lh.supramarginal_1)(lh.transversetemporal_2)

0.00365	2.00E-05	0.78169	0.88442	(Left-Pallidum)(lh.inferiorparietal_4)(lh.precuneus_11)(lh.transversetemporal_2)
0.00369	2.00E-05	0.87004	0.94975	(lh.inferiorparietal_5)(lh.precuneus_11)(lh.superiortemporal_3)(lh.transversetemporal_2)
0.00372	2.00E-05	0.86282	0.94472	(lh.bankssts_3)(lh.inferiorparietal_5)(lh.precuneus_11)(lh.transversetemporal_2)
0.00374	2.00E-05	0.69675	0.81407	(lh.fusiform_5)(lh.inferiorparietal_4)(lh.insula_1)(lh.supramarginal_1)
0.00378	2.00E-05	0.80144	0.8995	(Left-Putamen)(lh.isthmuscingulate_3)(lh.precuneus_11)(lh.supramarginal_1)
0.00378	2.00E-05	0.80144	0.8995	(Left-Putamen)(lh.precuneus_11)(lh.superiortemporal_2)(lh.supramarginal_1)
0.00378	2.00E-05	0.80144	0.8995	(lh.inferiorparietal_4)(lh.inferiorparietal_5)(lh.lingual_8)(lh.superiortemporal_3)
0.00378	2.00E-05	0.80144	0.8995	(Left-Putamen)(lh.precuneus_11)(lh.supramarginal_1)
0.00378	2.00E-05	0.80144	0.8995	(Left-Thalamus-Proper)(lh.inferiorparietal_4)(lh.inferiorparietal_5)(lh.transversetemporal_
0.00378	2.00E-05	0.80144	0.8995	(Left-Pallidum)(lh.inferiorparietal_4)(lh.inferiorparietal_5)(lh.superiortemporal_2)
0.00378	2.00E-05	0.80144	0.8995	(lh.inferiorparietal_4)(lh.inferiorparietal_5)(lh.transversetemporal_2)
0.00378	2.00E-05	0.80144	0.8995	(lh.inferiorparietal_4)(lh.inferiorparietal_5)(lh.isthmuscingulate_3)(lh.transversetemporal_
0.00378	2.00E-05	0.80144	0.8995	(Left-Putamen)(Left-Thalamus-Proper)(lh.precuneus_11)(lh.supramarginal_1)
0.00378	2.00E-05	0.7509	0.8593	(Brain_Stem)(Left-Pallidum)(lh.inferiorparietal_4)(lh.inferiorparietal_5)
0.00378	2.00E-05	0.7509	0.8593	(lh.fusiform_7)(lh.inferiorparietal_4)(lh.insula_4)(lh.parahippocampal_1)
0.00383	2.00E-05	0.7148	0.82915	(Left-Pallidum)(lh.lingual_7)(lh.precuneus_11)(lh.supramarginal_1)
0.00384	2.00E-05	0.78873	0.88945	(Left-Pallidum)(lh.bankssts_3)(lh.inferiorparietal_4)
0.00384	2.00E-05	0.78873	0.88945	(Left-Pallidum)(lh.bankssts_3)(lh.inferiorparietal_4)(lh.isthmuscingulate_3)
0.00384	2.00E-05	0.78873	0.88945	(lh.bankssts_3)(lh.inferiorparietal_4)(lh.inferiorparietal_5)(lh.superiortemporal_3)
0.00384	2.00E-05	0.78873	0.88945	(lh.inferiorparietal_4)(lh.inferiorparietal_5)(lh.lingual_6)(lh.parahippocampal_3)
0.00384	2.00E-05	0.78873	0.88945	(lh.inferiorparietal_4)(lh.inferiorparietal_5)(lh.lingual_6)(lh.transversetemporal_2)
0.00385	2.00E-05	0.73285	0.84422	(lh.insula_2)(lh.lingual_7)(lh.precuneus_11)(lh.supramarginal_1)
0.00385	2.00E-05	0.73285	0.84422	(Left-Amygdala)(lh.inferiorparietal_4)(lh.inferiorparietal_5)(lh.lingual_6)
0.00385	2.00E-05	0.73285	0.84422	(lh.inferiorparietal_4)(lh.inferiorparietal_5)(lh.lingual_7)(lh.transversetemporal_2)
0.00389	2.00E-05	0.77617	0.8794	(Left-Pallidum)(Left-Thalamus-Proper)(lh.precuneus_11)(lh.supramarginal_1)
0.00389	2.00E-05	0.77617	0.8794	(Left-Pallidum)(lh.precuneus_11)(lh.superiortemporal_2)(lh.supramarginal_1)
0.00389	2.00E-05	0.77617	0.8794	(Left-Pallidum)(lh.isthmuscingulate_3)(lh.precuneus_11)(lh.supramarginal_1)
0.00389	2.00E-05	0.77617	0.8794	(lh.fusiform_5)(lh.precuneus_11)(lh.superiortemporal_3)(lh.supramarginal_1)
0.00389	2.00E-05	0.77617	0.8794	(Left-Pallidum)(lh.precuneus_11)(lh.supramarginal_1)
0.00389	2.00E-05	0.77617	0.8794	(Left-Pallidum)(Left-Putamen)(lh.precuneus_11)(lh.supramarginal_1)
0.00393	2.00E-05	0.90975	0.97487	(Left-Putamen)(lh.inferiorparietal_5)(lh.precuneus_11)(lh.superiortemporal_3)
0.00399	2.00E-05	0.80866	0.90452	(Left-Pallidum)(lh.inferiorparietal_4)(lh.precuneus_11)(lh.superiortemporal_2)
0.00399	2.00E-05	0.80866	0.90452	(lh.inferiorparietal_4)(lh.precuneus_11)(lh.transversetemporal_2)
0.00399	2.00E-05	0.80866	0.90452	(lh.inferiorparietal_4)(lh.lingual_8)(lh.precuneus_11)(lh.superiortemporal_3)
0.00399	2.00E-05	0.80866	0.90452	(lh.inferiorparietal_4)(lh.isthmuscingulate_3)(lh.precuneus_11)(lh.transversetemporal_2)
0.00399	2.00E-05	0.80866	0.90452	(lh.fusiform_5)(lh.inferiorparietal_4)(lh.inferiorparietal_5)(lh.lingual_6)

0.00399	2.00E-05	0.80866	0.90452	(Left-Thalamus-Proper)(lh.inferiorparietal_4)(lh.precuneus_11)(lh.transversetemporal_2)
0.00399	2.00E-05	0.80866	0.90452	(lh.inferiorparietal_4)(lh.inferiorparietal_5)(lh.insula_2)(lh.superiortemporal_3)
0.004	2.00E-05	0.73944	0.84925	(lh.inferiorparietal_4)(lh.isthmuscingulate_3)(lh.supramarginal_1)
0.004	2.00E-05	0.73944	0.84925	(lh.inferiorparietal_4)(lh.supramarginal_1)
0.00403	2.00E-05	0.79577	0.89447	(lh.bankssts_3)(lh.fusiform_5)(lh.inferiorparietal_4)(lh.inferiorparietal_5)
0.00403	2.00E-05	0.79577	0.89447	(lh.bankssts_3)(lh.inferiorparietal_4)(lh.precuneus_11)(lh.superiortemporal_3)
0.00403	2.00E-05	0.79577	0.89447	(lh.inferiorparietal_4)(lh.lingual_6)(lh.parahippocampal_3)(lh.precuneus_11)
0.00403	2.00E-05	0.79577	0.89447	(lh.bankssts_3)(lh.inferiorparietal_4)(lh.insula_2)(lh.lingual_8)
0.00403	2.00E-05	0.79577	0.89447	(Left-Putamen)(lh.inferiorparietal_4)(lh.lingual_6)(lh.transversetemporal_2)
0.00403	2.00E-05	0.79577	0.89447	(lh.bankssts_3)(lh.inferiorparietal_4)(lh.insula_2)(lh.lingual_6)
0.00403	2.00E-05	0.79577	0.89447	(lh.inferiorparietal_4)(lh.inferiorparietal_5)(lh.insula_2)(lh.transversetemporal_2)
0.00403	2.00E-05	0.79577	0.89447	(lh.inferiorparietal_4)(lh.lingual_6)(lh.precuneus_11)(lh.transversetemporal_2)
0.00403	2.00E-05	0.79577	0.89447	(Left-Pallidum)(lh.inferiorparietal_4)(lh.inferiorparietal_5)(lh.lingual_8)
0.00412	2.00E-05	0.70397	0.8191	(lh.fusiform_6)(lh.inferiorparietal_5)(lh.precuneus_11)(lh.supramarginal_1)
0.00415	2.00E-05	0.9278	0.98492	(Left-Putamen)(lh.inferiorparietal_5)(lh.precuneus_11)(lh.superiortemporal_2)
0.00417	2.00E-05	0.78339	0.88442	(lh.fusiform_5)(lh.inferiorparietal_5)(lh.precuneus_11)(lh.supramarginal_1)
0.00417	2.00E-05	0.78339	0.88442	(lh.insula_2)(lh.lingual_6)(lh.precuneus_11)(lh.supramarginal_1)
0.00419	2.00E-05	0.74007	0.84925	(lh.inferiorparietal_4)(lh.lingual_7)(lh.precuneus_11)(lh.transversetemporal_2)
0.00419	2.00E-05	0.74007	0.84925	(lh.inferiorparietal_4)(lh.inferiorparietal_5)(lh.lingual_7)(lh.parahippocampal_3)
0.00419	2.00E-05	0.74007	0.84925	(lh.inferiorparietal_4)(lh.lingual_6)(lh.parahippocampal_1)(lh.precuneus_11)
0.00419	2.00E-05	0.74007	0.84925	(lh.inferiorparietal_4)(lh.inferiorparietal_5)(lh.superiortemporal_3)(lh.superiortemporal_8)
0.00419	2.00E-05	0.74007	0.84925	(Left-Putamen)(lh.lingual_7)(lh.precuneus_11)(lh.supramarginal_1)
0.0042	2.00E-05	0.72202	0.83417	(lh.inferiorparietal_4)(lh.inferiorparietal_5)(lh.superiortemporal_8)(lh.transversetemporal_
0.0042	2.00E-05	0.81588	0.90955	(lh.fusiform_5)(lh.inferiorparietal_4)(lh.lingual_6)(lh.precuneus_11)
0.0042	2.00E-05	0.81588	0.90955	(Left-Thalamus-Proper)(lh.inferiorparietal_4)(lh.inferiorparietal_5)(lh.superiortemporal_3)
0.0042	2.00E-05	0.81588	0.90955	(lh.fusiform_5)(lh.inferiorparietal_4)(lh.inferiorparietal_5)(lh.insula_2)
0.0042	2.00E-05	0.81588	0.90955	(lh.inferiorparietal_4)(lh.inferiorparietal_5)(lh.superiortemporal_3)
0.0042	2.00E-05	0.81588	0.90955	(lh.inferiorparietal_4)(lh.insula_2)(lh.precuneus_11)(lh.superiortemporal_3)
0.0042	2.00E-05	0.81588	0.90955	(lh.inferiorparietal_4)(lh.inferiorparietal_5)(lh.superiortemporal_2)(lh.superiortemporal_3)
0.0042	2.00E-05	0.81588	0.90955	(lh.fusiform_5)(lh.inferiorparietal_4)(lh.inferiorparietal_5)(lh.lingual_8)
0.0042	2.00E-05	0.81588	0.90955	(lh.inferiorparietal_4)(lh.inferiorparietal_5)(lh.isthmuscingulate_3)(lh.superiortemporal_3)
0.00434	2.00E-05	0.90253	0.96985	(Left-Pallidum)(Left-Putamen)(lh.inferiorparietal_5)(lh.precuneus_11)
0.00434	2.00E-05	0.90253	0.96985	(Left-Pallidum)(Left-Thalamus-Proper)(lh.inferiorparietal_5)(lh.precuneus_11)
0.00434	2.00E-05	0.90253	0.96985	(Left-Pallidum)(lh.inferiorparietal_5)(lh.isthmuscingulate_3)(lh.precuneus_11)
0.00434	2.00E-05	0.90253	0.96985	(Left-Putamen)(lh.bankssts_3)(lh.inferiorparietal_5)(lh.precuneus_11)
0.00434	2.00E-05	0.90253	0.96985	(Left-Pallidum)(lh.inferiorparietal_5)(lh.precuneus_11)

0.00438	2.00E-05	0.69314	0.80905	(lh.fusiform_7)(lh.inferiorparietal_4)(lh.precuneus_11)(lh.superiortemporal_11)
0.0044	2.00E-05	0.8231	0.91457	(Left-Thalamus-Proper)(lh.inferiorparietal_4)(lh.precuneus_11)(lh.superiortemporal_3)
0.0044	2.00E-05	0.8231	0.91457	(lh.fusiform_5)(lh.inferiorparietal_4)(lh.insula_2)(lh.precuneus_11)
0.0044	2.00E-05	0.8231	0.91457	(Left-Putamen)(lh.fusiform_5)(lh.inferiorparietal_4)(lh.inferiorparietal_5)
0.0044	2.00E-05	0.8231	0.91457	(lh.inferiorparietal_4)(lh.precuneus_11)(lh.superiortemporal_3)
0.0044	2.00E-05	0.8231	0.91457	(lh.inferiorparietal_4)(lh.isthmuscingulate_3)(lh.precuneus_11)(lh.superiortemporal_3)
0.0044	2.00E-05	0.8231	0.91457	(lh.inferiorparietal_4)(lh.precuneus_11)(lh.superiortemporal_2)(lh.superiortemporal_3)
0.0044	2.00E-05	0.8231	0.91457	(lh.fusiform_5)(lh.inferiorparietal_4)(lh.lingual_8)(lh.precuneus_11)
0.00442	2.00E-05	0.76534	0.86935	(Brain_Stem)(lh.inferiorparietal_4)(lh.inferiorparietal_5)(lh.insula_2)
0.00442	2.00E-05	0.76534	0.86935	(lh.inferiorparietal_4)(lh.inferiorparietal_5)(lh.lingual_7)
0.00442	2.00E-05	0.76534	0.86935	(lh.inferiorparietal_5)(lh.insula_2)(lh.supramarginal_1)(lh.transversetemporal_2)
0.00442	2.00E-05	0.76534	0.86935	(lh.inferiorparietal_4)(lh.inferiorparietal_5)(lh.isthmuscingulate_3)(lh.lingual_7)
0.00442	2.00E-05	0.76534	0.86935	(Brain_Stem)(lh.inferiorparietal_4)(lh.lingual_6)(lh.precuneus_11)
0.00442	2.00E-05	0.76534	0.86935	(Left-Thalamus-Proper)(lh.inferiorparietal_4)(lh.inferiorparietal_5)(lh.lingual_7)
0.00445	2.00E-05	0.79061	0.88945	(Left-Putamen)(lh.lingual_6)(lh.precuneus_11)(lh.supramarginal_1)
0.00456	2.00E-05	0.74729	0.85427	(lh.inferiorparietal_4)(lh.inferiorparietal_5)(lh.insula_4)(lh.lingual_6)
0.00456	2.00E-05	0.74729	0.85427	(lh.inferiorparietal_4)(lh.precuneus_11)(lh.superiortemporal_3)(lh.superiortemporal_8)
0.00456	2.00E-05	0.74729	0.85427	(Left-Pallidum)(lh.inferiorparietal_5)(lh.supramarginal_1)(lh.transversetemporal_2)
0.00456	2.00E-05	0.74729	0.85427	(lh.inferiorparietal_4)(lh.inferiorparietal_5)(lh.superiortemporal_2)(lh.superiortemporal_8)
0.00456	2.00E-05	0.74729	0.85427	(lh.inferiorparietal_4)(lh.lingual_7)(lh.parahippocampal_3)(lh.precuneus_11)
0.00456	2.00E-05	0.74729	0.85427	(lh.inferiorparietal_4)(lh.inferiorparietal_5)(lh.lingual_7)(lh.superiortemporal_3)
0.00458	2.00E-05	0.83032	0.9196	(Left-Putamen)(lh.fusiform_5)(lh.inferiorparietal_4)(lh.precuneus_11)
0.00459	2.00E-05	0.72924	0.8392	(lh.inferiorparietal_4)(lh.inferiorparietal_5)(lh.parahippocampal_3)(lh.superiortemporal_8)
0.00459	2.00E-05	0.72924	0.8392	(lh.entorhinal_1)(lh.inferiorparietal_4)(lh.inferiorparietal_5)(lh.precuneus_11)
0.00459	2.00E-05	0.72924	0.8392	(lh.inferiorparietal_4)(lh.precuneus_11)(lh.superiortemporal_8)(lh.transversetemporal_2)
0.00466	2.00E-05	0.89531	0.96482	(lh.bankssts_3)(lh.inferiorparietal_5)(lh.insula_2)(lh.precuneus_11)
0.00474	2.00E-05	0.79783	0.89447	(Left-Pallidum)(lh.fusiform_5)(lh.inferiorparietal_4)(lh.inferiorparietal_5)
0.00474	2.00E-05	0.79783	0.89447	(lh.inferiorparietal_4)(lh.inferiorparietal_5)(lh.insula_2)(lh.parahippocampal_3)
0.00477	2.00E-05	0.77256	0.87437	(lh.inferiorparietal_5)(lh.superiortemporal_2)(lh.supramarginal_1)(lh.transversetemporal_
0.00477	2.00E-05	0.77256	0.87437	(Brain_Stem)(Left-Putamen)(lh.inferiorparietal_4)(lh.inferiorparietal_5)
0.00477	2.00E-05	0.77256	0.87437	(Left-Thalamus-Proper)(lh.inferiorparietal_4)(lh.lingual_7)(lh.precuneus_11)
0.00477	2.00E-05	0.77256	0.87437	(lh.inferiorparietal_4)(lh.isthmuscingulate_3)(lh.lingual_7)(lh.precuneus_11)
0.00477	2.00E-05	0.77256	0.87437	(lh.inferiorparietal_4)(lh.lingual_7)(lh.precuneus_11)
0.00477	2.00E-05	0.77256	0.87437	(Left-Thalamus-Proper)(lh.inferiorparietal_5)(lh.supramarginal_1)(lh.transversetemporal_2
0.00483	2.00E-05	0.70036	0.81407	(lh.fusiform_6)(lh.precuneus_11)(lh.superiortemporal_3)(lh.supramarginal_1)
0.0049	2.00E-05	0.88809	0.9598	(lh.inferiorparietal_5)(lh.precuneus_11)(lh.superiortemporal_2)(lh.transversetemporal_2)

0.00495	2.00E-05	0.75451	0.8593	(lh.inferiorparietal_4)(lh.lingual_7)(lh.precuneus_11)(lh.superiortemporal_3)
0.00495	2.00E-05	0.75451	0.8593	(lh.inferiorparietal_4)(lh.precuneus_11)(lh.superiortemporal_2)(lh.superiortemporal_8)
0.00495	2.00E-05	0.75451	0.8593	(lh.fusiform_7)(lh.inferiorparietal_4)(lh.inferiorparietal_5)(lh.insula_4)
0.00495	2.00E-05	0.75451	0.8593	(lh.inferiorparietal_4)(lh.inferiorparietal_5)(lh.lingual_7)(lh.lingual_8)
0.00495	2.00E-05	0.75451	0.8593	(lh.inferiorparietal_4)(lh.inferiorparietal_5)(lh.parahippocampal_2)(lh.precuneus_11)
0.00497	2.00E-05	0.92058	0.9799	(lh.inferiorparietal_5)(lh.insula_2)(lh.precuneus_11)(lh.superiortemporal_2)
0.00497	2.00E-05	0.92058	0.9799	(Left-Putamen)(lh.fusiform_5)(lh.inferiorparietal_5)(lh.precuneus_11)
0.00501	2.00E-05	0.73646	0.84422	(lh.fusiform_6)(lh.inferiorparietal_4)(lh.inferiorparietal_5)(lh.insula_2)
0.00501	2.00E-05	0.73646	0.84422	(lh.inferiorparietal_4)(lh.inferiorparietal_5)(lh.lingual_6)(lh.parahippocampal_1)
0.00501	2.00E-05	0.73646	0.84422	(lh.inferiorparietal_4)(lh.parahippocampal_3)(lh.precuneus_11)(lh.superiortemporal_8)
0.00503	2.00E-05	0.80505	0.8995	(Left-Pallidum)(lh.fusiform_5)(lh.inferiorparietal_4)(lh.precuneus_11)
0.00503	2.00E-05	0.80505	0.8995	(lh.isthmuscingulate_3)(lh.precuneus_11)(lh.superiortemporal_2)(lh.supramarginal_1)
0.00503	2.00E-05	0.80505	0.8995	(Left-Thalamus-Proper)(lh.isthmuscingulate_3)(lh.precuneus_11)(lh.supramarginal_1)
0.00503	2.00E-05	0.80505	0.8995	(Left-Putamen)(lh.inferiorparietal_4)(lh.inferiorparietal_5)(lh.parahippocampal_3)
0.00503	2.00E-05	0.80505	0.8995	(Left-Thalamus-Proper)(lh.precuneus_11)(lh.superiortemporal_2)(lh.supramarginal_1)
0.00503	2.00E-05	0.80505	0.8995	(Left-Pallidum)(lh.inferiorparietal_4)(lh.insula_2)(lh.lingual_6)
0.00503	2.00E-05	0.80505	0.8995	(lh.inferiorparietal_4)(lh.insula_2)(lh.parahippocampal_3)(lh.precuneus_11)
0.00503	2.00E-05	0.80505	0.8995	(lh.isthmuscingulate_3)(lh.precuneus_11)(lh.supramarginal_1)
0.00503	2.00E-05	0.80505	0.8995	(lh.precuneus_11)(lh.superiortemporal_2)(lh.supramarginal_1)
0.00503	2.00E-05	0.80505	0.8995	(Left-Thalamus-Proper)(lh.precuneus_11)(lh.supramarginal_1)
0.00503	2.00E-05	0.80505	0.8995	(lh.precuneus_11)(lh.supramarginal_1)
0.00505	2.00E-05	0.88087	0.95477	(lh.insula_2)(lh.lingual_6)(lh.precuneus_11)(lh.transversetemporal_2)
0.00505	2.00E-05	0.88087	0.95477	(lh.inferiorparietal_5)(lh.insula_2)(lh.lingual_6)(lh.transversetemporal_2)
0.00524	2.00E-05	0.76761	0.86935	(lh.bankssts_3)(lh.fusiform_6)(lh.precuneus_11)(lh.transversetemporal_2)
0.00532	2.00E-05	0.70758	0.8191	(lh.fusiform_6)(lh.insula_2)(lh.precuneus_11)(lh.supramarginal_1)
0.00533	2.00E-05	0.81227	0.90452	(lh.inferiorparietal_5)(lh.lingual_7)(lh.precuneus_11)(lh.transversetemporal_2)
0.00533	2.00E-05	0.81227	0.90452	(Left-Thalamus-Proper)(lh.bankssts_3)(lh.inferiorparietal_4)(lh.inferiorparietal_5)
0.00533	2.00E-05	0.81227	0.90452	(lh.bankssts_3)(lh.inferiorparietal_4)(lh.inferiorparietal_5)
0.00533	2.00E-05	0.81227	0.90452	(lh.bankssts_3)(lh.inferiorparietal_4)(lh.inferiorparietal_5)(lh.isthmuscingulate_3)
0.00533	2.00E-05	0.81227	0.90452	(Left-Putamen)(lh.inferiorparietal_4)(lh.parahippocampal_3)(lh.precuneus_11)
0.00536	2.00E-05	0.76173	0.86432	(Brain_Stem)(lh.inferiorparietal_4)(lh.inferiorparietal_5)(lh.lingual_8)
0.00536	2.00E-05	0.76173	0.86432	(lh.inferiorparietal_4)(lh.lingual_7)(lh.lingual_8)(lh.precuneus_11)
0.00536	2.00E-05	0.76173	0.86432	(lh.fusiform_5)(lh.precuneus_11)(lh.supramarginal_1)(lh.transversetemporal_2)
0.00536	2.00E-05	0.76173	0.86432	(Brain_Stem)(Left-Pallidum)(lh.inferiorparietal_4)(lh.precuneus_11)
0.00536	2.00E-05	0.76173	0.86432	(lh.inferiorparietal_5)(lh.lingual_6)(lh.supramarginal_1)(lh.transversetemporal_2)
0.00544	2.00E-05	0.72563	0.83417	(lh.inferiorparietal_5)(lh.parahippocampal_2)(lh.precuneus_11)(lh.supramarginal_1)

0.00544	2.00E-05	0.72563	0.83417	(lh.fusiform_7)(lh.insula_4)(lh.precuneus_11)(lh.supramarginal_1)
0.00544	2.00E-05	0.72563	0.83417	(lh.insula_4)(lh.parahippocampal_1)(lh.precuneus_11)(lh.supramarginal_1)
0.00544	2.00E-05	0.72563	0.83417	(lh.fusiform_7)(lh.parahippocampal_1)(lh.precuneus_11)(lh.supramarginal_1)
0.00546	2.00E-05	0.74368	0.84925	(lh.fusiform_5)(lh.inferiorparietal_4)(lh.inferiorparietal_5)(lh.superiortemporal_8)
0.00546	2.00E-05	0.74368	0.84925	(lh.fusiform_6)(lh.inferiorparietal_4)(lh.inferiorparietal_5)
0.00546	2.00E-05	0.74368	0.84925	(lh.lingual_7)(lh.precuneus_11)(lh.supramarginal_1)
0.00546	2.00E-05	0.74368	0.84925	(lh.parahippocampal_3)(lh.precuneus_11)(lh.supramarginal_1)(lh.transversetemporal_2)
0.00546	2.00E-05	0.74368	0.84925	(lh.fusiform_6)(lh.inferiorparietal_4)(lh.insula_2)(lh.precuneus_11)
0.00546	2.00E-05	0.74368	0.84925	(Brain_Stem)(lh.precuneus_11)(lh.superiortemporal_3)(lh.supramarginal_1)
0.00546	2.00E-05	0.74368	0.84925	(lh.isthmuscingulate_3)(lh.lingual_7)(lh.precuneus_11)(lh.supramarginal_1)
0.00546	2.00E-05	0.74368	0.84925	(lh.lingual_7)(lh.precuneus_11)(lh.superiortemporal_2)(lh.supramarginal_1)
0.00546	2.00E-05	0.74368	0.84925	(Left-Thalamus-Proper)(lh.lingual_7)(lh.precuneus_11)(lh.supramarginal_1)
0.00546	2.00E-05	0.74368	0.84925	(Left-Amygdala)(Left-Pallidum)(lh.inferiorparietal_4)(lh.inferiorparietal_5)
0.00546	2.00E-05	0.74368	0.84925	(lh.fusiform_6)(lh.inferiorparietal_4)(lh.inferiorparietal_5)(lh.isthmuscingulate_3)
0.00546	2.00E-05	0.74368	0.84925	(Left-Putamen)(lh.fusiform_6)(lh.inferiorparietal_4)(lh.inferiorparietal_5)
0.00546	2.00E-05	0.74368	0.84925	(lh.inferiorparietal_4)(lh.lingual_8)(lh.parahippocampal_1)(lh.precuneus_11)
0.00546	2.00E-05	0.74368	0.84925	(Left-Thalamus-Proper)(lh.fusiform_6)(lh.inferiorparietal_4)(lh.inferiorparietal_5)
0.00546	2.00E-05	0.74368	0.84925	(Left-Amygdala)(lh.inferiorparietal_4)(lh.lingual_6)(lh.precuneus_11)
0.0055	2.00E-05	0.787	0.88442	(lh.fusiform_5)(lh.insula_2)(lh.precuneus_11)(lh.supramarginal_1)
0.00558	2.00E-05	0.77465	0.87437	(Left-Pallidum)(lh.inferiorparietal_4)(lh.lingual_6)(lh.transversetemporal_2)
0.00558	2.00E-05	0.77465	0.87437	(lh.bankssts_3)(lh.inferiorparietal_4)(lh.lingual_6)(lh.parahippocampal_3)
0.00558	2.00E-05	0.77465	0.87437	(lh.inferiorparietal_4)(lh.inferiorparietal_5)(lh.parahippocampal_3)(lh.transversetemporal_
0.00558	2.00E-05	0.77465	0.87437	(lh.bankssts_3)(lh.inferiorparietal_4)(lh.lingual_8)(lh.parahippocampal_3)
0.00558	2.00E-05	0.77465	0.87437	(lh.bankssts_3)(lh.inferiorparietal_4)(lh.lingual_8)(lh.transversetemporal_2)
0.00558	2.00E-05	0.77465	0.87437	(lh.bankssts_3)(lh.inferiorparietal_4)(lh.lingual_6)(lh.transversetemporal_2)
0.00562	2.00E-05	0.81949	0.90955	(Left-Thalamus-Proper)(lh.bankssts_3)(lh.inferiorparietal_4)(lh.precuneus_11)
0.00562	2.00E-05	0.81949	0.90955	(lh.bankssts_3)(lh.inferiorparietal_4)(lh.precuneus_11)
0.00562	2.00E-05	0.81949	0.90955	(lh.inferiorparietal_4)(lh.inferiorparietal_5)(lh.lingual_8)(lh.superiortemporal_2)
0.00562	2.00E-05	0.81949	0.90955	(lh.bankssts_3)(lh.inferiorparietal_4)(lh.isthmuscingulate_3)(lh.precuneus_11)
0.00565	2.00E-05	0.91336	0.97487	(Left-Thalamus-Proper)(lh.inferiorparietal_5)(lh.precuneus_11)(lh.superiortemporal_3)
0.00565	2.00E-05	0.91336	0.97487	(lh.inferiorparietal_5)(lh.precuneus_11)(lh.superiortemporal_2)(lh.superiortemporal_3)
0.00565	2.00E-05	0.91336	0.97487	(lh.inferiorparietal_5)(lh.lingual_6)(lh.precuneus_11)(lh.superiortemporal_2)
0.00565	2.00E-05	0.91336	0.97487	(lh.inferiorparietal_5)(lh.precuneus_11)(lh.superiortemporal_3)
0.00565	2.00E-05	0.91336	0.97487	(lh.fusiform_5)(lh.inferiorparietal_5)(lh.insula_2)(lh.precuneus_11)
0.00565	2.00E-05	0.91336	0.97487	(lh.inferiorparietal_5)(lh.isthmuscingulate_3)(lh.precuneus_11)(lh.superiortemporal_3)
0.0058	2.00E-05	0.76895	0.86935	(Left-Putamen)(lh.superiortemporal_3)(lh.supramarginal_1)(lh.transversetemporal_2)

0.0058	2.00E-05	0.76895	0.86935	(Left-Pallidum)(lh.fusiform_5)(lh.precuneus_11)(lh.supramarginal_1)
0.0058	2.00E-05	0.76895	0.86935	(Left-Caudate)(lh.inferiorparietal_4)(lh.inferiorparietal_5)(lh.precuneus_11)
0.0058	2.00E-05	0.76895	0.86935	(Left-Thalamus-Proper)(lh.superiortemporal_3)(lh.supramarginal_1)(lh.transversetemporal
0.00584	2.00E-05	0.7148	0.82412	(lh.inferiorparietal_4)(lh.inferiorparietal_5)(lh.parahippocampal_2)(lh.superiortemporal_8)
0.00584	2.00E-05	0.7148	0.82412	(lh.inferiorparietal_5)(lh.lingual_7)(lh.supramarginal_1)(lh.transversetemporal_2)
0.00584	2.00E-05	0.7148	0.82412	(lh.fusiform_6)(lh.isthmuscingulate_3)(lh.precuneus_11)(lh.supramarginal_1)
0.00584	2.00E-05	0.7148	0.82412	(Left-Putamen)(lh.fusiform_6)(lh.precuneus_11)(lh.supramarginal_1)
0.00584	2.00E-05	0.7148	0.82412	(lh.fusiform_6)(lh.precuneus_11)(lh.superiortemporal_2)(lh.supramarginal_1)
0.00584	2.00E-05	0.7148	0.82412	(lh.fusiform_6)(lh.inferiorparietal_4)(lh.inferiorparietal_5)(lh.transversetemporal_2)
0.00584	2.00E-05	0.7148	0.82412	(lh.fusiform_6)(lh.precuneus_11)(lh.supramarginal_1)
0.00584	2.00E-05	0.7148	0.82412	(Left-Thalamus-Proper)(lh.fusiform_6)(lh.precuneus_11)(lh.supramarginal_1)
0.00588	2.00E-05	0.79422	0.88945	(Left-Thalamus-Proper)(lh.inferiorparietal_5)(lh.superiortemporal_3)(lh.supramarginal_1)
0.00588	2.00E-05	0.79422	0.88945	(lh.lingual_6)(lh.precuneus_11)(lh.superiortemporal_2)(lh.supramarginal_1)
0.00588	2.00E-05	0.79422	0.88945	(lh.inferiorparietal_5)(lh.superiortemporal_3)(lh.supramarginal_1)
0.00588	2.00E-05	0.79422	0.88945	(lh.lingual_6)(lh.precuneus_11)(lh.supramarginal_1)
0.00588	2.00E-05	0.79422	0.88945	(lh.inferiorparietal_4)(lh.inferiorparietal_5)(lh.insula_1)(lh.precuneus_11)
0.00588	2.00E-05	0.79422	0.88945	(lh.inferiorparietal_5)(lh.superiortemporal_2)(lh.superiortemporal_3)(lh.supramarginal_1)
0.00588	2.00E-05	0.79422	0.88945	(Left-Thalamus-Proper)(lh.lingual_6)(lh.precuneus_11)(lh.supramarginal_1)
0.00588	2.00E-05	0.79422	0.88945	(Left-Putamen)(lh.fusiform_5)(lh.precuneus_11)(lh.supramarginal_1)
0.00588	2.00E-05	0.79422	0.88945	(lh.isthmuscingulate_3)(lh.lingual_6)(lh.precuneus_11)(lh.supramarginal_1)
0.00588	2.00E-05	0.79422	0.88945	(Left-Putamen)(lh.inferiorparietal_5)(lh.superiortemporal_3)(lh.supramarginal_1)
0.00588	2.00E-05	0.79422	0.88945	(lh.inferiorparietal_5)(lh.isthmuscingulate_3)(lh.superiortemporal_3)(lh.supramarginal_1)
0.00588	2.00E-05	0.79422	0.88945	(Left-Pallidum)(lh.inferiorparietal_4)(lh.inferiorparietal_5)(lh.superiortemporal_3)
0.00588	2.00E-05	0.79422	0.88945	(lh.inferiorparietal_4)(lh.precuneus_11)(lh.superiortemporal_3)(lh.transversetemporal_2)
0.0059	3.00E-05	0.82671	0.91457	(lh.inferiorparietal_4)(lh.insula_2)(lh.lingual_6)
0.0059	3.00E-05	0.82671	0.91457	(Left-Thalamus-Proper)(lh.inferiorparietal_4)(lh.insula_2)(lh.lingual_6)
0.0059	3.00E-05	0.82671	0.91457	(lh.inferiorparietal_5)(lh.lingual_6)(lh.precuneus_11)(lh.superiortemporal_8)
0.0059	3.00E-05	0.82671	0.91457	(lh.inferiorparietal_4)(lh.lingual_8)(lh.precuneus_11)(lh.superiortemporal_2)
0.0059	3.00E-05	0.82671	0.91457	(lh.inferiorparietal_4)(lh.insula_2)(lh.isthmuscingulate_3)(lh.lingual_6)
0.0059	3.00E-05	0.82671	0.91457	(Left-Putamen)(lh.inferiorparietal_4)(lh.insula_2)(lh.lingual_6)
0.00592	3.00E-05	0.78169	0.8794	(lh.inferiorparietal_4)(lh.parahippocampal_3)(lh.precuneus_11)(lh.transversetemporal_2)
0.00592	3.00E-05	0.78169	0.8794	(Left-Pallidum)(lh.bankssts_3)(lh.fusiform_5)(lh.inferiorparietal_4)
0.00592	3.00E-05	0.78169	0.8794	(Left-Pallidum)(lh.inferiorparietal_4)(lh.inferiorparietal_5)(lh.parahippocampal_3)
0.00594	3.00E-05	0.7509	0.85427	(Left-Thalamus-Proper)(lh.fusiform_6)(lh.inferiorparietal_4)(lh.precuneus_11)
0.00594	3.00E-05	0.7509	0.85427	(lh.fusiform_6)(lh.inferiorparietal_4)(lh.precuneus_11)
0.00594	3.00E-05	0.7509	0.85427	(lh.bankssts_3)(lh.inferiorparietal_5)(lh.supramarginal_1)(lh.transversetemporal_2)

0.00594	3.00E-05	0.7509	0.85427	(lh.fusiform_5)(lh.inferiorparietal_4)(lh.precuneus_11)(lh.superiortemporal_8)
0.00594	3.00E-05	0.7509	0.85427	(Brain_Stem)(lh.inferiorparietal_5)(lh.precuneus_11)(lh.supramarginal_1)
0.00594	3.00E-05	0.7509	0.85427	(Left-Putamen)(lh.fusiform_6)(lh.inferiorparietal_4)(lh.precuneus_11)
0.00594	3.00E-05	0.7509	0.85427	(lh.fusiform_6)(lh.inferiorparietal_4)(lh.isthmuscingulate_3)(lh.precuneus_11)
0.00594	3.00E-05	0.7509	0.85427	(Left-Pallidum)(lh.inferiorparietal_4)(lh.parahippocampal_1)(lh.precuneus_11)
0.00595	3.00E-05	0.73285	0.8392	(lh.inferiorparietal_4)(lh.insula_4)(lh.parahippocampal_1)(lh.superiortemporal_8)
0.00595	3.00E-05	0.73285	0.8392	(lh.inferiorparietal_4)(lh.inferiorparietal_5)(lh.lateraloccipital_9)(lh.precuneus_11)
0.00595	3.00E-05	0.73285	0.8392	(lh.inferiorparietal_4)(lh.inferiorparietal_5)(lh.insula_4)(lh.superiortemporal_8)
0.00595	3.00E-05	0.73285	0.8392	(lh.entorhinal_1)(lh.fusiform_7)(lh.inferiorparietal_4)(lh.inferiorparietal_5)
0.00595	3.00E-05	0.73285	0.8392	(lh.fusiform_6)(lh.inferiorparietal_4)(lh.lingual_6)(lh.lingual_8)
0.00595	3.00E-05	0.73285	0.8392	(lh.inferiorparietal_5)(lh.lingual_7)(lh.superiortemporal_3)(lh.supramarginal_1)
0.00609	3.00E-05	0.93141	0.98492	(Left-Putamen)(lh.lingual_6)(lh.precuneus_11)
0.00609	3.00E-05	0.93141	0.98492	(Left-Putamen)(lh.isthmuscingulate_3)(lh.lingual_6)(lh.precuneus_11)
0.00609	3.00E-05	0.93141	0.98492	(Left-Putamen)(Left-Thalamus-Proper)(lh.lingual_6)(lh.precuneus_11)
0.00617	3.00E-05	0.83394	0.9196	(lh.inferiorparietal_4)(lh.inferiorparietal_5)(lh.superiortemporal_2)
0.00617	3.00E-05	0.83394	0.9196	(Left-Putamen)(lh.inferiorparietal_4)(lh.lingual_6)
0.00617	3.00E-05	0.83394	0.9196	(Left-Putamen)(Left-Thalamus-Proper)(lh.inferiorparietal_4)(lh.lingual_6)
0.00617	3.00E-05	0.83394	0.9196	(Left-Thalamus-Proper)(lh.inferiorparietal_4)(lh.inferiorparietal_5)(lh.superiortemporal_2)
0.00617	3.00E-05	0.83394	0.9196	(lh.inferiorparietal_4)(lh.inferiorparietal_5)(lh.isthmuscingulate_3)(lh.superiortemporal_2)
0.00617	3.00E-05	0.83394	0.9196	(Left-Putamen)(lh.inferiorparietal_4)(lh.isthmuscingulate_3)(lh.lingual_6)
0.00618	3.00E-05	0.90614	0.96985	(Left-Putamen)(lh.isthmuscingulate_3)(lh.precuneus_11)(lh.transversetemporal_2)
0.00618	3.00E-05	0.90614	0.96985	(Left-Putamen)(Left-Thalamus-Proper)(lh.inferiorparietal_5)(lh.transversetemporal_2)
0.00618	3.00E-05	0.90614	0.96985	(Left-Putamen)(lh.precuneus_11)(lh.transversetemporal_2)
0.00618	3.00E-05	0.90614	0.96985	(Left-Putamen)(lh.inferiorparietal_5)(lh.isthmuscingulate_3)(lh.transversetemporal_2)
0.00618	3.00E-05	0.90614	0.96985	(lh.fusiform_5)(lh.inferiorparietal_5)(lh.lingual_6)(lh.precuneus_11)
0.00618	3.00E-05	0.90614	0.96985	(Left-Putamen)(lh.inferiorparietal_5)(lh.transversetemporal_2)
0.00618	3.00E-05	0.90614	0.96985	(lh.inferiorparietal_5)(lh.insula_2)(lh.precuneus_11)(lh.superiortemporal_3)
0.00618	3.00E-05	0.90614	0.96985	(Left-Putamen)(Left-Thalamus-Proper)(lh.precuneus_11)(lh.transversetemporal_2)
0.00622	3.00E-05	0.70397	0.81407	(lh.fusiform_6)(lh.lingual_6)(lh.precuneus_11)(lh.supramarginal_1)
0.00625	3.00E-05	0.77617	0.87437	(Brain_Stem)(lh.inferiorparietal_4)(lh.insula_2)(lh.precuneus_11)
0.00625	3.00E-05	0.77617	0.87437	(Brain_Stem)(Left-Thalamus-Proper)(lh.inferiorparietal_4)(lh.inferiorparietal_5)
0.00627	3.00E-05	0.78873	0.88442	(lh.bankssts_3)(lh.inferiorparietal_4)(lh.isthmuscingulate_3)(lh.parahippocampal_3)
0.00627	3.00E-05	0.78873	0.88442	(Left-Putamen)(lh.inferiorparietal_5)(lh.lateraloccipital_9)(lh.transversetemporal_2)
0.00627	3.00E-05	0.78873	0.88442	(lh.bankssts_3)(lh.inferiorparietal_4)(lh.transversetemporal_2)
0.00627	3.00E-05	0.78873	0.88442	(Left-Pallidum)(lh.inferiorparietal_4)(lh.parahippocampal_3)(lh.precuneus_11)
0.00627	3.00E-05	0.78873	0.88442	(lh.bankssts_3)(lh.inferiorparietal_4)(lh.parahippocampal_3)

0.00627	3.00E-05	0.78873	0.88442	(lh.bankssts_3)(lh.inferiorparietal_4)(lh.isthmuscingulate_3)(lh.transversetemporal_2)
0.00627	3.00E-05	0.80144	0.89447	(lh.inferiorparietal_4)(lh.inferiorparietal_5)(lh.superiortemporal_2)(lh.transversetemporal_
0.00627	3.00E-05	0.80144	0.89447	(lh.inferiorparietal_4)(lh.lingual_8)(lh.parahippocampal_3)(lh.precuneus_11)
0.00627	3.00E-05	0.80144	0.89447	(Left-Pallidum)(lh.inferiorparietal_4)(lh.precuneus_11)(lh.superiortemporal_3)
0.00627	3.00E-05	0.80144	0.89447	(lh.inferiorparietal_4)(lh.inferiorparietal_5)(lh.isthmuscingulate_2)(lh.lingual_6)
0.00641	3.00E-05	0.72202	0.82915	(lh.inferiorparietal_4)(lh.lingual_6)(lh.parahippocampal_1)(lh.superiortemporal_8)
0.00641	3.00E-05	0.72202	0.82915	(lh.bankssts_3)(lh.fusiform_6)(lh.inferiorparietal_4)(lh.inferiorparietal_5)
0.00641	3.00E-05	0.72202	0.82915	(lh.lingual_7)(lh.parahippocampal_3)(lh.precuneus_11)(lh.supramarginal_1)
0.00641	3.00E-05	0.72202	0.82915	(lh.inferiorparietal_4)(lh.inferiorparietal_5)(lh.lateraloccipital_9)(lh.lingual_6)
0.00641	3.00E-05	0.72202	0.82915	(lh.fusiform_6)(lh.inferiorparietal_4)(lh.precuneus_11)(lh.transversetemporal_2)
0.00641	3.00E-05	0.84116	0.92462	(lh.inferiorparietal_5)(lh.insula_2)(lh.lingual_7)(lh.precuneus_11)
0.00641	3.00E-05	0.84116	0.92462	(lh.inferiorparietal_4)(lh.precuneus_11)(lh.superiortemporal_2)
0.00641	3.00E-05	0.84116	0.92462	(lh.fusiform_7)(lh.insula_4)(lh.parahippocampal_1)(lh.precuneus_11)
0.00641	3.00E-05	0.84116	0.92462	(Left-Thalamus-Proper)(lh.inferiorparietal_4)(lh.precuneus_11)(lh.superiortemporal_2)
0.00641	3.00E-05	0.84116	0.92462	(lh.inferiorparietal_4)(lh.isthmuscingulate_3)(lh.precuneus_11)(lh.superiortemporal_2)
0.00645	3.00E-05	0.75812	0.8593	(Left-Pallidum)(lh.insula_2)(lh.supramarginal_1)(lh.transversetemporal_2)
0.00645	3.00E-05	0.75812	0.8593	(Left-Thalamus-Proper)(lh.inferiorparietal_4)(lh.parahippocampal_1)(lh.precuneus_11)
0.00645	3.00E-05	0.75812	0.8593	(lh.lingual_6)(lh.superiortemporal_3)(lh.supramarginal_1)(lh.transversetemporal_2)
0.00645	3.00E-05	0.75812	0.8593	(Left-Putamen)(lh.inferiorparietal_4)(lh.parahippocampal_1)(lh.precuneus_11)
0.00645	3.00E-05	0.75812	0.8593	(Brain_Stem)(lh.inferiorparietal_4)(lh.inferiorparietal_5)(lh.superiortemporal_3)
0.00645	3.00E-05	0.75812	0.8593	(lh.inferiorparietal_4)(lh.insula_2)(lh.parahippocampal_1)(lh.precuneus_11)
0.00649	3.00E-05	0.74007	0.84422	(lh.entorhinal_1)(lh.fusiform_7)(lh.inferiorparietal_4)(lh.precuneus_11)
0.00649	3.00E-05	0.74007	0.84422	(lh.fusiform_7)(lh.inferiorparietal_4)(lh.lingual_6)(lh.superiortemporal_8)
0.00649	3.00E-05	0.74007	0.84422	(lh.inferiorparietal_4)(lh.inferiorparietal_5)(lh.lingual_8)(lh.parahippocampal_1)
0.00649	3.00E-05	0.74007	0.84422	(Left-Amygdala)(lh.inferiorparietal_4)(lh.inferiorparietal_5)(lh.lingual_8)
0.00649	3.00E-05	0.74007	0.84422	(lh.inferiorparietal_4)(lh.insula_4)(lh.precuneus_11)(lh.superiortemporal_8)
0.00649	3.00E-05	0.74007	0.84422	(Brain_Stem)(Left-Pallidum)(lh.precuneus_11)(lh.supramarginal_1)
0.00662	3.00E-05	0.79577	0.88945	(lh.inferiorparietal_4)(lh.inferiorparietal_5)(lh.lingual_8)(lh.parahippocampal_3)
0.00662	3.00E-05	0.79577	0.88945	(lh.inferiorparietal_4)(lh.insula_2)(lh.lingual_6)(lh.transversetemporal_2)
0.00662	3.00E-05	0.79577	0.88945	(lh.inferiorparietal_4)(lh.inferiorparietal_5)(lh.lingual_8)(lh.transversetemporal_2)
0.00662	3.00E-05	0.79577	0.88945	(lh.bankssts_3)(lh.inferiorparietal_4)(lh.lingual_6)(lh.lingual_8)
0.00662	3.00E-05	0.79577	0.88945	(Left-Pallidum)(lh.inferiorparietal_4)(lh.lingual_6)(lh.lingual_8)
0.00664	3.00E-05	0.84838	0.92965	(Left-Putamen)(lh.inferiorparietal_5)(lh.lingual_7)(lh.precuneus_11)
0.00667	3.00E-05	0.80866	0.8995	(Left-Pallidum)(lh.inferiorparietal_4)(lh.lingual_6)
0.00667	3.00E-05	0.80866	0.8995	(lh.inferiorparietal_5)(lh.precuneus_11)(lh.superiortemporal_8)(lh.transversetemporal_2)
0.00667	3.00E-05	0.80866	0.8995	(lh.inferiorparietal_4)(lh.precuneus_11)(lh.superiortemporal_2)(lh.transversetemporal_2)

0.00667	3.00E-05	0.80866	0.8995	(Left-Pallidum)(Left-Thalamus-Proper)(lh.inferiorparietal_4)(lh.lingual_6)
0.00667	3.00E-05	0.80866	0.8995	(lh.inferiorparietal_4)(lh.inferiorparietal_5)(lh.insula_2)(lh.isthmuscingulate_2)
0.00667	3.00E-05	0.80866	0.8995	(Left-Pallidum)(lh.inferiorparietal_4)(lh.isthmuscingulate_3)(lh.lingual_6)
0.00667	3.00E-05	0.80866	0.8995	(lh.inferiorparietal_4)(lh.inferiorparietal_5)(lh.isthmuscingulate_3)(lh.parahippocampal_3)
0.00667	3.00E-05	0.80866	0.8995	(Left-Pallidum)(Left-Putamen)(lh.inferiorparietal_4)(lh.lingual_6)
0.00667	3.00E-05	0.80866	0.8995	(Left-Thalamus-Proper)(lh.inferiorparietal_4)(lh.inferiorparietal_5)(lh.parahippocampal_3)
0.00667	3.00E-05	0.80866	0.8995	(lh.inferiorparietal_4)(lh.isthmuscingulate_2)(lh.lingual_6)(lh.precuneus_11)
0.00667	3.00E-05	0.80866	0.8995	(lh.inferiorparietal_4)(lh.inferiorparietal_5)(lh.parahippocampal_3)
0.00673	3.00E-05	0.78339	0.8794	(Left-Putamen)(lh.superiortemporal_2)(lh.supramarginal_1)(lh.transversetemporal_2)
0.00673	3.00E-05	0.78339	0.8794	(lh.inferiorparietal_5)(lh.lingual_6)(lh.superiortemporal_3)(lh.supramarginal_1)
0.00673	3.00E-05	0.78339	0.8794	(Left-Thalamus-Proper)(lh.bankssts_3)(lh.precuneus_11)(lh.supramarginal_1)
0.00673	3.00E-05	0.78339	0.8794	(lh.bankssts_3)(lh.isthmuscingulate_3)(lh.precuneus_11)(lh.supramarginal_1)
0.00673	3.00E-05	0.78339	0.8794	(Left-Putamen)(Left-Thalamus-Proper)(lh.supramarginal_1)(lh.transversetemporal_2)
0.00673	3.00E-05	0.78339	0.8794	(lh.bankssts_3)(lh.precuneus_11)(lh.supramarginal_1)
0.00673	3.00E-05	0.78339	0.8794	(lh.bankssts_3)(lh.precuneus_11)(lh.superiortemporal_2)(lh.supramarginal_1)
0.00673	3.00E-05	0.78339	0.8794	(Left-Putamen)(lh.supramarginal_1)(lh.transversetemporal_2)
0.00673	3.00E-05	0.78339	0.8794	(Brain_Stem)(Left-Putamen)(lh.inferiorparietal_4)(lh.precuneus_11)
0.00673	3.00E-05	0.78339	0.8794	(Left-Putamen)(lh.isthmuscingulate_3)(lh.supramarginal_1)(lh.transversetemporal_2)
0.00684	3.00E-05	0.71119	0.8191	(lh.fusiform_5)(lh.fusiform_6)(lh.precuneus_11)(lh.supramarginal_1)
0.00684	3.00E-05	0.71119	0.8191	(lh.lingual_7)(lh.superiortemporal_3)(lh.supramarginal_1)(lh.transversetemporal_2)
0.00685	3.00E-05	0.8917	0.9598	(lh.inferiorparietal_5)(lh.lingual_6)(lh.transversetemporal_2)
0.00685	3.00E-05	0.8917	0.9598	(Left-Thalamus-Proper)(lh.inferiorparietal_5)(lh.lingual_6)(lh.transversetemporal_2)
0.00685	3.00E-05	0.8917	0.9598	(lh.lingual_6)(lh.precuneus_11)(lh.transversetemporal_2)
0.00685	3.00E-05	0.8917	0.9598	(lh.isthmuscingulate_3)(lh.lingual_6)(lh.precuneus_11)(lh.transversetemporal_2)
0.00685	3.00E-05	0.8917	0.9598	(lh.bankssts_3)(lh.inferiorparietal_5)(lh.lingual_6)(lh.precuneus_11)
0.00685	3.00E-05	0.8917	0.9598	(Left-Thalamus-Proper)(lh.lingual_6)(lh.precuneus_11)(lh.transversetemporal_2)
0.00685	3.00E-05	0.8917	0.9598	(lh.inferiorparietal_5)(lh.isthmuscingulate_3)(lh.lingual_6)(lh.transversetemporal_2)
0.00697	3.00E-05	0.86282	0.9397	(lh.inferiorparietal_5)(lh.parahippocampal_3)(lh.precuneus_11)(lh.transversetemporal_2)
0.00697	3.00E-05	0.86282	0.9397	(Left-Pallidum)(lh.lingual_6)(lh.precuneus_11)(lh.transversetemporal_2)
0.00697	3.00E-05	0.86282	0.9397	(Left-Pallidum)(lh.inferiorparietal_5)(lh.lingual_6)(lh.transversetemporal_2)
0.00699	3.00E-05	0.76534	0.86432	(lh.inferiorparietal_4)(lh.inferiorparietal_5)(lh.lingual_7)(lh.superiortemporal_2)
0.00699	4.00E-05	0.76534	0.86432	(lh.insula_2)(lh.superiortemporal_3)(lh.supramarginal_1)(lh.transversetemporal_2)
0.00701	4.00E-05	0.88448	0.95477	(lh.fusiform_5)(lh.inferiorparietal_5)(lh.precuneus_11)(lh.transversetemporal_2)
0.00702	4.00E-05	0.72924	0.83417	(lh.bankssts_3)(lh.fusiform_6)(lh.inferiorparietal_4)(lh.precuneus_11)
0.00702	4.00E-05	0.72924	0.83417	(Left-Caudate)(lh.precuneus_11)(lh.supramarginal_1)(lh.transversetemporal_2)
0.00702	4.00E-05	0.72924	0.83417	(lh.inferiorparietal_5)(lh.insula_4)(lh.precuneus_11)(lh.supramarginal_1)

0.00702	4.00E-05	0.72924	0.83417	(Brain_Stem)(lh.precuneus_11)(lh.supramarginal_1)(lh.transversetemporal_2)
0.00702	4.00E-05	0.72924	0.83417	(lh.inferiorparietal_4)(lh.insula_1)(lh.precuneus_11)(lh.superiortemporal_8)
0.00702	4.00E-05	0.72924	0.83417	(lh.inferiorparietal_4)(lh.inferiorparietal_5)(lh.insula_2)(lh.lateraloccipital_9)
0.00702	4.00E-05	0.72924	0.83417	(lh.inferiorparietal_4)(lh.parahippocampal_1)(lh.precuneus_11)(lh.transversetemporal_2)
0.00702	4.00E-05	0.72924	0.83417	(lh.inferiorparietal_4)(lh.inferiorparietal_5)(lh.lateraloccipital_9)(lh.lingual_8)
0.00702	4.00E-05	0.72924	0.83417	(Left-Amygdala)(lh.inferiorparietal_5)(lh.precuneus_11)(lh.supramarginal_1)
0.00705	4.00E-05	0.87004	0.94472	(lh.inferiorparietal_5)(lh.lingual_8)(lh.precuneus_11)(lh.transversetemporal_2)
0.00707	4.00E-05	0.87726	0.94975	(Left-Pallidum)(lh.bankssts_3)(lh.inferiorparietal_5)(lh.precuneus_11)
0.00708	4.00E-05	0.74729	0.84925	(Left-Pallidum)(lh.inferiorparietal_4)(lh.lingual_6)(lh.superiortemporal_8)
0.00708	4.00E-05	0.74729	0.84925	(lh.fusiform_7)(lh.inferiorparietal_4)(lh.lingual_6)(lh.parahippocampal_1)
0.00708	4.00E-05	0.74729	0.84925	(lh.bankssts_3)(lh.superiortemporal_3)(lh.supramarginal_1)(lh.transversetemporal_2)
0.00708	4.00E-05	0.74729	0.84925	(Brain_Stem)(lh.inferiorparietal_4)(lh.inferiorparietal_5)(lh.transversetemporal_2)
0.00708	4.00E-05	0.74729	0.84925	(lh.inferiorparietal_4)(lh.insula_4)(lh.lingual_6)(lh.parahippocampal_1)
0.00708	4.00E-05	0.74729	0.84925	(Left-Pallidum)(lh.inferiorparietal_4)(lh.inferiorparietal_5)(lh.parahippocampal_1)
0.00708	4.00E-05	0.81588	0.90452	(lh.inferiorparietal_4)(lh.insula_2)(lh.isthmuscingulate_2)(lh.precuneus_11)
0.00708	4.00E-05	0.81588	0.90452	(Left-Thalamus-Proper)(lh.inferiorparietal_4)(lh.parahippocampal_3)(lh.precuneus_11)
0.00708	4.00E-05	0.81588	0.90452	(lh.inferiorparietal_4)(lh.isthmuscingulate_3)(lh.parahippocampal_3)(lh.precuneus_11)
0.00708	4.00E-05	0.81588	0.90452	(lh.inferiorparietal_4)(lh.insula_2)(lh.lingual_6)(lh.lingual_8)
0.00708	4.00E-05	0.81588	0.90452	(Left-Putamen)(lh.inferiorparietal_4)(lh.inferiorparietal_5)(lh.isthmuscingulate_2)
0.00708	4.00E-05	0.81588	0.90452	(lh.inferiorparietal_4)(lh.parahippocampal_3)(lh.precuneus_11)
0.0072	4.00E-05	0.92419	0.9799	(lh.insula_2)(lh.isthmuscingulate_3)(lh.lingual_6)(lh.precuneus_11)
0.0072	4.00E-05	0.92419	0.9799	(Left-Thalamus-Proper)(lh.insula_2)(lh.lingual_6)(lh.precuneus_11)
0.0072	4.00E-05	0.92419	0.9799	(lh.insula_2)(lh.lingual_6)(lh.precuneus_11)
0.0072	4.00E-05	0.92419	0.9799	(Left-Putamen)(lh.insula_2)(lh.lingual_6)(lh.precuneus_11)
0.00722	4.00E-05	0.79061	0.88442	(Left-Pallidum)(lh.inferiorparietal_4)(lh.inferiorparietal_5)(lh.isthmuscingulate_2)
0.00722	4.00E-05	0.79061	0.88442	(lh.inferiorparietal_5)(lh.insula_2)(lh.superiortemporal_3)(lh.supramarginal_1)
0.00722	4.00E-05	0.79061	0.88442	(lh.fusiform_6)(lh.inferiorparietal_5)(lh.precuneus_11)(lh.transversetemporal_2)
0.00748	4.00E-05	0.8231	0.90955	(lh.bankssts_3)(lh.inferiorparietal_5)(lh.precuneus_11)(lh.superiortemporal_8)
0.00748	4.00E-05	0.8231	0.90955	(Left-Pallidum)(lh.inferiorparietal_5)(lh.lingual_7)(lh.precuneus_11)
0.00748	4.00E-05	0.8231	0.90955	(Left-Putamen)(lh.inferiorparietal_4)(lh.isthmuscingulate_2)(lh.precuneus_11)
0.00748	4.00E-05	0.8231	0.90955	(Left-Putamen)(lh.inferiorparietal_4)(lh.lingual_6)(lh.lingual_8)
0.00751	4.00E-05	0.71841	0.82412	(lh.bankssts_3)(lh.inferiorparietal_4)(lh.parahippocampal_1)(lh.superiortemporal_8)
0.00751	4.00E-05	0.71841	0.82412	(Brain_Stem)(lh.inferiorparietal_4)(lh.inferiorparietal_5)(lh.lingual_7)
0.00756	4.00E-05	0.77256	0.86935	(Left-Putamen)(lh.lingual_6)(lh.supramarginal_1)(lh.transversetemporal_2)
0.00756	4.00E-05	0.77256	0.86935	(lh.isthmuscingulate_2)(lh.precuneus_11)(lh.superiortemporal_3)(lh.supramarginal_1)
0.00756	4.00E-05	0.77256	0.86935	(lh.lingual_8)(lh.precuneus_11)(lh.superiortemporal_3)(lh.supramarginal_1)

0.00756	4.00E-05	0.77256	0.86935	(lh.inferiorparietal_4)(lh.lingual_7)(lh.precuneus_11)(lh.superiortemporal_2)
0.00756	4.00E-05	0.77256	0.86935	(Brain_Stem)(lh.inferiorparietal_4)(lh.lingual_8)(lh.precuneus_11)
0.00761	4.00E-05	0.95307	0.99497	(Left-Putamen)(Left-Thalamus-Proper)(lh.isthmuscingulate_3)(lh.precuneus_11)
0.00761	4.00E-05	0.95307	0.99497	(Left-Putamen)(Left-Thalamus-Proper)(lh.precuneus_11)
0.00761	4.00E-05	0.95307	0.99497	(Left-Putamen)(lh.isthmuscingulate_3)(lh.precuneus_11)
0.00761	4.00E-05	0.95307	0.99497	(Left-Putamen)(lh.precuneus_11)
0.00767	4.00E-05	0.73646	0.8392	(lh.bankssts_3)(lh.inferiorparietal_4)(lh.lingual_6)(lh.superiortemporal_8)
0.00767	4.00E-05	0.73646	0.8392	(Left-Putamen)(lh.inferiorparietal_4)(lh.inferiorparietal_5)(lh.lateraloccipital_9)
0.00767	4.00E-05	0.73646	0.8392	(lh.fusiform_7)(lh.insula_4)(lh.parahippocampal_1)(lh.supramarginal_1)
0.00767	4.00E-05	0.73646	0.8392	(lh.inferiorparietal_4)(lh.inferiorparietal_5)(lh.isthmuscingulate_2)(lh.superiortemporal_8)
0.00767	4.00E-05	0.73646	0.8392	(lh.bankssts_3)(lh.inferiorparietal_4)(lh.parahippocampal_1)(lh.precuneus_11)
0.00767	4.00E-05	0.73646	0.8392	(lh.fusiform_6)(lh.inferiorparietal_4)(lh.insula_2)(lh.lingual_6)
0.00767	4.00E-05	0.73646	0.8392	(lh.lingual_6)(lh.lingual_7)(lh.precuneus_11)(lh.supramarginal_1)
0.00769	4.00E-05	0.76056	0.8593	(lh.bankssts_3)(lh.fusiform_6)(lh.lingual_8)(lh.transversetemporal_2)
0.0077	4.00E-05	0.75451	0.85427	(Left-Amygdala)(lh.inferiorparietal_4)(lh.inferiorparietal_5)
0.0077	4.00E-05	0.75451	0.85427	(Brain_Stem)(Left-Amygdala)(lh.inferiorparietal_4)(lh.inferiorparietal_5)
0.0077	4.00E-05	0.75451	0.85427	(Left-Thalamus-Proper)(lh.inferiorparietal_4)(lh.lingual_6)(lh.superiortemporal_8)
0.0077	4.00E-05	0.75451	0.85427	(lh.inferiorparietal_4)(lh.isthmuscingulate_3)(lh.lingual_6)(lh.superiortemporal_8)
0.0077	4.00E-05	0.75451	0.85427	(Left-Amygdala)(lh.inferiorparietal_4)(lh.inferiorparietal_5)(lh.isthmuscingulate_3)
0.0077	4.00E-05	0.75451	0.85427	(lh.inferiorparietal_4)(lh.insula_2)(lh.lingual_6)(lh.superiortemporal_8)
0.0077	4.00E-05	0.75451	0.85427	(Left-Putamen)(lh.inferiorparietal_4)(lh.lingual_6)(lh.superiortemporal_8)
0.0077	4.00E-05	0.75451	0.85427	(Left-Amygdala)(lh.inferiorparietal_4)(lh.inferiorparietal_5)(lh.insula_2)
0.0077	5.00E-05	0.75451	0.85427	(Left-Putamen)(lh.inferiorparietal_4)(lh.inferiorparietal_5)(lh.parahippocampal_1)
0.0077	5.00E-05	0.75451	0.85427	(lh.inferiorparietal_4)(lh.inferiorparietal_5)(lh.insula_2)(lh.parahippocampal_1)
0.0077	5.00E-05	0.75451	0.85427	(lh.inferiorparietal_4)(lh.lingual_6)(lh.superiortemporal_8)
0.0077	5.00E-05	0.75451	0.85427	(Left-Caudate)(lh.inferiorparietal_5)(lh.precuneus_11)(lh.supramarginal_1)
0.0077	5.00E-05	0.75451	0.85427	(Left-Amygdala)(Left-Thalamus-Proper)(lh.inferiorparietal_4)(lh.inferiorparietal_5)
0.0077	5.00E-05	0.75451	0.85427	(Left-Amygdala)(Left-Putamen)(lh.inferiorparietal_4)(lh.inferiorparietal_5)
0.0077	5.00E-05	0.75451	0.85427	(Left-Amygdala)(Left-Pallidum)(lh.inferiorparietal_4)(lh.precuneus_11)
0.0077	5.00E-05	0.75451	0.85427	(lh.inferiorparietal_4)(lh.inferiorparietal_5)(lh.insula_4)(lh.lingual_8)
0.0077	5.00E-05	0.75451	0.85427	(Brain_Stem)(lh.insula_2)(lh.precuneus_11)(lh.supramarginal_1)
0.0077	5.00E-05	0.75451	0.85427	(lh.lingual_8)(lh.precuneus_11)(lh.supramarginal_1)(lh.transversetemporal_2)
0.0077	5.00E-05	0.75451	0.85427	(Left-Thalamus-Proper)(lh.inferiorparietal_4)(lh.inferiorparietal_5)(lh.parahippocampal_1)
0.00774	5.00E-05	0.79783	0.88945	(Left-Thalamus-Proper)(lh.fusiform_5)(lh.precuneus_11)(lh.supramarginal_1)
0.00774	5.00E-05	0.79783	0.88945	(lh.inferiorparietal_4)(lh.inferiorparietal_5)(lh.parahippocampal_3)(lh.superiortemporal_3)
0.00774	5.00E-05	0.79783	0.88945	(lh.fusiform_5)(lh.isthmuscingulate_3)(lh.precuneus_11)(lh.supramarginal_1)

0.00774	5.00E-05	0.79783	0.88945	(lh.fusiform_5)(lh.precuneus_11)(lh.supramarginal_1)
0.00774	5.00E-05	0.79783	0.88945	(lh.fusiform_5)(lh.inferiorparietal_4)(lh.inferiorparietal_5)(lh.transversetemporal_2)
0.00774	5.00E-05	0.79783	0.88945	(lh.fusiform_5)(lh.precuneus_11)(lh.superiortemporal_2)(lh.supramarginal_1)
0.00774	5.00E-05	0.79783	0.88945	(Left-Pallidum)(lh.inferiorparietal_4)(lh.isthmuscingulate_2)(lh.precuneus_11)
0.00787	5.00E-05	0.83032	0.91457	(lh.fusiform_5)(lh.inferiorparietal_4)(lh.inferiorparietal_5)
0.00787	5.00E-05	0.83032	0.91457	(lh.fusiform_7)(lh.inferiorparietal_5)(lh.precuneus_11)(lh.superiortemporal_8)
0.00787	5.00E-05	0.83032	0.91457	(lh.fusiform_5)(lh.inferiorparietal_4)(lh.inferiorparietal_5)(lh.isthmuscingulate_3)
0.00787	5.00E-05	0.83032	0.91457	(Left-Thalamus-Proper)(lh.fusiform_5)(lh.inferiorparietal_4)(lh.inferiorparietal_5)
0.00815	5.00E-05	0.77978	0.87437	(Left-Putamen)(lh.insula_2)(lh.supramarginal_1)(lh.transversetemporal_2)
0.00815	5.00E-05	0.77978	0.87437	(lh.inferiorparietal_4)(lh.insula_2)(lh.precuneus_11)(lh.superiortemporal_6)
0.00815	5.00E-05	0.77978	0.87437	(lh.insula_2)(lh.isthmuscingulate_3)(lh.supramarginal_1)(lh.transversetemporal_2)
0.00815	5.00E-05	0.77978	0.87437	(lh.insula_2)(lh.supramarginal_1)(lh.transversetemporal_2)
0.00815	5.00E-05	0.77978	0.87437	(Left-Putamen)(lh.inferiorparietal_4)(lh.precuneus_11)(lh.superiortemporal_6)
0.00815	5.00E-05	0.77978	0.87437	(lh.inferiorparietal_5)(lh.lingual_8)(lh.precuneus_11)(lh.supramarginal_1)
0.00815	5.00E-05	0.77978	0.87437	(Left-Thalamus-Proper)(lh.insula_2)(lh.supramarginal_1)(lh.transversetemporal_2)
0.00815	5.00E-05	0.77978	0.87437	(lh.inferiorparietal_5)(lh.isthmuscingulate_2)(lh.precuneus_11)(lh.supramarginal_1)
0.00815	5.00E-05	0.77978	0.87437	(lh.insula_2)(lh.superiortemporal_2)(lh.supramarginal_1)(lh.transversetemporal_2)
0.00823	5.00E-05	0.76761	0.86432	(lh.bankssts_3)(lh.fusiform_6)(lh.lingual_6)(lh.transversetemporal_2)
0.00823	5.00E-05	0.72563	0.82915	(lh.entorhinal_1)(lh.inferiorparietal_4)(lh.inferiorparietal_5)(lh.lingual_6)
0.00823	5.00E-05	0.72563	0.82915	(lh.inferiorparietal_5)(lh.precuneus_11)(lh.precuneus_6)(lh.transversetemporal_2)
0.00823	5.00E-05	0.72563	0.82915	(lh.inferiorparietal_4)(lh.lingual_8)(lh.parahippocampal_1)(lh.superiortemporal_8)
0.00823	5.00E-05	0.72563	0.82915	(lh.inferiorparietal_4)(lh.inferiorparietal_5)(lh.parahippocampal_1)(lh.parahippocampal_2)
0.00823	5.00E-05	0.72563	0.82915	(Left-Putamen)(lh.lingual_7)(lh.supramarginal_1)(lh.transversetemporal_2)
0.00823	5.00E-05	0.72563	0.82915	(lh.parahippocampal_2)(lh.precuneus_11)(lh.superiortemporal_3)(lh.supramarginal_1)
0.00823	5.00E-05	0.72563	0.82915	(Left-Pallidum)(lh.fusiform_6)(lh.inferiorparietal_4)(lh.inferiorparietal_5)
0.00823	5.00E-05	0.72563	0.82915	(lh.inferiorparietal_4)(lh.inferiorparietal_5)(lh.superiortemporal_6)(lh.superiortemporal_8)
0.00823	5.00E-05	0.72563	0.82915	(lh.inferiorparietal_4)(lh.inferiorparietal_5)(lh.parahippocampal_1)(lh.transversetemporal_
0.00823	6.00E-05	0.72563	0.82915	(lh.inferiorparietal_4)(lh.inferiorparietal_5)(lh.insula_1)(lh.superiortemporal_8)
0.00825	6.00E-05	0.83755	0.9196	(lh.fusiform_5)(lh.inferiorparietal_4)(lh.isthmuscingulate_3)(lh.precuneus_11)
0.00825	6.00E-05	0.83755	0.9196	(lh.inferiorparietal_5)(lh.insula_4)(lh.parahippocampal_1)(lh.precuneus_11)
0.00825	6.00E-05	0.83755	0.9196	(Left-Thalamus-Proper)(lh.fusiform_5)(lh.inferiorparietal_4)(lh.precuneus_11)
0.00825	6.00E-05	0.83755	0.9196	(lh.fusiform_5)(lh.inferiorparietal_4)(lh.precuneus_11)
0.00825	6.00E-05	0.83755	0.9196	(lh.inferiorparietal_5)(lh.lingual_6)(lh.lingual_7)(lh.precuneus_11)
0.00825	6.00E-05	0.83755	0.9196	(lh.fusiform_7)(lh.inferiorparietal_5)(lh.parahippocampal_1)(lh.precuneus_11)
0.00826	6.00E-05	0.80505	0.89447	(lh.inferiorparietal_4)(lh.inferiorparietal_5)(lh.isthmuscingulate_2)(lh.superiortemporal_3)
0.00826	6.00E-05	0.80505	0.89447	(lh.inferiorparietal_4)(lh.parahippocampal_3)(lh.precuneus_11)(lh.superiortemporal_3)

0.00826	6.00E-05	0.80505	0.89447	(lh.fusiform_5)(lh.inferiorparietal_4)(lh.precuneus_11)(lh.transversetemporal_2)
0.00836	6.00E-05	0.76173	0.8593	(Left-Pallidum)(lh.inferiorparietal_4)(lh.inferiorparietal_5)(lh.insula_4)
0.00836	6.00E-05	0.76173	0.8593	(lh.inferiorparietal_4)(lh.insula_2)(lh.lingual_6)(lh.lingual_7)
0.00836	6.00E-05	0.76173	0.8593	(Left-Putamen)(lh.bankssts_3)(lh.supramarginal_1)(lh.transversetemporal_2)
0.00836	6.00E-05	0.76173	0.8593	(Left-Pallidum)(Left-Thalamus-Proper)(lh.supramarginal_1)(lh.transversetemporal_2)
0.00836	6.00E-05	0.76173	0.8593	(Left-Pallidum)(Left-Putamen)(lh.supramarginal_1)(lh.transversetemporal_2)
0.00836	6.00E-05	0.76173	0.8593	(lh.fusiform_5)(lh.inferiorparietal_4)(lh.inferiorparietal_5)(lh.lingual_7)
0.00836	6.00E-05	0.76173	0.8593	(Brain_Stem)(Left-Putamen)(lh.precuneus_11)(lh.supramarginal_1)
0.00837	6.00E-05	0.74368	0.84422	(Left-Pallidum)(lh.inferiorparietal_4)(lh.lingual_6)(lh.lingual_7)
0.00837	6.00E-05	0.74368	0.84422	(lh.fusiform_6)(lh.inferiorparietal_4)(lh.inferiorparietal_5)(lh.superiortemporal_2)
0.00837	6.00E-05	0.74368	0.84422	(lh.inferiorparietal_4)(lh.lingual_6)(lh.lingual_8)(lh.superiortemporal_8)
0.00837	6.00E-05	0.74368	0.84422	(lh.fusiform_6)(lh.inferiorparietal_4)(lh.insula_2)(lh.lingual_8)
0.00837	6.00E-05	0.74368	0.84422	(Left-Thalamus-Proper)(lh.fusiform_6)(lh.inferiorparietal_4)(lh.lingual_6)
0.00837	6.00E-05	0.74368	0.84422	(Left-Putamen)(lh.fusiform_6)(lh.inferiorparietal_4)(lh.lingual_6)
0.00837	6.00E-05	0.74368	0.84422	(lh.fusiform_5)(lh.fusiform_6)(lh.inferiorparietal_4)(lh.inferiorparietal_5)
0.00837	6.00E-05	0.74368	0.84422	(lh.fusiform_6)(lh.inferiorparietal_4)(lh.isthmuscingulate_3)(lh.lingual_6)
0.00837	6.00E-05	0.74368	0.84422	(lh.fusiform_6)(lh.inferiorparietal_4)(lh.lingual_6)
0.00837	6.00E-05	0.74368	0.84422	(lh.inferiorparietal_4)(lh.isthmuscingulate_2)(lh.precuneus_11)(lh.superiortemporal_8)
0.00873	6.00E-05	0.73239	0.83417	(lh.inferiorparietal_4)(lh.isthmuscingulate_2)(lh.isthmuscingulate_3)(lh.supramarginal_1)
0.00873	7.00E-05	0.73239	0.83417	(lh.inferiorparietal_4)(lh.isthmuscingulate_2)(lh.supramarginal_1)
0.00874	7.00E-05	0.7148	0.8191	(lh.fusiform_6)(lh.inferiorparietal_4)(lh.lingual_6)(lh.transversetemporal_2)
0.00876	7.00E-05	0.90975	0.96985	(lh.bankssts_3)(lh.inferiorparietal_5)(lh.precuneus_11)
0.00876	7.00E-05	0.90975	0.96985	(Left-Thalamus-Proper)(lh.bankssts_3)(lh.inferiorparietal_5)(lh.precuneus_11)
0.00876	7.00E-05	0.90975	0.96985	(Left-Putamen)(lh.inferiorparietal_5)(lh.lingual_8)(lh.precuneus_11)
0.00876	7.00E-05	0.90975	0.96985	(lh.bankssts_3)(lh.inferiorparietal_5)(lh.isthmuscingulate_3)(lh.precuneus_11)
0.00878	7.00E-05	0.787	0.8794	(lh.fusiform_5)(lh.lingual_6)(lh.precuneus_11)(lh.supramarginal_1)
0.00878	7.00E-05	0.787	0.8794	(lh.fusiform_7)(lh.inferiorparietal_4)(lh.inferiorparietal_5)(lh.insula_2)
0.00878	7.00E-05	0.787	0.8794	(Left-Pallidum)(lh.inferiorparietal_5)(lh.insula_2)(lh.supramarginal_1)
0.00878	7.00E-05	0.787	0.8794	(lh.fusiform_5)(lh.inferiorparietal_5)(lh.superiortemporal_3)(lh.supramarginal_1)
0.00878	7.00E-05	0.787	0.8794	(Left-Thalamus-Proper)(lh.isthmuscingulate_3)(lh.supramarginal_1)(lh.transversetemporal_
0.00878	7.00E-05	0.787	0.8794	(lh.isthmuscingulate_3)(lh.superiortemporal_2)(lh.supramarginal_1)(lh.transversetempora
0.00878	7.00E-05	0.787	0.8794	(lh.supramarginal_1)(lh.transversetemporal_2)
0.00878	7.00E-05	0.787	0.8794	(Brain_Stem)(Left-Thalamus-Proper)(lh.inferiorparietal_4)(lh.precuneus_11)
0.00878	7.00E-05	0.787	0.8794	(lh.isthmuscingulate_3)(lh.supramarginal_1)(lh.transversetemporal_2)
0.00878	7.00E-05	0.787	0.8794	(Left-Thalamus-Proper)(lh.supramarginal_1)(lh.transversetemporal_2)
0.00878	7.00E-05	0.787	0.8794	(Left-Thalamus-Proper)(lh.superiortemporal_2)(lh.supramarginal_1)(lh.transversetemporal

0.00878	7.00E-05	0.787	0.8794	(lh.superiortemporal_2)(lh.supramarginal_1)(lh.transversetemporal_2)
0.00878	7.00E-05	0.77465	0.86935	(lh.bankssts_3)(lh.inferiorparietal_5)(lh.lateraloccipital_9)(lh.transversetemporal_2)
0.00878	7.00E-05	0.77465	0.86935	(lh.inferiorparietal_4)(lh.lingual_6)(lh.parahippocampal_3)(lh.transversetemporal_2)
0.00878	8.00E-05	0.77465	0.86935	(lh.bankssts_3)(lh.fusiform_6)(lh.inferiorparietal_5)(lh.transversetemporal_2)
0.0088	8.00E-05	0.81227	0.8995	(lh.fusiform_6)(lh.inferiorparietal_5)(lh.lingual_6)(lh.precuneus_11)
0.0088	8.00E-05	0.81227	0.8995	(Left-Pallidum)(lh.inferiorparietal_4)(lh.insula_2)(lh.lingual_8)
0.0088	8.00E-05	0.81227	0.8995	(lh.inferiorparietal_4)(lh.isthmuscingulate_2)(lh.precuneus_11)(lh.superiortemporal_3)
0.0088	8.00E-05	0.81227	0.8995	(lh.fusiform_5)(lh.inferiorparietal_4)(lh.inferiorparietal_5)(lh.superiortemporal_3)
0.0088	8.00E-05	0.81227	0.8995	(lh.bankssts_3)(lh.inferiorparietal_4)(lh.inferiorparietal_5)(lh.superiortemporal_2)
0.00892	8.00E-05	0.93502	0.98492	(Left-Putamen)(lh.inferiorparietal_5)(lh.lingual_6)
0.00892	8.00E-05	0.93502	0.98492	(Left-Putamen)(Left-Thalamus-Proper)(lh.inferiorparietal_5)(lh.lingual_6)
0.00892	8.00E-05	0.93502	0.98492	(Left-Putamen)(lh.inferiorparietal_5)(lh.isthmuscingulate_3)(lh.lingual_6)
0.00892	8.00E-05	0.93502	0.98492	(lh.inferiorparietal_5)(lh.isthmuscingulate_3)(lh.precuneus_11)(lh.superiortemporal_2)
0.00892	8.00E-05	0.93502	0.98492	(Left-Thalamus-Proper)(lh.inferiorparietal_5)(lh.precuneus_11)(lh.superiortemporal_2)
0.00892	8.00E-05	0.93502	0.98492	(lh.inferiorparietal_5)(lh.precuneus_11)(lh.superiortemporal_2)
0.009	8.00E-05	0.73285	0.83417	(Left-Pallidum)(lh.inferiorparietal_4)(lh.parahippocampal_1)(lh.superiortemporal_8)
0.009	8.00E-05	0.73285	0.83417	(lh.entorhinal_1)(lh.inferiorparietal_4)(lh.lingual_6)(lh.precuneus_11)
0.009	8.00E-05	0.73285	0.83417	(lh.fusiform_6)(lh.inferiorparietal_4)(lh.inferiorparietal_5)(lh.superiortemporal_3)
0.009	8.00E-05	0.73285	0.83417	(Left-Putamen)(lh.precuneus_11)(lh.precuneus_6)(lh.transversetemporal_2)
0.009	9.00E-05	0.73285	0.83417	(Left-Putamen)(lh.inferiorparietal_5)(lh.precuneus_6)(lh.transversetemporal_2)
0.009	9.00E-05	0.73285	0.83417	(Left-Amygdala)(Left-Pallidum)(lh.precuneus_11)(lh.supramarginal_1)
0.009	9.00E-05	0.73285	0.83417	(lh.inferiorparietal_4)(lh.precuneus_11)(lh.superiortemporal_6)(lh.superiortemporal_8)
0.009	9.00E-05	0.73285	0.83417	(Left-Pallidum)(lh.fusiform_6)(lh.inferiorparietal_4)(lh.precuneus_11)
0.009	9.00E-05	0.73285	0.83417	(lh.bankssts_3)(lh.inferiorparietal_4)(lh.inferiorparietal_5)(lh.parahippocampal_1)
0.00906	9.00E-05	0.76895	0.86432	(Brain_Stem)(lh.inferiorparietal_4)(lh.precuneus_11)(lh.superiortemporal_3)
0.00906	9.00E-05	0.76895	0.86432	(Left-Putamen)(lh.inferiorparietal_4)(lh.lingual_6)(lh.lingual_7)
0.00906	9.00E-05	0.76895	0.86432	(Left-Thalamus-Proper)(lh.inferiorparietal_4)(lh.inferiorparietal_5)(lh.insula_4)
0.00906	9.00E-05	0.76895	0.86432	(Left-Putamen)(lh.inferiorparietal_4)(lh.inferiorparietal_5)(lh.insula_4)
0.00906	9.00E-05	0.76895	0.86432	(lh.fusiform_5)(lh.inferiorparietal_4)(lh.lingual_7)(lh.precuneus_11)
0.00906	9.00E-05	0.76895	0.86432	(lh.inferiorparietal_4)(lh.inferiorparietal_5)(lh.insula_2)(lh.insula_4)
0.00906	9.00E-05	0.76895	0.86432	(lh.fusiform_5)(lh.inferiorparietal_5)(lh.supramarginal_1)(lh.transversetemporal_2)
0.00906	0.0001	0.76895	0.86432	(lh.insula_2)(lh.lingual_6)(lh.supramarginal_1)(lh.transversetemporal_2)
0.00912	0.0001	0.7509	0.84925	(lh.insula_2)(lh.precuneus_11)(lh.superiortemporal_6)(lh.supramarginal_1)
0.00912	0.0001	0.7509	0.84925	(Left-Thalamus-Proper)(lh.fusiform_6)(lh.inferiorparietal_4)(lh.lingual_8)
0.00912	0.0001	0.7509	0.84925	(lh.inferiorparietal_5)(lh.parahippocampal_3)(lh.supramarginal_1)(lh.transversetemporal_[illegible]
0.00912	0.0001	0.7509	0.84925	(lh.inferiorparietal_4)(lh.insula_4)(lh.lingual_8)(lh.parahippocampal_1)

0.00912	0.0001	0.7509	0.84925	(Left-Pallidum)(lh.superiortemporal_3)(lh.supramarginal_1)(lh.transversetemporal_2)
0.00912	0.0001	0.7509	0.84925	(lh.fusiform_6)(lh.inferiorparietal_4)(lh.precuneus_11)(lh.superiortemporal_2)
0.00912	0.0001	0.7509	0.84925	(lh.fusiform_6)(lh.inferiorparietal_4)(lh.isthmuscingulate_3)(lh.lingual_8)
0.00912	0.0001	0.7509	0.84925	(Brain_Stem)(lh.inferiorparietal_4)(lh.inferiorparietal_5)(lh.parahippocampal_3)
0.00912	0.0001	0.7509	0.84925	(lh.precuneus_11)(lh.superiortemporal_2)(lh.superiortemporal_6)(lh.supramarginal_1)
0.00912	0.00011	0.7509	0.84925	(Left-Amygdala)(lh.inferiorparietal_4)(lh.lingual_8)(lh.precuneus_11)
0.00912	0.00011	0.7509	0.84925	(Left-Pallidum)(lh.lingual_6)(lh.supramarginal_1)(lh.transversetemporal_2)
0.00912	0.00011	0.7509	0.84925	(lh.fusiform_7)(lh.inferiorparietal_4)(lh.lingual_8)(lh.parahippocampal_1)
0.00912	0.00011	0.7509	0.84925	(lh.inferiorparietal_4)(lh.inferiorparietal_5)(lh.lingual_6)(lh.parahippocampal_2)
0.00912	0.00011	0.7509	0.84925	(Left-Putamen)(lh.fusiform_6)(lh.inferiorparietal_4)(lh.lingual_8)
0.00912	0.00011	0.7509	0.84925	(lh.fusiform_5)(lh.fusiform_6)(lh.inferiorparietal_4)(lh.precuneus_11)
0.00912	0.00011	0.7509	0.84925	(lh.precuneus_11)(lh.superiortemporal_6)(lh.supramarginal_1)
0.00912	0.00011	0.7509	0.84925	(Left-Thalamus-Proper)(lh.precuneus_11)(lh.superiortemporal_6)(lh.supramarginal_1)
0.00912	0.00011	0.7509	0.84925	(lh.fusiform_6)(lh.inferiorparietal_4)(lh.lingual_8)
0.00912	0.00012	0.7509	0.84925	(lh.isthmuscingulate_3)(lh.precuneus_11)(lh.superiortemporal_6)(lh.supramarginal_1)
0.00912	0.00012	0.7509	0.84925	(Left-Putamen)(lh.precuneus_11)(lh.superiortemporal_6)(lh.supramarginal_1)
0.00921	0.00012	0.70397	0.80905	(lh.fusiform_6)(lh.lingual_8)(lh.precuneus_11)(lh.supramarginal_1)
0.00921	0.00012	0.70397	0.80905	(Left-Pallidum)(lh.lingual_7)(lh.supramarginal_1)(lh.transversetemporal_2)
0.00921	0.00012	0.70397	0.80905	(lh.lateraloccipital_9)(lh.precuneus_11)(lh.superiortemporal_3)(lh.supramarginal_1)
0.00921	0.00012	0.70397	0.80905	(lh.inferiorparietal_5)(lh.lateraloccipital_9)(lh.superiortemporal_3)(lh.supramarginal_1)
0.00924	0.00013	0.90253	0.96482	(Left-Thalamus-Proper)(lh.insula_2)(lh.precuneus_11)(lh.transversetemporal_2)
0.00924	0.00013	0.90253	0.96482	(lh.insula_2)(lh.precuneus_11)(lh.transversetemporal_2)
0.00924	0.00013	0.90253	0.96482	(Left-Thalamus-Proper)(lh.inferiorparietal_5)(lh.insula_2)(lh.transversetemporal_2)
0.00924	0.00013	0.90253	0.96482	(Left-Pallidum)(lh.insula_2)(lh.lingual_6)(lh.precuneus_11)
0.00924	0.00013	0.90253	0.96482	(lh.inferiorparietal_5)(lh.insula_2)(lh.isthmuscingulate_3)(lh.transversetemporal_2)
0.00924	0.00013	0.90253	0.96482	(lh.inferiorparietal_5)(lh.insula_2)(lh.transversetemporal_2)
0.00924	0.00014	0.90253	0.96482	(lh.inferiorparietal_5)(lh.insula_2)(lh.lingual_8)(lh.precuneus_11)
0.00924	0.00014	0.90253	0.96482	(Left-Pallidum)(lh.inferiorparietal_5)(lh.precuneus_11)(lh.superiortemporal_2)
0.00924	0.00014	0.90253	0.96482	(Left-Putamen)(lh.inferiorparietal_5)(lh.insula_2)(lh.transversetemporal_2)
0.00924	0.00014	0.90253	0.96482	(Left-Putamen)(lh.insula_2)(lh.precuneus_11)(lh.transversetemporal_2)
0.00924	0.00014	0.90253	0.96482	(lh.insula_2)(lh.isthmuscingulate_3)(lh.precuneus_11)(lh.transversetemporal_2)
0.00935	0.00014	0.81949	0.90452	(lh.fusiform_5)(lh.inferiorparietal_4)(lh.precuneus_11)(lh.superiortemporal_3)
0.00935	0.00015	0.81949	0.90452	(lh.bankssts_3)(lh.inferiorparietal_4)(lh.precuneus_11)(lh.superiortemporal_2)
0.00935	0.00015	0.81949	0.90452	(lh.fusiform_7)(lh.parahippocampal_1)(lh.precuneus_11)(lh.superiortemporal_8)
0.00935	0.00015	0.81949	0.90452	(Left-Putamen)(lh.inferiorparietal_4)(lh.lingual_6)(lh.superiortemporal_3)
0.00936	0.00015	0.78169	0.87437	(lh.bankssts_3)(lh.fusiform_5)(lh.inferiorparietal_4)(lh.parahippocampal_3)

0.00936	0.00016	0.78169	0.87437	(lh.bankssts_3)(lh.fusiform_6)(lh.isthmuscingulate_3)(lh.transversetemporal_2)
0.00936	0.00016	0.78169	0.87437	(Left-Pallidum)(lh.inferiorparietal_4)(lh.lingual_6)(lh.parahippocampal_3)
0.00936	0.00016	0.78169	0.87437	(lh.bankssts_3)(lh.fusiform_6)(lh.transversetemporal_2)
0.00936	0.00016	0.78169	0.87437	(lh.inferiorparietal_5)(lh.lateraloccipital_9)(lh.lingual_6)(lh.transversetemporal_2)
0.00936	0.00017	0.78169	0.87437	(lh.bankssts_3)(lh.fusiform_5)(lh.inferiorparietal_4)(lh.transversetemporal_2)
0.00936	0.00017	0.78169	0.87437	(Left-Pallidum)(lh.inferiorparietal_4)(lh.lingual_8)(lh.transversetemporal_2)
0.00944	0.00017	0.79422	0.88442	(lh.inferiorparietal_4)(lh.insula_1)(lh.lingual_6)(lh.precuneus_11)
0.00944	0.00018	0.79422	0.88442	(lh.inferiorparietal_5)(lh.insula_2)(lh.lingual_6)(lh.supramarginal_1)
0.00944	0.00018	0.79422	0.88442	(lh.fusiform_7)(lh.inferiorparietal_4)(lh.insula_2)(lh.precuneus_11)
0.00954	0.00018	0.89531	0.9598	(Left-Pallidum)(lh.fusiform_5)(lh.inferiorparietal_5)(lh.precuneus_11)
0.00959	0.00019	0.72202	0.82412	(lh.inferiorparietal_4)(lh.parahippocampal_2)(lh.precuneus_11)(lh.superiortemporal_8)
0.00959	0.00019	0.72202	0.82412	(lh.fusiform_6)(lh.inferiorparietal_4)(lh.lingual_8)(lh.transversetemporal_2)
0.00959	0.00019	0.72202	0.82412	(lh.fusiform_7)(lh.inferiorparietal_4)(lh.inferiorparietal_5)(lh.lingual_7)
0.00959	0.0002	0.72202	0.82412	(lh.insula_2)(lh.lingual_7)(lh.supramarginal_1)(lh.transversetemporal_2)
0.00963	0.0002	0.87365	0.94472	(lh.inferiorparietal_5)(lh.lingual_6)(lh.superiortemporal_3)(lh.transversetemporal_2)
0.00963	0.0002	0.87365	0.94472	(lh.lingual_6)(lh.precuneus_11)(lh.superiortemporal_3)(lh.transversetemporal_2)
0.00969	0.00021	0.88809	0.95477	(lh.inferiorparietal_5)(lh.lingual_6)(lh.parahippocampal_3)(lh.precuneus_11)
0.00969	0.00021	0.88809	0.95477	(lh.bankssts_3)(lh.inferiorparietal_5)(lh.precuneus_11)(lh.superiortemporal_3)
0.00971	0.00022	0.88087	0.94975	(Left-Putamen)(lh.bankssts_3)(lh.precuneus_11)(lh.transversetemporal_2)
0.00971	0.00022	0.88087	0.94975	(Left-Putamen)(lh.bankssts_3)(lh.inferiorparietal_5)(lh.transversetemporal_2)
0.00971	0.00023	0.88087	0.94975	(Left-Pallidum)(lh.insula_2)(lh.precuneus_11)(lh.transversetemporal_2)
0.00971	0.00023	0.88087	0.94975	(Left-Pallidum)(lh.inferiorparietal_5)(lh.insula_2)(lh.transversetemporal_2)
0.0098	0.00024	0.77617	0.86935	(Left-Putamen)(lh.inferiorparietal_4)(lh.insula_4)(lh.precuneus_11)
0.0098	0.00024	0.77617	0.86935	(Brain_Stem)(lh.inferiorparietal_4)(lh.inferiorparietal_5)(lh.superiortemporal_2)
0.0098	0.00025	0.77617	0.86935	(lh.insula_2)(lh.parahippocampal_3)(lh.precuneus_11)(lh.supramarginal_1)
0.0098	0.00026	0.77617	0.86935	(Left-Thalamus-Proper)(lh.inferiorparietal_4)(lh.insula_4)(lh.precuneus_11)
0.0098	0.00026	0.77617	0.86935	(Left-Thalamus-Proper)(lh.inferiorparietal_4)(lh.inferiorparietal_5)(lh.superiortemporal_6)
0.0098	0.00027	0.77617	0.86935	(lh.inferiorparietal_4)(lh.insula_4)(lh.precuneus_11)
0.0098	0.00028	0.77617	0.86935	(lh.inferiorparietal_4)(lh.inferiorparietal_5)(lh.superiortemporal_6)
0.0098	0.00029	0.77617	0.86935	(lh.inferiorparietal_4)(lh.inferiorparietal_5)(lh.isthmuscingulate_3)(lh.superiortemporal_6)
0.0098	0.00029	0.77617	0.86935	(lh.inferiorparietal_4)(lh.insula_4)(lh.isthmuscingulate_3)(lh.precuneus_11)
0.0098	0.0003	0.77617	0.86935	(Left-Thalamus-Proper)(lh.lingual_6)(lh.supramarginal_1)(lh.transversetemporal_2)
0.0098	0.00031	0.77617	0.86935	(lh.inferiorparietal_4)(lh.insula_2)(lh.insula_4)(lh.precuneus_11)
0.0098	0.00032	0.77617	0.86935	(Left-Caudate)(lh.inferiorparietal_4)(lh.insula_2)(lh.precuneus_11)
0.0098	0.00033	0.77617	0.86935	(lh.lingual_6)(lh.superiortemporal_2)(lh.supramarginal_1)(lh.transversetemporal_2)
0.00984	0.00034	0.74007	0.8392	(lh.inferiorparietal_4)(lh.inferiorparietal_5)(lh.insula_4)(lh.transversetemporal_2)

0.00984	0.00036	0.74007	0.8392	(lh.fusiform_6)(lh.inferiorparietal_4)(lh.precuneus_11)(lh.superiortemporal_3)
0.00984	0.00037	0.74007	0.8392	(Left-Pallidum)(lh.bankssts_3)(lh.supramarginal_1)(lh.transversetemporal_2)
0.00984	0.00038	0.74007	0.8392	(lh.fusiform_5)(lh.lingual_7)(lh.precuneus_11)(lh.supramarginal_1)
0.00984	0.0004	0.74007	0.8392	(lh.inferiorparietal_4)(lh.parahippocampal_1)(lh.superiortemporal_8)
0.00984	0.00042	0.74007	0.8392	(Left-Thalamus-Proper)(lh.inferiorparietal_4)(lh.parahippocampal_1)(lh.superiortemporal_
0.00984	0.00043	0.74007	0.8392	(lh.entorhinal_1)(lh.inferiorparietal_4)(lh.parahippocampal_1)(lh.precuneus_11)
0.00984	0.00045	0.74007	0.8392	(lh.inferiorparietal_4)(lh.parahippocampal_1)(lh.parahippocampal_3)(lh.precuneus_11)
0.00984	0.00048	0.74007	0.8392	(lh.bankssts_3)(lh.inferiorparietal_4)(lh.lingual_8)(lh.superiortemporal_8)
0.00984	0.0005	0.74007	0.8392	(lh.inferiorparietal_4)(lh.isthmuscingulate_3)(lh.parahippocampal_1)(lh.superiortemporal_
0.00984	0.00053	0.74007	0.8392	(lh.inferiorparietal_4)(lh.insula_2)(lh.parahippocampal_1)(lh.superiortemporal_8)
0.00984	0.00056	0.74007	0.8392	(Left-Putamen)(lh.inferiorparietal_4)(lh.parahippocampal_1)(lh.superiortemporal_8)
0.0099	0.00059	0.82671	0.90955	(Left-Pallidum)(Left-Thalamus-Proper)(lh.inferiorparietal_4)(lh.insula_2)
0.0099	0.00063	0.82671	0.90955	(Left-Pallidum)(lh.inferiorparietal_4)(lh.insula_2)
0.0099	0.00067	0.82671	0.90955	(lh.inferiorparietal_4)(lh.insula_2)(lh.lingual_6)(lh.superiortemporal_2)
0.0099	0.00071	0.82671	0.90955	(Left-Pallidum)(lh.inferiorparietal_4)(lh.insula_2)(lh.isthmuscingulate_3)
0.0099	0.00077	0.82671	0.90955	(Left-Pallidum)(Left-Putamen)(lh.inferiorparietal_4)(lh.insula_2)
0.00992	0.00083	0.75812	0.85427	(Brain_Stem)(lh.inferiorparietal_4)(lh.precuneus_11)(lh.transversetemporal_2)
0.00992	0.00091	0.75812	0.85427	(Left-Pallidum)(lh.inferiorparietal_4)(lh.insula_2)(lh.lingual_7)
0.00992	0.001	0.75812	0.85427	(lh.bankssts_3)(lh.inferiorparietal_4)(lh.lingual_7)(lh.precuneus_11)
0.00992	0.00111	0.75812	0.85427	(lh.inferiorparietal_4)(lh.parahippocampal_1)(lh.precuneus_11)(lh.superiortemporal_2)
0.00992	0.00125	0.75812	0.85427	(Brain_Stem)(lh.bankssts_3)(lh.inferiorparietal_4)(lh.inferiorparietal_5)
0.00992	0.00143	0.75812	0.85427	(Brain_Stem)(Left-Pallidum)(lh.inferiorparietal_4)(lh.lingual_6)
0.00992	0.00167	0.75812	0.85427	(lh.bankssts_3)(lh.insula_2)(lh.supramarginal_1)(lh.transversetemporal_2)
0.00992	0.002	0.75812	0.85427	(lh.insula_1)(lh.precuneus_11)(lh.supramarginal_1)(lh.transversetemporal_2)
0.00992	0.0025	0.75812	0.85427	(Left-Pallidum)(lh.fusiform_7)(lh.inferiorparietal_4)(lh.parahippocampal_1)
0.00992	0.00333	0.75812	0.85427	(Left-Caudate)(lh.insula_2)(lh.precuneus_11)(lh.supramarginal_1)
0.00996	0.005	0.78873	0.8794	(lh.fusiform_6)(lh.lingual_6)(lh.precuneus_11)(lh.transversetemporal_2)
0.00996	0.01	0.78873	0.8794	(lh.inferiorparietal_5)(lh.insula_2)(lh.lateraloccipital_9)(lh.transversetemporal_2)

2)
2)
2)

2)

2)

2)

)

2)

)

)

)

2)

:)

2)

|_2)

2)

)
2)

_2)
l_2)

l_2)

2)

8)

8)
