# Supplementary material for: Good neighbors, bad neighbors: the frequent network neighborhood mapping of the hippocampus enlightens several structural factors of the human intelligence on a 414-subject cohort
Source: Sci Rep. 2020 Jul 20;10:11967. doi: 10.1038/s41598-020-68914-2 (PMC7371878; doi:10.1038/s41598-020-68914-2)
Supplement: Supplementary file 9 — Supplementary Information 9. [file 41598_2020_68914_MOESM9_ESM.pdf]

| p-value | Holm-Bonferroni | frequency_upper | frequency_lower | name |
|---|---|---|---|---|
| 0.00503 | 3.00E-05 | 0.80505 | 0.69347 | (Right-Accumbens-area)(Right-Pallidum)(rh.bankssts_3) |
| 0.00503 | 3.00E-05 | 0.80505 | 0.69347 | (Right-Accumbens-area)(Right-Thalamus-Proper)(rh.bankssts_3)(rh.isthmuscingulate_2) |
| 0.00503 | 3.00E-05 | 0.80505 | 0.69347 | (Right-Accumbens-area)(Right-Pallidum)(Right-Putamen)(rh.bankssts_3) |
| 0.00503 | 3.00E-05 | 0.80505 | 0.69347 | (Right-Accumbens-area)(Right-Putamen)(rh.bankssts_3)(rh.insula_2) |
| 0.00503 | 3.00E-05 | 0.80505 | 0.69347 | (Right-Accumbens-area)(Right-Thalamus-Proper)(rh.bankssts_3)(rh.lingual_7) |
| 0.00503 | 3.00E-05 | 0.80505 | 0.69347 | (Right-Accumbens-area)(rh.bankssts_3)(rh.insula_2)(rh.lingual_7) |
| 0.00503 | 3.00E-05 | 0.80505 | 0.69347 | (Right-Accumbens-area)(rh.bankssts_3)(rh.isthmuscingulate_2) |
| 0.00503 | 3.00E-05 | 0.80505 | 0.69347 | (Right-Accumbens-area)(Right-Putamen)(Right-Thalamus-Proper)(rh.bankssts_3) |
| 0.00503 | 3.00E-05 | 0.80505 | 0.69347 | (Right-Accumbens-area)(Right-Pallidum)(rh.bankssts_3)(rh.insula_2) |
| 0.00503 | 3.00E-05 | 0.80505 | 0.69347 | (Right-Accumbens-area)(Right-Putamen)(rh.bankssts_3) |
| 0.00503 | 3.00E-05 | 0.80505 | 0.69347 | (Right-Accumbens-area)(Right-Thalamus-Proper)(rh.bankssts_3) |
| 0.00503 | 3.00E-05 | 0.80505 | 0.69347 | (Right-Accumbens-area)(Right-Pallidum)(Right-Thalamus-Proper)(rh.bankssts_3) |
| 0.00503 | 3.00E-05 | 0.80505 | 0.69347 | (Right-Accumbens-area)(Right-Putamen)(rh.bankssts_3)(rh.lingual_7) |
| 0.00503 | 3.00E-05 | 0.80505 | 0.69347 | (Right-Accumbens-area)(Right-Putamen)(rh.bankssts_3)(rh.isthmuscingulate_2) |
| 0.00503 | 3.00E-05 | 0.80505 | 0.69347 | (Right-Accumbens-area)(rh.bankssts_3)(rh.isthmuscingulate_2)(rh.lingual_7) |
| 0.00503 | 3.00E-05 | 0.80505 | 0.69347 | (Right-Accumbens-area)(rh.bankssts_2)(rh.bankssts_3)(rh.insula_2) |
| 0.00503 | 4.00E-05 | 0.80505 | 0.69347 | (Right-Accumbens-area)(Right-Pallidum)(rh.bankssts_3)(rh.lingual_7) |
| 0.00503 | 4.00E-05 | 0.80505 | 0.69347 | (Right-Accumbens-area)(Right-Thalamus-Proper)(rh.bankssts_3)(rh.insula_2) |
| 0.00503 | 4.00E-05 | 0.80505 | 0.69347 | (Right-Accumbens-area)(Right-Putamen)(rh.bankssts_2)(rh.bankssts_3) |
| 0.00503 | 4.00E-05 | 0.80505 | 0.69347 | (Right-Accumbens-area)(Right-Pallidum)(rh.bankssts_3)(rh.isthmuscingulate_2) |
| 0.00503 | 4.00E-05 | 0.80505 | 0.69347 | (Right-Accumbens-area)(Right-Pallidum)(rh.bankssts_2)(rh.bankssts_3) |
| 0.00503 | 4.00E-05 | 0.80505 | 0.69347 | (Right-Accumbens-area)(rh.bankssts_3)(rh.insula_2)(rh.isthmuscingulate_2) |
| 0.00503 | 4.00E-05 | 0.80505 | 0.69347 | (Right-Accumbens-area)(rh.bankssts_2)(rh.bankssts_3)(rh.isthmuscingulate_2) |
| 0.00503 | 4.00E-05 | 0.80505 | 0.69347 | (Right-Accumbens-area)(rh.bankssts_3)(rh.insula_2) |
| 0.00503 | 4.00E-05 | 0.80505 | 0.69347 | (Right-Accumbens-area)(Right-Thalamus-Proper)(rh.bankssts_2)(rh.bankssts_3) |
| 0.0068 | 5.00E-05 | 0.80144 | 0.69347 | (Right-Accumbens-area)(rh.bankssts_3)(rh.insula_5) |
| 0.0068 | 5.00E-05 | 0.80144 | 0.69347 | (Right-Accumbens-area)(Right-Putamen)(rh.bankssts_3)(rh.superiortemporal_9) |
| 0.0068 | 5.00E-05 | 0.80144 | 0.69347 | (Right-Accumbens-area)(rh.bankssts_3)(rh.insula_5)(rh.lingual_7) |
| 0.0068 | 5.00E-05 | 0.80144 | 0.69347 | (Right-Accumbens-area)(rh.bankssts_3)(rh.superiortemporal_9) |
| 0.0068 | 5.00E-05 | 0.80144 | 0.69347 | (Right-Accumbens-area)(rh.bankssts_3)(rh.insula_2)(rh.superiortemporal_9) |
| 0.0068 | 5.00E-05 | 0.80144 | 0.69347 | (Right-Accumbens-area)(Right-Pallidum)(rh.bankssts_3)(rh.superiortemporal_9) |
| 0.0068 | 5.00E-05 | 0.80144 | 0.69347 | (Right-Accumbens-area)(rh.bankssts_3)(rh.insula_2)(rh.insula_5) |
| 0.0068 | 5.00E-05 | 0.80144 | 0.69347 | (Right-Accumbens-area)(rh.bankssts_2)(rh.bankssts_3)(rh.insula_5) |

| | | | | |
|---|---|---|---|---|
| 0.0068 | 5.00E-05 | 0.80144 | 0.69347 | (Right-Accumbens-area)(Right-Putamen)(rh.bankssts_3)(rh.insula_5) |
| 0.0068 | 5.00E-05 | 0.80144 | 0.69347 | (Right-Accumbens-area)(rh.bankssts_3)(rh.lingual_7)(rh.superiortemporal_9) |
| 0.0068 | 5.00E-05 | 0.80144 | 0.69347 | (Right-Accumbens-area)(rh.bankssts_3)(rh.isthmuscingulate_2)(rh.superiortemporal_9) |
| 0.0068 | 5.00E-05 | 0.80144 | 0.69347 | (Right-Accumbens-area)(Right-Thalamus-Proper)(rh.bankssts_3)(rh.insula_5) |
| 0.0068 | 5.00E-05 | 0.80144 | 0.69347 | (Right-Accumbens-area)(Right-Thalamus-Proper)(rh.bankssts_3)(rh.superiortemporal_9) |
| 0.0068 | 5.00E-05 | 0.80144 | 0.69347 | (Right-Accumbens-area)(rh.bankssts_2)(rh.bankssts_3)(rh.superiortemporal_9) |
| 0.0068 | 5.00E-05 | 0.80144 | 0.69347 | (Right-Accumbens-area)(rh.bankssts_3)(rh.insula_5)(rh.isthmuscingulate_2) |
| 0.0068 | 5.00E-05 | 0.80144 | 0.69347 | (Right-Accumbens-area)(Right-Pallidum)(rh.bankssts_3)(rh.insula_5) |
