# Supplementary material for: Good neighbors, bad neighbors: the frequent network neighborhood mapping of the hippocampus enlightens several structural factors of the human intelligence on a 414-subject cohort
Source: Sci Rep. 2020 Jul 20;10:11967. doi: 10.1038/s41598-020-68914-2 (PMC7371878; doi:10.1038/s41598-020-68914-2)
Supplement: Supplementary file 10 — Supplementary Information 10. [file 41598_2020_68914_MOESM10_ESM.pdf]

p-value	Holm-Bonferroni	frequency_upper	frequency_lower	name
0.00044	2.00E-05	0.8556	0.95477	(rh.insula_4)(rh.precuneus_2)(rh.precuneus_3)(rh.superiortemporal_1)
0.00059	2.00E-05	0.86643	0.9598	(rh.insula_2)(rh.insula_4)(rh.precuneus_3)(rh.superiortemporal_1)
0.00059	2.00E-05	0.86643	0.9598	(Right-Putamen)(rh.insula_4)(rh.precuneus_3)(rh.superiortemporal_1)
0.00059	2.00E-05	0.86643	0.9598	(Right-Thalamus-Proper)(rh.insula_4)(rh.precuneus_3)(rh.superiortemporal_1)
0.00059	2.00E-05	0.86643	0.9598	(rh.insula_4)(rh.precuneus_3)(rh.superiortemporal_1)
0.00059	2.00E-05	0.86643	0.9598	(rh.insula_4)(rh.isthmuscingulate_2)(rh.precuneus_3)(rh.superiortemporal_1)
0.00064	2.00E-05	0.85921	0.95477	(rh.insula_4)(rh.precuneus_3)(rh.superiortemporal_1)(rh.superiortemporal_3)
0.00067	2.00E-05	0.85199	0.94975	(rh.inferiorparietal_4)(rh.insula_4)(rh.precuneus_3)(rh.superiortemporal_1)
0.00129	2.00E-05	0.86643	0.95477	(rh.insula_4)(rh.lingual_7)(rh.precuneus_3)(rh.superiortemporal_1)
0.00129	2.00E-05	0.86643	0.95477	(rh.bankssts_2)(rh.insula_4)(rh.precuneus_3)(rh.superiortemporal_1)
0.00129	2.00E-05	0.86643	0.95477	(Right-Pallidum)(rh.insula_4)(rh.precuneus_3)(rh.superiortemporal_1)
0.00138	2.00E-05	0.85199	0.94472	(rh.inferiorparietal_10)(rh.insula_4)(rh.precuneus_3)(rh.superiortemporal_1)
0.00147	2.00E-05	0.8917	0.96985	(Right-Pallidum)(rh.precuneus_2)(rh.precuneus_3)(rh.superiortemporal_1)
0.00162	2.00E-05	0.88448	0.96482	(rh.inferiorparietal_4)(rh.precuneus_2)(rh.precuneus_3)(rh.superiortemporal_1)
0.00162	2.00E-05	0.88448	0.96482	(Right-Pallidum)(rh.inferiorparietal_4)(rh.precuneus_3)(rh.superiortemporal_1)
0.00187	2.00E-05	0.76056	0.87437	(rh.insula_4)(rh.precuneus_3)(rh.superiortemporal_1)(rh.transversetemporal_1)
0.00187	2.00E-05	0.76056	0.87437	(rh.insula_4)(rh.precuneus_3)(rh.supramarginal_9)(rh.transversetemporal_1)
0.00187	2.00E-05	0.76056	0.87437	(rh.insula_4)(rh.parahippocampal_1)(rh.precuneus_3)(rh.superiortemporal_1)
0.00187	2.00E-05	0.77978	0.88945	(Right-Putamen)(rh.insula_4)(rh.superiortemporal_1)(rh.transversetemporal_1)
0.00187	2.00E-05	0.77978	0.88945	(rh.insula_2)(rh.insula_4)(rh.superiortemporal_1)(rh.transversetemporal_1)
0.00187	2.00E-05	0.77978	0.88945	(Right-Thalamus-Proper)(rh.insula_4)(rh.superiortemporal_1)(rh.transversetemporal_1)
0.00187	2.00E-05	0.77978	0.88945	(rh.insula_4)(rh.isthmuscingulate_2)(rh.superiortemporal_1)(rh.transversetemporal_1)
0.00188	2.00E-05	0.90253	0.97487	(Right-Pallidum)(rh.isthmuscingulate_2)(rh.precuneus_3)(rh.superiortemporal_1)
0.00188	2.00E-05	0.90253	0.97487	(Right-Pallidum)(Right-Thalamus-Proper)(rh.precuneus_3)(rh.superiortemporal_1)
0.00188	2.00E-05	0.90253	0.97487	(Right-Putamen)(rh.insula_4)(rh.isthmuscingulate_2)(rh.superiortemporal_1)
0.00188	2.00E-05	0.90253	0.97487	(rh.insula_2)(rh.insula_4)(rh.isthmuscingulate_2)(rh.superiortemporal_1)
0.00188	2.00E-05	0.90253	0.97487	(Right-Pallidum)(Right-Putamen)(rh.precuneus_3)(rh.superiortemporal_1)
0.00188	2.00E-05	0.90253	0.97487	(rh.insula_4)(rh.isthmuscingulate_2)(rh.superiortemporal_1)
0.00188	2.00E-05	0.90253	0.97487	(Right-Thalamus-Proper)(rh.insula_2)(rh.insula_4)(rh.superiortemporal_1)
0.00188	2.00E-05	0.90253	0.97487	(Right-Thalamus-Proper)(rh.insula_4)(rh.superiortemporal_1)
0.00188	2.00E-05	0.90253	0.97487	(Right-Thalamus-Proper)(rh.insula_4)(rh.isthmuscingulate_2)(rh.superiortemporal_1)
0.00188	2.00E-05	0.90253	0.97487	(Right-Putamen)(rh.insula_4)(rh.superiortemporal_1)
0.00188	2.00E-05	0.90253	0.97487	(Right-Pallidum)(rh.precuneus_3)(rh.superiortemporal_1)
0.00188	2.00E-05	0.90253	0.97487	(rh.insula_4)(rh.superiortemporal_1)
0.00188	2.00E-05	0.90253	0.97487	(rh.insula_2)(rh.insula_4)(rh.superiortemporal_1)

0.00188	2.00E-05	0.90253	0.97487	(Right-Putamen)(rh.insula_2)(rh.insula_4)(rh.superiortemporal_1)
0.00188	2.00E-05	0.90253	0.97487	(Right-Putamen)(Right-Thalamus-Proper)(rh.insula_4)(rh.superiortemporal_1)
0.00198	2.00E-05	0.76761	0.8794	(rh.inferiorparietal_4)(rh.insula_4)(rh.supramarginal_9)(rh.transversetemporal_1)
0.00198	2.00E-05	0.76761	0.8794	(rh.inferiorparietal_4)(rh.insula_4)(rh.superiortemporal_1)(rh.transversetemporal_1)
0.00208	2.00E-05	0.77465	0.88442	(rh.insula_4)(rh.precuneus_2)(rh.supramarginal_9)(rh.transversetemporal_1)
0.00208	2.00E-05	0.77465	0.88442	(rh.insula_4)(rh.superiortemporal_1)(rh.superiortemporal_3)(rh.transversetemporal_1)
0.00208	2.00E-05	0.77465	0.88442	(rh.insula_4)(rh.superiortemporal_1)(rh.supramarginal_9)(rh.transversetemporal_1)
0.00208	2.00E-05	0.77465	0.88442	(rh.insula_4)(rh.precuneus_2)(rh.superiortemporal_1)(rh.transversetemporal_1)
0.00208	2.00E-05	0.77465	0.88442	(rh.insula_4)(rh.superiortemporal_3)(rh.supramarginal_9)(rh.transversetemporal_1)
0.00212	2.00E-05	0.89531	0.96985	(rh.insula_2)(rh.insula_4)(rh.superiortemporal_1)(rh.superiortemporal_3)
0.00212	2.00E-05	0.89531	0.96985	(Right-Putamen)(rh.insula_4)(rh.superiortemporal_1)(rh.superiortemporal_3)
0.00212	2.00E-05	0.89531	0.96985	(rh.insula_4)(rh.isthmuscingulate_2)(rh.superiortemporal_1)(rh.superiortemporal_3)
0.00212	2.00E-05	0.89531	0.96985	(Right-Putamen)(rh.inferiorparietal_4)(rh.precuneus_3)(rh.superiortemporal_1)
0.00212	2.00E-05	0.89531	0.96985	(Right-Thalamus-Proper)(rh.inferiorparietal_4)(rh.precuneus_3)(rh.superiortemporal_1)
0.00212	2.00E-05	0.89531	0.96985	(rh.insula_2)(rh.precuneus_2)(rh.precuneus_3)(rh.superiortemporal_1)
0.00212	2.00E-05	0.89531	0.96985	(rh.inferiorparietal_4)(rh.isthmuscingulate_2)(rh.precuneus_3)(rh.superiortemporal_1)
0.00212	2.00E-05	0.89531	0.96985	(rh.inferiorparietal_4)(rh.precuneus_3)(rh.superiortemporal_1)
0.00212	2.00E-05	0.89531	0.96985	(Right-Thalamus-Proper)(rh.insula_4)(rh.superiortemporal_1)(rh.superiortemporal_3)
0.00212	2.00E-05	0.89531	0.96985	(rh.insula_4)(rh.superiortemporal_1)(rh.superiortemporal_3)
0.00218	2.00E-05	0.78169	0.88945	(rh.insula_4)(rh.supramarginal_9)(rh.transversetemporal_1)
0.00218	2.00E-05	0.78169	0.88945	(rh.insula_4)(rh.superiortemporal_1)(rh.transversetemporal_1)
0.00231	3.00E-05	0.88809	0.96482	(Right-Putamen)(rh.inferiorparietal_4)(rh.insula_4)(rh.superiortemporal_1)
0.00231	3.00E-05	0.88809	0.96482	(rh.insula_2)(rh.insula_4)(rh.precuneus_2)(rh.superiortemporal_1)
0.00231	3.00E-05	0.88809	0.96482	(rh.inferiorparietal_4)(rh.insula_4)(rh.superiortemporal_1)
0.00231	3.00E-05	0.88809	0.96482	(Right-Thalamus-Proper)(rh.insula_4)(rh.precuneus_2)(rh.superiortemporal_1)
0.00231	3.00E-05	0.88809	0.96482	(rh.inferiorparietal_4)(rh.insula_2)(rh.precuneus_3)(rh.superiortemporal_1)
0.00231	3.00E-05	0.88809	0.96482	(rh.insula_4)(rh.isthmuscingulate_2)(rh.precuneus_2)(rh.superiortemporal_1)
0.00231	3.00E-05	0.88809	0.96482	(Right-Thalamus-Proper)(rh.inferiorparietal_4)(rh.insula_4)(rh.superiortemporal_1)
0.00231	3.00E-05	0.88809	0.96482	(rh.insula_4)(rh.precuneus_2)(rh.superiortemporal_1)
0.00231	3.00E-05	0.88809	0.96482	(Right-Putamen)(rh.insula_4)(rh.precuneus_2)(rh.superiortemporal_1)
0.00231	3.00E-05	0.88809	0.96482	(rh.inferiorparietal_4)(rh.insula_4)(rh.isthmuscingulate_2)(rh.superiortemporal_1)
0.00231	3.00E-05	0.88809	0.96482	(rh.inferiorparietal_4)(rh.insula_2)(rh.insula_4)(rh.superiortemporal_1)
0.00247	3.00E-05	0.88087	0.9598	(rh.inferiorparietal_4)(rh.insula_4)(rh.superiortemporal_1)(rh.superiortemporal_3)
0.00247	3.00E-05	0.88087	0.9598	(rh.insula_4)(rh.precuneus_2)(rh.superiortemporal_1)(rh.superiortemporal_3)
0.00258	3.00E-05	0.87365	0.95477	(rh.inferiorparietal_4)(rh.insula_4)(rh.precuneus_2)(rh.superiortemporal_1)
0.00269	3.00E-05	0.85921	0.94472	(Right-Caudate)(rh.insula_4)(rh.precuneus_3)(rh.superiortemporal_1)

0.00269	3.00E-05	0.85921	0.94472	(rh.insula_4)(rh.precuneus_3)(rh.superiortemporal_1)(rh.supramarginal_9)
0.00269	3.00E-05	0.85199	0.9397	(rh.insula_4)(rh.parahippocampal_2)(rh.precuneus_3)(rh.superiortemporal_1)
0.00272	3.00E-05	0.90614	0.97487	(rh.precuneus_2)(rh.precuneus_3)(rh.superiortemporal_1)
0.00272	3.00E-05	0.90614	0.97487	(rh.isthmuscingulate_2)(rh.precuneus_2)(rh.precuneus_3)(rh.superiortemporal_1)
0.00272	3.00E-05	0.90614	0.97487	(Right-Putamen)(rh.precuneus_2)(rh.precuneus_3)(rh.superiortemporal_1)
0.00272	3.00E-05	0.90614	0.97487	(rh.insula_2)(rh.isthmuscingulate_2)(rh.precuneus_3)(rh.superiortemporal_1)
0.00272	3.00E-05	0.90614	0.97487	(Right-Thalamus-Proper)(rh.insula_2)(rh.precuneus_3)(rh.superiortemporal_1)
0.00272	3.00E-05	0.90614	0.97487	(Right-Putamen)(rh.insula_2)(rh.precuneus_3)(rh.superiortemporal_1)
0.00272	3.00E-05	0.90614	0.97487	(Right-Thalamus-Proper)(rh.precuneus_2)(rh.precuneus_3)(rh.superiortemporal_1)
0.00272	3.00E-05	0.90614	0.97487	(rh.insula_2)(rh.precuneus_3)(rh.superiortemporal_1)
0.00293	3.00E-05	0.77256	0.8794	(Right-Caudate)(rh.insula_4)(rh.superiortemporal_1)(rh.transversetemporal_1)
0.00304	3.00E-05	0.89892	0.96985	(Right-Pallidum)(rh.insula_2)(rh.precuneus_3)(rh.superiortemporal_1)
0.00314	3.00E-05	0.77978	0.88442	(Right-Pallidum)(rh.insula_4)(rh.superiortemporal_1)(rh.transversetemporal_1)
0.00326	3.00E-05	0.76761	0.87437	(rh.inferiorparietal_10)(rh.insula_4)(rh.superiortemporal_1)(rh.transversetemporal_1)
0.00326	3.00E-05	0.76761	0.87437	(rh.inferiorparietal_10)(rh.insula_4)(rh.supramarginal_9)(rh.transversetemporal_1)
0.00335	3.00E-05	0.787	0.88945	(Right-Thalamus-Proper)(rh.insula_4)(rh.supramarginal_9)(rh.transversetemporal_1)
0.00335	3.00E-05	0.787	0.88945	(rh.insula_4)(rh.isthmuscingulate_2)(rh.supramarginal_9)(rh.transversetemporal_1)
0.00335	3.00E-05	0.787	0.88945	(Right-Putamen)(rh.insula_4)(rh.supramarginal_9)(rh.transversetemporal_1)
0.00335	3.00E-05	0.787	0.88945	(rh.insula_2)(rh.insula_4)(rh.supramarginal_9)(rh.transversetemporal_1)
0.00341	3.00E-05	0.91697	0.9799	(Right-Putamen)(rh.precuneus_3)(rh.superiortemporal_1)
0.00341	3.00E-05	0.91697	0.9799	(Right-Putamen)(Right-Thalamus-Proper)(rh.precuneus_3)(rh.superiortemporal_1)
0.00341	3.00E-05	0.91697	0.9799	(Right-Thalamus-Proper)(rh.isthmuscingulate_2)(rh.precuneus_3)(rh.superiortemporal_1)
0.00341	3.00E-05	0.91697	0.9799	(Right-Thalamus-Proper)(rh.precuneus_3)(rh.superiortemporal_1)
0.00341	3.00E-05	0.91697	0.9799	(rh.precuneus_3)(rh.superiortemporal_1)
0.00341	3.00E-05	0.91697	0.9799	(rh.isthmuscingulate_2)(rh.precuneus_3)(rh.superiortemporal_1)
0.00341	3.00E-05	0.91697	0.9799	(Right-Putamen)(rh.isthmuscingulate_2)(rh.precuneus_3)(rh.superiortemporal_1)
0.00345	3.00E-05	0.77465	0.8794	(rh.insula_4)(rh.precuneus_2)(rh.precuneus_3)(rh.transversetemporal_1)
0.00365	3.00E-05	0.78169	0.88442	(rh.insula_4)(rh.lingual_7)(rh.superiortemporal_1)(rh.transversetemporal_1)
0.00365	3.00E-05	0.78169	0.88442	(rh.bankssts_2)(rh.insula_4)(rh.supramarginal_9)(rh.transversetemporal_1)
0.00365	3.00E-05	0.78169	0.88442	(rh.insula_4)(rh.isthmuscingulate_1)(rh.precuneus_3)(rh.superiortemporal_1)
0.00365	3.00E-05	0.78169	0.88442	(rh.bankssts_2)(rh.insula_4)(rh.superiortemporal_1)(rh.transversetemporal_1)
0.00365	3.00E-05	0.78169	0.88442	(rh.insula_4)(rh.lingual_7)(rh.supramarginal_9)(rh.transversetemporal_1)
0.00384	3.00E-05	0.78873	0.88945	(rh.insula_2)(rh.parahippocampal_1)(rh.precuneus_3)(rh.superiortemporal_1)
0.00434	3.00E-05	0.90253	0.96985	(rh.bankssts_2)(rh.insula_2)(rh.insula_4)(rh.superiortemporal_1)
0.00434	3.00E-05	0.90253	0.96985	(rh.insula_2)(rh.insula_4)(rh.lingual_7)(rh.superiortemporal_1)
0.00434	3.00E-05	0.90253	0.96985	(Right-Pallidum)(Right-Thalamus-Proper)(rh.insula_4)(rh.superiortemporal_1)

0.00434	3.00E-05	0.90253	0.96985	(rh.bankssts_2)(rh.insula_4)(rh.isthmuscingulate_2)(rh.superiortemporal_1)
0.00434	3.00E-05	0.90253	0.96985	(Right-Pallidum)(Right-Putamen)(rh.insula_4)(rh.superiortemporal_1)
0.00434	3.00E-05	0.90253	0.96985	(Right-Putamen)(rh.bankssts_2)(rh.insula_4)(rh.superiortemporal_1)
0.00434	3.00E-05	0.90253	0.96985	(rh.insula_4)(rh.isthmuscingulate_2)(rh.lingual_7)(rh.superiortemporal_1)
0.00434	3.00E-05	0.90253	0.96985	(Right-Thalamus-Proper)(rh.bankssts_2)(rh.insula_4)(rh.superiortemporal_1)
0.00434	3.00E-05	0.90253	0.96985	(Right-Pallidum)(rh.lingual_7)(rh.precuneus_3)(rh.superiortemporal_1)
0.00434	3.00E-05	0.90253	0.96985	(Right-Putamen)(rh.insula_4)(rh.lingual_7)(rh.superiortemporal_1)
0.00434	3.00E-05	0.90253	0.96985	(Right-Pallidum)(rh.insula_4)(rh.isthmuscingulate_2)(rh.superiortemporal_1)
0.00434	3.00E-05	0.90253	0.96985	(rh.insula_4)(rh.lingual_7)(rh.superiortemporal_1)
0.00434	3.00E-05	0.90253	0.96985	(Right-Pallidum)(rh.insula_2)(rh.insula_4)(rh.superiortemporal_1)
0.00434	3.00E-05	0.90253	0.96985	(Right-Thalamus-Proper)(rh.insula_4)(rh.lingual_7)(rh.superiortemporal_1)
0.00434	3.00E-05	0.90253	0.96985	(Right-Pallidum)(rh.bankssts_2)(rh.precuneus_3)(rh.superiortemporal_1)
0.00434	3.00E-05	0.90253	0.96985	(Right-Pallidum)(rh.insula_4)(rh.superiortemporal_1)
0.00434	3.00E-05	0.90253	0.96985	(rh.bankssts_2)(rh.insula_4)(rh.superiortemporal_1)
0.00445	3.00E-05	0.79061	0.88945	(Right-Putamen)(rh.insula_4)(rh.precuneus_2)(rh.transversetemporal_1)
0.00445	3.00E-05	0.79061	0.88945	(Right-Thalamus-Proper)(rh.insula_4)(rh.precuneus_2)(rh.transversetemporal_1)
0.00445	3.00E-05	0.79061	0.88945	(rh.insula_2)(rh.insula_4)(rh.precuneus_2)(rh.transversetemporal_1)
0.00445	3.00E-05	0.79061	0.88945	(rh.insula_4)(rh.isthmuscingulate_2)(rh.precuneus_2)(rh.transversetemporal_1)
0.00452	3.00E-05	0.71119	0.82412	(rh.fusiform_5)(rh.insula_4)(rh.superiortemporal_1)(rh.transversetemporal_1)
0.00466	3.00E-05	0.89531	0.96482	(rh.insula_4)(rh.lingual_7)(rh.superiortemporal_1)(rh.superiortemporal_3)
0.00466	3.00E-05	0.89531	0.96482	(rh.inferiorparietal_4)(rh.lingual_7)(rh.precuneus_3)(rh.superiortemporal_1)
0.00466	3.00E-05	0.89531	0.96482	(rh.bankssts_2)(rh.inferiorparietal_4)(rh.precuneus_3)(rh.superiortemporal_1)
0.00466	3.00E-05	0.89531	0.96482	(rh.bankssts_2)(rh.insula_4)(rh.superiortemporal_1)(rh.superiortemporal_3)
0.00466	3.00E-05	0.89531	0.96482	(Right-Pallidum)(rh.insula_4)(rh.superiortemporal_1)(rh.superiortemporal_3)
0.00478	3.00E-05	0.93863	0.98995	(Right-Pallidum)(rh.superiortemporal_1)
0.00478	3.00E-05	0.93863	0.98995	(Right-Pallidum)(Right-Putamen)(rh.isthmuscingulate_2)(rh.superiortemporal_1)
0.00478	3.00E-05	0.93863	0.98995	(Right-Pallidum)(Right-Thalamus-Proper)(rh.isthmuscingulate_2)(rh.superiortemporal_1)
0.00478	3.00E-05	0.93863	0.98995	(Right-Pallidum)(Right-Thalamus-Proper)(rh.superiortemporal_1)
0.00478	3.00E-05	0.93863	0.98995	(Right-Pallidum)(Right-Putamen)(Right-Thalamus-Proper)(rh.superiortemporal_1)
0.00478	3.00E-05	0.93863	0.98995	(Right-Pallidum)(rh.isthmuscingulate_2)(rh.superiortemporal_1)
0.00478	3.00E-05	0.93863	0.98995	(Right-Pallidum)(Right-Putamen)(rh.superiortemporal_1)
0.00483	3.00E-05	0.70036	0.81407	(rh.lateraloccipital_10)(rh.precuneus_3)(rh.superiortemporal_1)(rh.superiortemporal_6)
0.0049	3.00E-05	0.88809	0.9598	(rh.bankssts_2)(rh.insula_4)(rh.precuneus_2)(rh.superiortemporal_1)
0.0049	3.00E-05	0.88809	0.9598	(Right-Pallidum)(rh.insula_4)(rh.precuneus_2)(rh.superiortemporal_1)
0.0049	3.00E-05	0.88809	0.9598	(rh.inferiorparietal_10)(rh.insula_4)(rh.superiortemporal_1)
0.0049	3.00E-05	0.88809	0.9598	(Right-Thalamus-Proper)(rh.inferiorparietal_10)(rh.insula_4)(rh.superiortemporal_1)

0.0049	3.00E-05	0.88809	0.9598	(rh.inferiorparietal_10)(rh.insula_2)(rh.insula_4)(rh.superiortemporal_1)
0.0049	3.00E-05	0.88809	0.9598	(rh.insula_4)(rh.lingual_7)(rh.precuneus_2)(rh.superiortemporal_1)
0.0049	3.00E-05	0.88809	0.9598	(rh.inferiorparietal_4)(rh.insula_4)(rh.lingual_7)(rh.superiortemporal_1)
0.0049	3.00E-05	0.88809	0.9598	(Right-Putamen)(rh.inferiorparietal_10)(rh.insula_4)(rh.superiortemporal_1)
0.0049	3.00E-05	0.88809	0.9598	(Right-Pallidum)(rh.inferiorparietal_10)(rh.precuneus_3)(rh.superiortemporal_1)
0.0049	3.00E-05	0.88809	0.9598	(rh.inferiorparietal_10)(rh.insula_4)(rh.isthmuscingulate_2)(rh.superiortemporal_1)
0.0049	3.00E-05	0.88809	0.9598	(rh.bankssts_2)(rh.inferiorparietal_4)(rh.insula_4)(rh.superiortemporal_1)
0.0049	3.00E-05	0.88809	0.9598	(Right-Pallidum)(rh.inferiorparietal_4)(rh.insula_4)(rh.superiortemporal_1)
0.00497	3.00E-05	0.92058	0.9799	(Right-Pallidum)(Right-Thalamus-Proper)(rh.inferiorparietal_4)(rh.superiortemporal_1)
0.00497	3.00E-05	0.92058	0.9799	(Right-Pallidum)(Right-Putamen)(rh.inferiorparietal_4)(rh.superiortemporal_1)
0.00497	3.00E-05	0.92058	0.9799	(Right-Pallidum)(rh.inferiorparietal_4)(rh.isthmuscingulate_2)(rh.superiortemporal_1)
0.00497	3.00E-05	0.92058	0.9799	(Right-Pallidum)(rh.inferiorparietal_4)(rh.superiortemporal_1)
0.00505	4.00E-05	0.88087	0.95477	(rh.inferiorparietal_10)(rh.insula_4)(rh.superiortemporal_1)(rh.superiortemporal_3)
0.00505	4.00E-05	0.88087	0.95477	(rh.inferiorparietal_4)(rh.precuneus_3)(rh.superiortemporal_1)(rh.superiortemporal_3)
0.00505	4.00E-05	0.88087	0.95477	(rh.insula_4)(rh.precuneus_2)(rh.superiortemporal_1)(rh.supramarginal_9)
0.00505	4.00E-05	0.88087	0.95477	(rh.inferiorparietal_10)(rh.inferiorparietal_4)(rh.precuneus_3)(rh.superiortemporal_1)
0.00512	4.00E-05	0.87365	0.94975	(rh.inferiorparietal_10)(rh.insula_4)(rh.precuneus_2)(rh.superiortemporal_1)
0.00512	4.00E-05	0.87365	0.94975	(rh.inferiorparietal_10)(rh.inferiorparietal_4)(rh.insula_4)(rh.superiortemporal_1)
0.00512	4.00E-05	0.87365	0.94975	(rh.insula_4)(rh.precuneus_2)(rh.precuneus_3)(rh.supramarginal_9)
0.00512	4.00E-05	0.87365	0.94975	(rh.inferiorparietal_4)(rh.insula_4)(rh.precuneus_2)(rh.precuneus_3)
0.00513	4.00E-05	0.77978	0.8794	(Right-Putamen)(rh.insula_4)(rh.precuneus_3)(rh.transversetemporal_1)
0.00513	4.00E-05	0.77978	0.8794	(Right-Caudate)(rh.insula_4)(rh.supramarginal_9)(rh.transversetemporal_1)
0.00513	4.00E-05	0.77978	0.8794	(rh.insula_2)(rh.insula_4)(rh.precuneus_3)(rh.transversetemporal_1)
0.00513	4.00E-05	0.77978	0.8794	(rh.insula_4)(rh.isthmuscingulate_2)(rh.precuneus_3)(rh.transversetemporal_1)
0.00513	4.00E-05	0.77978	0.8794	(Right-Thalamus-Proper)(rh.insula_4)(rh.precuneus_3)(rh.transversetemporal_1)
0.00524	4.00E-05	0.76761	0.86935	(rh.inferiorparietal_4)(rh.middletemporal_1)(rh.precuneus_3)(rh.superiortemporal_1)
0.00524	4.00E-05	0.76761	0.86935	(rh.inferiorparietal_4)(rh.insula_4)(rh.precuneus_3)(rh.transversetemporal_1)
0.0055	4.00E-05	0.787	0.88442	(Right-Pallidum)(rh.insula_4)(rh.supramarginal_9)(rh.transversetemporal_1)
0.0055	4.00E-05	0.787	0.88442	(rh.inferiorparietal_4)(rh.insula_4)(rh.isthmuscingulate_2)(rh.transversetemporal_1)
0.0055	4.00E-05	0.787	0.88442	(Right-Thalamus-Proper)(rh.inferiorparietal_4)(rh.insula_4)(rh.transversetemporal_1)
0.0055	4.00E-05	0.787	0.88442	(Right-Putamen)(rh.inferiorparietal_4)(rh.insula_4)(rh.transversetemporal_1)
0.0055	4.00E-05	0.787	0.88442	(rh.inferiorparietal_4)(rh.insula_2)(rh.insula_4)(rh.transversetemporal_1)
0.00558	4.00E-05	0.77465	0.87437	(rh.insula_4)(rh.precuneus_3)(rh.superiortemporal_3)(rh.transversetemporal_1)
0.0058	4.00E-05	0.76895	0.86935	(rh.inferiorparietal_4)(rh.lateraloccipital_10)(rh.precuneus_3)(rh.superiortemporal_1)
0.00592	4.00E-05	0.78169	0.8794	(rh.insula_4)(rh.precuneus_3)(rh.transversetemporal_1)
0.00592	4.00E-05	0.78169	0.8794	(rh.inferiorparietal_10)(rh.inferiortemporal_2)(rh.precuneus_3)(rh.superiortemporal_1)

0.00592	4.00E-05	0.78169	0.8794	(rh.inferiorparietal_4)(rh.insula_4)(rh.superiortemporal_3)(rh.transversetemporal_1)
0.00592	4.00E-05	0.78169	0.8794	(rh.inferiorparietal_4)(rh.inferiortemporal_2)(rh.precuneus_3)(rh.superiortemporal_1)
0.00592	4.00E-05	0.78169	0.8794	(rh.inferiorparietal_4)(rh.insula_4)(rh.precuneus_2)(rh.transversetemporal_1)
0.00609	4.00E-05	0.93141	0.98492	(Right-Putamen)(Right-Thalamus-Proper)(rh.inferiorparietal_4)(rh.superiortemporal_1)
0.00609	4.00E-05	0.93141	0.98492	(Right-Putamen)(rh.inferiorparietal_4)(rh.superiortemporal_1)
0.00609	4.00E-05	0.93141	0.98492	(Right-Thalamus-Proper)(rh.inferiorparietal_4)(rh.isthmuscingulate_2)(rh.superiortempora
0.00609	4.00E-05	0.93141	0.98492	(Right-Putamen)(rh.inferiorparietal_4)(rh.isthmuscingulate_2)(rh.superiortemporal_1)
0.00609	4.00E-05	0.93141	0.98492	(rh.inferiorparietal_4)(rh.isthmuscingulate_2)(rh.superiortemporal_1)
0.00609	4.00E-05	0.93141	0.98492	(Right-Thalamus-Proper)(rh.inferiorparietal_4)(rh.superiortemporal_1)
0.00609	4.00E-05	0.93141	0.98492	(rh.inferiorparietal_4)(rh.superiortemporal_1)
0.00617	4.00E-05	0.83394	0.9196	(rh.insula_4)(rh.middletemporal_4)(rh.precuneus_3)(rh.superiortemporal_1)
0.00618	4.00E-05	0.90614	0.96985	(rh.insula_2)(rh.lingual_7)(rh.precuneus_3)(rh.superiortemporal_1)
0.00618	4.00E-05	0.90614	0.96985	(rh.bankssts_2)(rh.insula_2)(rh.precuneus_3)(rh.superiortemporal_1)
0.00618	4.00E-05	0.90614	0.96985	(Right-Pallidum)(rh.inferiorparietal_4)(rh.precuneus_2)(rh.superiortemporal_1)
0.00618	4.00E-05	0.90614	0.96985	(rh.bankssts_2)(rh.precuneus_2)(rh.precuneus_3)(rh.superiortemporal_1)
0.00618	4.00E-05	0.90614	0.96985	(rh.lingual_7)(rh.precuneus_2)(rh.precuneus_3)(rh.superiortemporal_1)
0.00625	4.00E-05	0.77617	0.87437	(Right-Pallidum)(rh.lateraloccipital_10)(rh.precuneus_3)(rh.superiortemporal_1)
0.00627	4.00E-05	0.78873	0.88442	(rh.inferiortemporal_2)(rh.precuneus_2)(rh.precuneus_3)(rh.superiortemporal_1)
0.00627	4.00E-05	0.78873	0.88442	(rh.inferiorparietal_4)(rh.insula_4)(rh.transversetemporal_1)
0.00627	4.00E-05	0.78873	0.88442	(rh.insula_2)(rh.precuneus_3)(rh.superiortemporal_1)(rh.transversetemporal_1)
0.00627	4.00E-05	0.78873	0.88442	(rh.insula_4)(rh.precuneus_2)(rh.superiortemporal_3)(rh.transversetemporal_1)
0.00627	4.00E-05	0.80144	0.89447	(Right-Thalamus-Proper)(rh.insula_4)(rh.isthmuscingulate_2)(rh.transversetemporal_1)
0.00627	4.00E-05	0.80144	0.89447	(rh.fusiform_5)(rh.inferiorparietal_4)(rh.insula_4)(rh.superiortemporal_1)
0.00627	4.00E-05	0.80144	0.89447	(rh.insula_4)(rh.isthmuscingulate_2)(rh.transversetemporal_1)
0.00627	4.00E-05	0.80144	0.89447	(rh.insula_2)(rh.insula_4)(rh.isthmuscingulate_2)(rh.transversetemporal_1)
0.00627	4.00E-05	0.80144	0.89447	(Right-Putamen)(rh.insula_2)(rh.insula_4)(rh.transversetemporal_1)
0.00627	4.00E-05	0.80144	0.89447	(Right-Putamen)(rh.insula_4)(rh.isthmuscingulate_2)(rh.transversetemporal_1)
0.00627	4.00E-05	0.80144	0.89447	(rh.insula_2)(rh.insula_4)(rh.transversetemporal_1)
0.00627	4.00E-05	0.80144	0.89447	(Right-Thalamus-Proper)(rh.insula_2)(rh.insula_4)(rh.transversetemporal_1)
0.00627	4.00E-05	0.80144	0.89447	(Right-Thalamus-Proper)(rh.insula_4)(rh.transversetemporal_1)
0.00627	5.00E-05	0.80144	0.89447	(Right-Putamen)(Right-Thalamus-Proper)(rh.insula_4)(rh.transversetemporal_1)
0.00627	5.00E-05	0.80144	0.89447	(Right-Putamen)(rh.insula_4)(rh.transversetemporal_1)
0.00627	5.00E-05	0.80144	0.89447	(rh.insula_4)(rh.transversetemporal_1)
0.00645	5.00E-05	0.75812	0.8593	(rh.inferiorparietal_4)(rh.insula_4)(rh.lateraloccipital_10)(rh.precuneus_3)
0.00658	5.00E-05	0.89892	0.96482	(rh.insula_2)(rh.precuneus_3)(rh.superiortemporal_1)(rh.superiortemporal_3)
0.00658	5.00E-05	0.89892	0.96482	(rh.precuneus_2)(rh.precuneus_3)(rh.superiortemporal_1)(rh.supramarginal_9)

0.00662	5.00E-05	0.79577	0.88945	(rh.insula_4)(rh.superiortemporal_3)(rh.transversetemporal_1)
0.00662	5.00E-05	0.79577	0.88945	(Right-Pallidum)(rh.parahippocampal_1)(rh.precuneus_3)(rh.superiortemporal_1)
0.00662	5.00E-05	0.79577	0.88945	(rh.inferiorparietal_4)(rh.insula_2)(rh.superiortemporal_1)(rh.transversetemporal_1)
0.00662	5.00E-05	0.79577	0.88945	(rh.fusiform_5)(rh.insula_4)(rh.precuneus_3)(rh.superiortemporal_1)
0.00662	5.00E-05	0.79577	0.88945	(rh.inferiortemporal_2)(rh.precuneus_3)(rh.superiortemporal_1)
0.00662	5.00E-05	0.79577	0.88945	(rh.insula_4)(rh.precuneus_2)(rh.transversetemporal_1)
0.00667	5.00E-05	0.80866	0.8995	(rh.fusiform_5)(rh.inferiorparietal_4)(rh.precuneus_3)(rh.superiortemporal_1)
0.00673	5.00E-05	0.78339	0.8794	(Right-Caudate)(rh.insula_4)(rh.precuneus_2)(rh.transversetemporal_1)
0.00682	5.00E-05	0.8556	0.93467	(rh.insula_4)(rh.insula_5)(rh.precuneus_3)(rh.superiortemporal_1)
0.00684	5.00E-05	0.71119	0.8191	(rh.fusiform_5)(rh.insula_4)(rh.precuneus_3)(rh.transversetemporal_1)
0.00685	5.00E-05	0.8917	0.9598	(rh.inferiorparietal_10)(rh.insula_2)(rh.precuneus_3)(rh.superiortemporal_1)
0.00685	5.00E-05	0.8917	0.9598	(rh.precuneus_2)(rh.precuneus_3)(rh.superiortemporal_1)(rh.superiortemporal_3)
0.00685	5.00E-05	0.8917	0.9598	(rh.insula_4)(rh.isthmuscingulate_2)(rh.precuneus_2)(rh.precuneus_3)
0.00685	5.00E-05	0.8917	0.9598	(rh.inferiorparietal_10)(rh.precuneus_2)(rh.precuneus_3)(rh.superiortemporal_1)
0.00685	5.00E-05	0.8917	0.9598	(rh.insula_2)(rh.insula_4)(rh.precuneus_2)(rh.precuneus_3)
0.00685	5.00E-05	0.8917	0.9598	(Right-Putamen)(rh.insula_4)(rh.precuneus_2)(rh.precuneus_3)
0.00685	5.00E-05	0.8917	0.9598	(Right-Thalamus-Proper)(rh.insula_4)(rh.precuneus_2)(rh.precuneus_3)
0.00685	5.00E-05	0.8917	0.9598	(Right-Pallidum)(rh.precuneus_3)(rh.superiortemporal_1)(rh.superiortemporal_3)
0.00685	5.00E-05	0.8917	0.9598	(rh.insula_4)(rh.precuneus_2)(rh.precuneus_3)
0.00699	6.00E-05	0.76534	0.86432	(rh.inferiorparietal_4)(rh.insula_4)(rh.lateraloccipital_10)(rh.superiortemporal_1)
0.00699	6.00E-05	0.76534	0.86432	(rh.insula_4)(rh.lateraloccipital_10)(rh.precuneus_2)(rh.precuneus_3)
0.00699	6.00E-05	0.76534	0.86432	(rh.insula_4)(rh.lateraloccipital_10)(rh.precuneus_2)(rh.superiortemporal_1)
0.00701	6.00E-05	0.88448	0.95477	(Right-Putamen)(rh.inferiorparietal_4)(rh.insula_4)(rh.precuneus_3)
0.00701	6.00E-05	0.88448	0.95477	(rh.inferiorparietal_4)(rh.insula_2)(rh.insula_4)(rh.precuneus_3)
0.00701	6.00E-05	0.88448	0.95477	(rh.insula_4)(rh.precuneus_2)(rh.precuneus_3)(rh.superiortemporal_3)
0.00701	6.00E-05	0.88448	0.95477	(rh.inferiorparietal_4)(rh.insula_4)(rh.isthmuscingulate_2)(rh.precuneus_3)
0.00701	6.00E-05	0.88448	0.95477	(Right-Thalamus-Proper)(rh.inferiorparietal_4)(rh.insula_4)(rh.precuneus_3)
0.00701	6.00E-05	0.88448	0.95477	(rh.inferiorparietal_4)(rh.insula_4)(rh.precuneus_3)
0.00702	6.00E-05	0.72924	0.83417	(rh.insula_4)(rh.precuneus_4)(rh.superiortemporal_1)(rh.transversetemporal_1)
0.00705	6.00E-05	0.87004	0.94472	(rh.inferiorparietal_10)(rh.insula_4)(rh.precuneus_2)(rh.precuneus_3)
0.00707	6.00E-05	0.87726	0.94975	(rh.inferiorparietal_4)(rh.insula_4)(rh.precuneus_3)(rh.superiortemporal_3)
0.00708	6.00E-05	0.74729	0.84925	(rh.insula_4)(rh.middletemporal_4)(rh.superiortemporal_1)(rh.transversetemporal_1)
0.00711	6.00E-05	0.94224	0.98995	(rh.insula_2)(rh.superiortemporal_1)
0.00711	6.00E-05	0.94224	0.98995	(Right-Thalamus-Proper)(rh.insula_2)(rh.superiortemporal_1)
0.00711	6.00E-05	0.94224	0.98995	(Right-Putamen)(rh.insula_2)(rh.superiortemporal_1)
0.00711	6.00E-05	0.94224	0.98995	(Right-Putamen)(rh.insula_2)(rh.isthmuscingulate_2)(rh.superiortemporal_1)

0.00711	6.00E-05	0.94224	0.98995	(Right-Putamen)(Right-Thalamus-Proper)(rh.insula_2)(rh.superiortemporal_1)
0.00711	6.00E-05	0.94224	0.98995	(Right-Thalamus-Proper)(rh.insula_2)(rh.isthmuscingulate_2)(rh.superiortemporal_1)
0.00711	6.00E-05	0.94224	0.98995	(rh.insula_2)(rh.isthmuscingulate_2)(rh.superiortemporal_1)
0.0072	6.00E-05	0.92419	0.9799	(Right-Putamen)(rh.inferiorparietal_4)(rh.insula_2)(rh.superiortemporal_1)
0.0072	6.00E-05	0.92419	0.9799	(Right-Thalamus-Proper)(rh.inferiorparietal_4)(rh.insula_2)(rh.superiortemporal_1)
0.0072	6.00E-05	0.92419	0.9799	(Right-Pallidum)(Right-Thalamus-Proper)(rh.precuneus_2)(rh.superiortemporal_1)
0.0072	6.00E-05	0.92419	0.9799	(Right-Pallidum)(rh.precuneus_2)(rh.superiortemporal_1)
0.0072	6.00E-05	0.92419	0.9799	(Right-Pallidum)(Right-Putamen)(rh.precuneus_2)(rh.superiortemporal_1)
0.0072	6.00E-05	0.92419	0.9799	(Right-Pallidum)(rh.isthmuscingulate_2)(rh.precuneus_2)(rh.superiortemporal_1)
0.0072	6.00E-05	0.92419	0.9799	(rh.inferiorparietal_4)(rh.insula_2)(rh.isthmuscingulate_2)(rh.superiortemporal_1)
0.0072	6.00E-05	0.92419	0.9799	(rh.inferiorparietal_4)(rh.insula_2)(rh.superiortemporal_1)
0.00722	7.00E-05	0.79061	0.88442	(Right-Pallidum)(rh.insula_4)(rh.precuneus_2)(rh.transversetemporal_1)
0.00751	7.00E-05	0.71841	0.82412	(rh.fusiform_5)(rh.inferiorparietal_4)(rh.insula_4)(rh.transversetemporal_1)
0.00751	7.00E-05	0.71841	0.82412	(rh.fusiform_5)(rh.insula_4)(rh.supramarginal_9)(rh.transversetemporal_1)
0.00756	7.00E-05	0.77256	0.86935	(Right-Caudate)(rh.insula_4)(rh.precuneus_3)(rh.transversetemporal_1)
0.00756	7.00E-05	0.77256	0.86935	(rh.insula_4)(rh.lateraloccipital_10)(rh.superiortemporal_1)(rh.superiortemporal_3)
0.00761	7.00E-05	0.95307	0.99497	(Right-Thalamus-Proper)(rh.superiortemporal_1)
0.00761	7.00E-05	0.95307	0.99497	(Right-Putamen)(Right-Thalamus-Proper)(rh.superiortemporal_1)
0.00761	7.00E-05	0.95307	0.99497	(Right-Putamen)(rh.superiortemporal_1)
0.00761	7.00E-05	0.95307	0.99497	(rh.superiortemporal_1)
0.00761	7.00E-05	0.95307	0.99497	(Right-Putamen)(rh.isthmuscingulate_2)(rh.superiortemporal_1)
0.00761	7.00E-05	0.95307	0.99497	(Right-Putamen)(Right-Thalamus-Proper)(rh.isthmuscingulate_2)(rh.superiortemporal_1)
0.00761	7.00E-05	0.95307	0.99497	(Right-Thalamus-Proper)(rh.isthmuscingulate_2)(rh.superiortemporal_1)
0.00761	7.00E-05	0.95307	0.99497	(rh.isthmuscingulate_2)(rh.superiortemporal_1)
0.00774	7.00E-05	0.79783	0.88945	(rh.insula_2)(rh.insula_4)(rh.superiortemporal_3)(rh.transversetemporal_1)
0.00774	7.00E-05	0.79783	0.88945	(Right-Thalamus-Proper)(rh.insula_4)(rh.superiortemporal_3)(rh.transversetemporal_1)
0.00774	7.00E-05	0.79783	0.88945	(Right-Pallidum)(rh.inferiorparietal_4)(rh.superiortemporal_1)(rh.transversetemporal_1)
0.00774	7.00E-05	0.79783	0.88945	(rh.insula_4)(rh.isthmuscingulate_2)(rh.superiortemporal_3)(rh.transversetemporal_1)
0.00774	7.00E-05	0.79783	0.88945	(rh.inferiorparietal_4)(rh.precuneus_2)(rh.superiortemporal_1)(rh.transversetemporal_1)
0.00774	7.00E-05	0.79783	0.88945	(Right-Putamen)(rh.insula_4)(rh.superiortemporal_3)(rh.transversetemporal_1)
0.00809	7.00E-05	0.91697	0.97487	(rh.bankssts_2)(rh.isthmuscingulate_2)(rh.precuneus_3)(rh.superiortemporal_1)
0.00809	8.00E-05	0.91697	0.97487	(rh.lingual_7)(rh.precuneus_3)(rh.superiortemporal_1)
0.00809	8.00E-05	0.91697	0.97487	(Right-Thalamus-Proper)(rh.bankssts_2)(rh.precuneus_3)(rh.superiortemporal_1)
0.00809	8.00E-05	0.91697	0.97487	(rh.inferiorparietal_4)(rh.precuneus_2)(rh.superiortemporal_1)
0.00809	8.00E-05	0.91697	0.97487	(Right-Putamen)(rh.inferiorparietal_4)(rh.precuneus_2)(rh.superiortemporal_1)
0.00809	8.00E-05	0.91697	0.97487	(rh.isthmuscingulate_2)(rh.lingual_7)(rh.precuneus_3)(rh.superiortemporal_1)

0.00809	8.00E-05	0.91697	0.97487	(Right-Putamen)(rh.lingual_7)(rh.precuneus_3)(rh.superiortemporal_1)
0.00809	8.00E-05	0.91697	0.97487	(rh.bankssts_2)(rh.precuneus_3)(rh.superiortemporal_1)
0.00809	8.00E-05	0.91697	0.97487	(Right-Thalamus-Proper)(rh.lingual_7)(rh.precuneus_3)(rh.superiortemporal_1)
0.00809	8.00E-05	0.91697	0.97487	(Right-Pallidum)(rh.inferiorparietal_4)(rh.insula_2)(rh.superiortemporal_1)
0.00809	8.00E-05	0.91697	0.97487	(Right-Putamen)(rh.bankssts_2)(rh.precuneus_3)(rh.superiortemporal_1)
0.00809	8.00E-05	0.91697	0.97487	(rh.inferiorparietal_4)(rh.isthmuscingulate_2)(rh.precuneus_2)(rh.superiortemporal_1)
0.00809	8.00E-05	0.91697	0.97487	(Right-Thalamus-Proper)(rh.inferiorparietal_4)(rh.precuneus_2)(rh.superiortemporal_1)
0.00815	8.00E-05	0.77978	0.87437	(rh.insula_4)(rh.isthmuscingulate_2)(rh.lateraloccipital_10)(rh.superiortemporal_1)
0.00815	8.00E-05	0.77978	0.87437	(Right-Thalamus-Proper)(rh.insula_4)(rh.lateraloccipital_10)(rh.superiortemporal_1)
0.00815	8.00E-05	0.77978	0.87437	(rh.insula_4)(rh.lateraloccipital_10)(rh.superiortemporal_1)
0.00815	8.00E-05	0.77978	0.87437	(Right-Pallidum)(rh.insula_4)(rh.precuneus_3)(rh.transversetemporal_1)
0.00815	9.00E-05	0.77978	0.87437	(rh.insula_2)(rh.insula_4)(rh.lateraloccipital_10)(rh.superiortemporal_1)
0.00815	9.00E-05	0.77978	0.87437	(rh.insula_2)(rh.lateraloccipital_10)(rh.precuneus_3)(rh.superiortemporal_1)
0.00815	9.00E-05	0.77978	0.87437	(rh.lateraloccipital_10)(rh.precuneus_2)(rh.precuneus_3)(rh.superiortemporal_1)
0.00815	9.00E-05	0.77978	0.87437	(Right-Caudate)(rh.inferiorparietal_4)(rh.insula_4)(rh.transversetemporal_1)
0.00815	9.00E-05	0.77978	0.87437	(Right-Putamen)(rh.insula_4)(rh.lateraloccipital_10)(rh.superiortemporal_1)
0.00823	9.00E-05	0.76761	0.86432	(rh.inferiorparietal_10)(rh.insula_4)(rh.precuneus_3)(rh.transversetemporal_1)
0.00823	9.00E-05	0.72563	0.82915	(rh.fusiform_5)(rh.insula_4)(rh.precuneus_2)(rh.transversetemporal_1)
0.00826	9.00E-05	0.80505	0.89447	(rh.fusiform_5)(rh.insula_4)(rh.precuneus_2)(rh.superiortemporal_1)
0.00826	9.00E-05	0.80505	0.89447	(Right-Pallidum)(rh.precuneus_2)(rh.superiortemporal_1)(rh.transversetemporal_1)
0.00826	9.00E-05	0.80505	0.89447	(rh.insula_4)(rh.precuneus_3)(rh.superiortemporal_1)(rh.superiortemporal_6)
0.00876	9.00E-05	0.90975	0.96985	(rh.inferiorparietal_4)(rh.insula_2)(rh.precuneus_2)(rh.superiortemporal_1)
0.00878	9.00E-05	0.787	0.8794	(Right-Putamen)(rh.inferiorparietal_10)(rh.insula_4)(rh.transversetemporal_1)
0.00878	0.0001	0.787	0.8794	(Right-Pallidum)(rh.inferiorparietal_4)(rh.insula_4)(rh.transversetemporal_1)
0.00878	0.0001	0.787	0.8794	(rh.inferiorparietal_10)(rh.insula_2)(rh.insula_4)(rh.transversetemporal_1)
0.00878	0.0001	0.787	0.8794	(Right-Thalamus-Proper)(rh.inferiorparietal_10)(rh.insula_4)(rh.transversetemporal_1)
0.00878	0.0001	0.787	0.8794	(rh.inferiorparietal_10)(rh.insula_4)(rh.isthmuscingulate_2)(rh.transversetemporal_1)
0.00878	0.0001	0.77465	0.86935	(rh.inferiorparietal_10)(rh.inferiorparietal_4)(rh.insula_4)(rh.transversetemporal_1)
0.00878	0.0001	0.77465	0.86935	(rh.middletemporal_4)(rh.precuneus_3)(rh.precuneus_4)(rh.superiortemporal_1)
0.00892	0.0001	0.93502	0.98492	(Right-Pallidum)(Right-Putamen)(rh.insula_2)(rh.superiortemporal_1)
0.00892	0.0001	0.93502	0.98492	(Right-Pallidum)(Right-Thalamus-Proper)(rh.insula_2)(rh.superiortemporal_1)
0.00892	0.0001	0.93502	0.98492	(Right-Pallidum)(rh.insula_2)(rh.superiortemporal_1)
0.00892	0.0001	0.93502	0.98492	(Right-Pallidum)(rh.insula_2)(rh.isthmuscingulate_2)(rh.superiortemporal_1)
0.009	0.00011	0.73285	0.83417	(rh.insula_4)(rh.precuneus_4)(rh.supramarginal_9)(rh.transversetemporal_1)
0.00906	0.00011	0.76895	0.86432	(rh.insula_4)(rh.insula_5)(rh.superiortemporal_1)(rh.transversetemporal_1)
0.00906	0.00011	0.76895	0.86432	(rh.insula_4)(rh.lateraloccipital_10)(rh.precuneus_3)(rh.superiortemporal_3)

0.00923	0.00011	0.85921	0.93467	(rh.insula_5)(rh.precuneus_2)(rh.precuneus_3)(rh.superiortemporal_1)
0.00924	0.00011	0.90253	0.96482	(rh.inferiorparietal_10)(rh.precuneus_3)(rh.superiortemporal_1)
0.00924	0.00011	0.90253	0.96482	(Right-Thalamus-Proper)(rh.insula_2)(rh.insula_4)(rh.precuneus_3)
0.00924	0.00011	0.90253	0.96482	(Right-Thalamus-Proper)(rh.inferiorparietal_10)(rh.precuneus_3)(rh.superiortemporal_1)
0.00924	0.00011	0.90253	0.96482	(rh.isthmuscingulate_2)(rh.precuneus_3)(rh.superiortemporal_1)(rh.superiortemporal_3)
0.00924	0.00011	0.90253	0.96482	(Right-Thalamus-Proper)(rh.insula_4)(rh.isthmuscingulate_2)(rh.precuneus_3)
0.00924	0.00012	0.90253	0.96482	(Right-Putamen)(rh.insula_4)(rh.isthmuscingulate_2)(rh.precuneus_3)
0.00924	0.00012	0.90253	0.96482	(Right-Thalamus-Proper)(rh.precuneus_3)(rh.superiortemporal_1)(rh.superiortemporal_3)
0.00924	0.00012	0.90253	0.96482	(Right-Thalamus-Proper)(rh.insula_4)(rh.precuneus_3)
0.00924	0.00012	0.90253	0.96482	(Right-Pallidum)(rh.insula_4)(rh.lingual_7)(rh.superiortemporal_1)
0.00924	0.00012	0.90253	0.96482	(rh.precuneus_3)(rh.superiortemporal_1)(rh.superiortemporal_3)
0.00924	0.00012	0.90253	0.96482	(rh.insula_4)(rh.precuneus_3)
0.00924	0.00013	0.90253	0.96482	(rh.bankssts_2)(rh.insula_4)(rh.lingual_7)(rh.superiortemporal_1)
0.00924	0.00013	0.90253	0.96482	(Right-Putamen)(rh.precuneus_3)(rh.superiortemporal_1)(rh.superiortemporal_3)
0.00924	0.00013	0.90253	0.96482	(Right-Putamen)(rh.insula_2)(rh.insula_4)(rh.precuneus_3)
0.00924	0.00013	0.90253	0.96482	(rh.insula_2)(rh.insula_4)(rh.precuneus_3)
0.00924	0.00013	0.90253	0.96482	(Right-Putamen)(Right-Thalamus-Proper)(rh.insula_4)(rh.precuneus_3)
0.00924	0.00013	0.90253	0.96482	(Right-Putamen)(rh.inferiorparietal_10)(rh.precuneus_3)(rh.superiortemporal_1)
0.00924	0.00014	0.90253	0.96482	(rh.insula_4)(rh.isthmuscingulate_2)(rh.precuneus_3)
0.00924	0.00014	0.90253	0.96482	(Right-Putamen)(rh.insula_4)(rh.precuneus_3)
0.00924	0.00014	0.90253	0.96482	(rh.insula_2)(rh.insula_4)(rh.isthmuscingulate_2)(rh.precuneus_3)
0.00924	0.00014	0.90253	0.96482	(rh.inferiorparietal_10)(rh.isthmuscingulate_2)(rh.precuneus_3)(rh.superiortemporal_1)
0.00924	0.00014	0.90253	0.96482	(Right-Pallidum)(rh.bankssts_2)(rh.insula_4)(rh.superiortemporal_1)
0.00936	0.00014	0.78169	0.87437	(rh.insula_4)(rh.lingual_7)(rh.precuneus_3)(rh.transversetemporal_1)
0.00936	0.00015	0.78169	0.87437	(rh.inferiorparietal_10)(rh.insula_4)(rh.precuneus_2)(rh.transversetemporal_1)
0.00936	0.00015	0.78169	0.87437	(rh.bankssts_2)(rh.insula_4)(rh.precuneus_3)(rh.transversetemporal_1)
0.00936	0.00015	0.78169	0.87437	(rh.inferiorparietal_10)(rh.insula_4)(rh.superiortemporal_3)(rh.transversetemporal_1)
0.00944	0.00015	0.79422	0.88442	(Right-Caudate)(Right-Thalamus-Proper)(rh.insula_4)(rh.transversetemporal_1)
0.00944	0.00016	0.79422	0.88442	(Right-Caudate)(Right-Putamen)(rh.insula_4)(rh.transversetemporal_1)
0.00944	0.00016	0.79422	0.88442	(rh.inferiortemporal_2)(rh.insula_4)(rh.precuneus_3)(rh.superiortemporal_1)
0.00944	0.00016	0.79422	0.88442	(Right-Caudate)(rh.insula_4)(rh.isthmuscingulate_2)(rh.transversetemporal_1)
0.00947	0.00016	0.86643	0.9397	(rh.inferiorparietal_10)(rh.inferiorparietal_4)(rh.insula_4)(rh.precuneus_3)
0.00947	0.00017	0.86643	0.9397	(rh.inferiorparietal_4)(rh.insula_4)(rh.precuneus_3)(rh.supramarginal_9)
0.00954	0.00017	0.89531	0.9598	(Right-Pallidum)(rh.precuneus_3)(rh.superiortemporal_1)(rh.supramarginal_9)
0.00954	0.00017	0.89531	0.9598	(rh.insula_4)(rh.precuneus_3)(rh.superiortemporal_3)
0.00954	0.00018	0.89531	0.9598	(rh.insula_4)(rh.isthmuscingulate_2)(rh.superiortemporal_1)(rh.supramarginal_9)

0.00954	0.00018	0.89531	0.9598	(Right-Thalamus-Proper)(rh.insula_4)(rh.precuneus_3)(rh.superiortemporal_3)
0.00954	0.00018	0.89531	0.9598	(Right-Putamen)(rh.insula_4)(rh.superiortemporal_1)(rh.supramarginal_9)
0.00954	0.00019	0.89531	0.9598	(Right-Caudate)(rh.insula_2)(rh.insula_4)(rh.superiortemporal_1)
0.00954	0.00019	0.89531	0.9598	(Right-Thalamus-Proper)(rh.insula_4)(rh.superiortemporal_1)(rh.supramarginal_9)
0.00954	0.00019	0.89531	0.9598	(rh.insula_2)(rh.insula_4)(rh.superiortemporal_1)(rh.supramarginal_9)
0.00954	0.0002	0.89531	0.9598	(Right-Caudate)(Right-Putamen)(rh.insula_4)(rh.superiortemporal_1)
0.00954	0.0002	0.89531	0.9598	(rh.insula_4)(rh.isthmuscingulate_2)(rh.precuneus_3)(rh.superiortemporal_3)
0.00954	0.0002	0.89531	0.9598	(Right-Caudate)(Right-Pallidum)(rh.precuneus_3)(rh.superiortemporal_1)
0.00954	0.00021	0.89531	0.9598	(Right-Putamen)(rh.insula_4)(rh.precuneus_3)(rh.superiortemporal_3)
0.00954	0.00021	0.89531	0.9598	(Right-Caudate)(rh.insula_4)(rh.isthmuscingulate_2)(rh.superiortemporal_1)
0.00954	0.00022	0.89531	0.9598	(rh.insula_2)(rh.insula_4)(rh.precuneus_3)(rh.superiortemporal_3)
0.00954	0.00022	0.89531	0.9598	(Right-Caudate)(Right-Thalamus-Proper)(rh.insula_4)(rh.superiortemporal_1)
0.00954	0.00023	0.89531	0.9598	(Right-Caudate)(rh.insula_4)(rh.superiortemporal_1)
0.00954	0.00023	0.89531	0.9598	(rh.insula_4)(rh.superiortemporal_1)(rh.supramarginal_9)
0.00959	0.00024	0.72202	0.82412	(rh.fusiform_5)(rh.inferiortemporal_2)(rh.precuneus_3)(rh.superiortemporal_1)
0.00963	0.00024	0.87365	0.94472	(rh.inferiorparietal_10)(rh.insula_4)(rh.precuneus_3)(rh.superiortemporal_3)
0.00963	0.00025	0.87365	0.94472	(rh.insula_4)(rh.parahippocampal_2)(rh.precuneus_2)(rh.superiortemporal_1)
0.00963	0.00026	0.87365	0.94472	(rh.inferiorparietal_10)(rh.insula_4)(rh.parahippocampal_2)(rh.superiortemporal_1)
0.00963	0.00026	0.87365	0.94472	(rh.inferiorparietal_4)(rh.insula_4)(rh.parahippocampal_2)(rh.superiortemporal_1)
0.00969	0.00027	0.88809	0.95477	(rh.insula_4)(rh.parahippocampal_2)(rh.superiortemporal_1)
0.00969	0.00028	0.88809	0.95477	(Right-Putamen)(rh.insula_4)(rh.parahippocampal_2)(rh.superiortemporal_1)
0.00969	0.00029	0.88809	0.95477	(rh.bankssts_2)(rh.inferiorparietal_10)(rh.insula_4)(rh.superiortemporal_1)
0.00969	0.00029	0.88809	0.95477	(rh.inferiorparietal_4)(rh.precuneus_3)(rh.superiortemporal_1)(rh.supramarginal_9)
0.00969	0.0003	0.88809	0.95477	(rh.insula_2)(rh.insula_4)(rh.parahippocampal_2)(rh.superiortemporal_1)
0.00969	0.00031	0.88809	0.95477	(Right-Pallidum)(rh.parahippocampal_2)(rh.precuneus_3)(rh.superiortemporal_1)
0.00969	0.00032	0.88809	0.95477	(Right-Caudate)(rh.inferiorparietal_4)(rh.precuneus_3)(rh.superiortemporal_1)
0.00969	0.00033	0.88809	0.95477	(Right-Thalamus-Proper)(rh.insula_4)(rh.parahippocampal_2)(rh.superiortemporal_1)
0.00969	0.00034	0.88809	0.95477	(rh.inferiorparietal_10)(rh.insula_4)(rh.lingual_7)(rh.superiortemporal_1)
0.00969	0.00036	0.88809	0.95477	(rh.insula_4)(rh.superiortemporal_1)(rh.superiortemporal_3)(rh.supramarginal_9)
0.00969	0.00037	0.88809	0.95477	(Right-Caudate)(rh.insula_4)(rh.superiortemporal_1)(rh.superiortemporal_3)
0.00969	0.00038	0.88809	0.95477	(rh.insula_4)(rh.isthmuscingulate_2)(rh.parahippocampal_2)(rh.superiortemporal_1)
0.00969	0.0004	0.88809	0.95477	(Right-Pallidum)(rh.inferiorparietal_10)(rh.insula_4)(rh.superiortemporal_1)
0.00971	0.00042	0.88087	0.94975	(rh.inferiorparietal_4)(rh.parahippocampal_2)(rh.precuneus_3)(rh.superiortemporal_1)
0.00971	0.00043	0.88087	0.94975	(Right-Putamen)(rh.inferiorparietal_10)(rh.insula_4)(rh.precuneus_3)
0.00971	0.00045	0.88087	0.94975	(Right-Thalamus-Proper)(rh.inferiorparietal_10)(rh.insula_4)(rh.precuneus_3)
0.00971	0.00048	0.88087	0.94975	(Right-Caudate)(rh.inferiorparietal_4)(rh.insula_4)(rh.superiortemporal_1)

0.00971	0.0005	0.88087	0.94975	(rh.inferiorparietal_4)(rh.insula_4)(rh.superiortemporal_1)(rh.supramarginal_9)
0.00971	0.00053	0.88087	0.94975	(rh.inferiorparietal_10)(rh.insula_4)(rh.isthmuscingulate_2)(rh.precuneus_3)
0.00971	0.00056	0.88087	0.94975	(rh.insula_4)(rh.parahippocampal_2)(rh.superiortemporal_1)(rh.superiortemporal_3)
0.00971	0.00059	0.88087	0.94975	(rh.inferiorparietal_10)(rh.insula_2)(rh.insula_4)(rh.precuneus_3)
0.00971	0.00063	0.88087	0.94975	(rh.inferiorparietal_10)(rh.insula_4)(rh.precuneus_3)
0.00971	0.00067	0.88087	0.94975	(Right-Caudate)(rh.insula_4)(rh.precuneus_2)(rh.superiortemporal_1)
0.0098	0.00071	0.77617	0.86935	(rh.insula_2)(rh.insula_4)(rh.lateraloccipital_10)(rh.precuneus_3)
0.0098	0.00077	0.77617	0.86935	(Right-Putamen)(rh.insula_4)(rh.lateraloccipital_10)(rh.precuneus_3)
0.0098	0.00083	0.77617	0.86935	(Right-Thalamus-Proper)(rh.insula_4)(rh.lateraloccipital_10)(rh.precuneus_3)
0.0098	0.00091	0.77617	0.86935	(rh.insula_4)(rh.lateraloccipital_10)(rh.precuneus_3)
0.0098	0.001	0.77617	0.86935	(rh.insula_4)(rh.isthmuscingulate_2)(rh.lateraloccipital_10)(rh.precuneus_3)
0.00992	0.00111	0.75812	0.85427	(rh.inferiorparietal_10)(rh.insula_4)(rh.lateraloccipital_10)(rh.precuneus_3)
0.00996	0.00125	0.78873	0.8794	(rh.inferiorparietal_4)(rh.precuneus_3)(rh.superiortemporal_1)(rh.transversetemporal_1)
0.00996	0.00143	0.78873	0.8794	(rh.bankssts_2)(rh.inferiorparietal_4)(rh.insula_4)(rh.transversetemporal_1)
0.00996	0.00167	0.78873	0.8794	(rh.inferiorparietal_4)(rh.superiortemporal_3)(rh.supramarginal_9)(rh.transversetemporal_
0.00996	0.002	0.78873	0.8794	(rh.inferiorparietal_4)(rh.superiortemporal_1)(rh.superiortemporal_3)(rh.transversetempo
0.00996	0.0025	0.78873	0.8794	(rh.parahippocampal_1)(rh.precuneus_3)(rh.superiortemporal_1)(rh.superiortemporal_3)
0.00996	0.00333	0.78873	0.8794	(rh.inferiorparietal_4)(rh.insula_4)(rh.isthmuscingulate_1)(rh.precuneus_3)
0.00996	0.005	0.78873	0.8794	(rh.inferiorparietal_10)(rh.insula_4)(rh.transversetemporal_1)
0.00996	0.01	0.78873	0.8794	(rh.inferiorparietal_4)(rh.insula_4)(rh.lingual_7)(rh.transversetemporal_1)

l_1)

_1)
ɔral_1)
