# Supplementary material for: Good neighbors, bad neighbors: the frequent network neighborhood mapping of the hippocampus enlightens several structural factors of the human intelligence on a 414-subject cohort
Source: Sci Rep. 2020 Jul 20;10:11967. doi: 10.1038/s41598-020-68914-2 (PMC7371878; doi:10.1038/s41598-020-68914-2)
Supplement: Supplementary file 12 — Supplementary Information 12. [file 41598_2020_68914_MOESM12_ESM.pdf]

p-value	Holm-Bonferroni	frequency_upper	frequency_lower	name
1.00E-05	0	0.74368	0.90452	(lh.inferiorparietal_4)(lh.inferiorparietal_5)(rh.insula_4)(rh.superiortemporal_1)
1.00E-05	0	0.77617	0.92462	(lh.inferiorparietal_4)(lh.inferiorparietal_5)(lh.precuneus_11)(rh.superiortemporal_1)
1.00E-05	0	0.75451	0.90955	(lh.inferiorparietal_4)(lh.precuneus_11)(rh.insula_4)(rh.superiortemporal_1)
2.00E-05	0	0.76534	0.91457	(lh.inferiorparietal_4)(lh.inferiorparietal_5)(rh.precuneus_3)(rh.superiortemporal_1)
3.00E-05	0	0.77617	0.9196	(lh.inferiorparietal_4)(lh.precuneus_11)(rh.precuneus_3)(rh.superiortemporal_1)
4.00E-05	0	0.84838	0.96482	(lh.inferiorparietal_5)(lh.precuneus_11)(rh.insula_4)(rh.superiortemporal_1)
4.00E-05	0	0.75812	0.90452	(lh.inferiorparietal_4)(lh.inferiorparietal_5)(lh.precuneus_11)(rh.insula_4)
4.00E-05	0	0.787	0.92462	(Right-Pallidum)(lh.inferiorparietal_4)(lh.precuneus_11)(rh.superiortemporal_1)
4.00E-05	0	0.77256	0.91457	(lh.inferiorparietal_4)(lh.inferiorparietal_5)(rh.inferiorparietal_4)(rh.superiortemporal_1)
4.00E-05	0	0.77256	0.91457	(lh.inferiorparietal_4)(lh.inferiorparietal_5)(lh.lingual_6)(rh.superiortemporal_1)
4.00E-05	0	0.77978	0.9196	(lh.inferiorparietal_4)(lh.lingual_6)(lh.precuneus_11)(rh.superiortemporal_1)
4.00E-05	0	0.77978	0.9196	(Right-Pallidum)(lh.inferiorparietal_4)(lh.inferiorparietal_5)(rh.superiortemporal_1)
6.00E-05	0	0.79783	0.92965	(Left-Putamen)(lh.inferiorparietal_4)(lh.precuneus_11)(rh.superiortemporal_1)
6.00E-05	0	0.79061	0.92462	(lh.inferiorparietal_4)(lh.insula_2)(lh.precuneus_11)(rh.superiortemporal_1)
6.00E-05	0	0.79061	0.92462	(Left-Putamen)(lh.inferiorparietal_4)(lh.inferiorparietal_5)(rh.superiortemporal_1)
6.00E-05	0	0.79061	0.92462	(lh.inferiorparietal_4)(lh.precuneus_11)(rh.insula_2)(rh.superiortemporal_1)
6.00E-05	0	0.78339	0.9196	(lh.inferiorparietal_4)(lh.inferiorparietal_5)(lh.insula_2)(rh.superiortemporal_1)
6.00E-05	0	0.78339	0.9196	(lh.inferiorparietal_4)(lh.precuneus_11)(rh.inferiorparietal_4)(rh.superiortemporal_1)
6.00E-05	0	0.78339	0.9196	(lh.inferiorparietal_4)(lh.inferiorparietal_5)(rh.insula_2)(rh.superiortemporal_1)
6.00E-05	0	0.78339	0.9196	(lh.inferiorparietal_4)(lh.inferiorparietal_5)(rh.precuneus_2)(rh.superiortemporal_1)
7.00E-05	0	0.88087	0.9799	(Right-Pallidum)(lh.inferiorparietal_5)(lh.precuneus_11)(rh.superiortemporal_1)
7.00E-05	0	0.83755	0.95477	(lh.inferiorparietal_5)(rh.insula_4)(rh.precuneus_3)(rh.superiortemporal_1)
8.00E-05	0	0.8917	0.98492	(Left-Putamen)(lh.inferiorparietal_5)(lh.precuneus_11)(rh.superiortemporal_1)
9.00E-05	0	0.76534	0.90452	(Right-Amygdala)(lh.inferiorparietal_4)(lh.precuneus_11)(rh.superiortemporal_1)
9.00E-05	0	0.77256	0.90955	(lh.inferiorparietal_4)(rh.inferiorparietal_4)(rh.precuneus_3)(rh.superiortemporal_1)
9.00E-05	0	0.77256	0.90955	(Left-Thalamus-Proper)(lh.inferiorparietal_4)(rh.insula_4)(rh.superiortemporal_1)
9.00E-05	0	0.77256	0.90955	(Right-Putamen)(lh.inferiorparietal_4)(rh.insula_4)(rh.superiortemporal_1)
9.00E-05	0	0.77256	0.90955	(Left-Putamen)(lh.inferiorparietal_4)(rh.insula_4)(rh.superiortemporal_1)
9.00E-05	0	0.77256	0.90955	(lh.inferiorparietal_4)(rh.insula_2)(rh.insula_4)(rh.superiortemporal_1)
9.00E-05	0	0.77256	0.90955	(lh.inferiorparietal_4)(rh.insula_4)(rh.superiortemporal_1)
9.00E-05	0	0.77256	0.90955	(lh.inferiorparietal_4)(rh.insula_4)(rh.isthmuscingulate_2)(rh.superiortemporal_1)
9.00E-05	0	0.77256	0.90955	(Right-Thalamus-Proper)(lh.inferiorparietal_4)(rh.insula_4)(rh.superiortemporal_1)
9.00E-05	0	0.77256	0.90955	(lh.inferiorparietal_4)(lh.isthmuscingulate_3)(rh.insula_4)(rh.superiortemporal_1)
